# Asymmetric Cyclopropanation and Epoxidation via a Catalytically Formed Chiral Auxiliary

**DOI:** 10.1002/anie.202113925

**Published:** 2022-02-01

**Authors:** Mikus Puriņš, Jerome Waser

**Affiliations:** ^1^ Laboratory of Catalysis and Organic Synthesis and NCCR Catalysis Institut des Sciences et Ingénierie Chimique Ecole Polytechnique Fédérale de Lausanne 1015 Lausanne Switzerland

**Keywords:** Asymmetric Synthesis, Cyclopropanation, Cyclopropanol, Epoxidation, Rubottom Oxidation

## Abstract

For the enantioselective diversification of a single starting material, a different chiral catalyst is usually required for each transformation. Herein, we extend the concept of catalytically formed chiral auxiliary from hydrogenation to the asymmetric cyclopropanation and epoxidation of tetra‐substituted olefins, alleviating the need for different chiral catalysts in the alkene functionalization step. The chiral auxiliary is catalytically constructed from propargylic amines in a Pd‐catalyzed enantioselective carboetherification step using a commercially available trifluoroacetaldehyde hemiacetal tether. The installed auxiliary is then controlling the stereochemistry of the cyclopropanation and the epoxidation using standard highly reactive reagents to give enantioenriched spirocyclic aminomethylcyclopropanols and α‐amino‐α‐hydroxy ketones.

## Graphical abstract

Catalytic asymmetric installation of a chiral auxiliary gives access to enantioenriched oxazolidines bearing a tetra‐substituted olefin from readily available propargylic amines. In this work, we extend the use of the catalytically formed chiral auxiliary, previously limited to hydrogenation, to asymmetric cyclopropanation and epoxidation using simple and robust reaction conditions for accessing highly substituted products.

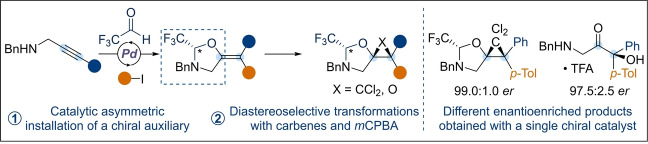

Asymmetric catalysis has been developed intensively in the last decades and is currently one of the most efficient methods for accessing enantioenriched compounds essential for the fragrance, agrochemical and pharmaceutical industries. However, catalytic asymmetric reactions are sensitive to the substrate structure and often require specialized reagents or additives.^[1]^ As a consequence, only a few privileged catalysts can be used to perform chemically distinct transformations over a broad range of substrates.[^2^, ^3^] To diversify a single compound, each transformation requires a different, fine‐tuned chiral catalyst (Scheme 1A). In this respect, the use of chiral auxiliaries offers some advantages—the reaction conditions are usually robust and highly reactive intermediates are tolerated. In addition, control of stereoselectivity is often independent of the reaction considered, thus the same chiral auxiliary can be used to impart stereocontrol in multiple, mechanistically unrelated transformations.^[4]^ For example, Evans’ chiral auxiliary was originally developed for stereoselective aldol reactions, but has then found a myriad of additional applications ‐ asymmetric alkylation, halogenation, hydroxylation, 1,4‐additions, Diels–Alder, [3+2] cycloaddition and carbonyl reduction among others.^[5]^ However, this approach still suffers from the need of a stoichiometric amount of enantiopure molecules, as well as low step and atom economy.

**Scheme 1:**
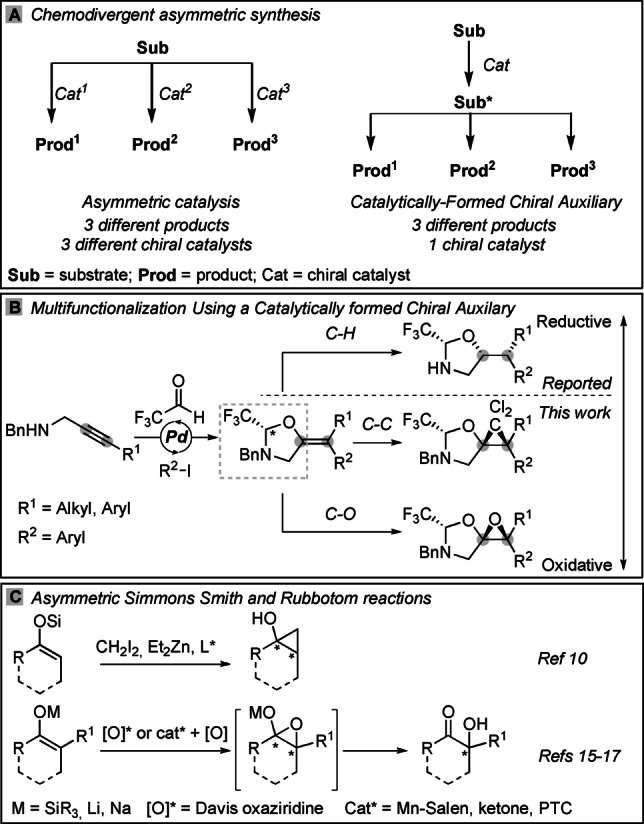
A) Chemodivergent asymmetric synthesis. B) Previous work: hydrogenation; and targeted transformations: cyclopropanation and epoxidation. C) Reported approaches for enantioselective cyclopropanation and epoxidation of enol‐ether substrates.

To overcome the disadvantages associated with the use of chiral auxiliaries, we recently introduced the concept of catalytically formed chiral auxiliaries (Scheme 1A).[^6^, ^7^] In this approach, the auxiliary is generated using asymmetric catalysis in a synthetically useful step and the formed auxiliary enables diastereoselective functionalization of the product.

In our previous work, a tethered palladium‐catalyzed carboetherification of propargylic amines was used to give tetra‐substituted alkene‐bound oxazolidines with high enantioselectivity (Scheme 1B).^[6]^ A simple diastereoselective hydrogenation led then to protected amino alcohols. To access more complex building blocks, we considered cyclopropanation and epoxidation, which would give difficult to access highly substituted compounds via double C−C or C−O bond formation. The racemic cyclopropanation of enol ethers is well‐established^[8]^ and asymmetric variations have been achieved using Simmons–Smith reactions with either a chiral auxiliary^[9]^ or a dipeptide ligand (Scheme 1C).^[10]^ However, in these reports tetra‐substituted substrates were not tolerated. Charette's conditions^[11]^ have also been applied to a trisubstituted enol ether using an alcohol as directing group.^[12]^ In our case, the products would contain an aminomethyl cyclopropanol scaffold, which has been reported in almost 300 WO patents.^[13]^ In contrast, the epoxidation of silyl enol ethers is a well‐documented step in the Rubottom oxidation (Scheme 1C).^[14]^ The asymmetric variant can be done with chiral electrophilic oxygen sources, such as the Davis oxaziridine,^[15]^ or oxidants combined with a chiral Mn‐salen complex^[16]^ or a ketone based organocatalyst.^[17]^ However, with both approaches the geometry of the enol ether/enolate is crucial to the stereochemical outcome and non‐biased tetra‐substituted substrates are challenging to generate with high geometrical purity. The collapse of the epoxy‐enol ethers provides an α‐hydroxy ketone motif, which is commonly found in various natural products and has been applied in medicinal chemistry.^[18]^

Herein, we report the successful use of catalytically formed chiral oxazolidine auxiliaries for asymmetric C−C and C−O bond formation. This was achieved using cyclopropanation with free dichlorocarbenes under phase transfer conditions and a Rubottom oxidation, both processes occurring with high facial selectivity. The dichlorocyclopropanol derivatives obtained with the former approach are well established building blocks in synthetic chemistry since the seminal works of Stork and Wenkert, especially for their use in ring expansion reactions.^[19]^ Nevertheless, the synthesis of geminally diarylated derivatives is unprecedented. The highly substituted products obtained using our strategy are currently beyond the reach of direct enantioselective catalysis.

The oxazolidine starting materials were prepared using the enantioselective tethered carboetherification of propargylic amines developed by our group,^[6]^ which could be easily scaled up to a 2.0 mmol scale (Scheme 2, for full details, see the Supporting Information, sections B.5 and B.6). It was found that the *er* of the model oxazolidine product **2** **a** can be easily upgraded by a simple crystallization to give the product with 99.0 : 1.0 *er*.

**Scheme 2:**
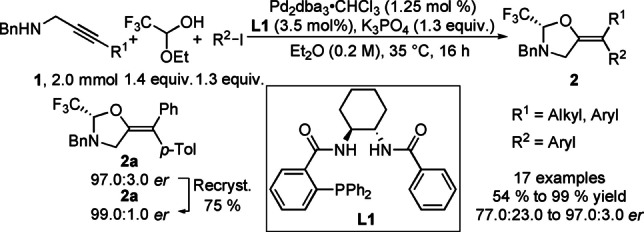
Pd‐catalyzed enantioselective carboetherification of propargylic amines **1** forming the oxazolidine chiral auxiliary.

Various cyclopropanation approaches were first examined. The Simmons–Smith reaction[^20^, ^21^] failed to deliver the desired product (Table 1, Entry 1, for full details, see the Supporting Information, section C). The combination of ethyldiazoacetate with a Rh catalyst^[22]^ did not afford the cyclopropane product as well (Entry 2). In this case, mainly the dimerization products were observed. The low reactivity of the C−C double bond in the oxazolidine **2** **a** can be attributed to the steric bulk originating from the four substituents. Therefore, most mild catalytic methods based on the formation of metal‐stabilized carbenes or carbenoids, for which enantioselective versions exist or could be developed, failed for this class of substrates. However, due to the nature of the stereoinduction using chiral‐auxiliaries based on strong covalent bonds, harsher reaction conditions can also be considered. Thus, the generation of highly reactive free carbenes was investigated. Using conditions known to generate difluorocarbene^[23]^ no conversion was observed (Entry 3). In contrast, the generation of dichlorocarbene with CHCl_3_, aqueous NaOH and cetyltrimethyl ammonium bromide (CTAB) as the phase transfer catalyst^[19g]^ provided the product in 84 % NMR yield with 19 : 1 *dr* (Entry 4, 75 % isolated yield of the major diastereoisomer). Using the analogous conditions with CHBr_3_ instead, the corresponding product was not observed (Entry 5).

**Table 1:** Screening of reaction conditions for the stereoselective cyclopropanation reaction of oxazolidine **2** **a**.

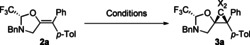			
			
Entry	Conditions	Reactive intermediate	Results
1	2.0 equiv CH_2_I_2_, 2.5 equiv. Et_2_Zn	mixture	ND^[a]^
2	N_2_CHCO_2_Et, Rh cat.	Rh=CHCO_2_Et	ND
3	TMSCF_3_, NaI	:CF_2_	ND
4	CHCl_3_, NaOH, CTAB	:CCl_2_	84 % yield,^[b]^ 19 : 1 *dr* ^[c]^
5	CHCl_3_, NaOH, CTAB	:CBr_2_	ND

[a] ND: not detected. [b] NMR yield. 75 % yield of the pure major diastereoisomer was isolated after column chromatography. [c] Determined from the crude ^19^F NMR.

We then investigated the scope of the reaction (Scheme 3). For scale‐up, we used a recrystallized sample of **2** **a** with 99.0 : 1.0 *er* without a significant drop in the yield of **3** **a** on a 1.0 mmol scale. We first tested substrates with variation on the substituent *cis* to oxygen (products **3** **b**–**3** **g**). The diastereoisomer of the model product **3** **b** was obtained in 65 % yield. Both an electron‐poor and an electron‐rich substituent in the *para* position were tolerated to give products **3** **c** and **3** **d** in 66 % and 76 % yield respectively. Alkyl substituted cyclopropanes **3** **e**–**3** **g** were obtained in 67–82 % yield.

**Scheme 3:**
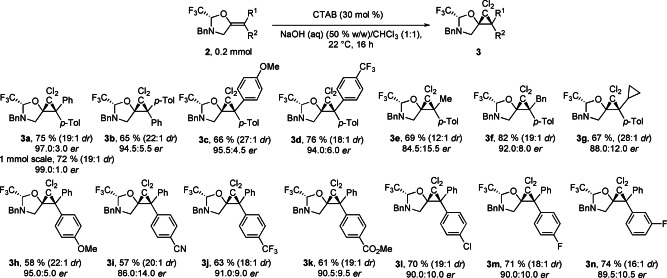
Scope of the asymmetric dichlorocyclopropanation. The *dr* is indicated as measured in the crude ^19^F NMR. The yields indicated are for the pure major diastereoisomer isolated after column chromatography. In all cases the *er* of the product was matching that of the starting material.

Variation of the *trans* substituent on the double bond was then examined (products **3** **h**–**3** **n**). An electron donating substituent in the *para* position was tolerated to give product **3** **h** in 58 % yield. Different electron withdrawing functional groups on the *para* position can be used, including cyano, trifluoromethyl and an ester giving products **3** **i**–**3** **k** in 57–63 % yield. Halogen substituted products **3** **l**–**3** **n** could also be obtained in 70–74 % yield. In all cases the *er* of the product matched the *er* of the starting material. The *dr* could be determined from the crude ^19^F NMR—in most cases it was around 20 : 1, with the lowest being 12 : 1 with the smallest Me substituent in product **3** **f**. Heterocyclic substitution was not tolerated under these reaction conditions (see the Supporting Information, section D.5).

Next, we turned our attention to the epoxidation of the model substrate **2** **a**. In analogy to the Rubottom oxidation of silyl enol ethers, we used *m*CPBA in a biphasic aqueous NaHCO_3_/DCM^[24]^ system (Table 2). We were pleased to see the formation of the desired product in 75 % NMR yield with 17 : 1 *dr* (Entry 1). However, in this case it was not possible to separate the two diastereoisomers. When performing solvolysis of the epoxide and the tether, the stereocenters at the C2 and C5 positions would be lost. At this point, the minor diastereoisomer would lead to the opposite stereochemistry on the tertiary alcohol position. This means that the final *er* of the α‐hydroxy ketone is dependent both on the *er* of the starting material, and the *dr* of the epoxidation, and therefore obtaining high *dr* is essential. Adding *m*CPBA as a solution over 10 minutes increased the yield to 87 %, without affecting the *dr* (Entry 2). A solvent switch to Et_2_O or toluene increased the *dr* up to >39 : 1, with toluene providing the product in 85 % yield (Entries 3, 4). In a control reaction without saturated aqueous bicarbonate, the product was obtained in 98 % yield and >39 : 1 dr, with very clean crude NMR profile (Entry 5). However, upon scaling the reaction to 0.20 mmol, some issues with conversion emerged (Entry 6). To eliminate the variation in the water content, the *m*CPBA solution was dried over Na_2_SO_4_ giving the desired product in slightly increased yield (Entry 7). Finally, to maximize the conversion, the reaction was performed at 0 °C for 1 hour, then at 22 °C for an additional 1 hour. The reaction could also be scaled to 0.40 mmol with good reproducibility (Entries 8, 9).

**Table 2:** Optimization of the epoxidation of **2** **a**.

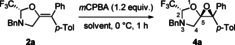					
					
Entry	Scale [mmol]	Solvent	NaHCO_3(aq)_	**4** **a** yield [%]^[a]^	*dr* ^[b]^
1^[c]^	0.05	DCM	+	75	17 : 1
2	0.05	DCM	+	87	21 : 1
3	0.05	Et_2_O	+	27	39 : 1
4	0.05	Toluene	+	85	>39 : 1
5	0.05	Toluene	−	98	>39 : 1
6	0.20	Toluene	−	83–98	>39 : 1
7^[d]^	0.20	Toluene	−	93–97	>39 : 1
8^[d,e]^	0.20	Toluene	−	97–99	>39 : 1
9^[d,e]^	0.40	Toluene	−	96–98	>39 : 1

[a] NMR yields. [b] Determined from the crude ^19^F NMR. [c] In this entry, *m*CPBA was added as a solid. In other entries, *m*CPBA was added as a solution in the corresponding solvent over 10 minutes using a syringe pump. [d] The *m*CPBA solution was dried over anhydrous Na_2_SO_4_. [e] The reaction was performed for 1 h at 0 °C, then 1 h at 22 °C.

Since epoxide **4** **a** was obtained in almost quantitative yield and was also sensitive to chromatographic purification, we performed the tether cleavage directly on the crude product in the presence of TFA in methanol (Scheme 4). The obtained product **5** **a** was more stable in the salt form. We then investigated the scope of the reaction. In this case, since we do not separate the two diastereomers before solvolysis of the tether, we observe some deterioration of the *er*. The model product **5** **a** was obtained in 80 % yield with 96.0 : 4.0 *er*. The reaction could be upscaled to 2.0 mmol using a recrystallized sample of **2** **a** providing the product in 80 % yield and 97.5 : 2.5 *er*. The enantiomer **ent‐5** **a** could be also obtained in similar yield and *er*. Electron‐rich substituents destabilized the products, thus product **5** **c** with two electron donating groups was not obtained. In contrast, oxazolidine **2** **d** with an electron withdrawing group was less reactive, requiring to increase the amount of *m*CPBA to 1.5 equivalents to give product **5** **d** in 60 % yield and 93.5 : 6.5 *er*. Alkyl substituted ketones **5** **e**–**5** **g** were obtained in 55–73 % yields, with a higher drop in the *er* (lower *dr* in the first step) in the case of the smallest methyl group. Various functional groups in the *para* position of the *trans* substituent of olefins **2** were tolerated, including a nitrile, an ester or halogens to give products **5** **h**–**5** **m** in 45–83 % yield.^[25]^ A fluorine substituent was tolerated in *para*, *meta* and *ortho* position to give products **5** **m**–**5** **o** in 45–71 % yields. Finally, a pyridine substituted α‐hydroxy ketone **5** **p** could be obtained in 36 % yield and 75.0 : 25.0 *er*.

**Scheme 4:**
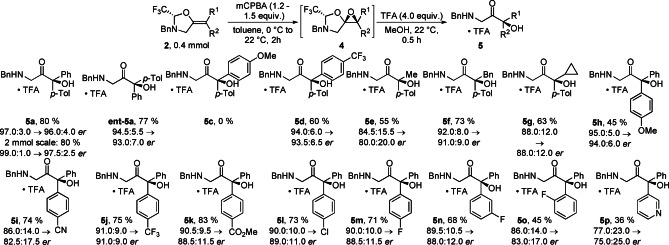
Scope of the asymmetric epoxidation/tether solvolysis. *er* are indicated as: *er* of the starting material→*er* of the product.

Products modification allowed to further diversify the structure of the obtained compounds (Scheme 5). For the dichlorocyclopropane **3** **a**, the benzyl protecting group could be easily cleaved under heterogeneous hydrogenation conditions to give product **6** in 75 % yield.

**Scheme 5:**
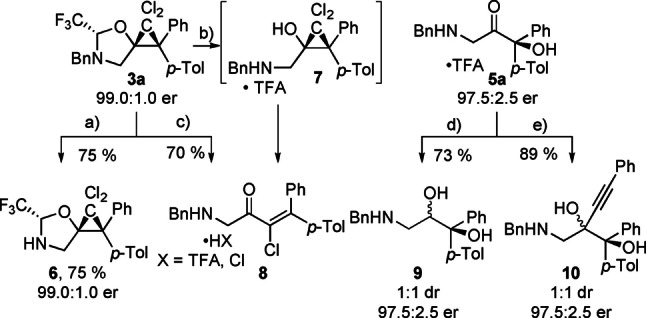
Product modifications. Conditions: a) Pd(OH)_2_ (20 mol %), MeOH/AcOH (2 : 1), 22 °C, 16 h; b) TFA (4.0 equiv.), HFIP, 22 °C, 16 h; c) MeOH, 60 °C, 16 h; d) NaBH_4_ (4.0 equiv.), MeOH, 0 °C, 2 h; e) *n*BuLi (6.0 equiv,), phenylacetylene (6.0 equiv.),THF, 0 to 22 °C, 2 h.

It was possible to remove the tether from product **3** **a** using a TFA/HFIP system. The amino alcohol product was detected in the crude mixture, but upon basification, or any attempts of chromatographic separation (for full details see the Supporting Information, section D.6) of the salt **7**, degradation was observed. The products of the decomposition were identified as a mixture of geometrical isomers of enone **8**, presumably formed from a ring opening reaction of **7**.[^19a^, ^19b^, ^19h^, ^19i^] Interestingly, a thermal ring opening was observed by heating product **3** **a** in methanol, to give the same enone **8** as a single isomer in 70 % yield.

The carbonyl group in product **5** **a** was easily reduced with NaBH_4_, to give the aminodiol **9** in 73 % yield and 1 : 1 *dr*. The addition of lithium phenyl acetylide provided the diol **10** with an 89 % yield and 1 : 1 *dr*. Both **9** and **10** were now stable even with a non‐protonated amine.

In summary, we have shown that a catalytically formed oxazolidine chiral auxiliary can be used to control the facial selectivity of chemically distinct transformations. In this work we achieved C−C and C−O bond formation via diastereoselective cyclopropanation and epoxidation. The cyclopropanation was realized using a free carbene, and the asymmetric epoxidation with *m*CPBA. This approach allows the asymmetric modification of fully substituted enol ethers, a class of substrates that could not be used in current state‐of‐the‐art enantioselective catalysis. The concept of combining catalytic enantioselective chiral auxiliary formation and a diastereoselective transformation can be expected to be the basis of many asymmetric diversity oriented synthetic strategies in the future.^[26]^

## Conflict of interest

The authors declare no conflict of interest.

## Supporting information

As a service to our authors and readers, this journal provides supporting information supplied by the authors. Such materials are peer reviewed and may be re‐organized for online delivery, but are not copy‐edited or typeset. Technical support issues arising from supporting information (other than missing files) should be addressed to the authors.

Supporting Information

Supporting Information
