# Supplementary material for: Asymmetric Cyclopropanation and Epoxidation via a Catalytically Formed Chiral Auxiliary
Source: Angew Chem Int Ed Engl. 2022 Feb 1;61(11):e202113925. doi: 10.1002/anie.202113925 (PMC9306854; doi:10.1002/anie.202113925)
Supplement: Supplementary file 2 — Supporting Information [file ANIE-61-0-s002.pdf]

Angewandte Chemie
Eine Zeitschrift der Gesellschaft Deutscher Chemiker

Supporting Information

Asymmetric Cyclopropanation and Epoxidation via a Catalytically Formed Chiral Auxiliary

*M. Puriņš, J. Waser**

Supporting Information for

"Asymmetric Cyclopropanation and Epoxidation via a Catalytically Formed Chiral Auxiliary"

Mikus Puriņš and Jerome Waser*

Laboratory of Catalysis and Organic Synthesis, Ecole Polytechnique Fédérale de Lausanne, EPFL, SB ISIC LCSO, BCH 1402, 1015 Lausanne (Switzerland)

*Correspondence to: jerome.waser@epfl.ch

Table of Contents

A. General Information S3

B. Synthesis of the Starting Materials and Ligands S4

B.1. Synthesis of the Propargylic Amines Precursor **S2** S4

B.2. Synthesis of the Propargylic Amines S4

B.4. Synthesis of the Ligand **L1** S8

B.5. General Procedure for the Enantioselective Carboetherification of Propargylic Amines S10

B.6. Characterization of Products of the Enantioselevtive Carboetherification S10

C. Optimization Studies S16

C.1. Stereoselective cyclopropanation S16

C.2. Stereoselective epoxidation S18

D. Asymmetric cyclopropanation and epoxidation S20

D.1. General Procedure for the Asymmetric cyclopropanation of the Tetrasubstituted Olefins. S20

D.2. Characterization of the cyclopropanation products S20

D.3. General Procedure for the Asymmetric Epoxidation/Solvolysis of the Tetrasubstituted Olefins S26

D.4. Characterization of the oxidized products S26

D.5. Unsuccessful substrates. S32

D.6. Product modifications S32

E. X-Ray Crystallographic Data S37

F. References S38

G. NMR Spectra S40

G.1.Cyclopropanation products S40

G.2.Epoxidation products S61

G.3.Product modifications S84

H. HPLC Traces S98

H.1.Starting materials S98

H.2.Cyclopropanation products S115

H.3.Epoxidation products S130

H.4.Product modifications S145

A. General Information

The NMR spectra were recorded on a Brucker DPX-400 spectrometer at 400 MHz for ^{1}H, 101 MHz for ^{13}C, 376 MHz for ^{19}F and 162 MHz for ^{31}P. The chemical shift (δ) for ^{1}H and ^{13}C are given in ppm relative to residual signals of the solvents (chloroform-d - 7.26 ppm ^{1}H NMR and 77.16 ppm ^{13}C NMR; methanol-d4 3.31 ppm ^{1}H NMR and 49.0 ppm ^{13}C NMR; dmso-d6 2.50 ppm ^{1}H NMR and 39.52 ppm ^{13}C NMR). Carbon spectra have been measured using broadband {^{1}H} decoupling. Coupling constants are given in Hertz. The following abbreviations are used to indicate the multiplicity: s, singlet; d, doublet; q, quartet; m, multiplet; bs, broad signal; app, apparent. Infrared spectra were recorded on a JASCO FT-IR B4100 spectrophotometer with an ATR PRO410-S and a ZnSe prisma and are reported as cm^{-1} (w = weak, m = medium, s = strong, br = broad). High resolution mass spectrometric measurements were performed by the mass spectrometry service of ISIC at the EPFL on a MICROMASS (ESI) Q-TOF Ultima API. The raw data obtained from the Q-TOF Waters instrument does not take into account the mass of the electron for the ion, the obtained raw data has been therefore corrected by removing the mass of the electron (5 mDa). The diffraction data for crystal structures were collected by mass spectrometry service of ISIC at the EPFL at low temperature using Cu (323) or Mo (520) K_a radiation on a Rigaku SuperNova dual system in combination with Atlas type CCD detector. The data reduction and correction were carried out by *CrysAlis^{Pro}* (Rigaku Oxford Diffraction, release 1.171.40.68a, **2019**). The solutions and refinements were performed by *SHELXT*[1] and *SHELXL*[2], respectively. The crystal structures were refined using full-matrix least-squares based on F^2 with all non-H atoms defined in anisotropic manner. Hydrogen atoms were placed in calculated positions by means of the “riding” model. Yields of isolated products refer to materials of >95% purity as determined by ^{1}H NMR.

The authors are indebted to the team of the research support service of ISIC at EPFL, particularly to the NMR, X-Ray, and the High Resolution Mass Spectrometry Units.

General Procedures. All reactions were set up under a nitrogen atmosphere in oven-dried glassware using standard Schlenk techniques, unless otherwise stated. Synthesis grade solvents were used as purchased; anhydrous solvents (THF, Et_2O, Toluene and DCM) were taken from a commercial SPS solvent dispenser (H_2O content < 10 ppm, *Karl-Fischer* titration). Chromatographic purification of products was accomplished using flash chromatography (FC) on SiliaFlash P60 silica gel (230 - 400 mesh). For thin layer chromatography (TLC) analysis throughout this work, Pre-coated TLC sheets ALUGRAM® Xtra SIL G/UV_{254} were employed, using UV light as the visualizing agent and basic aqueous potassium permanganate ($KMnO_4$) stain solutions, and heat as developing agents. Organic solutions were concentrated under reduced pressure on a Büchi rotatory evaporator.

Determination of Enantiomeric Purity: HPLC analysis on chiral stationary phase was performed on a Agilent Acquity instrument using a Daicel CHIRALPAK IA and IB-N5 chiral columns. The exact conditions for the analyses are specified within the characterization section. HPLC traces were compared to racemic samples prepared by running the reactions using racemic ligands.

Materials. Most of the starting materials used in this study are commercial and were purchased in the highest purity available from Sigma-Aldrich, Fluka, Alfa Aesar, Fluorochem, Enamine and used as received, without further purifications. Tris(dibenzylideneacetone)dipalladium was purchased from Fluorochem and recrystalised in 200 mg portions following a reported procedure.[3] mCPBA was purchased from Sigma-Aldrich (≤ 77 %) and used as received, without further purification. The purity was assumed 77 wt% for stoichiometry calculations. mCPBA toluene solution was dried by stirring in the presence of anhydrous Na_2SO_4 for 5 min. Two different batches of mCPBA have been used without drop in yield or selectivity. The synthesis of starting materials **1** and **2** has already been described by our group. The procedures are taken from the indicated publication[4] for clarity and to facilitate the reproduction of the results.

B. Synthesis of the Starting Materials and Ligands

B.1. Synthesis of the Propargylic Amines Precursor S2

N-Benzylprop-2-yn-1-amine (S2)

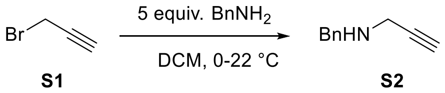

Scheme 1. Synthesis of Benzyl Propargyl amine **S2**.

To a flame-dried 250 mL two-necked round-bottom flask, benzylamine (55 mL, 0.50 mol, 5.0 equiv.) and DCM (60 mL) were added. The mixture was cooled to 0 °C. Then, *via* an addition funnel, propargyl bromide (80 wt% solution in toluene, 10.8 mL, 100 mmol, 1.0 equiv.) in DCM (40 mL) was added drop-wise over 1 hour. The reaction mixture was allowed to reach room temperature and stirred for 5 h. The reaction mixture was filtered through a plug of silica and concentrated *in vacuo* to approx. 100 mbar. The mixture was distilled under reduced pressure to give the *N*-benzylprop-2-yn-1-amine **S1** as a colorless oil (7.3 g, 50 mmol, ~90% purity according to ^{1}H NMR (T = 50 – 55 °C, 0.35 mbar).

^{1}H NMR (400 MHz, Chloroform-*d*) δ 7.41 – 7.31 (m, 4H, Ar*H*), 7.31 – 7.24 (m, 1H, ArH), 3.90 (s, 2H, PhC*H$_2$*), 3.44 (d, *J* = 2.4 Hz, 2H, C*H$_2$*C≡CH), 2.28 (t, *J* = 2.4 Hz, 1H, C≡C*H*), 1.49 (s, 1H, N*H*).

^{13}C{^{1}H} NMR (101 MHz, Chloroform-*d*) δ 139.5, 128.52, 128.49, 127.2, 82.2, 71.6, 52.4, 37.4.

Spectral data were consistent with the values reported in literature.[5]

B.2. Synthesis of the Propargylic Amines

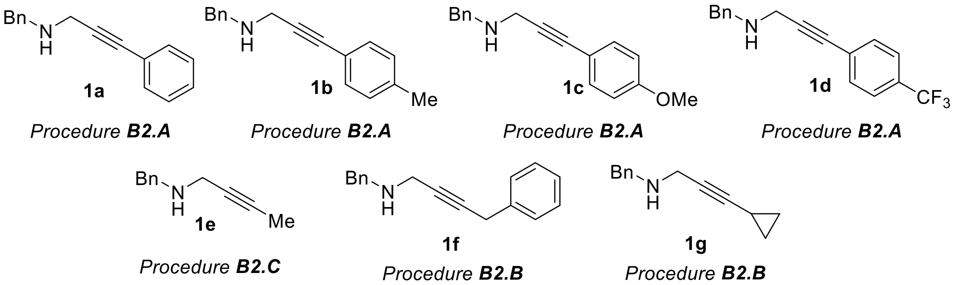

Scheme 3. Propargylic amines synthesized according to the general procedures reported.

General Procedure B2.A

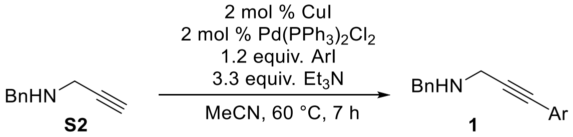

Scheme 4. General Procedure B2.A.

To a flame-dried 100 mL round bottom flask equipped with a Teflon-coated magnetic stirring bar, $Pd(PPh_3)_2Cl_2$ (42 mg, 60 µmol, 2 mol%), CuI (11 mg, 60 µmol, 2 mol%), Et_3N (0.90 g, 1.2 mL, 9.0 mmol, 3.3 equiv.) and degassed (by bubbling dry N_2 for 10 minutes) MeCN (30 mL) were added. Then, the iodoarene (1.1 equiv.) was added and the mixture was heated to 60 °C and stirred for 5 minutes. Benzyl propargyl amine **57** (0.39 g, 2.7 mmol, 1.0 equiv.) was added and the reaction mixture was stirred for 7 hours at 60 °C. Then, the reaction mixture was cooled down to ambient temperature and concentrated *in vacuo*. The resulting crude was dissolved in EtOAc (20 mL), then washed with water (20 mL) and brine (20 mL). The organic layer was dried over Na_2SO_4, filtered, and concentrated *in vacuo*. The crude was purified with Biotage flash chromatography system using Buchi FlashPure cartridge with EcoFlex silica (10% – 40% EtOAc in pentane).

General Procedure B2.B

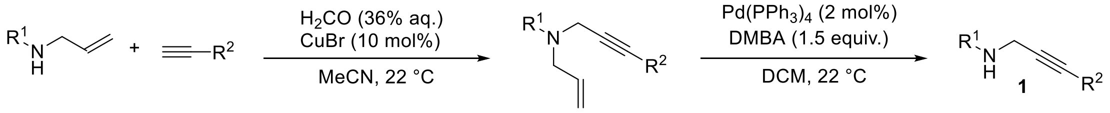

Scheme 6. General Procedure B2.C.

Following an adapted version of a reported procedure.[6] To a solution of CuBr (0.20 g, 1.4 mmol, 13 mol%) in MeCN (c = 0.15 M) was added allyl amine (1.3 equiv.), formaldehyde (3 equiv.) and alkyne (1 equiv.). The reaction mixture was stirred at room temperature for 16 hours after which it was concentrated by rotary evaporation. The residue was diluted with Et_2O (20 mL) and washed with aq. NaOH solution (5.0 M; 3 x 10 mL), dried over $MgSO_4$, filtered and concentrated by rotary evaporation. The crude material was purified by flash column chromatography (SiO_2, 0-2% EtOAc in pentane).

Deallylation: The tertiary amine obtained from the previous step (1 equiv.) was added to a solution of $Pd(PPh_3)_4$ (2 mol%) and 1,3-dimethylbarbituric acid (1.5 equiv.) in DCM (c = 0.18 M) under an N_2 atmosphere. The reaction mixture was stirred at room temperature for 16 hours. The reaction mixture was concentrated to a quarter of its original volume and diluted with ether (40 mL) and washed with sat. $NaHCO_3$ (3 x 15 mL). The organic layer was extracted with aq. HCl (1.0 M; 3 x 15 mL) after which the combined aqueous layers and any precipitated solids were basified with K_2CO_3 (pH >7) and extracted with DCM (3 x 25 mL). The combined extracts were dried over $MgSO_4$, filtered and concentrated by rotary evaporation. The crude material was purified by flash column chromatography (SiO_2, 20-50% EtOAc in pentane).

Procedure B2.C for the Synthesis of 1e

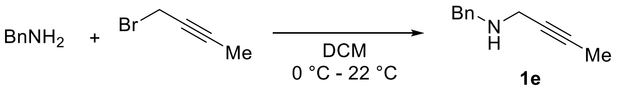

Scheme 7. General Procedure B2.D for the synthesis of **74**.

A solution of benzyl amine (4-6 equiv.) in DCM (15 mL) at 0 °C was stirred vigorously while a solution of bromo-2-butyne (2.5 mL, 27 mmol, 1 equiv.) in DCM (15 mL) was slowly added. The reaction mixture was then warmed to room temperature and stirred for 5 hours. It was then filtered through silica gel, eluting with 40% EtOAc in pentane and the resulting solution concentrated. Purification was performed by column chromatography (SiO_2, 10-40% EtOAc in pentane) to afford benzyl butynylamine **71** as a straw yellow oil (3.4 g, 21 mmol, 74% yield). Further purification could be achieved by Kugelrohr distillation (86 °C at $5x10^{-1}$ mbar).

^{1}H NMR (400 MHz, Chloroform-*d*) δ 7.39 – 7.21 (m, 5H, Ar*H*), 3.86 (s, 2H, ArC*H₂*), 3.38 (q, *J* = 2.4 Hz, 2H, CH_2C≡C), 1.85 (t, *J* = 2.4 Hz, 3H, CH_3), 1.57 (bs, 1H N*H*).

^{13}C NMR (101 MHz, Chloroform-*d*) δ 139.7, 128.4, 128.3, 127.0, 79.1, 77.1, 52.5, 37.8, 3.5.

Spectral data was consistent with the values reported in literature.[6]

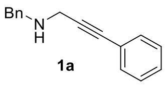

N-Benzyl-3-phenylprop-2-yn-1-amine (1a)

Prepared following an up-scaled general procedure B2.A using *N*-benzylprop-2-yn-1-amine **S2** (2.20 g, 13.5 mmol, 1.0 equiv.), iodobenzene (3.1 g, 1.7 mL, 15 mmol, 1.1 equiv.), Et_3N (4.5 g, 6.3 mL, 45 mmol, 3.3 equiv.), $Pd(PPh_3)_2Cl_2$ (211 mg, 300 µmol, 2 mol%) and CuI (57 mg, 300 µmol, 2 mol%). Purification was performed by Biotage flash column chromatography system with a 120 g cartridge (SiO_2, 10 – 40% EtOAc in pentane) to afford *N*-benzyl-3-phenylprop-2-yn-1-amine (**1a**) as an orange oil (2.5 g, 11 mmol, 75% yield).

^{1}H NMR (400 MHz, Chloroform-*d*) δ 7.52 – 7.20 (m, 9H, Ar*H*), 3.96 (s, 2H, PhCH_2), 3.66 (s, 2H, CH_2C≡C), 1.73 (br. s, 1H, N*H*).

^{13}C{^{1}H} NMR (101 MHz, Chloroform-*d*) δ 139.5, 131.7, 128.5 (2C), 128.3, 128.1, 127.2, 123.2, 87.5, 83.8, 52.5, 38.3.

Spectral data were consistent with the values reported in literature.[6]

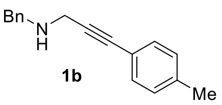

N-Benzyl-3-(*p*-tolyl)prop-2-yn-1-amine (1b)

Prepared following general procedure B2.A using *p*-tolyliodobenzene (667 mg, 3.06 mmol, 1.1 equiv.). Purification was performed by Biotage flash column chromatography system with a 25 g cartridge (SiO_2, 10 – 40% EtOAc in pentane) to afford *N*-benzyl-3-(p-tolyl)prop-2-yn-1amine (**63**) as an orange oil (512 mg, 2.13 mmol, 79% yield).

^{1}H NMR (400 MHz, Chloroform-d) δ 7.41 – 7.29 (m, 6H, Ar*H*), 7.29 – 7.22 (m, 1H, Ar*H*), 7.12 (d, *J* = 7.9 Hz, 2H, Ar*H*), 3.95 (s, 2H, PhCH_2), 3.65 (s, 2H, CH_2C-C≡C), 2.35 (s, 3H), 1.68 (br. s., 1H, N*H*)

^{13}C{^{1}H} NMR (101 MHz, $CDCl_3$) δ 139.7, 138.3, 131.7, 129.2, 128.62, 128.59, 127.3, 120.3, 86.7, 84.0, 52.6, 38.4, 21.6.

Spectral data were consistent with the values reported in literature.[6]

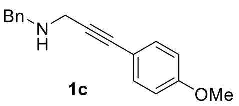

N-Benzyl-3-(4-methoxyphenyl)prop-2-yn-1-amine (1c)

Prepared following modified general procedure B2.A using $Pd(PPh_3)_2Cl_2$ (90 mg, 0.13 mmol, 5 mol%), dppf (86 mg, 0.16 mmol, 6 mol%), CuI (25 mg, 0.13 mmol, 5 mol%), DABCO (0.76 g, 6.8 mmol, 2.6 equiv.) and 4-iodo-anisole (0.79 g, 6.4 mmol, 1.3 mmol) in DMSO (10 mL; degassed by bubbling N_2). The crude material was dry-loaded onto SiO_2 and purified by column chromatography (SiO_2, 15-30% EtOAc in pentane) affording *N*-benzyl-3-(4-methoxyphenyl)prop-2-yn-1-amine (**1c**) as a light orange solid (0.28 g, 1.1 mmol, 43% yield).

^{1}H NMR (400 MHz, Chloroform-*d*) δ 7.42 – 7.23 (m, 7H, Ar*H*), 6.87 – 6.81 (m, 2H, Ar*H*), 3.95 (s, 2H, ArCH_2), 3.81 (s, 3H, CH_3), 3.64 (s, 2H, CH_2C≡C), 1.64 (bs, 1H, N*H*).

^{13}C NMR (101 MHz, Chloroform-*d*) δ 159.4, 139.6, 133.0, 128.4 (2C), 127.1, 115.3, 113.9, 86.0, 83.5, 55.3, 52.5, 38.3.

Spectral data was consistent with the values reported in literature.[7]

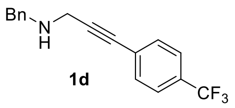

1d

N-Benzyl-3-(4-(trifluoromethyl)phenyl)prop-2-yn-1-amine (1d)

Prepared following modified general procedure B2.A using $Pd(PPh_3)_2Cl_2$ (90 mg, 0.13 mmol, 5 mol%), dppf (86 mg, 0.16 mmol, 6 mol%), CuI (25 mg, 0.13 mmol, 5 mol%), DABCO (0.76 g, 6.8 mmol, 2.6 equiv.) and 4-trifluro-Iodobenzene (0.92 g, 3.4 mmol, 1.3 equiv.) in DMSO (10 mL; degassed by bubbling N_2). The crude material was dry-loaded onto SiO_2 and purified by column chromatography (SiO_2, 10-20% EtOAc in pentane) affording *N*-benzyl-3-(4-(trifluoromethyl)phenyl)prop-2-yn-1-amine (**1d**) as a dark orange oil (0.55 g, 1.9 mmol, 72% yield)

^{1}H NMR (400 MHz, Chloroform-*d*) δ 7.61 – 7.24 (m, 9H, Ar*H*), 3.95 (s, 2H, ArC*H*$_2$), 3.67 (s, 2H, C*H*$_2$C≡C), 1.76 (bs, 1H, N*H*).

^{13}C{^{1}H} NMR (101 MHz, Chloroform-*d*) δ 139.3, 131.9, 129.8 (q, *J* = 32.7 Hz), 128.5, 128.4, 127.2, 127.0, 125.2 (q, *J* = 3.9 Hz), 123.91 (q, *J* = 272.2 Hz), 90.2, 82.5, 52.6, 38.2.

^{19}F NMR (376 MHz, Chloroform-*d*) δ -63.2.

Spectral data was consistent with the values reported in literature.[7]

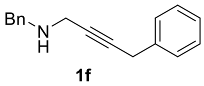

1f

N-Benzyl 4-phenyl-but-2-ynylamine (1f)

Prepared following general procedure B2.B using CuBr (0.18 g, 1.3 mmol, 12 mol%), allyl benzylamine (1.9 g, 13 mmol, 1.3 equiv), formaldehyde (2.5 mL, 33 mmol 36% aq. solution, 3.1 equiv) and phenylpropyne (1.2 g, 10 mmol, 1 equiv.) in MeCN (60 mL). Purification of the crude product by column chromatography (SiO_2, 0-2% EtOAc in pentane) to afford *N*-allyl-*N*-benzyl-4-phenyl-but-2-ynylamine as a colourless oil (2.6 g, 9.3 mmol, 89% yield).

Deallylation: the obtained tertiary amine (1.0 g, 3.6 mmol, 1 equiv.) was treated with $Pd(PPh_3)_4$ (84 mg, 73 µmol, 2 mol%) and 1,3-dimethylbarbituric acid (0.85 g, 5.5 mmol, 1.5 equiv.) in DCM (22 mL). Purification by flash column chromatography (SiO_2, 20-30% EtOAc in pentane) to afford *N*-benzyl-4-phenyl-but-2-ynylamine (**1f**) as a straw coloured oil (0.76 g, 3.0 mmol, 83% yield)

^{1}H NMR (400 MHz, Chloroform-*d*) δ 7.41 – 7.20 (m, 10H, Ar*H*), 3.90 (s, 2H, ArC*H*$_2$N), 3.65 (t, *J* = 2.3 Hz, 2H, C≡CC*H*$_2$Ph), 3.48 (t, *J* = 2.3 Hz, 2H, NC*H*$_2$C≡C), 1.65 (br. s., 1H, N*H*).

^{13}C{^{1}H} NMR (101 MHz, Chloroform-*d*) δ 139.5, 137.0, 128.5, 128.4 (2C), 127.9, 127.1, 126.6, 81.4, 80.2, 52.5, 37.9, 25.2.

Spectral data was consistent with the values reported in literature.[8]

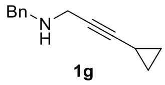

1g

N-Benzyl 4-phenyl-but-2-ynylamine (1g)

Prepared following general procedure B2.B using CuBr (0.36 g, 2.5 mmol, 12 mol%), allyl benzylamine (3.9 mL, 25 mmol, 1.3 equiv), formaldehyde (36% aq. solution; 5.0 mL, 65 mmol, 3.3 equiv.) and ethynylcyclopropane (1.7 mL, 20 mmol, 1 equiv.) in MeCN (130 mL). Purification of the crude material by column chromatography (SiO_2, 0-2% EtOAc in pentane) afforded *N*-allyl-*N*-benzyl 3-cyclopropyl-prop-2-ynylamine as a colourless oil (4.0 g, 18 mmol, 89% yield).

Deallylation: the obtained tertiary amine (1.0 g, 4.4 mmol, 1.0 equiv.) was treated with $Pd(PPh_3)_4$ (0.10 g, 89 µmol, 2 mol%) and 1,3-dimethylbarbituric acid (1.0 g, 6.7 mmol, 1.5 equiv.) in DCM (22 mL). The crude material was purified by column chromatography (SiO_3, 20-30% EtOAc in pentane) to afford *N*-benzyl 3-cyclopropyl-prop-2-ynylamine (**1g**) as a lightly straw coloured oil (0.82 g, 4.4 mmol, 99% yield).

^{1}H NMR (400 MHz, Chloroform-*d*) δ 7.45 – 7.19 (m, 5H, Ar*H*), 3.84 (s, 2H, ArC*H*$_2$), 3.37 (d, *J* = 2.0 Hz, 2H, C*H*$_2$C≡C), 1.50 (bs, 1H, N*H*), 1.25 (dddd, *J* = 10.1, 8.6, 5.0, 2.5 Hz, 1H, C*H*(CH$_2$)$_2$), 0.80 – 0.63 (m, 4H, CH(C*H*$_2$)$_2$)).

^{13}C{^{1}H} NMR (101 MHz, Chloroform-*d*) δ 139.7, 128.4 (2C), 127.0, 87.0, 73.3, 52.5, 37.9, 8.1, -0.5.

Spectral data was consistent with the values reported in literature.[6]

B.4. Synthesis of the Ligand L1

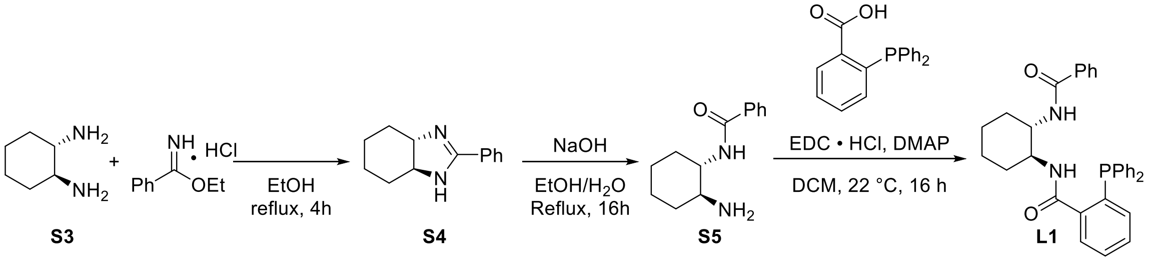

Scheme 9. Synthesis of ligand **L9**.

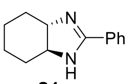

S4

(3a*S*,7a*S*)-2-phenyl-3a,4,5,6,7,7a-hexahydro-1H-benzo[d]imidazole (S4)

In accordance with a reported procedure,[9] ethyl benzimidate hydrochloride (3.3 g, 18 mmol, 1.2 equiv.) in ethanol (15 mL) was stirred at room temperature under nitrogen and (1*S*,2*S*)-cyclohexane-1,2-diamine (1.70 g, 15.0 mmol, 1.0 equiv.) was added to the solution in one portion. The solution was heated to reflux and stirred for 4 hours. 1 M NaOH (50 mL) was then added and the mixture was extracted with 5% MeOH in DCM. The organic layer was dried over sodium sulfate and concentrated to afford the crude product, which was purified by silica gel chromatography (gradient from DCM to DCM/MeOH/NH_3 100:10:1) to obtain the product as a white solid (2.50 g, 12.5 mmol, 83%). $[\alpha]D^{20}$ = -132.8 (c = 0.51, $CHCl_3$).

^{1}H NMR (400 MHz, Chloroform-*d*) δ 7.84 – 7.74 (m, 2H, Ar*H*), 7.40 (m, 3H, Ar*H*), 5.50-4.50 (bs, 1H, N*H*), 3.12 (m, 2H, NC*H*CH_2 and NHC*H*CH_2), 2.36 – 2.25 (m, 2H, NCHCH_2), 1.92 – 1.79 (m, 2H, NHCHCH_2), 1.62 – 1.49 (m, 2H, -$CH_2CH_2CH_2$-), 1.45 – 1.28 (m, 2H, -$CH_2CH_2CH_2$-).

^{13}C{^{1}H} NMR (101 MHz, Chloroform-*d*) δ 165.5, 131.0, 130.7, 128.6, 126.7, 69.8, 31.1, 25.2.

Spectral data was consistent with the values reported in literature.[15]

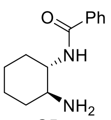

S5

N-((1*S*,2*S*)-2-aminocyclohexyl)benzamide (S5)

In accordance with a reported procedure,[9] to compound **S4** (2.30 g, 11.5 mmol) was added 19 mL 5% NaOH and 42 mL EtOH/H_2O (2:1) and the solution was heated to reflux for 16 hours. After cooling to room temperature, the ethanol was removed in vacuum, and the crude product was extracted with DCM. The product was purified by silica column chromatography (gradient from DCM to DCM/MeOH/NH_3 100:10:1) to provide the desired product as a white solid. (1.6 g, 7.3 mmol, 64%).

$[\alpha]D^{20}$ = -16.5 (c = 0.51, $CHCl_3$).

^{1}H NMR (400 MHz, Chloroform-*d*) δ 7.82 – 7.76 (m, 2H, Ar*H*), 7.54 – 7.39 (m, 3H, Ar*H*), 6.12 (d, *J* = 8.4 Hz, 1H, N*H*), 3.71 (dddd, *J* = 11.9, 9.9, 8.3, 4.1 Hz, 1H, NHC*H*CH_2), 2.49 (td, *J* = 10.2, 3.9 Hz, 1H, NH_2C*H*CH_2), 2.14 (ddd, *J* = 12.7, 4.0, 2.1 Hz, 1H, NHCHCH_aH_b), 2.08 – 1.98 (m, 1H, NHCHCH_aH_b), 1.76 (dq, *J* = 9.7, 2.7 Hz, 2H, NH_2CHCH_2), 1.54 – 1.12 (m, 6H, NH_2 and 2 x -$CH_2CH_2CH_2$-).

^{13}C{^{1}H} NMR (101 MHz, Chloroform-*d*) δ 167.9, 134.9, 131.5, 128.7, 127.0, 56.8, 55.8, 35.9, 32.7, 25.3, 25.2.

Spectral data was consistent with the values reported in literature.[15]

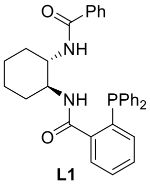

L1

N-((1*S*,2*S*)-2-benzamidocyclohexyl)-2-(diphenylphosphino)benzamide (L1)

To a stirred solution of 2-(diphenylphosphino)benzoic acid (1.5 g, 5.0 mmol, 1.1 equiv.) and DMAP (280 mg, 2.30 mmol, 0.5 equiv.) in DCM (20 mL) was added EDC HCl (966 mg, 5.00 mmol, 1.1 equiv.) at 0 °C. The mixture was stirred for few minutes and allowed to reach room temperature. Then, compound **S5** (1.0g, 4.6 mmol, 1 equiv.) was added followed by 8 mL of DCM. The resulting mixture was stirred at room temperature for 16 hours. The mixture was then quenched with 1 M HCl (50 mL) and extracted with DCM (2x50 mL). The combined organic layers were washed with brine and dried over sodium sulfate. The solvent was removed in vacuum and the crude mixture was purified by column chromatography (pentane/EtOAc up to 1:1) and recrystallized from boiling acetonitrile to obtain the desired compound **L1** as a white solid (1.4 g, 60%).

$[\alpha]D^{20}$ = +21.3 (c = 0.5, $CHCl_3$, >99% e.e.).

^{1}H NMR (400 MHz, Chloroform-*d*) δ 7.84 – 7.76 (m, 2H, Ar*H*), 7.51 – 7.12 (m, 16H, Ar*H*), 7.09 (d, *J* = 7.6 Hz, 1H, N*H*), 6.90 – 6.83 (m, 1H, Ar*H*), 6.19 (d, *J* = 8.4 Hz, 1H, N*H*), 3.96 (tdd, *J* = 11.8, 8.4, 3.9 Hz, 1H, NHC*H*CH_2), 3.83 (dtd, *J* = 10.8, 7.4, 3.9 Hz, 1H, NHC*H*CH_2), 2.21 (dd, *J* = 14.8, 7.6 Hz, 1H,

NHCHCH_aH$_b$), 1.86 (dd, J = 12.9, 3.6 Hz, 1H, NHCHCH$_a$$H_b$), 1.80 – 1.67 (m, 2H, NHCHCH_aH$_b$ and NHCHCH$_a$$H_b$), 1.29 (q, J = 12.7, 10.9 Hz, 3H, -CH$_2$CH_2CH$_2$-), 1.15 – 1.00 (m, 1H, -CH$_2$CH_2CH$_2$-).

^{13}C{^{1}H} NMR (101 MHz, Chloroform-*d*) δ 170.2, 167.7, 141.2, 141.0, 137.4, 137.3, 137.2, 136.1, 135.9, 134.5, 134.3, 134.07, 134.05, 133.87, 133.85, 131.3, 130.4, 129.0, 128.9, 128.84, 128.76, 128.7, 128.6, 128.5, 127.7, 127.6, 127.3, 55.5, 53.3, 32.5, 32,0, 25.0, 24.7.[a]

^{31}P NMR (162 MHz, Chloroform-*d*) δ -10.97.

HRMS (ESI/QTOF) m/z: [M + H]$^+$ Calculated for $C_{32}H_{32}N_2O_2P^+$ 507.2196; Found 507.2201.

IR (cm^{-1}) 3279 (m), 3064 (m), 2935 (m), 2860 (m), 1634 (s), 1545 (s), 1334 (m).

The (*R*,*R*)-**L9** ligand and the *rac*-**L9** were prepared using the same route starting from (*R*,*R*)-cyclohexane-1,2-diamine and racemic cyclohexane-1,2-diamine respectively.

[a]The peaks are listed not accounting for C-P coupling

B.5. General Procedure for the Enantioselective Carboetherification of Propargylic Amines

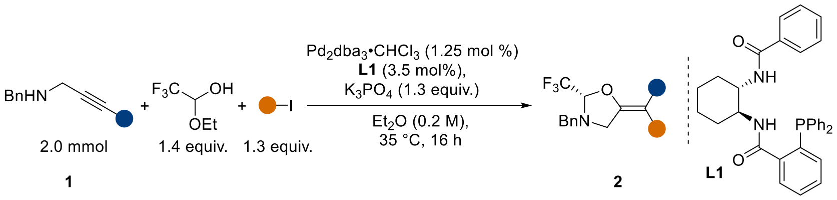

Scheme 13. Enantioselevtive Carboetherification of Propargylic Amines

An oven-dried 20 mL microwave vial equipped with a Teflon coated stirring bar was charged with $Pd_2(dba)_3$ • $CHCl_3$ (26 mg, 25 µmol, 1.25 mol%), the ligand (36 mg, 70 µmol, 3.5 mol%) and K_3PO_4 (552 mg, 2.60 mmol, 1.3 equiv.). The vial was then sealed, purged with N_2 and placed in a heating metal block. 12.5 mL of Et_2O were added and the suspension was stirred at 35 °C for 10 minutes. Propargylic amine (2.00 mmol, 1.0 equiv) and 1-ethoxy-2,2,2-trifluoroethanol (85% in EtOH, 0.38 mL, 2.4 mmol 1.4 equiv.) were added followed by the aryl iodide (2.40 mmol, 1.3 equiv.). The resulting suspension was stirred at 35 °C for 16 hours. Next, the reaction mixture was filtered through a plug of silica gel eluting with 15 mL of 20 % (v/v) EtOAc in pentane and concentrated in vacuo. The crude material was purified by flash column chromatography on silica gel to afford the corresponding product. Spectral data was consistent with the values reported in literature.[4]

B.6. Characterization of Products of the Enantioselevtive Carboetherification

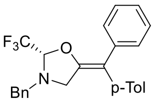

2a

(*S*,*E*)-3-Benzyl-5-(phenyl(p-tolyl)methylene)-2-(trifluoromethyl)oxazolidine (2a)

An oven dried 50 mL round-bottom flask equipped with a Teflon stir bar was charged with $Pd_2(dba)_3$ • $CHCl_3$ (65 mg, 63 µmol, 1.25 mol%), the ligand (90 mg, 0.18 mmol, 3.5 mol%) and K_3PO_4 (1.38 g, 6.50 mmol, 1.3 equiv.). The flask was then purged with N_2 and placed in a heating metal block. 20 mL of Et_2O were added and the suspension was stirred at 35 °C for 10 minutes N-benzyl-3-phenylprop-2-yn-1-amine (1.11 g, 5.00 mmol, 1.0 equiv) and 1-ethoxy-2,2,2-trifluoroethanol (85% in EtOH, 0.96 mL, 7.0 mmol, 1.4 equiv.) were added followed by 1-iodo-4-methylbenzene (1.42 g, 6.50 mmol, 1.3 equiv.) and the remaining 5 mL of Et_2O to rinse the wall. The resulting suspension was stirred at 35 °C for 16 hours. Then, the reaction mixture was filtered through a plug of deactivated silica gel eluting with 50 mL of pentane/EtOAc 9:1 and concentrated in vacuo and analyzed by ^{1}H NMR with an internal standard (trichloroethylene, 0.1 equiv., NMR yield: >99%). The crude material was purified by flash column chromatography (pentane/EtOAc gradient 100:0 to 100:3) to give the corresponding product **2a** (2.04 g, 4.98 mmol, >99% yield) as a white solid. The enantiomeric ratio was determined to be 97.0:3.0 by HPLC analysis on a Daicel Chiralpak IB N-5 column: 99:1 hexane/IPA, flow rate 1 mL/min, λ = 254 nm: τ_{Minor} = 6.9 min τ_{Major} = 8.6 min.

^{1}H NMR (400 MHz, Chloroform-*d*) δ 7.39 – 7.26 (m, 9H, Ar*H*), 7.22 – 7.16 (m, 1H, Ar*H*), 7.14 (d, *J* = 7.8 Hz, 2H, Ar*H*), 7.05 (d, *J* = 8.1 Hz, 2H, Ar*H*), 5.13 (q, *J* = 5.3 Hz, 1H, C*H*CF_3), 3.99 (d, *J* = 13.3 Hz, 1H, PhCH_aH_b), 3.94 (d, *J* = 16.0 Hz, 1H, NCH_aH_bC=C), 3.89 (d, *J* = 13.2 Hz, 1H, PhCH_aH_b), 3.54 (d, *J* = 16.0 Hz, 1H, NCH_aH_bC=C),). 2.35 (s, 3H, CH_3).

^{13}C{^{1}H} NMR (101 MHz, Chloroform-*d*) δ 148.4, 138.8, 137.2, 137.1, 136.7, 130.0, 129.4, 129.1, 128.8 (2C), 128.04, 128.02, 126.3, 122.9 (q, $J_{C\text{-}F}$ = 283.9 Hz), 112.9, 94.00 (q, $J_{C\text{-}F}$ = 34.4 Hz), 60.5, 54.9, 21.3.

^{19}F NMR (376 MHz, Chloroform-*d*) δ -80.3.

Recrystallization: A 50 mL round bottom-flask equipped with a Teflon stir bar and a reflux condenser was charged with oxazolidine **2a** (1.000 g, 2.44 mmol, 1.0 equiv., 97.0:3.0 *er*) and MeOH (20 mL). The mixture was heated to maintain gentle reflux. Then, MeOH was added in portions (2.5 mL) until the material was fully dissolved (around 10 mL). The heating mantle was removed and the mixture was cooled to 22 °C while maintaining the stirring. The wite precitipate was filtered and washed woth cold methanol (2 × 5 mL) to give the corresponding product **2a** (750 mg, 75 % yield). The enantiomeric ratio was determined to be 99.0:1.0 by HPLC analysis on a Daicel Chiralpak IB N-5 column: 99:1 hexane/IPA, flow rate 1 mL/min, λ = 254 nm: τ_{Minor} = 6.9 min τ_{Major} = 8.6 min.

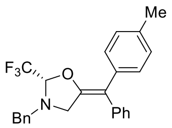

2b

(*R*,*Z*)-3-Benzyl-5-(phenyl(p-tolyl)methylene)-2-(trifluoromethyl)oxazolidine (2b)

Prepared according to the general procedure B5 using N-benzyl-3-(p-tolyl)prop-2-yn-1-amine (471 mg, 2.00 mmol, 1.0 equiv.), iodobenzene (530 mg, 290 µl, 2.60 mmol, 1.3 equiv.) and the *(R,R)*-**L1** ligand. The crude material was purified by flash column chromatography (pentane/EtOAc gradient 100:0 to 97:3) to give the corresponding olefin **2b.** (611 mg, 1.49 mmol, 75 % yield) as colorless oil. The enantiomeric ratio was determined to be 94.5:5.5 by HPLC analysis on a Daicel Chiralpak IB N-5 column: 99:1 hexane/IPA, flow rate 1 mL/min, λ = 254 nm: τ_{Major} = 7.3 min, τ_{Minor} = 8.7 min.

^{1}H NMR (400 MHz, Chloroform-d) δ 7.36 – 7.28 (m, 7H, Ar*H*), 7.27 – 7.21 (m, 3H, Ar*H*), 7.18 – 7.13 (m, 2H, Ar*H*), 7.10 (d, *J* = 8.0 Hz, 2H, Ar*H*), 5.11 (q, *J* = 5.3 Hz, 1H, C*H*CF_3), 4.03 – 3.83 (m, 3H, PhCH_2 and NC$H_a$$H_b$C=C), 3.52 (d, *J* = 15.7 Hz, 1H, NC$H_a$$H_b$C=C), 2.33 (s, 3H, ArC$H_3$).

^{13}C{^{1}H} NMR (101 MHz, Chloroform-d) δ 148.1, 140.3, 137.1, 136.1, 135.8, 130.2, 129.0, 128.81, 128.77 (2C), 128.7, 128.0, 127.0, 122.9 (q, *J* = 284.0 Hz), 113.0, 93.9 (q, *J* = 34.3 Hz), 60.5, 54.8, 21.3.

^{19}F NMR (376 MHz, Chloroform-*d*) δ -80.4 (d, 3F, *J* = 5.3 Hz).

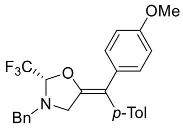

2c

(*S*,*Z*)-3-Benzyl-5-((4-methoxyphenyl)(*p*-tolyl)methylene)-2-(trifluoromethyl)oxazolidine (2c)

Prepared according to the general procedure B5 using N-benzyl-3-(4-(methoxy)phenyl)prop-2-yn-1-amine (503 mg, 2.0 mmol, 1.0 equiv.) and *p*-iodotoluene (567 mg, 2.60 mmol, 1.3 equiv.). The crude material was purified by flash column chromatography (pentane/EtOAc gradient 100:0 to 97:3) to give the corresponding olefin **2c** (634 mg, 1.44 mmol, 72 % yield) as amorphous white solid. The enantiomeric ratio was determined to be 94.0:6.0 by HPLC analysis on a Daicel Chiralpak IB N-5 column: 99:1 hexane/IPA, flow rate 1 mL/min, λ = 254 nm: τ_{Minor} = 12.3 min, τ_{Major} = 28.0 min.

^{1}H NMR (400 MHz, Chloroform-d) δ 7.36 – 7.27 (m, 7H, Ar*H*), 7.16 – 7.09 (m, 2H, Ar*H*), 7.07 – 7.00 (m, 2H, Ar*H*), 6.87 – 6.78 (m, 2H, Ar*H*), 5.10 (q, *J* = 5.3 Hz, 1H, C*H*CF_3), 4.03 – 3.85 (m, 3H, PhCH_2 and NC$H_a$$H_b$C=C), 3.80 (s, 3H, OC$H_3$), 3.52 (dd, *J* = 15.7, 0.9 Hz, 1H, NC$H_a$$H_b$C=C), 2.34 (s, 3H, ArC$H_3$).

^{13}C{^{1}H} NMR (101 MHz, Chloroform-d) δ 158.0, 147.3, 147.3, 137.4, 137.1, 136.7, 131.4, 130.2, 130.0, 129.4, 128.8, 128.0, 122.9 (q, *J* = 283.9 Hz), 113.5, 112.5, 93.8 (q, *J* = 34.2 Hz), 60.5, 55.4, 54.8, 21.3.

^{19}F NMR (376 MHz, Chloroform-*d*) δ -80.3 (d, 3F, *J* = 5.3 Hz).

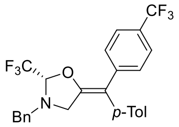

2d

(*S*,*Z*)-3-Benzyl-5-(*p*-tolyl(4-(trifluoromethyl)phenyl)methylene)-2-(trifluoromethyl)oxazolidine (2d)

Prepared according to the general procedure B5 using N-benzyl-3-(4-(trifluoromethyl)phenyl)prop-2-yn-1-amine (116 mg, 0.400 mmol, 1.0 equiv.) and *p*-iodotoluene (113 mg, 0.520 mmol, 1.3 equiv.). The crude material was purified by flash column chromatography (pentane/EtOAc gradient 100:0 to 97:3) to give the corresponding olefin **2d** (672 mg, 1.41 mmol, 70 % yield) as colorless oil. The enantiomeric ratio was determined to be 94.0:6.0 by HPLC analysis on a Daicel Chiralpak IB N-5 column: 99:1 hexane/IPA, flow rate 1 mL/min, λ = 254 nm: τ_{Minor} = 7.3 min, τ_{Major} = 8.7 min.

^{1}H NMR (400 MHz, Chloroform-d) δ 7.51 (d, *J* = 8.5 Hz, 2H, Ar*H*), 7.46 (d, *J* = 8.5 Hz, 2H, Ar*H*), 7.36 – 7.27 (m, 5H, Ar*H*), 7.15 (d, *J* = 8.0 Hz, 2H, Ar*H*), 7.02 (d, *J* = 8.0 Hz, 2H, Ar*H*), 5.17 (q, *J* = 5.2 Hz, 1H, C*H*CF_3), 3.99 (d, *J* = 13.3 Hz, 1H, PhC$H_a$$H_b$), 3.95 (d, *J* = 16.2 Hz, 1H, NC$H_a$$H_b$C=C), 3.90 (d, *J* = 13.3 Hz, 1H, PhC$H_a$$H_b$), 3.54 (d, *J* = 16.2 Hz, 1H, NC$H_a$$H_b$C=C), 2.36 (s, 3H, ArC$H_3$).

^{13}C{^{1}H} NMR (101 MHz, Chloroform-d) δ 150.2, 142.4, 137.3, 138.8, 136.3, 130.0, 129.7, 129.1, 128.83, 128.78, 128.2, 127.9 (q, *J* = 32.0 Hz), 124.9 (q, *J* = 3.7 Hz), 124.5 (q, *J* = 272 Hz), 122.7 (q, *J* = 284.0 Hz) 111.7, 94.4 (q *J* = 34.4 Hz), 60.6, 55.1, 21.3.

^{19}F NMR (376 MHz, Chloroform-*d*) δ -62.4 (s, 3F, ArCF_3), -80.4 (d, 3F, *J* = 5.2 Hz, CHCF_3).

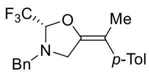

2e

(*S*,*E*)-3-Benzyl-5-(1-(*p*-tolyl)ethylidene)-2-(trifluoromethyl)oxazolidine (2e)

Prepared according to the general procedure B5 using *N*-benzylbut-2-yn-1-amine (319 mg, 2.00 mmol, 1.0 equiv.) and *p*-iodotoluene (567 mg, 2.40 mmol, 1.3 equiv.). The crude material was purified by flash column chromatography (pentane/EtOAc gradient 100:0 to 97:3) to give the corresponding olefin **2e** (525 mg, 1.51 mmol, 76 % yield) as amorphous white solid. The

enantiomeric ratio was determined to be 85.5:14.5 by HPLC analysis on a Daicel Chiralpak IB N-5 column: 99:1 hexane/IPA, flow rate 1 mL/min, λ = 254 nm: τ_{Minor} = 5.5 min, τ_{Major} = 6.4 min.

^{1}H NMR (400 MHz, Chloroform-d) δ 7.35 – 7.26 (m, 5H, Ar*H*), 7.10 (d, *J* = 8.0 Hz, 2H, *m*-Me-Ar*H*), 7.07 – 7.02 (m, 2H, *o*-Me-Ar*H*), 4.96 (q, *J* = 5.3 Hz, 1H, $CHCF_3$), 3.97 (d, *J* = 14.9 Hz, 1H, NCH_aH_bC=C), 3.92 (d, *J* = 13.3 Hz, 1H, $PhCH_aH_b$), 3.81 (d, *J* = 13.3 Hz, 1H, $PhCH_aH_b$), 3.45 (dt, *J* = 14.9, 1.3 Hz, 1H, NCH_aH_bC=C), 2.32 (s, 3H, $ArCH_3$), 2.07 (t, *J* = 1.7 Hz, 3H, C—CCH_3).

^{13}C{^{1}H} NMR (101 MHz, Chloroform-d) δ 146.9, 138.3, 137.3, 136.0, 129.1, 128.7 (2C), 127.9, 127.4, 123.0 (q, *J* = 283.9 Hz), 107.6, 94.6 (q, *J* = 34.0 Hz), 60.3, 53.3, 21.2, 16.6.

^{19}F NMR (376 MHz, Chloroform-*d*)) δ -80.6 (d, 3F, *J* = 5.3 Hz).

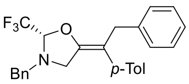
2f

(*S*,*E*)-3-Benzyl-5-(2-phenyl-1-(*p*-tolyl)ethylidene)-2-(trifluoromethyl)oxazolidine (2f)

Prepared according to the general procedure B5 using *N*-benzyl-4-phenylbut-2-yn-1-amine (471 mg, 2.00 mmol, 1.0 equiv.) and *p*-iodotoluene (567 mg, 2.40 mmol, 1.3 equiv.). The crude material was purified by flash column chromatography (pentane/EtOAc gradient 100:0 to 97:3) to give the corresponding olefin **2f** (680 mg, 1.61 mmol, 80 % yield) as amorphous white solid. The enantiomeric ratio was determined to be 92.0:8.0 by HPLC analysis on a Daicel Chiralpak IB N-5 column: 99:1 hexane/IPA, flow rate 1 mL/min, λ = 254 nm: τ_{Minor} = 6.3 min, τ_{Major} = 7.1 min.

^{1}H NMR (400 MHz, Chloroform-d) δ 7.39 – 7.28 (m, 5H, Ar*H*), 7.25 – 7.18 (m, 2H, Ar*H*), 7.18 – 7.10 (m, 3H, Ar*H*), 7.03 (d, *J* = 8.0 Hz, 2H, *m*-Me-Ar*H*), 6.95 (d, *J* = 8.0 Hz, 2H, *o*-Me-Ar*H*), 5.02 (q, *J* = 5.3 Hz, 1H, $CHCF_3$), 4.03 – 3.94 (m, 2H, NCH_aH_bC=C and $PhCHaH_bN$), 3.94 – 3.73 (m, 3H, $PhCHaH_bN$ and C=CCH_2Ph), 3.47 (d, *J* = 15.2 Hz, 1H, $NCHaH_bC$=C), 2.28 (s, 3H, $ArCH_3$).

^{13}C{^{1}H} NMR (101 MHz, Chloroform-d) δ 148.0, 140.5, 137.2, 136.8, 136.2, 129.1, 128.74, 128.71, 128.6, 128.4, 128.3, 128.2, 127.9, 123.0 (q, *J* = 283.9 Hz), 111.4, 92.8 (q, *J* = 34.1 Hz), 60.4, 53.3, 37.2, 21.2.

^{19}F NMR (376 MHz, Chloroform-*d*) δ -80.5 (d, 3F, *J* = 5.3 Hz).

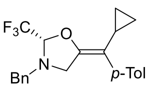
2g

(*S*,*E*)-3-Benzyl-5-(cyclopropyl(*p*-tolyl)methylene)-2-(trifluoromethyl)oxazolidine (2g)

Prepared according to the general procedure B5 using *N*-benzyl-3-cyclopropylprop-2-yn-1-amine (371 mg, 2.00 mmol, 1.0 equiv.) and *p*-iodotoluene (567 mg, 2.60 mmol, 1.3 equiv.). The crude material was purified by flash column chromatography (pentane/EtOAc gradient 100:0 to 97:3) to give the corresponding olefin **2g** (407 mg, 1.09 mmol, 54 % yield) as colorlss oil. The enantiomeric ratio was determined to be 88.0:12.0 by HPLC analysis on a Daicel Chiralpak IB N-5 column: 99:1 hexane/IPA, flow rate 1 mL/min, λ = 254 nm: τ_{Minor} = 4.8 min, τ_{Major} = 5.5 min.

^{1}H NMR (400 MHz, Chloroform-d) δ 7.33 – 7.24 (m, 5H, Ar*H*), 7.07 (d, *J* = 7.9 Hz, 2H, Ar*H*), 6.95 (d, *J* = 7.9 Hz, 2H, Me-Ar*H*), 5.02 (q, *J* = 5.3 Hz, 1H, $CHCF_3$), 3.95 (d, *J* = 13.3 Hz, 1H, $PhCH_aH_b$), 3.81 (d, *J* = 13.3 Hz, 1H, $PhCH_aH_b$), 3.71 (d, *J* = 15.2 Hz, 1H, NCH_aH_bC=C), 3.21 (d, *J* = 15.2 Hz, 1H, NCH_aH_bC=C), 2.31 (s, 3H, $ArCH_3$), 2.01 – 1.88 (m, 1H, $CH(CH_2)CH_2$), 0.68 – 0.58 (m, 2H, $CH(CH_2)CH_2$), 0.37 – 0.18 (m, 2H, $CH(CH_2)CH_2$).

^{13}C{^{1}H} NMR (101 MHz, Chloroform-d) δ 147.6, 137.4, 136.5, 134.5, 129.7, 129.0, 128.69, 128.67, 127.8, 123.0 (q, *J* = 284.0 Hz), 112.7, 93.2 (q, *J* = 34.0 Hz), 60.4, 53.5, 21.3, 11.6, 4.8, 4.4.

^{19}F NMR (376 MHz, Chloroform-*d*) δ -80.5 (d, 3F, *J* = 5.3 Hz).

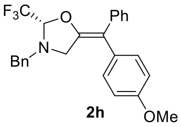
2h

(*S*,*E*)-3-Benzyl-5-((4-methoxyphenyl)(phenyl)methylene)-2-(trifluoromethyl)oxazolidine (2h)

Prepared according to the general procedure B5 using N-benzyl-3-phenylprop-2-yn-1-amine (430 µL, 2.00 mmol, 1.0 equiv.) and 4-iodoanisole (562 mg, 2.60 mmol, 1.3 equiv). 2.5 mol% of $Pd_2(dba)_3$ • $CHCl_3$ (52 mg, 0.050 mmol) and 7 mol% of ligand (71 mg, 0.14 mmol) were used. The crude material was purified by flash column chromatography (pentane/EtOAc gradient 100:0 to 100:3) to give the corresponding olefin **2h** (685 mg, 1.61, 81 % yield) as a white solid. The enantiomeric ratio was determined to be 95.0:5.0 by HPLC analysis on a Daicel Chiralpak IB N-5 column: 99:1 hexane/IPA, flow rate 1 mL/min, λ = 254 nm: τ_{Minor} = 10.5 min, τ_{Major} = 16.5 min.

^{1}H NMR (400 MHz, Chloroform-*d*) δ 7.40 – 7.25 (m, 9H, Ar*H*), 7.22 – 7.15 (m, 1H, Ar*H*), 7.08 (d, *J* = 8.7 Hz, 2H, Ar*H*), 6.86 (d, *J* = 8.7 Hz, 2H, Ar*H*), 5.13 (q, *J* = 5.3 Hz, 1H, $CHCF_3$), 4.00 (d, *J* = 13.3 Hz, 1H,

PhCH_aH$_b$), 3.96 – 3.85 (m, 2H, PhCH$_a$$H_b$ and NCH_aH$_b$C=C), 3.81 (s, 3H, OCH_3), 3.52 (dd, J = 15.6, 1.5 Hz, 1H, NCH$_a$$H_b$C=C).

^{13}C{^{1}H} NMR (101 MHz, Chloroform-d) δ 158.6, 148.4, 138.9, 137.1, 132.4, 131.3, 129.0, 128.77, 128.76, 128.0 (2C), 126.3, 122.9 (q, J = 284.0 Hz), 114.1, 112.5, 94.0 (q, J = 34.4 Hz), 60.5, 55.4, 54.9.

^{19}F NMR (376 MHz, Chloroform-d) δ -80.3 (d, J = 4.9 Hz).

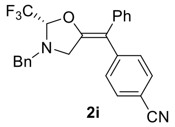

2i

(S,E)-4-((3-Benzyl-2-(trifluoromethyl)oxazolidin-5-ylidene)(phenyl)methyl)benzonitrile (2i)

Prepared according to the general procedure B5 using N-benzyl-3-phenylprop-2-yn-1-amine (430 µL, 2.00 mmol, 1.0 equiv.) and 4-iodobenzonitrile (595 mg, 2.60 mmol, 1.3 equiv). The crude material was purified by flash column chromatography (pentane/EtOAc gradient 100:0 to 100:10) to give the corresponding olefin **2i** (560 mg, 1.33 mmol, 67 % yield) as a white foam. The enantiomeric ratio was determined to be 85.0:15.0 by HPLC analysis on a Daicel Chiralpak IA column: 95:5 hexane/IPA, flow rate 1 mL/min, λ = 254 nm: τ_{Major} = 6.6 min, τ_{Minor} = 7.2 min.

^{1}H NMR (400 MHz, Chloroform-d) δ 7.60 (d, J = 8.4 Hz, 2H, ArH), 7.38 – 7.21 (m, 12H, ArH), 5.16 (q, J = 5.1 Hz, 1H, CHCF$_3$), 4.02 (d, J = 13.3 Hz, 1H, PhCH_aH$_b$), 3.97 (d, J = 15.9 Hz, 1H, NCH_aH$_b$C=C), 3.90 (d, J = 13.3 Hz, 1H, PhCH$_a$$H_b$), 3.55 (d, J = 15.9, 1H, NCH$_a$$H_b$C=C).

^{13}C{^{1}H} NMR (101 MHz, Chloroform-d) δ 149.9, 145.3, 137.6, 136.6, 132.5, 130.6, 129.3, 128.9, 128.7, 128.4, 128.3, 127.1, 122.7 (q, J = 283.9 Hz), 118.9, 112.3, 110.7, 94.1 (q, J = 34.6 Hz) 60.4, 54.7.

^{19}F{^{1}H} NMR (376 MHz, Chloroform-d) δ -80.3.

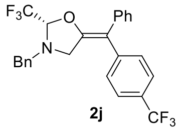

2j

(S,E)-3-Benzyl-5-(phenyl(4-(trifluoromethyl)phenyl)methylene)-2-(trifluoromethyl)oxazolidine (2j)

Prepared according to the general procedure B5 using N-benzyl-3-phenylprop-2-yn-1-amine (430 µL, 2.00 mmol, 1.0 equiv.) and 4-iodobenzotrifluoride (352 µL, 653 mg, 2.6 mmol, 1.3 equiv). The crude material was purified by flash column chromatography (pentane/EtOAc gradient 100:0 to 100:3) to give the corresponding olefin **2j** (723 mg, 1.56 mmol, 91 % yield) as a colorless oil. The enantiomeric ratio was determined to be 91.0:9.0 by HPLC analysis on a Daicel Chiralpak IB N-5 column: 99:1 hexane/IPA, flow rate 1 mL/min, λ = 254 nm: τ_{Minor} = 13.8 min, τ_{Major} = 16.7 min.

^{1}H NMR (400 MHz, Chloroform-d) δ 7.58 (d, J = 8.1 Hz, 2H, ArH), 7.40 – 7.17 (m, 12H, ArH), 5.16 (q, J = 5.2 Hz, 1H, CHCF$_3$), 4.01 (d, J = 13.3 Hz, 1H, PhCH_aH$_b$), 3.99 – 3.93 (m, 1H, NCH_aH$_b$C=C), 3.91 (d, J = 13.3 Hz, 1H, PhCH$_a$$H_b$), 3.54 (dd, J = 15.8, 1.4 Hz, 1H, NCH$_a$$H_b$C=C).

^{13}C{^{1}H} NMR (101 MHz, Chloroform-d) δ 149.4, 144.0, 138.0, 136.7, 130.4, 129.3 (q, J = 32.2 Hz), 129.2, 128.9, 128.8, 128.3, 128.2, 126.8, 125.7 (q, J = 3.8 Hz), 124.3 (q, J = 275.6 Hz) 122.73 (q, J = 283.9 Hz), 112.2, 94.1 (q, J = 34.5 Hz), 60.5, 54.8.

^{19}F{^{1}H} NMR (377 MHz, Chloroform-d) δ -62.5, -80.3.

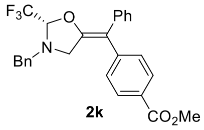

2k

(S,E)-Methyl-4-((3-benzyl-2-(trifluoromethyl)oxazolidin-5-ylidene)(phenyl)methyl)benzoate (2k)

Prepared according to the general procedure B5 using N-benzyl-3-phenylprop-2-yn-1-amine (430 µL, 2.00 mmol, 1.0 equiv.) and methyl 4-iodobenzoate (681 mg, 2.60 mmol, 1.3 equiv). The crude material was purified by flash column chromatography (pentane/EtOAc gradient 100:0 to 100:10) to give the corresponding olefin **2k** (715 mg, 1.58 mmol, 79 % yield) as a white foam. The enantiomeric ratio was determined to be 90.5:9.5 by HPLC analysis on a Daicel IA column: 95:5 hexane/IPA, flow rate 1 mL/min, λ = 254 nm: τ_{Major} = 6.2 min, τ_{Minor} = 7.1 min.

^{1}H NMR (400 MHz, Chloroform-d) δ 7.98 (d, J = 8.4 Hz, 2H, ArH), 7.37 – 7.27 (m, 9H, ArH), 7.21 (d, J = 8.4 Hz, 3H, ArH), 5.15 (q, J = 5.2 Hz, 1H, CHCF$_3$), 4.00 (d, J = 13.3 Hz, 1H, PhCH_aH$_b$), 3.95 (d, J = 15.8, 1H, NCH_aH$_b$C=C), 3.91 (m, 4H, OCH_3 and PhCH$_a$H$_b$), 3.55 (dd, J = 15.8, 1.3 Hz, 1H, NCH$_a$$H_b$C=C).

^{13}C{^{1}H} NMR (101 MHz, Chloroform-d) δ 167.0, 149.3, 145.2, 138.0, 136.8, 130.1, 130.0, 129.2, 128.85, 128.79, 128.76, 128.22, 128.17, 126.8, 122.70 (q, J = 284.1 Hz), 112.7, 94.1 (q, J = 34.5 Hz), 60.5, 54.8, 52.3.

^{19}F{^{1}H} NMR (376 MHz, Chloroform-d) δ -80.3.

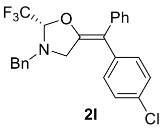

2l

(*S*,*E*)-3-Benzyl-5-((4-chlorophenyl)(phenyl)methylene)-2-(trifluoromethyl)oxazolidine (2l)

Prepared according to the general procedure B5 using N-benzyl-3-phenylprop-2-yn-1-amine (430 μL, 2.00 mmol, 1.0 equiv.) and 1-chloro-4-iodobenzene (653 mg, 2.60 mmol, 1.3 equiv.). The crude material was purified by flash column chromatography (pentane/EtOAc gradient 100:0 to 100:3) to give the corresponding olefin **2l** (613 mg, 1.43 mmol, 71 % yield) as a pale yellow oil. The enantiomeric ratio was determined to be 90.0:10.0 by HPLC analysis on a Daicel Chiralpak IB N-5 column: 99:1 hexane/IPA, flow rate 1 mL/min, λ = 254 nm: τ_{Minor} = 10.9 min, τ_{Major} = 13.2 min.

^{1}H NMR (400 MHz, Chloroform-*d*) δ 7.39 – 7.26 (m, 11H, Ar*H*), 7.23 – 7.17 (m, 1H, Ar*H*), 7.09 (d, *J* = 8.4 Hz, 2H, Ar*H*), 5.14 (q, *J* = 5.3 Hz, 1H, C*H*CF_3), 4.00 (d, *J* = 13.3 Hz, 1H, PhCH_aH_b), 3.96 – 3.85 (m, 2H, PhCH_aH_b and NCH_aH_bC=C), 3.51 (dd, *J* = 15.8, 1.5 Hz, 1H, NCH_aH_bC=C).

^{13}C{^{1}H} NMR (101 MHz, Chloroform-*d*) δ 148.9, 138.6, 138.2, 136.8, 133.0, 131.5, 129.03, 128.97, 128.84, 128.75, 128.18, 128.15, 126.6, 122.8 (q, *J* = 283.8 Hz), 112.0, 94.1 (q, *J* = 34.5 Hz), 60.5, 54.8.

^{19}F{^{1}H} NMR (376 MHz, Chloroform-*d*) δ -80.3.

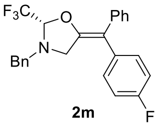

2m

(*S*,*E*)-3-Benzyl-5-((4-fluorophenyl)(phenyl)methylene)-2-(trifluoromethyl)oxazolidine (2m)

Prepared according to the general procedure B5 using N-benzyl-3-phenylprop-2-yn-1-amine (430 μL, 2.00 mmol, 1.0 equiv.) and 4-fluoroiodobenzene (300 μL, 2.60 mmol, 1.3 equiv). The crude material was purified by flash column chromatography (pentane/EtOAc gradient 100:0 to 100:3) to give the corresponding olefin **2m** (611 mg, 1.48 mmol, 74 % yield) as a colorless oil. The enantiomeric ratio was determined to be 90.0:10.0 by HPLC analysis on a Daicel Chiralpak IB N-5 column: 99:1 hexane/IPA, flow rate 1 mL/min, λ = 254 nm: τ_{Minor} = 9.6 min, τ_{Major} = 11.0 min.

^{1}H NMR (400 MHz, Chloroform-*d*) δ 7.41 – 7.25 (m, 9H, Ar*H*), 7.24 – 7.17 (m, 1H, Ar*H*), 7.16 – 7.10 (m, 2H, Ar*H*), 7.02 (td, *J* = 8.3, 1.5 Hz, 2H, Ar*H*), 5.18 – 5.12 (m, 1H, C*H*CF_3), 4.00 (d, *J* = 13.4 Hz, 1H, PhCH_aH_b), 3.95 – 3.85 (m, 2H, PhCH_aH_b and NCH_aH_bC=C), 3.49 (d, *J* = 15.4 Hz, 1H, NCH_aH_bC=C).

^{13}C{^{1}H} NMR (101 MHz, Chloroform-*d*) δ 162.0 (d, *J* = 246.3 Hz), 148.8, 138.4, 136.9, 136.0 (d, *J* = 3.4 Hz), 131.8 (d, *J* = 8.0 Hz), 128.9, 128.82, 128.76, 128.14, 128.12, 126.5, 122.8 (q, *J* = 283.9 Hz), 115.7 (d, *J* = 21.3 Hz), 112.0, 94.1 (q, *J* = 34.4 Hz), 60.5, 54.9.

^{19}F NMR (376 MHz, Chloroform-*d*) δ -80.3 (d, *J* = 4.2 Hz), -115.2.

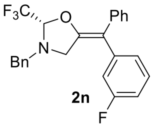

2n

(*S*,*E*)-3-Benzyl-5-((3-fluorophenyl)(phenyl)methylene)-2-(trifluoromethyl)oxazolidine (2n)

Prepared according to the general procedure B5 using N-benzyl-3-phenylprop-2-yn-1-amine (430 μL, 2.00 mmol, 1.0 equiv.) and 1-fluoro-3-iodobenzene (305 μL, 2.60 mmol, 1.3 equiv). The crude material was purified by flash column chromatography (pentane/EtOAc gradient 100:0 to 100:3) to give the corresponding olefin **2n** (630 mg, 1.52 mmol, 76 % yield) as a colorless oil. The enantiomeric excess was determined to be 89.5:10.5 by HPLC analysis on a Daicel Chiralpak IB N-5 column: 99:1 hexane/IPA, flow rate 1 mL/min, λ = 254 nm: τ_{Minor} = 9.0 min, τ_{Major} = 11.8 min.

^{1}H NMR (400 MHz, Chloroform-*d*) δ 7.41 – 7.26 (m, 10H, Ar*H*), 7.24 – 7.17 (m, 1H, Ar*H*), 7.02 – 6.91 (m, 2H, Ar*H*), 6.86 (ddd, *J* = 9.8, 2.5, 1.6 Hz, 1H, Ar*H*), 5.15 (q, *J* = 5.2 Hz, 1H, C*H*CF_3), 4.00 (d, *J* = 13.3 Hz, 1H, PhCH_aH_b), 3.95 (d, *J* = 16.0 Hz, 1H, NCH_aH_bC=C), 3.90 (d, *J* = 13.3 Hz, 1H, PhCH_aH_b), 3.55 (dt, *J* = 16.0, 1.4 Hz, 1H, NCH_aH_bC=C).

^{13}C{^{1}H} NMR (101 MHz, Chloroform-*d*) δ 163.0 (d, *J* = 246.6 Hz), 149.1, 142.4 (d, *J* = 7.8 Hz), 138.1, 136.8, 130.2 (d, *J* = 8.5 Hz), 129.0, 128.83, 128.78, 128.19, 128.14, 126.7, 125.87 (d, *J* = 2.9 Hz), 122.8 (q, *J* = 283.8 Hz), 117.1 (d, *J* = 21.0 Hz), 114.1 (d, *J* = 21.0 Hz), 112.2 (d, *J* = 2.0 Hz), 94.1 (q, *J* = 34.4 Hz), 60.5, 54.8.

^{19}F{^{1}H} NMR (376 MHz, Chloroform-*d*) δ -80.3, -112.9.

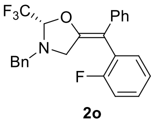

2o

(*S*,*E*)-3-Benzyl-5-((2-fluorophenyl)(phenyl)methylene)-2-(trifluoromethyl)oxazolidine (2o)

Prepared according to the modified general procedure B5 using N-benzyl-3-phenylprop-2-yn-1-amine (430 μL, 2.0 mmol, 1.0 equiv.) and 2-iodofluorobenzene (303 μL, 2.60

mmol, 1.3 equiv). The reaction was conducted at 60 °C using 1,2-dichloroethane as the solvent. The crude material was purified by flash column chromatography (pentane/EtOAc gradient 100:0 to 100:3) to give the corresponding olefin **2o** (520 mg, 1.26 mmol, 63 % yield) as a colorless oil. The enantiomeric ratio was determined to be 86.0:14.0 by HPLC analysis on a Daicel Chiralpak IB N-5 column: 99:1 hexane/IPA, flow rate 1 mL/min, λ = 254 nm: τ_{Minor} = 7.6 min, τ_{Major} = 9.0 min.

^{1}H NMR (400 MHz, Chloroform-*d*) δ 7.43 – 7.26 (m, 10H, Ar*H*), 7.21 – 7.06 (m, 4H, Ar*H*), 5.19 (q, *J* = 5.4 Hz, 1H, C*H*CF$_3$), 3.98 (d, *J* = 13.2 Hz, 1H, PhCH_aH$_b$), 3.90 (d, *J* = 13.3 Hz, 1H, PhCH$_a$$H_b$), 3.85 (d, *J* = 16.1 Hz, 1H, NCH_aH$_b$C=C), 3.43 (dd, *J* = 16.0, 1.4 Hz, 1H, NCH$_a$$H_b$C=C).

^{13}C{^{1}H} NMR (101 MHz, Chloroform-*d*) δ 160.5 (d, *J* = 246.1 Hz), 149.8, 137.7, 137.0, 132.9 (d, *J* = 3.2 Hz), 129.5 (d, *J* = 8.0 Hz), 128.9, 128.8, 128.4, 128.2, 128.1, 127.1 (d, *J* = 16.4 Hz), 126.5, 124.6 (d, *J* = 3.6 Hz), 122.74 (q, *J* = 283.8 Hz), 116.2 (d, *J* = 22.5 Hz), 106.0, 94.8 (q, *J* = 34.5 Hz), 60.7, 55.1.

^{19}F NMR (376 MHz, Chloroform-*d*) δ -80.3 (d, *J* = 5.4 Hz), -113.7 – -115.9 (m).

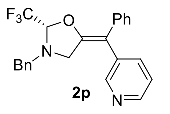

2p

(*S*,*E*)-3-Benzyl-5-(phenyl(pyridin-3-yl)methylene)-2-(trifluoromethyl)oxazolidine (2p)

Prepared according to the general procedure B5 using N-benzyl-3-phenylprop-2-yn-1-amine (430 µL, 0.40 mmol, 1.0 equiv.) and 3-iodopyridine (562 mg, 2.60 mmol, 1.3 equiv). The reaction was conducted at 60 °C using 1,2-dichloroethane as the solvent. The crude material was purified by flash column chromatography (pentane/EtOAc gradient 100:0 to 50:50) to give the corresponding olefin **2p** (435 mg, 1.10 mmol, 55 % yield) as an orange solid. The enantiomeric ratio was determined to be 77.0:23.0 by HPLC analysis on a Daicel Chiralpak IB N-5 column: 90:10 hexane/IPA, flow rate 1 mL/min, λ = 254 nm: τ_{Minor} = 9.9 min, τ_{Major} = 18.1 min.

^{1}H NMR (400 MHz, Chloroform-*d*) δ 8.49 (m, 2H, Ar*H*), 7.45 (dt, *J* = 7.9, 1.9 Hz, 1H, Ar*H*), 7.39 – 7.12 (m, 11H, Ar*H*), 5.17 (q, *J* = 5.2 Hz, 1H, C*H*CF$_3$), 4.01 (d, *J* = 13.3 Hz, 1H, PhCH_aH$_b$), 3.97 – 3.85 (m, 2H, PhCH$_a$$H_b$ and NCH_aH$_b$C=C), 3.55 (dd, *J* = 15.7, 1.4 Hz, 1H, NCH$_a$$H_b$C=C).

^{13}C{^{1}H} NMR (101 MHz, Chloroform-*d*) δ 150.8, 149.7, 148.4, 137.8, 137.7, 136.7, 136.0, 129.1, 128.9, 128.8, 128.3, 128.2, 126.8, 123.7, 122.7 (q, *J* = 283.8 Hz), 109.8, 94.2 (q, *J* = 34.5 Hz), 60.5, 54.8.

^{19}F{^{1}H} NMR (376 MHz, Chloroform-*d*) δ -80.3.

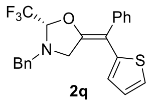

2q

(*S*,*E*)-3-Benzyl-5-(phenyl(thiophen-2-yl)methylene)-2-(trifluoromethyl)oxazolidine (2q)

Prepared according to the general procedure B5 using N-benzyl-3-phenylprop-2-yn-1-amine (430 µL, 2.00 mmol, 1.0 equiv.) and 2-iodothiophene (287 µL, 2.60 mmol, 1.3 equiv). The reaction was conducted at 60 °C using DCE as the solvent. The crude material was purified by flash column chromatography (pentane/EtOAc gradient 100:0 to 100:3) to give the corresponding olefin **2q** (640 mg, 1.60 mmol, 80 % yield) as a brown solid. The enantiomeric ratio was determined to be 94:6 by HPLC analysis on a Daicel IB N-5 column: 99:1 hexane/IPA, flow rate 1 mL/min, λ = 254 nm: τ_{Minor} = 9.3 min, τ_{Major} = 10.5 min.

^{1}H NMR (400 MHz, Chloroform-*d*) δ 7.44 – 7.29 (m, 9H, Ar*H*), 7.28 – 7.23 (m, 2H, Ar*H*), 6.97 (dd, *J* = 5.2, 3.5 Hz, 1H, Ar*H*), 6.76 (dd, *J* = 3.6, 1.2 Hz, 1H, Ar*H*), 5.11 (q, *J* = 5.3 Hz, 1H, C*H*CF$_3$), 4.12 (dd, *J* = 16.1, 1.0 Hz, 1H, NCH_aH$_b$C=C), 4.01 (d, *J* = 13.3 Hz, 1H, PhCH_aH$_b$), 3.94 (d, *J* = 13.3 Hz, 1H, PhCH$_a$$H_b$), 3.80 (dd, *J* = 16.1, 1.4 Hz, 1H, NCH$_a$$H_b$C=C).

^{13}C{^{1}H} NMR (101 MHz, Chloroform-*d*) δ 149.8, 142.3, 138.3, 136.9, 129.1, 128.8, 128.2 (2C), 128.1, 127.1, 126.98, 126.91, 125.1, 122.7 (q, *J* = 283.6 Hz), 107.1, 94.1 (q, *J* = 34.5 Hz), 60.7, 55.2.

^{19}F{^{1}H} NMR (376 MHz, Chloroform-*d*) δ -80.4.

C. Optimization Studies

C.1. Stereoselective cyclopropanation

The optimization reactions were conducted on the model oxazolidine **2a** (41 mg, 0.10 mmol, 1.0 equiv.). Reactions were performed in 8 mL round-bottom µW vials equipped with Teflon-coated magnetic stirring bars. The yields were determined by ^{1}HNMR analysis of the crude mixture using 1.0 equiv. of trichloroethylene as the internal standard (IS). Reaction conditions were chosen according to literature precedents.[10,11]

Table S1. Stereoselective Simmons-Smith cyclopropanation of oxazolidine **2a.**

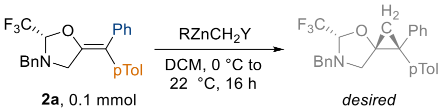

Entry	Conditions	SM,[a] %	Product,[a] %
1	CH_2I_2 (2.0 equiv.), Et_2Zn (2.5 equiv.)	95	0
2	CH_2ICl (5.0 equiv.), Et_2Zn (2.5 equiv.)	22	0
3	CH_2I_2 (2.5 equiv.), Et_2Zn (2.5 equiv.), TFA (1.25 equiv.)	<5	0
4	CH_2I_2 (2.0 equiv.)	>95	0

[a]NMR yield using trichloroethylene as an internal standard

Table S2. Stereoselective cyclopropanation of oxazolidine **2a** using diazo compounds.

The optimization reactions were conducted on the model oxazolidine **2a** (41 mg, 0.10 mmol, 1.0 equiv.). Reactions were performed in 8 mL round-bottom µW vials equipped with Teflon-coated magnetic stirring bars. The diazo compound was added dropwise over 50 minutes using a syringe pump. The yields were determined by ^{1}HNMR analysis of the crude mixture using 1.0 equiv. of trichloroethylene as the internal standard (IS).

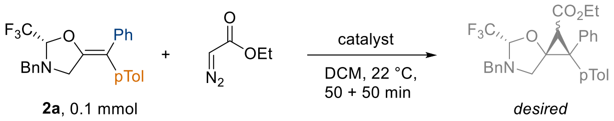

Entry	Catalyst	SM,[a] %	Product,[a] %
1	$(Rh(OAc)_2)_2$ (2 mol %)	71	<10
2	$(CuOTf)_2 \cdot C_6H_6$ (5 mol %)	>95	0
3	$Pd(OAc)_2$ (10 mol %)	>95	0
4	Rh_2esp_2 (2 mol %)	82	<10

5	$(RhOct_2)_2$ (2 mol %)	>95	0

[a]NMR yield using trichloroethylene as an internal standard.

The optimization reactions were conducted on the model oxazolidine **2a** (41 mg, 0.10 mmol, 1.0 equiv.). Reactions were performed in 8 mL round-bottom µW vials equipped with Teflon-coated magnetic stirring bars. The yields were determined by ^{1}HNMR analysis of the crude mixture using 1.0 equiv. of trichloroethylene as the internal standard (IS). Reaction conditions were chosen according to literature precedents.[12,13]

Table S3. Stereoselective cyclopropanation of oxazolidine **2a** with free carbenes.

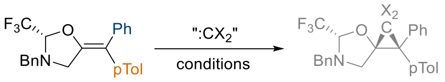

2a, 0.1 mmol — *desired*

Entry	Conditions	SM,[a] %	Product,[a] %
1	$CHBr_3$ (50 equiv.), 50 % aq. NaOH, $BnNEt_3^+Cl^-$ (30 mol %); 22 °C, 16 h	35	0
4	KOtBu (1.2 equiv.), $CHBr_3$ (1.1 equiv.), pentane 22 °C, 1 h	>95	0
3[b]	$TMSCF_3$ (2.5 equiv.), NaI (0.2 equiv.); THF, 65 °C, 16 h	>95	0
4[c]	$TMSCF_3$ (2.0 equiv.), NaI (2.2 equiv.); THF, 65 °C, 16 h	>95	0

[a]NMR yield using trichloroethylene as an internal standard. [b]Conditions reported for alkenes. [c]Conditions reported for alkynes.

Table S4. Stereoselective cyclopropanation of oxazolidine **2a** with dichlorocarbene.

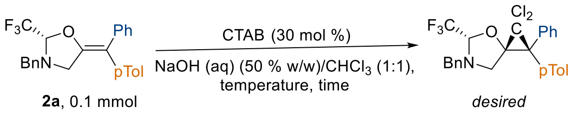

2a, 0.1 mmol — *desired*

Entry	Scale (mmol)	T (°C)	Time (h)	3a (%)[a]	dr[b]
1	0.05	22	1	67	19:1
4	0.20	22	1	85	18:1
3[c]	0.20	22	1	86	19:1
4[d]	0.20	22	16	84	19:1

5	0.20	0	16	53	21:1

[a]NMR yield using trichloroethylene as an internal standard. [b]dr determined by the integration of the crude ^{19}F NMR [c]Conditions reported for alkenes. [d]Conditions reported for alkynes.

C.2. Stereoselective epoxidation

The optimization reactions were conducted on the model oxazolidine **2a** (21 mg, 0.050 mmol, 1.0 equiv.). Reactions were performed in 8 mL round-bottom µW vials equipped with Teflon-coated magnetic stirring bars. The yields were determined by ^{1}HNMR analysis of the crude mixture using 1.0 equiv. of trichloroethylene as the internal standard (IS). The *dr* was determined by the integration of the crude ^{19}F NMR spectra.

Table S5. Stereoselective epoxidayion of oxazolidine **2a** with mCPBA.

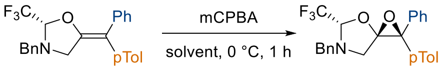

2a, 0.05 mmol **4a**

Entry	Solvent	With sat. aq. $NaHCO_3$	mCPBA equiv.	SM, %	NMR yield, %	*dr*
1	DCM	+	1.2	5	75	17:1
2	DCM	+	1.0	20	62	30:1
3	DCM	+	1.5	0	72	18:1
4[a]	DCM	+	1.2	13	87	21:1
5[a]	Et_2O	+	1.2	72	28	40:1
6[a]	Toluene	+	1.2	13	85	68:1
7[a]	Toluene	-	1.2	2	98	60:1
8[a,b]	Toluene	-	1.2	2	98 (88)[c,d]	58:1
9[a,c,e]	Toluene	-	1.2	3	97 (85)[c,d]	54:1

[a]Addition of the mCPBA solution over 10 minutes [b]0.2 mmol scale [c]Isolated yield. [d]Conservation of *er* confirmed [e]0.4 mmol scale

We found that it was possible to isolate the epoxide product, the diastereomers were non-separable and the product itself was sensitive to silica. Tether cleavage was found to be extremely facile in a TFA/MeOH system, giving the product in quant. yield. Here the product *er* is lower than the starting material *er*, because the two diastereomers contribute to opposite stereochemistry at C1 in the product **5a**.

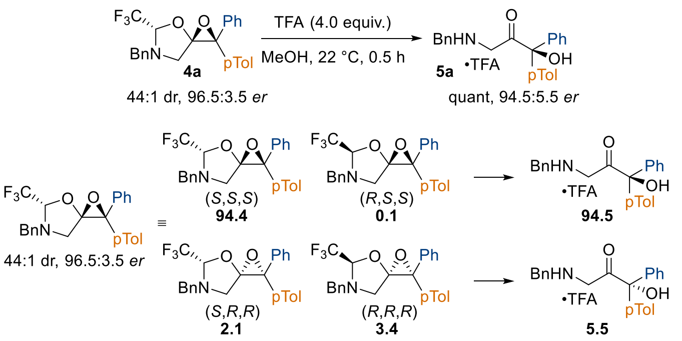

Scheme 13. Stereoselective epoxidation of oxazolidine **2a**.

Due to the easiness of the tether cleavage, we started to look for a telescoped transformation starting from the oxazolidine **2a**.

Table S6. Stereoselective epoxidation of oxazolidine **2a**.

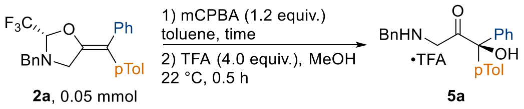

Entry	T, h	SM, %	1st step NMR Y %	dr	2st step NMR Y %	Isol. Yield, %	Purification
1	1	17	85	47:1	83	56	**C**
2	2	3	97	58:1	92	74	**C**
3	4	26	73	55:1	-	-	**C**
4	2	8	93	48:1	76	failed	**C**
5	16	3	97	50:1	90	64	**C**
6	1+1@22 °C	2	99	64:1	96	65	**C**
7	1+1@22 °C	3	97	52:1	92	74	**P/T**
8	1+1@22 °C	4	96	47:1	-	80	**P/T**
9	1+1@22 °C	2	98	42:1	90	77	**P/T**
10	1+1@22 °C	2	98	46:1	94	82	**P/T**

[a]Addition of the mCPBA solution over 10 minutes [b]0.2 mmol scale. [c]Conservation of er confirmed [d]0.4 mmol scale. **C** – chromatography. **P/T** – precipitation/trituration.

D. Asymmetric cyclopropanation and epoxidation

D.1. General Procedure for the Asymmetric cyclopropanation of the Tetrasubstituted Olefins.

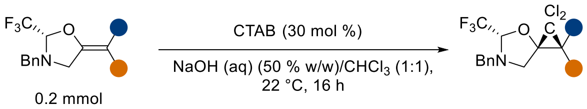

Scheme 13. Enantioselective cyclopropanation of the tetrasubstituted olefins

In an oven-dried 8 mL microwave vial equipped with a Teflon coated stirring bar was charged cetyl trimethyl ammonium bromide (22 mg, 30 mol %, 0.060 mmol), the oxazoline (0.200 mmol, 1.0 equiv), chloroform (1.6 mL) and $NaOH_{(aq)}$ (50 % w/w, 1.6 mL). The vial was then sealed and vigorously stirred at 22 °C for 16 h. **Careful: during the reaction the vial becomes pressurized!** Next, the reaction mixture was filtered through celite and the cake was washed with DCM (3 × 5 mL) and water (3 × 5 mL). The organic layer was separated and the aqueous layer was extracted with DCM (2 × 5 mL). The combined organic layers were dried over anhydrous Na_2SO_4 and concentrated in vacuo. The crude material was purified by flash column chromatography on silica gel to afford the corresponding product. Absolute and relative configuration were assigned based on the relative and absolute configuration of the starting materials.

D.2. Characterization of the cyclopropanation products

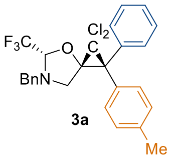

3a

(2*S*,3*R*,5*S*)-6-Benzyl-1,1-dichloro-2-phenyl-2-(p-tolyl)-5-(trifluoromethyl)-4-oxa-6-azaspiro[2.4]heptane (3a)

Prepared according to the general procedure D1 using **2a** (82 mg, 0.20 mmol, 1.0 equiv., 97.0:3.0 *er*). The *dr* was determined to be 19:1 by the integration of the crude ^{19}F NMR spectra. The crude material was purified by column chromatography (0 – 5 % (v/v) Et_2O in pentane). Product **3a** (74 mg, 0.15 mmol, 75 % yield) was isolated as an amorphous off-white solid. The enantiomeric ratio was determined to be 97.0:3.0 by HPLC analysis on a Daicel Chiralpak IB N-5 column: 99:1 hexane/IPA, flow rate 1 mL/min, λ = 230 nm: τ_{Minor} = 5.0 min, τ_{Major} = 6.7 min.

Rf (5 % Et_2O/Pent) = 0.70.

$[\alpha]D^{20}$ = +26.2 (c = 0.56, $CHCl_3$, 97.0:3.0 *er*).

^{1}H NMR (400 MHz, $CDCl_3$) δ 7.49 – 7.44 (m, 2H, Ar*H*), 7.44 – 7.39 (m, 2H, Ar*H*), 7.35 – 7.27 (m, 7H, Ar*H*), 7.23 – 7.15 (m, 1H, Ar*H*), 7.07 (d, *J* = 8.0 Hz, 2H, Ar*H*), 5.06 (q, *J* = 5.6 Hz, 1H, C*H*CF3), 4.07 (d, *J* = 13.0 Hz, 1H, PhCH_aH_bN), 4.02 (d, *J* = 13.0 Hz, 1H, PhCH_aH_bN), 3.92 (dq, *J* = 13.7, 1.5 Hz, 1H, NCH_aH_bC), 3.19 (dq, *J* = 13.7, 1.5 Hz, 1H, NCH_aH_bC)), 2.24 (s, 3H, ArCH_3).
^{13}C{^{1}H} NMR (101 MHz, $CDCl_3$) δ 138.6, 137.3, 137.2, 135.9, 129.50, 129.49, 129.0, 128.8, 128.7, 128.5, 128.1, 127.3, 123.1 (d, J_{C-F} = 283.5 Hz), 94.7 (q, J_{C-F} = 34.2 Hz), 74.4, 69.8, 60.0, 53.5, 45.0, 21.2.
^{19}F NMR (376 MHz, $CDCl_3$) δ –79.6 (dt, *J* = 5.6, 1.5 Hz, 3F, CHCF_3).

IR (cm^{-1}) 3028 (w), 2927 (w), 2854 (w), 1504 (w), 1288 (m), 1180 (m), 1142 (s).

HRMS (ESI/QTOF) m/z: $[M + H]^+$ Calcd for $C_{26}H_{23}Cl_2F_3NO^+$ 492.1103; Found 492.1107.

1 mmol scale:

An oven-dried 25 mL round bottom flask equipped with a Teflon coated stirring bar was charged with cetyl trimethyl ammonium bromide (109 mg, 0.300 mmol, 30 mol %), the oxazoline **2a** (409 mg, 1.00 mmol, 1.0 equiv., 99.0:1.0 *er*), chloroform (8.0 mL) and $NaOH_{(aq)}$ (50 % w/w, 8.0 mL). The flask was then sealed and vigorously stirred at 22 °C for 16 h with a nitrogen balloon. Next, the reaction mixture was filtered through celite and the cake was washed with DCM (3 × 15 mL) and water (3 × 15 mL). The organic layer was separated and the aqueous layer was extracted with DCM (2 × 15 mL). The combined organic layers were dried over anhydrous Na_2SO_4 and concentrated in vacuo. The *dr* was determined to be 19:1 by the integration of the crude ^{19}F NMR spectra. The crude material was purified by flash column chromatography on silica gel to afford the product **3a** (356 mg, 0.723 mmol, 72 %). The enantiomeric ratio was determined to be 99.0:1.0 by HPLC analysis on a Daicel Chiralpak IB N-5 column: 99:1 hexane/IPA, flow rate 1 mL/min, λ = 230 nm: τ_{Minor} = 5.0 min, τ_{Major} = 6.7 min. Absolute and relative configuration were assigned based on the relative and absolute configuration of the starting material.

$[\alpha]D^{20}$ = +29.8 (c = 0.54, $CHCl_3$, 99.0:1.0 *er*).

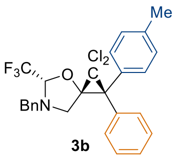

3b

(2*R*,3*R*,5*S*)-6-Benzyl-1,1-dichloro-2-phenyl-2-(p-tolyl)-5-(trifluoromethyl)-4-oxa-6-azaspiro[2.4]heptane (3b)

Prepared according to the general procedure D1 using **2b** (82 mg, 0.20 mmol, 1.0 equiv., 94.5:5.5 *er*). The *dr* was determined to be 22:1 by the integration of the crude ^{19}F NMR spectra. The crude material was purified by column chromatography (0 – 5 % (v/v) Et_2O in pentane) to give **3b** (64 mg, 0.13 mmol, 65 % yield) as an amorphous off-white solid. The enantiomeric ration was determined to be 94.5:5.5 by HPLC analysis on a Daicel Chiralpak IB N-5 column: 100:0 hexane/IPA, flow rate 1 mL/min, λ = 230 nm: τ_{Minor} = 14.3 min, τ_{Major} = 15.8 min.

Rf (5 % Et_2O/Pent) = 0.69.

$[\alpha]D^{20}$ = +17.5 (c = 0.47, $CHCl_3$, 94.5:5.5 *er*).

^{1}H NMR (400 MHz, $CDCl_3$) δ 7.47 – 7.21 (m, 11H, Ar*H*), 7.16 (t, *J* = 7.4 Hz, 1H, Ar*H*), 7.12 (d, *J* = 7.9 Hz, 2H, Ar*H*), 5.05 (q, *J* = 5.6 Hz, 1H, C*H*CF_3), 4.07 (d, *J* = 13.1 Hz, 1H, PhCH_aH_bN), 4.02 (d, *J* = 13.1 Hz, 1H, PhCH_aH_bN), 3.92 (dq, *J* = 13.6, 1.5 Hz, 1H, NCH_aH_bC), 3.19 (dq, *J* = 13.6, 1.5 Hz, 1H, NCH_aH_bC), 2.27 (s, 3H, ArCH_3)
^{13}C{^{1}H} NMR (101 MHz, $CDCl_3$) δ 139.0, 137.2, 137.1, 135.4, 129.4, 129.3, 129.0, 128.9, 128.8, 128.7, 128.1, 127.4, 123.0 (q, $J_{C\text{-}F}$ = 283.3 Hz), 94.6 (q, $J_{C\text{-}F}$ = 34.2 Hz) 74.4, 69.7, 60.0, 53.5, 45.0, 21.3.
^{19}F NMR (376 MHz, $CDCl_3$) δ –79.6 (dt, *J* = 5.6, 1.5 Hz, 3F, CHCF_3).

IR (cm^{-1}) 3028 (w), 2927 (w), 2854 (w), 1500 (w), 1450 (w), 1288 (m), 1180 (s), 1142 (s).

HRMS (ESI/QTOF) m/z: $[M + H]^+$ Calcd for $C_{26}H_{23}Cl_2F_3NO^+$ 492.1103; Found 492.1101.

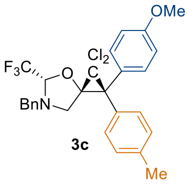

3c

(2*R*,3*R*,5*S*)-6-Benzyl-1,1-dichloro-2-(4-methoxyphenyl)-2-(p-tolyl)-5-(trifluoromethyl)-4-oxa-6-azaspiro[2.4]heptane (3c)

Prepared according to the general procedure D1 using **2c** (88 mg, 0.20 mmol, 1.0 equiv., 94.0:6.0 *er*). The *dr* was determined to be 27:1 by the integration of the crude ^{19}F NMR spectra. The crude material was purified by column chromatography (0 – 5 % (v/v) Et_2O in pentane) to give **3c** (69 mg, 0.13 mmol, 66 % yield) as an amorphous pale-yellow solid. The enantiomeric ratio was determined to be 95.5:4.5 by HPLC analysis on a Daicel Chiralpak IB N-5 column: 99:1 hexane/IPA, flow rate 1 mL/min, λ = 230 nm: τ_{Minor} = 6.0 min, τ_{Major} = 10.8 min. Absolute and relative configuration were assigned based on the relative and absolute configuration of the starting material.

Rf (5 % Et_2O/Pent) = 0.46.

$[\alpha]D^{20}$ = +22.0 (c = 0.48, $CHCl_3$, 95.5:4.5 *er*).

^{1}H NMR (400 MHz, $CDCl_3$) δ 7.45 – 7.35 (m, 4H, Ar*H*), 7.35 – 7.23 (m, 5H, Ar*H*), 7.07 (d, *J* = 7.9 Hz, 2H, Ar*H*), 6.90 – 6.78 (m, 2H, Ar*H*), 5.05 (d, *J* = 5.5 Hz, 1H, C*H*CF_3), 4.07 (d, *J* = 13.1 Hz, 1H, PhCH_aH_bN), 4.02 (d, *J* = 13.1 Hz, 1H, PhCH_aH_bN), 3.90 (dq, *J* = 13.8, 1.5 Hz, 1H, NCH_aH_bC), 3.74 (s, 3H, ArOCH_3), 3.18 (dd, *J* = 13.7, 1.5 Hz, 1H, NCH_aH_bC), 2.24 (s, 3H, ArCH_3).
^{19}F NMR (376 MHz, $CDCl_3$) δ –79.6 (dt, *J* = 5.6, 1.5 Hz, 3F, CHCF_3).

^{13}C{^{1}H} NMR (101 MHz, $CDCl_3$) δ 158.7, 137.21, 137.16, 136.2, 132.2, 130.9, 130.6, 129.5, 129.0, 128.7, 128.0, 123.1 ($J_{C\text{-}F}$ = 283.4 Hz), 114.0, 94.6 ($J_{C\text{-}F}$ = 34.3 Hz), 74.4, 69.9, 60.0, 55.3, 53.4, 44.5, 21.2.
IR (cm^{-1}) 3032 (w), 2927 (w), 2850 (w), 1512 (m), 1327 (s), 1173 (s), 1138 (s).

HRMS (ESI/QTOF) m/z: $[M + H]^+$ Calcd for $C_{27}H_{25}Cl_2F_3NO_2^+$ 522.1209; Found 522.1216.

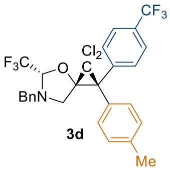

3d

(2*R*,3*R*,5*S*)-6-Benzyl-1,1-dichloro-2-(p-tolyl)-5-(trifluoromethyl)-2-(4-(trifluoromethyl)phenyl)-4-oxa-6-azaspiro[2.4]heptane (3d)

Prepared according to the general procedure D1 using **2d** (95 mg, 0.20 mmol, 1.0 equiv., 94.0:6.0 *er*). The *dr* was determined to be 18:1 by the integration of the crude ^{19}F NMR spectra. The crude material was purified by column chromatography (0 – 5 % (v/v) Et_2O in pentane) to give **3d** (85 mg, 0.15 mmol, 76 % yield) as an amorphous off-white solid. The enantiomeric ratio was determined to be 94.0:6.0 by HPLC analysis on a Daicel Chiralpak IB N-5 column: 99:1 hexane/IPA, flow rate 1 mL/min, λ = 230 nm: τ_{Minor} = 4.6 min, τ_{Major} = 5.0 min.

Rf (5 % Et_2O/Pent) = 0.74.

$[\alpha]D^{20}$ = +29.6 (c = 0.57, $CHCl_3$, 94.0:6.0 *er*).

^{1}H NMR (400 MHz, $CDCl_3$) δ 7.61 – 7.52 (m, 4H, Ar*H*), 7.44 – 7.38 (m, 2H, Ar*H*), 7.36 – 7.31 (m, 2H, Ar*H*), 7.31 – 7.26 (m, 3H, Ar*H*), 7.12 – 7.06 (m, 2H, Ar*H*), 5.07 (q, *J* = 5.6 Hz, 1H, C*H*CF_3), 4.06 (d, *J* = 13.0 Hz, 1H, PhCH_aH_bN), 4.02 (d, *J* = 13.0 Hz, 1H, PhCH_aH_bN), 3.92 (dq, *J* = 13.7, 1.5 Hz, 1H, NCH_aH_bC), 3.21 (dq, *J* = 13.7, 1.5 Hz, 1H, NCH_aH_bC), 2.25 (s, 3H, ArCH_3).

^{19}F NMR (376 MHz, $CDCl_3$) δ -62.7 (s, 3F, ArCF_3), –79.6 (dt, J = 5.6, 1.5 Hz, 3F, CHCF_3).
^{13}C{^{1}H} NMR (101 MHz, $CDCl_3$) δ 142.3, 137.9, 137.0, 134.9, 129.9, 129.8, 129.4 (q, J_{C-F} = 32.4 Hz), 129.0, 128.8, 128.8, 128.2, 125.5 (q, J_{C-F} = 4.7 Hz), 124.2 (q, J_{C-F} = 283.4 Hz), 123.5 (q, J_{C-F} = 283.4 Hz), 94.8 (q, J_{C-F} = 34.3 Hz), 74.3, 69.2, 60.1, 53.4, 44.6, 21.2.
IR (cm^{-1}) 3028 (w), 2927 (w), 2858 (w), 1512 (w), 1327 (s), 1173 (s), 1134 (s), 1288 (m).
HRMS (ESI/QTOF) m/z: [M + H]$^+$ Calcd for $C_{27}H_{22}Cl_2F_6NO^+$ 560.0977; Found 560.0981.

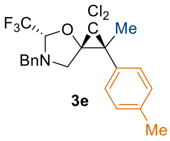

3e

(2*S*,3*R*,5*S*)-6-Benzyl-1,1-dichloro-2-methyl-2-(p-tolyl)-5-(trifluoromethyl)-4-oxa-6-azaspiro[2.4]heptane (3e)

Prepared according to the general procedure D1 using **2e** (69 mg, 0.20 mmol, 1.0 equiv., 85.5:14.5 *er*). The *dr* was determined to be 12:1 by the integration of the crude ^{19}F NMR spectra. The crude material was purified by column chromatography (0 – 5 % (v/v) Et_2O in pentane) to give **3e** (59 mg, 0.14 mmol, 69 % yield) as a colorless oil. The enantiomeric ratio was determined to be 84.5:15.5 by HPLC analysis on a Daicel Chiralpak IB N-5 column: 100:0 hexane/IPA, flow rate 1 mL/min, λ = 230 nm: τ_{Minor} = 7.8 min, τ_{Major} = 8.5 min.

Rf (5 % Et_2O/Pent) = 0.77.

[α]D^{20} = -12.9 (c = 0.51, $CHCl_3$, 84.5:15.5 *er*).

^{1}H NMR (400 MHz, $CDCl_3$) δ 7.42 – 7.37 (m, 2H, Ar*H*), 7.35 – 7.30 (m, 2H, Ar*H*), 7.30 – 7.24 (m, 1H, Ar*H*), 7.13 (s, 4H, Ar*H*), 5.00 (q, J = 5.2 Hz, 1H, C*H*CF_3), 4.09 (d, J = 13.0 Hz, 1H, PhC$H_a$$H_b$N), 3.93 (d, J = 13.0 Hz, 1H, PhC$H_a$$H_b$N), 3.51 (dd, J = 12.5, 1.2 Hz, 1H, NC$H_a$$H_b$C), 3.03 (dd, J = 12.5, 1.2 Hz, 1H, NC$H_a$$H_b$C), 2.31 (s, 3H, Ar$CH_3$), 1.62 (s, 3H, C$H_3$).
^{19}F NMR (376 MHz, $CDCl_3$) δ –80.6 (dt, J = 5.2, 1.2 Hz, 3F, CHCF_3).
^{13}C{^{1}H} NMR (101 MHz, $CDCl_3$) δ 137.3, 137.2, 135.9, 129.4, 128.91, 128.87, 128.7, 128.0, 123.1 (q, J = 283.2 Hz), 93.7 (q, J = 34.1 Hz), 73.7, 68.9, 59.8, 52.8, 38.6, 22.5, 21.3.
IR (cm^{-1}) 3028 (w), 2931 (w), 2854 (w), 1724 (w), 1450 (w), 1288 (m), 1173 (s), 1153 (s).
HRMS (ESI/QTOF) m/z: [M + H]$^+$ Calcd for $C_{21}H_{21}Cl_2F_3NO^+$ 430.0947; Found 430.0948.

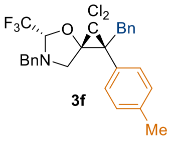

3f

(1*S*,3*R*,5*S*)-1,6-dibenzyl-2,2-dichloro-1-(p-tolyl)-5-(trifluoromethyl)-4-oxa-6-azaspiro[2.4]heptane (3f)

Prepared according to the general procedure D1 using **2f** (69 mg, 0.20 mmol, 1.0 equiv., 92.0:8.0 *er*). The *dr* was determined to be 19:1 by the integration of the crude ^{19}F NMR spectra. The crude material was purified by column chromatography (0 – 5 % (v/v) Et_2O in pentane) to give **3f** (81 mg, 0.16 mmol, 82 % yield) as a colorless oil. The enantiomeric ratio was determined to be 92.0:8.0 by HPLC analysis on a Daicel Chiralpak IB N-5 column: 100:0 hexane/IPA, flow rate 1 mL/min, λ = 230 nm: τ_{Minor} = 10.6 min, τ_{Major} = 13.1 min.

Rf (5 % Et_2O/Pent) = 0.77.

[α]D^{20} = -5.0 (c = 0.56, $CHCl_3$, 92.0:8.0 *er*).

^{1}H NMR (400 MHz, $CDCl_3$) δ 7.43 – 7.26 (m, 6H, Ar*H*), 7.14 – 7.05 (m, 2, Ar*H*), 6.96 – 6.89 (m, 2H, Ar*H*), 6.89 – 6.82 (m, 2H, Ar*H*), 6.71 (d, J = 8.1 Hz, 2H, Ar*H*), 5.07 (q, J = 5.3 Hz, 1H, C*H*CF_3), 4.03 (s, 2H, CH_2Ph), 3.56 (dq, J = 13.4, 1.2 Hz, 1H, NC$H_a$$H_b$C), 3.37 (d, J = 13.8 Hz, 1H, PhC$H_a$$H_b$N), 3.25 (d, J = 13.8 Hz, 1H, PhC$H_a$$H_b$N), 3.01 (dq, J = 13.4, 1.2 Hz, 1H, NC$H_a$$H_b$C), 2.24 (s, 3H, ArC$H_3$).
^{19}F NMR (376 MHz, $CDCl_3$) δ –80.1 (dt, J = 5.2, 1.2 Hz, 3F, CHCF_3).
^{13}C{^{1}H} NMR (101 MHz, $CDCl_3$) δ 137.3, 137.13, 137.08, 132.9, 130.2, 130.1, 129.0, 128.7, 128.6, 128.0, 127.9, 126.4, 123.0 (d, J = 282.2 Hz), 94.0 (d, J = 34.0 Hz), 73.9, 69.8, 59.9, 52.6, 42.8, 41.2, 21.3.
IR (cm^{-1}) 3032 (w), 2927 (w), 2858 (w), 1728 (w), 1450 (w), 1292 (m), 1176 (s), 1146 (s), 1219 (m).
HRMS (ESI/QTOF) m/z: M + H]$^+$ Calcd for $C_{27}H_{25}Cl_2F_3NO^+$ 506.1260; Found 506.1258.

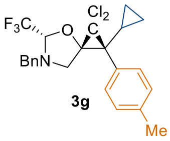

3g

(2*S*,3*R*,5*S*)-6-Benzyl-1,1-dichloro-2-cyclopropyl-2-(p-tolyl)-5-(trifluoromethyl)-4-oxa-6-azaspiro[2.4]heptane (3g)

Prepared according to the general procedure D1 using **2g** (75 mg, 0.20 mmol, 1.0 equiv., 88.0:12.0 *er*). The *dr* was determined to be 28:1 by the integration of the crude ^{19}F NMR spectra. The crude material was purified by column chromatography (0 – 5 % (v/v) Et_2O in pentane) to give **3g** (61 mg, 0.13 mmol, 67 % yield) as a colorless oil. The enantiomeric ratio was determined to be 88.0:12.0 by HPLC analysis on a Daicel Chiralpak IB N-5 column: 100:0 hexane/IPA, flow rate 1 mL/min, λ = 210 nm: τ_{Minor} = 8.1 min, τ_{Major} = 9.1 min.

Rf (5 % Et_2O/Pent) = 0.77.

[α]D[20] = -12.1 (c = 0.45, $CHCl_3$, 88.0:12.0 *er*).

^{1}H NMR (400 MHz, $CDCl_3$) δ 7.41 – 7.37 (m, 2H, Ar*H*), 7.35 – 7.23 (m, 3H, Ar*H*), 7.11 – 7.03 (m, 2H, Ar*H*), 7.01 – 6.93 (m, 2H, Ar*H*), 5.03 (q, *J* = 5.2 Hz, 1H, C*H*CF_3), 4.09 (d, *J* = 13.0 Hz, 1H, PhCH_aH_bN), 3.93 (d, *J* = 13.0 Hz, 1H, PhCH_aH_bN), 3.46 (dq, *J* = 12.7, 1.2 Hz, 1H, NCH_aH_bC), 2.99 (dq, *J* = 12.7, 1.2 Hz, 1H, NCH_aH_bC), 2.29 (s, 3H, ArCH_3), 1.46 (tt, *J* = 8.4, 5.4 Hz, 1H, C*H*(CH_aH_bCH_aH_b)), 0.51 (dddd, *J* = 9.0, 8.4, 5.4, 4.4 Hz, 1H, CH(CH_aH_bCH_aH_b)), 0.44 (dddd, *J* = 9.0, 8.4, 5.4, 4.4 Hz, 1H, CH(CH_aH_bCH_aH_b)), 0.11 (dtd, *J* = 9.9, 5.4, 4.4 Hz, 1H, CH(CH_aH_bCH_aH_b)), -0.20 (dtd, *J* = 9.9, 5.4, 4.4 Hz, 1H, CH(CH_aH_bCH_aH_b)).

^{19}F NMR (376 MHz, $CDCl_3$) δ –80.4 (dt, *J* = 5.2, 1.2 Hz, 3F, CHCF_3).

^{13}C{^{1}H} NMR (101 MHz, $CDCl_3$) δ 137.6, 137.3, 131.1, 129.7, 128.9, 128.7, 128.6, 127.9, 123.1 (q, $J_{C\text{-}F}$ = 283.5 Hz), 93.8 (q, $J_{C\text{-}F}$ = 34.2 Hz), 74.3, 69.8, 59.8, 53.3, 43.0, 21.3, 13.2, 2.7, 0.9.

IR (cm^{-1}) 3082 (w), 3024 (w), 1292 (m), 1161 (s), 999 (m).

HRMS (ESI/QTOF) m/z: $[M + H]^+$ Calcd for $C_{23}H_{23}Cl_2F_3NO^+$ 456.1103; Found 456.1099.

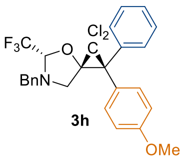

3h

(2*S*,3*R*,5*S*)-6-Benzyl-1,1-dichloro-2-(4-methoxyphenyl)-2-phenyl-5-(trifluoromethyl)-4-oxa-6-azaspiro[2.4]heptane (3h)

Prepared according to the general procedure D1 using **2h** (85 mg, 0.20 mmol, 1.0 equiv., 95.0:5.0 *er*). The *dr* was determined to be 22:1 by the integration of the crude ^{19}F NMR spectra. The crude material was purified by column chromatography (0 – 5 % (v/v) Et_2O in pentane) to give **3h** (59 mg, 0.12 mmol, 58 % yield) as an amorphous pale-yellow solid. The enantiomeric ratio was determined to be 95.0:5.0 by HPLC analysis on a Daicel Chiralpak IB N-5 column: 99:1 hexane/IPA, flow rate 1 mL/min, λ = 230 nm: τ_{Minor} = 7.1 min, τ_{Major} = 9.5 min.

Rf (5 % Et_2O/Pent) = 0.50.

[α]D[20] = +23.7 (c = 0.57, $CHCl_3$, 95.0:5.0 *er*).

^{1}H NMR (400 MHz, $CDCl_3$) δ 7.47 – 7.39 (m, 4H, Ar*H*), 7.36 – 7.27 (m, 7H, Ar*H*), 7.23 – 7.16 (m, 1H, Ar*H*), 6.83 – 6.75 (m, 2H, Ar*H*), 5.06 (q, *J* = 5.6 Hz, 1H, C*H*CF_3), 4.07 (d, *J* = 13.0 Hz, 1H, PhCH_aH_bN), 4.02 (d, *J* = 13.0 Hz, 1H, PhCH_aH_bN), 3.91 (dq, *J* = 13.8, 1.5 Hz, 1H, NCH_aH_bC), 3.72 (s, 3H, ArOCH_3), 3.20 (dq, *J* = 13.7, 1.5 Hz, 1H, NCH_aH_bC).

^{19}F NMR (376 MHz, $CDCl_3$) δ –79.6 (dt, *J* = 5.6, 1.5 Hz, 3F, CHCF_3).

^{13}C{^{1}H} NMR (101 MHz, $CDCl_3$) δ 158.8, 138.7, 137.2, 131.0, 130.1, 129.4, 129.0, 128.7, 128.5, 128.1, 127.2, 123.1 (d, *J* = 283.5 Hz), 114.2, 94.7 (q, *J* = 34.2 Hz), 74.4, 69.9, 60.0, 55.3, 53.5, 44.7.

IR (cm^{-1}) 3024 (w), 2927 (w), 2846 (w), 1512 (m), 1288 (m), 1250 (m), 1180 (s), 1142 (s).

HRMS (ESI/QTOF) m/z: $[M + H]^+$ Calcd for $C_{26}H_{23}Cl_2F_3NO_2^+$ 508.1052; Found 508.1052.

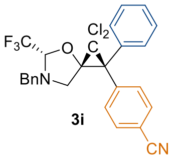

3i

4-((1*S*,3*R*,5*S*)-6-Benzyl-2,2-dichloro-1-phenyl-5-(trifluoromethyl)-4-oxa-6-azaspiro[2.4]heptan-1-yl)benzonitrile (3i)

Prepared according to the general procedure D1 using **2i** (84 mg, 0.20 mmol, 1.0 equiv., 85.0:15.0 *er*). The *dr* was determined to be 20:1 by the integration of the crude ^{19}F NMR spectra. The crude material was purified by column chromatography (0 – 10 % (v/v) Et_2O in pentane) to give **3i** (57 mg, 0.11 mmol, 57 % yield) as an amorphous off-white solid. The enantiomeric ratio was determined to be 86.0:14.0 by HPLC analysis on a Daicel Chiralpak IA column: 95:5 hexane/IPA, flow rate 1 mL/min, λ = 254 nm: τ_{Minor} = 6.1 min, τ_{Major} = 6.6 min.

Rf (5 % Et_2O/Pent) = 0.28.

[α]D[20] = +36.3 (c = 0.53, $CHCl_3$, 86.0:14.0 *er*).

^{1}H NMR (400 MHz, $CDCl_3$) δ 7.57 (d, *J* = 8.5 Hz, 2H, Ar*H*), 7.51 (d, *J* = 8.5 Hz, 2H, Ar*H*), 7.47 – 7.38 (m, 4H, Ar*H*), 7.38 – 7.28 (m, 5H, Ar*H*), 7.26 – 7.21 (m, 1H, Ar*H*), 5.08 (q, *J* = 5.5 Hz, 1H, C*H*CF_3), 4.04 (s, 2H, PhCH_2N), 3.84 (dq, *J* = 13.6, 1.5 Hz, 1H, NCH_aH_bC), 3.19 (dq, *J* = 13.6, 1.5 Hz, 1H, NCH_aH_bC).

^{19}F NMR (376 MHz, $CDCl_3$) δ –79.5 (dt, *J* = 5.6, 1.5 Hz, 3F, CHCF_3).

^{13}C{^{1}H} NMR (101 MHz, $CDCl_3$) δ 143.5, 136.9, 136.7, 132.5, 129.7, 129.5, 128.9, 128.7, 128.7, 128.1, 127.9, 122.8 (d, *J* = 283.2 Hz), 118.20, 111.47, 94.6 (q, *J* = 34.2 Hz), 74.1, 68.9, 59.9, 53.4, 45.0.

IR (cm^{-1}) 3028 (w), 2927 (w), 2854 (w), 1604 (w), 1500 (m), 1288 (m), 1180 (s), 1142 (s)

HRMS (ESI/QTOF) m/z: $[M + H]^+$ Calcd for $C_{26}H_{20}Cl_2F_3N_2O^+$ 503.0899; Found 503.0895.

(2*S*,3*R*,5*S*)-6-Benzyl-1,1-dichloro-2-phenyl-5-(trifluoromethyl)-2-(4-(trifluoromethyl)phenyl)-4-oxa-6-azaspiro[2.4]heptane (3j)

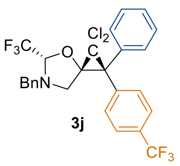

3j

Prepared according to the general procedure D1 using **2j** (93 mg, 0.20 mmol, 1.0 equiv., 91.0:9.0 *er*). The *dr* was determined to be 18:1 by the integration of the crude ^{19}F NMR spectra. The crude material was purified by column chromatography (0 – 5 % (v/v) Et_2O in pentane) to give **3j** (69 mg, 0.13 mmol, 63 % yield) as an amorphous off-white solid. The enantiomeric ratio was determined to be 91.0:9.0 by HPLC analysis on a Daicel Chiralpak IB N-5 column: 99:1 hexane/IPA, flow rate 1 mL/min, λ = 230 nm: τ_{Minor} = 5.5 min, τ_{Major} = 8.3 min.

Rf (5 % Et_2O/Pent) = 0.65.

[α]D^{20} = +24.3 (c = 0.49, $CHCl_3$, 91.0:9.0 *er*).

^{1}H NMR (400 MHz, $CDCl_3$) δ 7.53 (s, 4H, Ar*H*), 7.49 – 7.44 (m, 2H, Ar*H*), 7.44 – 7.38 (m, 2H, Ar*H*), 7.36 – 7.28 (m, 5H, Ar*H*), 7.26 – 7.19 (m, 1H, Ar*H*), 5.08 (q, *J* = 5.5 Hz, 1H, C*H*CF_3), 4.07 (d, *J* = 13.0 Hz, 1H, PhCH_aH_bN), 4.03 (d, *J* = 13.0 Hz, 1H, PhCH_aH_bN), 3.87 (dq, *J* = 13.7, 1.5 Hz, 1H, NCH_aH_bC), 3.19 (dq, *J* = 13.7, 1.5 Hz, 1H, NCH_aH_bC).
^{19}F NMR (376 MHz, $CDCl_3$) δ -62.8 (s, 3F, ArC*F*$_3$), –79.5 (dt, *J* = 5.5, 1.5 Hz, 3F, CHC*F*$_3$).
^{13}C{^{1}H} NMR (101 MHz, $CDCl_3$) δ 142.5, 137.5, 137.0, 129.5 (d, J_{C-F} = 32.7 Hz), 129.6, 129.4, 129.0, 128.79, 128.75, 128.2, 127.8, 125.9 (q, *J* = 3.7 Hz), 123.9 (d, *J* = 272.5 Hz), 123.0 (d, *J* = 283.5 Hz), 94.8 (q, *J* = 34.2 Hz), 74.3, 69.2, 60.0, 53.5, 45.0.
IR (cm^{-1}) 3035 (w), 2927 (w), 2850 (w), 1500 (w), 1327 (s), 1288 (m), 1173 (s), 1134 (s).

HRMS (ESI/QTOF) m/z: [M + H]$^+$ Calcd for $C_{26}H_{20}Cl_2F_6NO^+$ 546.0821; Found 546.0823.

Methyl 4-((1*S*,3*R*,5*S*)-6-benzyl-2,2-dichloro-1-phenyl-5-(trifluoromethyl)-4-oxa-6-azaspiro[2.4]heptan-1-yl)benzoate (3k)

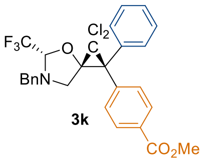

3k

Prepared according to the general procedure D1 using **2k** (91 mg, 0.20 mmol, 1.0 equiv., 90.5:9.5 *er*). The *dr* was determined to be 19:1 by the integration of the crude ^{19}F NMR spectra. The crude material was purified by column chromatography (0 – 10 % (v/v) Et_2O in pentane) to give **3k** (65 mg, 0.12 mmol, 61 % yield) as an amorphous off-white solid. The enantiomeric ratio was determined to be 90.5:9.5 by HPLC analysis on a Daicel Chiralpak IB N-5 column: 99:1 hexane/IPA, flow rate 1 mL/min, λ = 254 nm: τ_{Minor} = 6.9 min, τ_{Major} = 8.7 min.

Rf (5 % Et_2O/Pent) = 0.28.

[α]D^{20} = +38.2 (c = 0.51, $CHCl_3$, 90.5:9.5 *er*).

^{1}H NMR (400 MHz, $CDCl_3$) (400 MHz, $CDCl_3$) δ 7.98 – 7.89 (m, 2H, Ar*H*), 7.51 – 7.44 (m, 4H, Ar*H*), 7.44 – 7.38 (m, 2H, Ar*H*), 7.36 – 7.27 (m, 5H, Ar*H*), 7.25 – 7.18 (m, 1H, Ar*H*), 5.07 (q, *J* = 5.5 Hz, 1H, C*H*CF_3), 4.06 (d, *J* = 13.0 Hz, 1H, PhCH_aH_bN), 4.02 (d, *J* = 13.0 Hz, 1H, PhCH_aH_bN), 3.89 (dq, 1H, *J* = 13.7, 1.5 Hz, NCH_aH_bC), 3.86 (s, 3H, ArCOOC*H*$_3$), 3.18 (dq, *J* = 13.7, 1.6 Hz, 1H, NCH_aH_bC).
^{19}F NMR (376 MHz, $CDCl_3$) δ –79.5 (dt, *J* = 5.5, 1.5 Hz, 3F, CHC*F*$_3$).
^{13}C{^{1}H} NMR (101 MHz, $CDCl_3$) δ 166.5, 143.4, 137.5, 137.0, 130.1, 129.6, 129.3, 129.0, 129.0, 128.8, 128.7, 128.2, 127.7, 123.0 (d, *J* = 283.6 Hz), 94.7 (q, *J* = 34.3 Hz), 74.3, 69.3, 60.0, 53.5, 52.3, 45.2.
IR (cm^{-1}) 3028 (w), 2947 (w), 2850 (w), 1724 (s), 1446 (w), 1284 (s), 1184 (s), 1142 (s).

HRMS (ESI/QTOF) m/z: [M + H]$^+$ Calcd for $C_{27}H_{23}Cl_2F_3NO_3^+$ 536.1002; Found 536.1009.

(2*S*,3*R*,5*S*)-6-Benzyl-1,1-dichloro-2-(4-chlorophenyl)-2-phenyl-5-(trifluoromethyl)-4-oxa-6-azaspiro[2.4]heptane (3l)

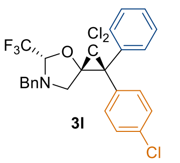

3l

Prepared according to the general procedure D1 using **2l** (86 mg, 0.20 mmol, 1.0 equiv., 90.0:10.0 *er*). The *dr* was determined to be 19:1 by the integration of the crude ^{19}F NMR spectra. The crude material was purified by column chromatography (0 – 5 % (v/v) Et_2O in pentane) to give **3l** (72 mg, 0.14 mmol, 70 % yield) as an amorphous off-white solid. The enantiomeric ratio was determined to be 90.0:10.0 by HPLC analysis on a Daicel Chiralpak IB N-5 column: 99:1 hexane/IPA, flow rate 1 mL/min, λ = 254 nm: τ_{Minor} = 5.2 min, τ_{Major} = 6.8 min.

Rf (5 % Et_2O/Pent) = 0.74.

[α]D^{20} = +31.3 (c = 0.54, $CHCl_3$, 90.0:10.0 *er*).

^{1}H NMR (400 MHz, $CDCl_3$) (400 MHz, $CDCl_3$) δ 7.47 – 7.39 (m, 4H, Ar*H*), 7.37 – 7.27 (m, 7H, Ar*H*), 7.27 – 7.18 (m, 4H, Ar*H*), 5.06 (q, *J* = 5.5 Hz, 1H, C*H*CF_3), 4.07 (d, *J* = 13.0 Hz, 1H, PhCH_aH_bN), 4.02 (d, *J* = 13.0 Hz, 1HPhCH_aH_bN), 3.86 (dq, *J* = 13.7, 1.5 Hz, 1H, NCH_aH_bC), 3.19 (dq, *J* = 13.7, 1.5 Hz, 1H, NCH_aH_bC).
^{19}F NMR (376 MHz, $CDCl_3$) δ –79.5 (dt, *J* = 5.5, 1.5 Hz, 3F, CHC*F*$_3$).

$^{13}C\{^1H\}$ NMR (101 MHz, $CDCl_3$) δ 137.9, 137.2, 137.0, 133.7, 130.3, 129.5, 129.1, 129.0, 128.8, 128.7, 128.2, 127.6, 123.0 (d, J = 283.7 Hz), 94.7 (q, J = 34.3 Hz), 74.3, 69.4, 60.0, 53.5, 44.7.
IR (cm^{-1}) 3032 (w), 2927 (w), 2850 (w), 1493 (m), 1288 (m), 1180 (s), 1142 (s).

HRMS (ESI/QTOF) m/z: $[M + H]^+$ Calcd for $C_{25}H_{20}Cl_3F_3NO^+$ 512.0557; Found 512.0562.

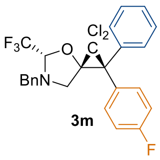

3m

(2*S*,3*R*,5*S*)-6-Benzyl-1,1-dichloro-2-(4-fluorophenyl)-2-phenyl-5-(trifluoromethyl)-4-oxa-6-azaspiro[2.4]heptane (3m)

Prepared according to the general procedure D1 using **2m** (83 mg, 0.20 mmol, 1.0 equiv., 90.0:10.0 *er*). The *dr* was determined to be 18:1 by the integration of the crude ^{19}F NMR spectra. The crude material was purified by column chromatography (0 – 5 % (v/v) Et_2O in pentane) to give **3m** (70 mg, 0.14 mmol, 71 % yield) as an amorphous off-white solid. The enantiomeric ratio was determined to be 90.0:10.0 by HPLC analysis on a Daicel Chiralpak IB N-5 column: 99:1 hexane/IPA, flow rate 1 mL/min, λ = 230 nm: τ_{Minor} = 5.7 min, τ_{Major} = 8.2 min.

Rf (5 % Et_2O/Pent) = 0.65.

$[\alpha]D^{20}$ = +19.0 (c = 0.51, $CHCl_3$, 90.0:10.0 *er*).

^1H NMR (400 MHz, $CDCl_3$) δ 7.48 – 7.40 (m, 4H, Ar*H*), 7.40 – 7.27 (m, 7H, Ar*H*), 7.25 – 7.17 (m, 1H, Ar*H*), 6.96 (t, J = 8.6 Hz, 2H, Ar*H*), 5.06 (q, J = 5.6 Hz, 1H, C*H*CF3), 4.07 (d, J = 13.0 Hz, 1H, PhCH_aH$_b$N), 4.02 (d, J = 13.0 Hz, 1H, PhCH$_a$$H_b$N), 3.88 (dq, J = 13.7, 1.5 Hz, 1H, NCH_aH$_b$C), 3.19 (dq, J = 13.7, 1.5 Hz, 1H, NCH$_a$$H_b$C).
^{19}F NMR (376 MHz, $CDCl_3$) δ -79.5 (dt, J = 5.6, 1.5 Hz, 3F, CHCF_3), -114.1 (ddd, J = 13.8, 8.6, 5.2 Hz, 1F, Ar*F*).
$^{13}C\{^1H\}$ NMR (101 MHz, $CDCl_3$) δ 162.0 (d, J = 247.7 Hz), 138.2, 137.1, 134.6 (d, J = 3.3 Hz), 130.7 (d, J = 8.1 Hz), 129.5, 129.0, 128.8, 128.6, 128.1, 127.5, 123.0 (d, J = 283.5 Hz), 115.9 (d, J = 21.7 Hz), 94.7 (q, J = 34.3 Hz), 74.3, 69.5, 60.0, 53.6, 44.6.
IR (cm^{-1}) 3032 (m), 2927 (m), 2854 (w), 1604 (m), 1508 (m), 1288 (m), 1227 (s), 1180 (s), 1142 (s).

HRMS (ESI/QTOF) m/z: $[M + H]^+$ Calcd for $C_{25}H_{20}Cl_2F_4NO^+$ 496.0853; Found 496.0859.

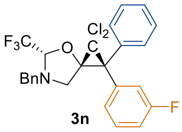

3n

(2*S*,3*R*,5*S*)-6-benzyl-1,1-dichloro-2-(3-fluorophenyl)-2-phenyl-5-(trifluoromethyl)-4-oxa-6-azaspiro[2.4]heptane (3n)

Prepared according to the general procedure D1 using **2n** (83 mg, 0.20 mmol, 1.0 equiv., 89.5:10.5 *er*). The *dr* was determined to be 16:1 by the integration of the crude ^{19}F NMR spectra. The crude material was purified by column chromatography (0 – 5 % (v/v) Et_2O in pentane) to give **3n** (73 mg, 0.15 mmol, 74 % yield) as an amorphous off-white solid. The enantiomeric ratio was determined to be 89.5:10.5 by HPLC analysis on a Daicel Chiralpak IB N-5 column: 99:1 hexane/IPA, flow rate 1 mL/min, λ = 230 nm: τ_{Minor} = 6.6 min, τ_{Major} = 8.9 min.

Rf (5 % Et_2O/Pent) = 0.62.

$[\alpha]D^{20}$ = +18.9 (c = 0.36, $CHCl_3$, 89.5:10.5 *er*).

^1H NMR (400 MHz, $CDCl_3$) δ 7.50 – 7.38 (m, 4H, Ar*H*), 7.37 – 7.27 (m, 6H, Ar*H*), 7.25 – 7.19 (m, 2H, Ar*H*), 7.19 – 7.09 (m, 2H, Ar*H*), 5.06 (q, J = 5.6 Hz, 1H, C*H*CF3), 4.07 (d, J = 13.0 Hz, 1H, PhCH_aH$_b$N), 4.03 (d, J = 13.0 Hz, 1H, PhCH$_a$$H_b$N), 3.90 (dq, J = 13.7, 1.5 Hz, 1H, NCH_aH$_b$C), 3.21 (dq, J = 13.7, 1.5 Hz, 1H, NCH$_a$$H_b$C).
^{19}F NMR (376 MHz, $CDCl_3$) δ -79.5 (dt, J = 5.6, 1.5 Hz, 3F, CHCF_3), -111.3 (td, J = 9.0, 5.6 Hz, 1F, Ar*F*).

$^{13}C\{^1H\}$ NMR (101 MHz, $CDCl_3$) δ 162.6 (d, J = 247.3 Hz), 140.8 (d, J = 7.3 Hz), 137.8, 137.1, 130.4 (d, J = 8.3 Hz), 129.6, 129.0, 128.8, 128.6, 128.1, 127.6, 124.7 (d, J = 3.1 Hz), 123.0 (d, J = 283.5 Hz), 116.1 (d, J = 22.4 Hz), 114.7 (d, J = 20.9 Hz), 94.7 (q, J = 34.2 Hz), 74.3, 69.3, 60.0, 53.5, 44.9.
IR (cm^{-1}) 3028 (w), 2924 (w), 2850 (w), 1500 (m), 1450 (w), 1323 (m), 1288 (m), 1176 (s), 1138 (s).

HRMS (ESI/QTOF) m/z: $[M + H]^+$ Calcd for $C_{25}H_{20}Cl_3F_3NO^+$ 512.0557; Found 512.0562.

D.3. General Procedure for the Asymmetric Epoxidation/Solvolysis of the Tetrasubstituted Olefins

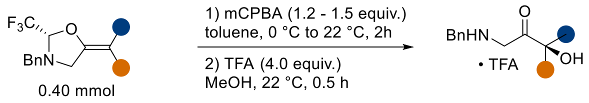

Scheme 14. Asymmetric epoxidation of tetrasubstituted olefin using mCPBA.

Epoxidation: A 25 mL round bottom flask equipped with a Teflon coated stirring bar was charged with the tetrasubstituted olefin (0.40 mmol) and toluene (4.0 mL). The flask was cooled to 0 °C. Then, a solution of mCPBA (108 mg, 0.440 mmol, 1.2 equiv.) in toluene (4.0 mL, dried over anhydrous Na_2SO_4) was added drop-wise over 10 minutes. The mixture was stirred at 0 °C for 1 hour, then warmed to 22 °C and stirred for an additional 1 h. Then, sat. aq. Na_2SO_3 (2.0 mL) was added, and the mixture stirred for 10 minutes at 22 °C. The organic layer was separated and washed with sat. aq. $NaHCO_3$ (3 × 5.0 mL), dried over Na_2SO_4, filtered and concentrated *in vacuo*. The crude mixture was analyzed with ^{1}H NMR using trichloroethylene (36 µL, 0.40 mmol, 1.0 equiv.) as the internal standard.

Hydrolysis: A 50 mL round bottom flask was charged with the crude from the previous step, MeOH (4.0 mL) and TFA (122 µL, 1.60 mmol, 4.0 equiv.). The resulting solution was stirred at 22 °C for 30 minutes. Then the reaction mixture was concentrated *in vacuo*.

Purification: The resulting crude mixture was dissolved in DCM (2.0 mL) and pentane (20 mL) was added to induce precipitation. The resulting slurry was concentrated in vacuo. The remaining solid was triturated with pentane (5 × 5.0 mL).

If the precipitation strategy fails, it is also possible to purify the corresponding compounds *via* flash column chromatography. To do so, the product was dissolved in a small amount of DCM (1 – 2 mL) and washed with 1 M NaOH (3 × 1.0 mL). **NB: it is imperative to avoid concentrating the unstable freebase form of the aminoketone!** The organic layer was then directly loaded onto the silica column and eluted with MeOH in DCM (0 – 2 %). Fractions containing the corresponding product were pooled and TFA (31 µL, 0.10 mmol, 1.0 equiv. assuming 100 % yield) was added. The resulting solutions was then concentrated *in vacuo*. The absolute configuration was assigned based on the relative and absolute configuration of the starting materials.

D.4. Characterization of the oxidized products

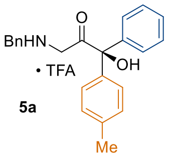

(*S*)-3-(Benzylamino)-1-hydroxy-1-phenyl-1-(p-tolyl)propan-2-one (5a)

Prepared according to the general procedure D3 using **2a** (164 mg, 0.400 mmol, 1.0 equiv., 97.0:3.0 *er*). The crude material was purified by precipitation/trituration to give the corresponding product **5a** (147 mg, 0.320 mmol, 80 % yield) as an amorphous white solid. The enantiomeric ratio was determined to be 96.0:4.0 by HPLC analysis on a Daicel Chiralpak IB N-5 column: 95:5 hexane/IPA, flow rate 1 mL/min, λ = 210 nm: τ_{Minor} = 17.2 min, τ_{Major} = 18.5 min.

Rf (1:2:98 ratio of Et_3N/MeOH/DCM) = 0.20.

$[\alpha]D^{20}$ = -2.2 (c = 0.55, MeOH, 96.0:4.0 *er*).

^{1}H NMR (400 MHz, CD_3OD) δ 7.48 – 7.39 (m, 3H, Ar*H*), 7.39 – 7.28 (m, 7H, Ar*H*), 7.24 (d, *J* = 8.3 Hz, 2H, Ar*H*), 7.18 (d, *J* = 8.3 Hz, 2H, Ar*H*), 4.44 (s, 2H, CH_2C=O), 4.15 (s, 2H, PhCH_2N), 2.34 (s, 3H, ArCH_3).

^{13}C{^{1}H} NMR (101 MHz, CD_3OD) δ 206.2, 163.15 (q, $J_{C\text{-}F}$ = 34.1 Hz), 143.0, 139.9, 139.4, 132.0, 131.1, 130.8, 130.3, 129.9, 129.29, 129.27, 128.8, 128.7, 118.3 (q, $J_{C\text{-}F}$ = 292.9 Hz), 86.1, 53.2, 51.7, 21.1.

^{19}F NMR (376 MHz, CD_3OD) δ –76.9 (s, 3H, CF_3COOH).

IR (cm^{-1}) 3394 (s), 2827 (w), 1674 (s), 1446 (w), 1423 (w), 1327 (w), 1196 (s), 1142 (s)

HRMS (ESI/QTOF) m/z: $[M + H]^+$ Calcd for $C_{23}H_{24}NO_2^+$ 346.1802; Found 346.1799.

2 mmol scale: A 100 mL round bottom flask equipped with a Teflon coated stirring bar was charged with the tetrasubstituted olefin **2a** (819 mg, 2.00 mmol, 1.0 equiv., 99.0:1.0 *er*) and toluene (20 mL). The flask was cooled to 0 °C. Then, using an addition funnel with a pressure equalizing arm, a solution of *m*CPBA (538 mg, 2.40 mmol, 1.2 equiv.) in toluene (20 mL, dried over anhydrous Na_2SO_4) was added drop-wise over 20 minutes. The mixture was stirred at 0 °C for 1 hour, then warmed to 22 °C and stirred for an additional 1 h. Then, sat. aq. Na_2SO_3 (20 mL) was added, and the mixture stirred for 10 minutes at 22 °C. The organic layer was separated and washed with sat. aq. $NaHCO_3$ (3 × 20 mL), dried over Na_2SO_4, filtered

and concentrated *in vacuo*. The crude mixture was analyzed with ^{1}H NMR using trichloroethylene (36 µL, 0.40 mmol, 0.25 equiv.) as the internal standard.

Purification: The resulting crude mixture was dissolved in DCM (2.0 mL) and pentane (100 mL) was added to induce precipitation. The resulting slurry was concentrated in vacuo. The remaining solid was triturated with pentane (5 × 20 mL) to give the corresponding product **5a** (735 mg, 1.60 mmol, 80 % yield). The enantiomeric ratio was determined to be 97.5:2.5 by HPLC analysis on a Daicel Chiralpak IB N-5 column: 95:5 hexane/IPA, flow rate 1 mL/min, λ = 210 nm: τ_{Minor} = 17.2 min, τ_{Major} = 18.5 min. $[\alpha]D^{20}$ = -3.2 (c = 0.59, MeOH, 97.5:2.5 *er*).

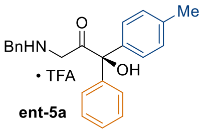

(*R*)-3-(Benzylamino)-1-hydroxy-1-phenyl-1-(p-tolyl)propan-2-one (ent-5a)

Prepared according to the general procedure D3 using **2b** (164 mg, 0.400 mmol, 1.0 equiv., 94.5:5.5 *er*). The crude material was purified by precipitation/trituration to give the corresponding product **ent-5a** (141 mg, 0.307 mmol, 77 % yield) as an amorphous white solid. The enantiomeric ratio was determined to be 93.0:7.0 by HPLC analysis on a Daicel Chiralpak IB N-5 column: 95:5 hexane/IPA, flow rate 1 mL/min λ = 210 nm: τ_{Major} = 17.2 min, τ_{Minor} = 18.5 min.

Rf (1:2:98 ratio of Et_3N/MeOH/DCM) = 0.20.

$[\alpha]D^{20}$ = +3.6 (c = 0.53, MeOH, 93.0:7.0 *er*).

^{1}H NMR (400 MHz, CD_3OD) δ 7.48 – 7.39 (m, 3H, Ar*H*), 7.38 – 7.28 (m, 7H, Ar*H*), 7.24 (d, *J* = 8.3 Hz, 2H, Ar*H*), 7.18 (d, *J* = 8.3 Hz, 2H, Ar*H*), 4.44 (s, 2H, CH_2C=O), 4.15 (s, 2H, PhCH_2N), 2.34 (s, 3H, ArCH_3).

^{13}C{^{1}H} NMR (101 MHz, CD_3OD) δ 206.2, 163.1 (q, J_{C-F} = 34.1 Hz), 143.0, 139.9, 139.4, 132.0, 131.1, 130.8, 130.3, 129.9, 129.29, 129.27, 128.8, 128.7, 118.3 (q, J_{C-F} = 292.9 Hz), 86.1, 53.2, 51.7, 21.1.

^{19}F NMR (376 MHz, CD_3OD) δ –76.9 (s, 3H, CF_3COOH).

IR (cm^{-1}) 3394 (s), 2989 (w), 2831 (w), 1674 (s), 1415 (w), 1196 (s), 1138 (m).

HRMS (ESI/QTOF) m/z: $[M + H]^+$ Calcd for $C_{23}H_{24}NO_2^+$ 346.1802; Found 346.1803.

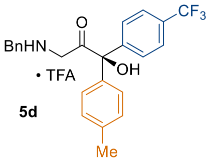

(*R*)-3-(Benzylamino)-1-hydroxy-1-(p-tolyl)-1-(4-(trifluoromethyl)phenyl)propan-2-one (5d)

Prepared according to the modified general procedure D3 using **2c** (191 mg, 0.400 mmol, 1.0 equiv., 94.0:6.0 *er*) and mCPBA (135 mg, 0.600 mmol, 1.5 equiv.). The crude material was purified by precipitation/trituration to give the corresponding product **5c** (127 mg, 0.241 mmol, 60 % yield) as an amorphous off-white solid. The enantiomeric ratio was determined to be 93.5:6.5 by HPLC analysis on a Daicel Chiralpak IB N-5 column: 95:5 hexane/IPA, flow rate 1 mL/min, λ = 230 nm: τ_{Minor} = 13.0 min, τ_{Major} = 19.0 min.

Rf (1:2:98 ratio of Et_3N/MeOH/DCM) = 0.24.

$[\alpha]D^{20}$ = -17.1 (c = 0.53, MeOH, 93.5:6.5 *er*).

^{1}H NMR (400 MHz, CD_3OD) δ 7.69 – 7.62 (m, 2H, Ar*H*), 7.62 – 7.54 (m, 2H, Ar*H*), 7.47 – 7.36 (m, 5H, Ar*H*), 7.27 – 7.18 (m, 4H, Ar*H*), 4.52 (d, *J* = 18.9 Hz, 1H, CH_aH$_b$C=O), 4.44 (d, *J* = 18.9 Hz, 1H, CH$_a$$H_b$C=O), 4.20 (d, *J* = 13.2 Hz, 1H, PhCH_aH$_b$N), 4.16 (d, *J* = 13.2 Hz, 2H, PhCH$_a$$H_b$N), 2.35 (s, 3H, ArC$H_3$).

^{13}C{^{1}H} NMR (101 MHz, CD_3OD) δ 205.8, 163.0 (q, J_{C-F} = 35.6 Hz), 147.4, 139.8, 139.2, 132.0, 131.4 131.1, 130.9, 130.3, 130.2, 129.6, 128.5, 126.1 (q, J_{C-F} = 3.8 Hz), 125.6 (q, J_{C-F} = 271.4 Hz), 118.2 (d, *J* = 294.3 Hz), 85.7, 53.3, 51.7, 21.1.

^{19}F NMR (376 MHz, CD_3OD) δ -64.1 (s, 3F, ArCF_3), -76.9 (s, 3F, CF_3COOH).

IR (cm^{-1}) 3402 (br.), 2947 (w), 2831 (w), 1674 (s), 1415 (w), 1327 (s), 1173 (s), 1138 (s).

HRMS (ESI/QTOF) m/z: $[M + H]^+$ Calcd for $C_{24}H_{23}F_3NO_2^+$ 414.1675; Found 414.1677.

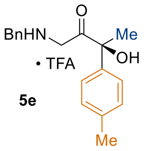

(*S*)-1-(Benzylamino)-3-hydroxy-3-(p-tolyl)butan-2-one (5e)

Prepared according to the general procedure D3 using **2e** (139 mg, 0.400 mmol, 1.0 equiv., 85.5:14.5 *er*). The crude material was purified by column chromatography to give the corresponding product **5e** (87 mg, 0.22 mmol, 55 % yield) as a brown oil. The enantiomeric ratio was determined to be 80.0:20.0 by HPLC analysis on a Daicel Chiralpak IB N-5 column: 95:5 hexane/IPA, flow rate 1 mL/min, λ = 210 nm: τ_{Minor} = 17.7 min, τ_{Major} = 20.3 min.

Rf (1:2:98 ratio of Et_3N/MeOH/DCM) = 0.20.

$[\alpha]D^{20}$ = -32.3 (c = 0.50, MeOH, 80.0:20.0 *er*).

Underlined <u>1H NMR</u> labels rendered as plain text.

1H NMR (400 MHz, CD_3OD) δ 7.33 – 7.26 (m, 5H, Ar*H*), 7.25 – 7.20 (m, 2H, Ar*H*), 7.12 – 7.07 (m, 2H, Ar*H*), 4.30 (d, *J* = 18.5 Hz, 1H, C$H_a$$H_b$C=O), 4.04 – 3.92 (m, 3H, C$H_a$$H_b$C=O and PhC$H_2$N), 2.22 (s, 3H, ArC$H_3$), 1.58 (s, 3H, C$H_3$).

$^{13}C\{^1H\}$ NMR δ 206.2, 162.5 (q, *J* = 35.8 Hz), 140.0, 139.1, 131.9, 131.0, 130.8, 130.34, 130.30, 126.3, 117.8 (d, *J* = 283.3 Hz), 80.8, 51.6, 51.4, 26.5, 21.0.

^{19}F NMR (376 MHz, CD_3OD) δ -77.1 (s, 3F, CF_3COOH).

IR (cm^{-1}) 3498 (br. s), 2993 (w), 2954 (w), 1724 (m), 1678 (s), 1512 (w), 1423 (w), 1200 (s), 1142 (s).

HRMS (ESI/QTOF) m/z: $[M + H]^+$ Calcd for $C_{18}H_{22}NO_2^+$ 284.1645; Found 284.1651.

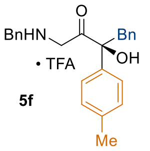

(*S*)-1-(Benzylamino)-3-hydroxy-4-phenyl-3-(p-tolyl)butan-2-one (5f)

Prepared according to the general procedure D3 using **2f** (164 mg, 0.400 mmol, 1.0 equiv., 92.0:8.0 *er*). The crude material was purified by precipitation/trituration to give the corresponding product **5f** (134 mg, 0.292 mmol, 73 % yield) as an amorphous off-white solid. The enantiomeric ratio was determined to be 91.0:9.0 by HPLC analysis on a Daicel Chiralpak IB N-5 column: 95:5 hexane/IPA, flow rate 1 mL/min, λ = 230 nm: τ_{Minor} = 17.7 min, τ_{Major} = 21.4 min.

Rf (1:2:98 ratio of Et_3N/MeOH/DCM) = 0.16.

$[\alpha]D^{20}$ = -39.7 (c = 0.60, MeOH, 91.0:9.0 *er*).

1H NMR (400 MHz, CD_3OD) δ 7.51 – 7.34 (m, 5H, Ar*H*), 7.29 – 7.22 (m, 2H, Ar*H*), 7.22 – 7.09 (m, 7H, Ar*H*), 4.09 – 3.93 (m, 4H, CH_2C=O and CH_2Ph), 3.58 (d, *J* = 14.0 Hz, 1H, PhC$H_a$$H_b$N), 3.22 (d, *J* = 14.0 Hz, 1H, PhC$H_a$$H_b$N), 2.33 (s, 3H, ArC$H_3$).

^{19}F NMR (376 MHz, CD_3OD) δ -77.0 (s, 3F, CF_3COOH).

$^{13}C\{^1H\}$ NMR (101 MHz, CD_3OD) δ 206.2, 162.2 (q, *J* = 35.6 Hz), 139.2, 138.6, 137.0, 132.0, 131.8, 131.0, 130.8, 130.3, 130.2, 128.9, 127.7, 126.8, 117.9 (d, *J* = 295.2 Hz), 84.2, 52.4, 51.5, 46.0, 21.0.

IR (cm^{-1}) 3614 (br.), 2989 (w), 1674 (s), 1508 (w), 1419 (m), 1196 (s), 1138 (s).

HRMS (ESI/QTOF) m/z: $[M + H]^+$ Calcd for $C_{24}H_{26}NO_2^+$ 360.1958; Found 360.1952.

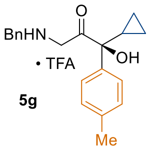

(*S*)-3-(Benzylamino)-1-cyclopropyl-1-hydroxy-1-(p-tolyl)propan-2-one (5g)

Prepared according to the general procedure D3 using **2g** (149 mg, 0.400 mmol, 1.0 equiv., 88.0:12.0 *er*). The crude material was purified by precipitation/trituration to give the corresponding product **5g** (106 mg, 0.250 mmol, 63 % yield) as an amorphous pale-yellow solid. The enantiomeric ratio was determined to be 88.0:12.0 by HPLC analysis on a Daicel Chiralpak IB N-5 column: 95:5 hexane/IPA, flow rate 1 mL/min, λ = 230 nm: τ_{Minor} = 13.0 min, τ_{Major} = 19.0 min.

Rf (1:2:98 ratio of Et_3N/MeOH/DCM) = 0.20.

$[\alpha]D^{20}$ = -2.4 (c = 0.50, MeOH, 88.0:12.0 *er*).

1H NMR (400 MHz, CD_3OD) δ 7.48 – 7.36 (m, 7H, Ar*H*), 7.20 (d, *J* = 8.0 Hz, 2H, Ar*H*), 4.29 (s, 2H, CH_2C=O), 4.14 (s, 2H, PhCH_2N), 2.33 (s, 3H, ArCH_3), 1.70 (ddd, *J* = 8.0, 6.1, 2.4 Hz, 1H, C*H*(CH_2CH_2)), 0.61 – 0.51 (m, 2H, CH(C$H_2$$CH_2$)), 0.51 – 0.36 (m, 2H, CH(CH_2CH_2)).

$^{13}C\{^1H\}$ NMR (101 MHz, CD_3OD) δ 206.6, 162.0 (q, *J* = 35.9 Hz), 139.3, 132.1, 131.0, 130.8, 130.3, 130.2, 130.0, 127.4, 117.8 (q, *J* = 293.2 Hz), 81.7, 52.7, 51.7, 21.1, 18.5, 1.1, 0.9.

^{19}F NMR (376 MHz, CD_3OD) δ -76.9 (s, 3F, CF_3COOH).

IR (cm^{-1}) 3614 (br.), 3008 (w), 2951 (w), 1724 (m), 1678 (s), 1516 (m), 1419 (m), 1200 (s), 1142 (s).

HRMS (ESI/QTOF) m/z: $[M + H]^+$ Calcd for $C_{20}H_{24}NO_2^+$ 310.1802; Found 310.1799.

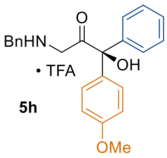

(*S*)-3-(Benzylamino)-1-hydroxy-1-(4-methoxyphenyl)-1-phenylpropan-2-one (5h)

Prepared according to the general procedure D3 using **2h** (170 mg, 0.400 mmol, 1.0 equiv., 95.0:5.0 *er*). The crude material was purified by precipitation/trituration to give the corresponding product **5h** (85 mg, 0.18 mmol, 45 % yield) as an amorphous yellow solid. The enantiomeric ratio was determined to be 94.0:6.0 by HPLC analysis on a Daicel Chiralpak IB N-5 column: 80:20 hexane/IPA, flow rate 1 mL/min, λ = 210 nm: τ_{Major} = 9.8 min, τ_{Minor} = 10.9 min.

Rf (1:2:98 ratio of Et_3N/MeOH/DCM) = 0.20.

$[\alpha]D^{20}$ = -1.6 (c = 0.47, MeOH, 94.0:6.0 *er*).

^{1}H NMR (400 MHz, CD_3OD) δ 7.44 – 7.40 (m, 3H, Ar*H*), 7.39 – 7.32 (m, 7H, Ar*H*), 7.27 (d, *J* = 8.9 Hz, 2H, Ar*H*), 6.91 (d, *J* = 8.9 Hz, 2H, Ar*H*), 4.51 – 4.36 (m, 2H, CH_2C=O), 4.15 (s, 2H, PhCH_2N), 3.80 (s, 3H, ArOCH_3).

^{19}F NMR (376 MHz, CD_3OD) δ -77.3 (s, 3F, CF_3COOH).

^{13}C{^{1}H} NMR (101 MHz, CD_3OD) δ 206.2, 161.6 (q, *J* = 36.7 Hz), 161.1, 143.1, 134.8, 132.0, 131.1, 130.8, 130.3, 130.1, 129.29, 129.28, 128.8, 117.5 (q, *J* = 288.7 Hz), 114.6, 85.9, 55.8, 53.2, 51.7.

IR (cm^{-1}) 3622 (br.), 2997 (w), 2954 (w), 1678 (s), 1512 (m), 1254 (m), 1196 (s), 1142 (s)

HRMS (ESI/QTOF) m/z: $[M + H]^+$ Calcd for $C_{23}H_{24}NO_3^+$ 362.1751; Found 362.1756.

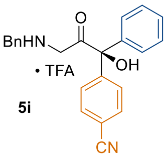

(*S*)-4-(3-(Benzylamino)-1-hydroxy-2-oxo-1-phenylpropyl)benzonitrile (5i)

Prepared according to the modified general procedure D3 using **2i** (148 mg, 0.400 mmol, 1.0 equiv., 86.0:14.0 *er*) and mCPBA (135 mg, 0.600 mmol, 1.5 equiv.). The crude material was purified by precipitation/trituration to give the corresponding product **5i** (140 mg, 0.300 mmol, 74 % yield) as an amorphous off-white solid. The enantiomeric ratio was determined to be 82.5:17.5 by HPLC analysis on a Daicel Chiralpak IB N-5 column: 95:5 hexane/IPA, flow rate 1 mL/min, λ = 210 nm: τ_{Major} = 9.4 min, τ_{Minor} = 11.2 min.

Rf (1:2:98 ratio of Et_3N/MeOH/DCM) = 0.20.

$[\alpha]D^{20}$ = +13.4 (c = 1.25, MeOH, 82.5:17.5 *er*).

^{1}H NMR (400 MHz, CD_3OD) δ 7.75 – 7.70 (m, 2H, Ar*H*), 7.60 – 7.55 (m, 2H, Ar*H*), 7.46 – 7.33 (m, 10H, Ar*H*), 4.54 (d, *J* = 18.9 Hz, 1H, CH_aH_bC=O), 4.44 (d, *J* = 18.9 Hz, 1H, CH_aH_bC=O), 4.22 (d, *J* = 13.1 Hz, 1H, PhCH_aH_bN), 4.17 (d, *J* = 13.1 Hz, 1H, PhCH_aH_bN).
^{19}F NMR (376 MHz, CD_3OD) δ -77.1 (s, 3F, CF_3COOH).

^{13}C{^{1}H} NMR (101 MHz, CD_3OD) δ 205.7, 162.7 (q, *J* = 33.4 Hz), 148.3, 141.9, 133.1, 132.0, 131.1, 130.9, 130.4, 129.84, 129.82, 129.7, 128.4, 119.4, 118.3 (q, *J* = 292.0 Hz) 113.2, 85.7, 53.4, 51.8.

IR (cm^{-1}) 3402 (br.), 2989 (w), 2233 (w), 1732 (m), 1674 (s), 1412 (w), 1196 (s), 1142 (s).

HRMS (ESI/QTOF) m/z: $[M + H]^+$ Calcd for $C_{23}H_{21}N_2O_2^+$ 357.1598; Found 357.1583.

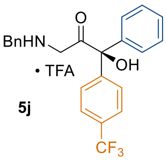

(*S*)-3-(Benzylamino)-1-hydroxy-1-phenyl-1-(4-(trifluoromethyl)phenyl)propan-2-one (5j)

Prepared according to the modified general procedure D5 using **2j** (185 mg, 0.400 mmol, 1.0 equiv., 91.0:9.0 *er*) and mCPBA (135 mg, 0.600 mmol, 1.5 equiv.). The crude material was purified by precipitation/trituration to give the corresponding product **5j** (155 mg, 0.302 mmol, 75 % yield) as an amorphous off-white solid. The enantiomeric ratio was determined to be 91.0:9.0 by HPLC analysis on a Daicel Chiralpak IB N-5 column: 95:5 hexane/IPA, flow rate 1 mL/min, λ = 210 nm: τ_{Major} = 9.4 min, τ_{Minor} = 11.2 min.

Rf (1:2:98 ratio of Et_3N/MeOH/DCM) = 0.24.

$[\alpha]D^{20}$ = +12.5 (c = 0.53, MeOH, 91.0:9.0 *er*).

^{1}H NMR (400 MHz, CD_3OD) δ 7.75 – 7.70 (m, 2H, Ar*H*), 7.60 – 7.55 (m, 2H, Ar*H*), 7.46 – 7.33 (m, 10H, Ar*H*), 4.54 (d, *J* = 18.9 Hz, 1H, CH_aH_bC=O), 4.44 (d, *J* = 18.9 Hz, 1H, CH_aH_bC=O), 4.22 (d, *J* = 13.1 Hz, 1H, PhCH_aH_bN), 4.17 (d, *J* = 13.1 Hz, 1H, PhCH_aH_bN).
^{19}F NMR (376 MHz, CD_3OD) δ -64.2 (s, 3F, ArCF_3), -76.9 (s, 3F, CF_3COOH).
^{13}C{^{1}H} NMR (101 MHz, CD_3OD) δ 205.8, 163.0 (d, *J* = 34.5 Hz), 147.3, 142.2, 132.0, 131.4 (q, *J* = 32.4 Hz), 131.1, 130.9, 130.4, 129.7, 129.6, 129.6, 128.5, 126.1 (d, *J* = 4.1 Hz), 125.5 (q, *J* = 271.3 Hz), 118.2 (q, *J* = 293.4 Hz), 85.8, 53.4, 51.7.

IR (cm^{-1}) 3394 (br.), 2993 (w), 2827 (w), 1674 (s), 1415 (w), 1327 (s), 1196 (s), 1134 (s).

HRMS (ESI/QTOF) m/z: $[M + H]^+$ Calcd for $C_{23}H_{21}F_3NO_2^+$ 400.1519; Found 400.1521.

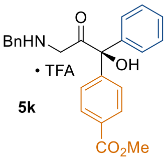

Methyl (*S*)-4-(3-(benzylamino)-1-hydroxy-2-oxo-1-phenylpropyl)benzoate (5k)

Prepared according to the modified general procedure D3 using **2k** (181 mg, 0.400 mmol, 1.0 equiv., 90.5:9.5 *er*) and mCPBA (135 mg, 0.600 mmol, 1.5 equiv.). The crude material was purified by precipitation/trituration to give the corresponding product **5k** (169 mg, 0.333 mmol, 83 % yield) as an amorphous off-white solid. The enantiomeric ratio was determined to be 88.5:11.5 by HPLC analysis on a Daicel Chiralpak IB N-5 column: 80:20 hexane/IPA, flow rate 1 mL/min, λ = 230 nm: τ_{Major} = 8.9 min, τ_{Minor} = 14.8 min.

Rf (1:2:98 ratio of Et_3N/MeOH/DCM) = 0.20.

$[\alpha]D^{20}$ = +7.2 (c = 1.55, MeOH, 88.5:11.5 *er*).

^{1}H NMR (400 MHz, CD_3OD) δ 8.04 – 7.97 (m, 2H, Ar*H*), 7.53 – 7.48 (m, 2H, Ar*H*), 7.45 – 7.37 (m, 10H, Ar*H*), 4.52 (d, *J* = 18.9 Hz, 1H, CH_aH_bC=O), 4.45 (d, *J* = 18.9 Hz, 1H, CH_aH_bC=O), 4.18 (s, 2H, PhCH_2N), 3.90 (s, 3H, COOCH_3).
^{19}F NMR (376 MHz, CD_3OD) δ -77.1 (s, 3F, CF_3COOH).
^{13}C{^{1}H} NMR (101 MHz, CD_3OD) δ 205.8, 168.1, 162.4 (q, *J* = 36.0 Hz), 148.0, 142.3, 132.0, 131.2, 131.1, 130.9, 130.4 (2C), 129.63, 129.57, 129.0, 128.6, 117.9 (q, *J* = 291.3 Hz), 85.9, 53.4, 52.7, 51.7.

IR (cm^{-1}) 3398 (br.), 2958 (w), 1678 (s), 1439 (m), 1284 (s), 1196 (s), 1138 (s).

HRMS (ESI/QTOF) m/z: $[M + H]^+$ Calcd for $C_{24}H_{24}NO_4^+$ 390.1700; Found 390.1699.

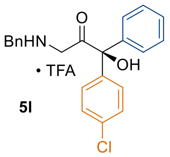

5l

(*S*)-3-(Benzylamino)-1-(4-chlorophenyl)-1-hydroxy-1-phenylpropan-2-one (5l)

Prepared according to the modified general procedure D3 using **2l** (172 mg, 0.400 mmol, 1.0 equiv., 90.0:10.0 *er*) and mCPBA (135 mg, 0.600 mmol, 1.5 equiv.). The crude material was purified by precipitation/trituration to give the corresponding product **5l** (140 mg, 0.292 mmol, 73 % yield) as an amorphous off-white solid. The enantiomeric ratio was determined to be 89.0:11.0 by HPLC analysis on a Daicel Chiralpak IB N-5 column: 95:5 hexane/IPA, flow rate 1 mL/min, λ = 230 nm: τ_{Major} = 15.3 min, τ_{Minor} = 20.6 min. A single crystal X-ray analysis confirmed the absolute configuration with 85 % probability (see section E).

Rf (1:2:98 ratio of Et_3N/MeOH/DCM) = 0.24.

$[\alpha]D^{20}$ = +95.0 (c = 0.50, MeOH, 89.0:11.0 *er*).

^{1}H NMR (400 MHz, CD_3OD) δ 7.46 – 7.41 (m, 3H, Ar*H*), 7.40 – 7.34 (m, 11H, Ar*H*), 4.49 (d, *J* = 18.9 Hz, 1H, CH_aH_bC=O), 4.42 (d, *J* = 18.9 Hz, 1H, CH_aH_bC=O), 4.19 – 4.14 (m, 2H, PhCH_2N).
^{19}F NMR (376 MHz, CD_3OD) δ -76.9 (s, 3F, CF_3COOH).
^{13}C{^{1}H} NMR (101 MHz, CD_3OD) δ 206.0, 163.1 (q, *J* = 34.1 Hz), 142.4, 141.8, 135.3, 132.0, 131.1, 130.8, 130.5, 130.3, 129.6, 129.5, 129.4, 128.6, 118.3 (q, *J* = 293.3 Hz), 85.7, 53.3, 51.7.

IR (cm^{-1}) 3394 (br.), 2943 (w), 2827 (w), 1682 (s), 1531 (m), 1423 (m), 1200 (s), 1142 (s), 1022 (s).

HRMS (ESI/QTOF) m/z: $[M + H]^+$ Calcd for $C_{22}H_{21}ClNO_2^+$ 366.1255; Found 366.1255.

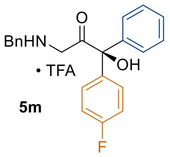

5m

(*S*)-3-(Benzylamino)-1-(4-fluorophenyl)-1-hydroxy-1-phenylpropan-2-one (5m)

Prepared according to the general procedure D3 using **2m** (165 mg, 0.400 mmol, 1.0 equiv., 90.0:10.0 *er*). The crude material was purified by precipitation/trituration to give the corresponding product **5m** (131 mg, 0.283 mmol, 71 % yield) as an amorphous off-white solid. The enantiomeric ratio was determined to be 88.5:11.5 by HPLC analysis on a Daicel Chiralpak IB N-5 column: 95:5 hexane/IPA, flow rate 1 mL/min, λ = 230 nm: τ_{Major} = 15.1 min, τ_{Minor} = 17.6 min.

Rf (1:2:98 ratio of Et_3N/MeOH/DCM) = 0.20.

$[\alpha]D^{20}$ = +9.6 (c = 0.55, MeOH, 88.5:11.5 *er*).

^{1}H NMR (400 MHz, CD_3OD) δ 7.46 – 7.41 (m, 3H, Ar*H*), 7.41 – 7.33 (m, 9H, Ar*H*), 7.09 (t, *J* = 8.8 Hz, 2H, Ar*H*), 4.50 (d, *J* = 18.9 Hz, 1H, CH_aH_bC=O), 4.42 (d, *J* = 18.9 Hz, 1H, CH_aH_bC=O), 4.21 – 4.12 (m, 2H, PhCH_2N).
^{19}F NMR (376 MHz, CD_3OD) δ -76.9 (s, 3F, CF_3COOH), -116.1 (tt, *J* = 8.7, 5.2 Hz, Ar*F*).
^{13}C{^{1}H} NMR (101 MHz, CD_3OD) δ 206.2, 164.0 (d, *J* = 246.3 Hz), 163.1 (q, *J* = 34.2 Hz), 142.6, 139.1 (d, *J* = 3.3 Hz), 132.0, 131.1, 131.0, 130.9 (d, *J* = 11.3 Hz), 130.3, 129.50, 129.46, 128.6, 118.3 (q, *J* = 293.0 Hz), 115.9 (d, *J* = 21.8 Hz), 85.7, 53.3, 51.7.

IR (cm^{-1}) 3402 (br.), 2823 (w), 1728 (m), 1674 (s), 1608 (w), 1508 (w), 1196 (s), 1142 (s).

HRMS (ESI/QTOF) m/z: $[M + H]^+$ Calcd for $C_{22}H_{21}FNO_2^+$ 350.1551; Found 350.1553.

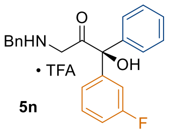

5n

(*S*)-3-(Benzylamino)-1-(3-fluorophenyl)-1-hydroxy-1-phenylpropan-2-one (5n)

Prepared according to the general procedure D3 using **2n** (165 mg, 0.400 mmol, 1.0 equiv., 89.5:10.5 *er*). The crude material was purified by precipitation/trituration to give the corresponding product **5n** (126 mg, 0.272 mmol, 68 % yield) as an amorphous off-white solid. The enantiomeric ratio was determined to be 88.0:12.0 by HPLC analysis on a Daicel Chiralpak IB N-5 column: 95:5 hexane/IPA, flow rate 1 mL/min, λ = 210 nm: τ_{Major} = 14.6 min, τ_{Minor} = 16.2 min.

Rf (1:2:98 ratio of Et_3N/MeOH/DCM) = 0.20.

$[\alpha]D^{20}$ = +7.2 (c = 0.48, MeOH, 88.0:12.0 *er*).

^{1}H NMR (400 MHz, CD_3OD) δ 7.46 – 7.41 (m, 3H, Ar*H*), 7.41 – 7.34 (m, 8H, Ar*H*), 7.18 (ddd, *J* = 7.9, 1.7, 1.0 Hz, 1H, Ar*H*), 7.13 (ddd, *J* = 10.5, 2.6, 1.7 Hz, 1H, Ar*H*), 7.09 (tdd, *J* = 8.4, 2.6, 1.0 Hz, 1H, Ar*H*), 4.50 (d, *J* = 18.8 Hz, 1H, CH_aH_bC=O), 4.44 (d, *J* = 18.8 Hz, 1H, CH_aH_bC=O), 4.17 (s, 2H, PhCH_2N).

^{19}F NMR (376 MHz, CD_3OD) δ -76.9 (s, 3F, CF_3COOH), -114.7 (ddd, J = 10.4, 8.6, 6.0 Hz, ArF).
^{13}C{^{1}H} NMR (101 MHz, CD_3OD) δ 205.9, 164.0 (d, J = 244.9 Hz), 161.0 (q, J = 34.2 Hz), 145.7 (d, J = 6.9 Hz), 142.4, 132.0, 131.07, 131.06 (d, J = 8.3 Hz), 130.9, 130.4, 129.6, 129.5, 128.6, 124.7 (d, J = 3.0 Hz), 118.2 (q, J = 295.1 Hz), 116.1 (d, J = 21.4 Hz), 115.7 (d, J = 23.5 Hz), 85.7, 53.4, 51.7.
IR (cm^{-1}) 3402 (br.), 2999 (m), 1733 (m), 1670 (s), 1591 (m), 1485 (m), 1444 (m), 1200 (s), 1139 (s).
HRMS (ESI/QTOF) m/z: $[M + H]^+$ Calcd for $C_{22}H_{21}FNO_2^+$ 350.1551; Found 350.1552.

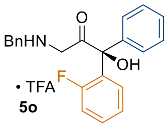

(*S*)-3-(Benzylamino)-1-(2-fluorophenyl)-1-hydroxy-1-phenylpropan-2-one (5o)

Prepared according to the general procedure D3 using **2o** (165 mg, 0.400 mmol, 1.0 equiv., 86.0:14.0 *er*). The crude material was purified by precipitation/trituration to give the corresponding product **5o** (83 mg, 0.18 mmol, 45 % yield) as an amorphous off-white solid. The enantiomeric ratio was determined to be 83.0:17.0 by HPLC analysis on a Daicel Chiralpak IB N-5 column: 95:5 hexane/IPA, flow rate 1 mL/min, λ = 210 nm: τ_{Major} = 17.2 min, τ_{Minor} = 20.3 min.

Rf (1:2:98 ratio of Et_3N/MeOH/DCM) = 0.20.

$[\alpha]D^{20}$ = +51.7 (c = 0.53, MeOH, 83.0:17.0 *er*).

^{1}H NMR (400 MHz, CD_3OD) δ 7.65 – 7.58 (m, 2H, ArH), 7.50 – 7.34 (m, 9H, ArH), 7.16 (ddd, J = 11.4, 8.2, 1.2 Hz, 1H, ArH), 7.07 (td, J = 7.6, 1.2 Hz, 1H, ArH), 6.85 (td, J = 7.8, 1.7 Hz, 1H, ArH), 4.66 (d, J = 18.8 Hz, 1H, CH_aH$_b$C=O), 4.22 (d, J = 18.8 Hz, 1H, CH$_a$$H_b$C=O), 4.17 (d, J = 13.0 Hz, 1H, PhCH_aH$_b$N), 4.13 (d, J = 13.0 Hz, 1H, PhCH$_a$$H_b$N).
^{19}F NMR (376 MHz, CD_3OD) δ -77.0 (s, 3F, CF_3COOH), -109.7 (ddd, J = 12.2, 7.8, 5.3 Hz, ArF).
^{13}C{^{1}H} NMR (101 MHz, CD_3OD) δ 205.1, 162.8 (q, J = 37.6 Hz), 162.4 (d, J = 247.5 Hz), 139.0, 132.3 (d, J = 13.3 Hz), 132.1, 132.0 (d, J = 6.9 Hz), 131.4 (d, J = 3.6 Hz), 131.1, 130.8, 130.3, 129.8, 129.5, 128.7, 125.0 (d, J = 3.2 Hz), 118.2 (d, J = 291.1 Hz), 116.9 (d, J = 22.4 Hz), 83.8, 52.0 (d, J = 3.3 Hz), 51.7.
IR (cm^{-1}) 3614 (br.), 1674 (s), 1485 (m), 1450 (w), 1196 (s), 1142 (s), 2951 (w).
HRMS (ESI/QTOF) m/z: $[M + H]^+$ Calcd for $C_{22}H_{21}FNO_2^+$ 350.1551; Found 350.1552.

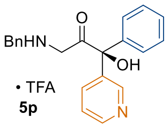

(*S*)-3-(Benzylamino)-1-hydroxy-1-phenyl-1-(pyridin-3-yl)propan-2-one (5p)

Prepared according to the modified general procedure D3 using **2p** (159 mg, 0.400 mmol, 1.0 equiv., 77.0:23.0 *er*) and mCPBA (135 mg, 0.600 mmol, 1.5 equiv.). The crude material was purified by precipitation/trituration to give the corresponding product **5p** (65 mg, 0.15 mmol, 36 % yield) as an amorphous yellow solid. The enantiomeric ratio was determined to be 75.0:25.0 by HPLC analysis on a Daicel Chiralpak IB N-5 column: 90:10 hexane/IPA, flow rate 1 mL/min, λ = 254 nm: τ_{Major} = 20.6 min, τ_{Minor} = 24.3 min.

Rf (1:2:98 ratio of Et_3N/MeOH/DCM) = 0.16.

$[\alpha]D^{20}$ = +18.1 (c = 0.50, MeOH, 75.0:25.0 *er*).

^{1}H NMR (400 MHz, CD_3OD) δ 8.79 (d, J = 2.2 Hz, 1H, HetArH), 8.74 (dd, J = 5.4, 1.5 Hz, 1H, HetArH), 8.33 (dt, J = 8.3, 1.8 Hz, 1H, HetArH), 7.86 (dd, J = 8.2, 5.4 Hz, 1H, HetArH), 7.50 – 7.37 (m, 10H, ArH), 4.70 (d, J = 18.9 Hz, 1H, CH_aH$_b$C=O), 4.43 (d, J = 18.9 Hz, 1H, CH$_a$$H_b$C=O), 4.24 (d, J = 13.0 Hz, 1H, PhCH_aH$_b$N), 4.20 (d, J = 13.1 Hz, 1H, PhCH$_a$$H_b$N).
^{19}F NMR (376 MHz, CD_3OD) δ -77.2 (s, 3F, CF_3COOH).
^{13}C{^{1}H} NMR (101 MHz, CD_3OD) δ 205.2, 161.9 (q, J = 36.6 Hz), 145.0, 144.6, 143.8, 142.5, 140.7, 131.9, 131.1, 130.9, 130.4, 130.4, 130.3, 128.0, 127.0, 117.6 (q, J = 289.5 Hz), 84.0, 53.3, 51.8.
IR (cm^{-1}) 3618 (br.), 2993 (w), 2816 (w), 1678 (s), 1466 (w), 1423 (w), 1327 (w), 1200 (s), 1142 (s).
HRMS (ESI/QTOF) m/z: $[M + H]^+$ Calcd for $C_{21}H_{21}N_2O_2^+$ 333.1598; Found 333.1603.

D.5. Unsuccessful substrates.

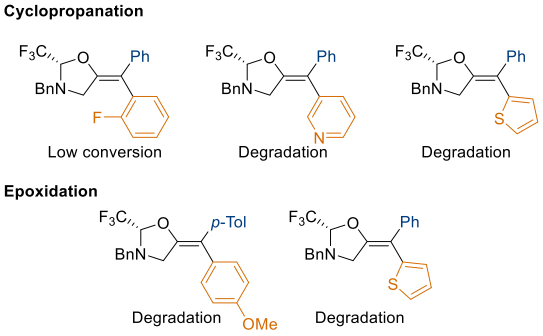

Scheme 14. Unsuccessful substrates in the cyclopropanation and epoxidation reactions.

D.6. Product modifications

(2*S*,3*R*,5*S*)-1,1-Dichloro-2-phenyl-2-(p-tolyl)-5-(trifluoromethyl)-4-oxa-6-azaspiro[2.4]heptane (6)

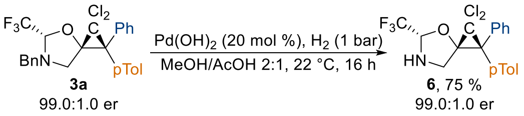

Scheme 14. Benzyl group removal from the dichlorocyclopropanation product.

An oven-dried 10 mL round-bottom flask equipped with a Teflon coated stirring bar was charged with $Pd(OH)_2/C$ (20 % (w/w), 20 mol %, 14 mg) and dichlorocyclopropane **3a** (49 mg, 0.10 mmol, 1.0 equiv., 99.0:1.0 *er*). The flask was sealed and evacuated and back-filled with N_2 three times. MeOH (1.3 mL) and AcOH (0.7 mL) were added and the suspension was stirred at room temperature for 10 minutes under a nitrogen flow. Then, a hydrogen balloon was connected to the flask through a needle and the mixture was vigorously stirred at room temperature for 16 hours. Then, the reaction mixture was degassed by bubbling nitrogen for 10 minutes and filtered through a plug of celite eluting with 5 mL of MeOH. The crude extract was washed with saturated $NaHCO_3$ and extracted with DCM (3 x 10 mL). The combined organic layer was dried over sodium sulfate, filtered and concentrated in vacuo. The crude material was purified by preparative TLC (10 % EtOAc/Pentane) to afford the corresponding product **6** (30 mg, 0.075 mmol, 75 % yield) as an off-white amorphous solid. The enantiomeric ratio was determined to be 99.0:1.0 by HPLC analysis on a Daicel Chiralpak IA column: 95:5 hexane/IPA, flow rate 1 mL/min, λ = 230 nm: τ_{Major} = 5.9 min, τ_{Minor} = 7.3 min. Absolute and relative configuration were assigned based on the relative and absolute configuration of the starting material.

Rf (5 % Et_2O/Pentane) = 0.20.

$[\alpha]D^{20}$ = +33.6 (c = 0.42, $CHCl_3$, 99.0:1.0 *er*).

^{1}H NMR (400 MHz, $CDCl_3$) δ 7.48 – 7.43 (m, 2H, Ar*H*), 7.33 – 7.27 (m, 4H, Ar*H*), 7.23 – 7.14 (m, 1H, Ar*H*), 7.11 (d, *J* = 7.9 Hz, 2H, Ar*H*), 5.28 (q, *J* = 5.5 Hz, 1H, C*H*CF$_3$), 3.73 (d, *J* = 13.2 Hz, 1H, NCH_aH$_b$C), 3.43 (d, *J* = 13.2 Hz, 1H, NCH$_a$$H_b$C), 2.27 (s, 3H, ArC$H_3$), 1.58 (br. s, 1H, N*H*).
^{19}F NMR (376 MHz, $CDCl_3$) δ -80.6 (d, *J* = 5.5Hz, CHCF_3).
^{13}C{^{1}H} NMR (101 MHz, $CDCl_3$) δ 138.7, 137.4, 135.8, 129.6, 129.4, 128.8, 128.5, 127.3, 123.2 (q, *J* = 282.8 Hz), 89.5 (q, *J* = 34.6 Hz), 75.5, 69.2, 48.1, 45.6, 21.2.
IR (cm^{-1}) 3352 (w), 3032 (w), 2927 (w), 1701 (w), 1508 (w), 1450 (w), 1284 (w), 1176 (s), 1146 (s).
HRMS (nanochip-ESI/LTQ-Orbitrap) m/z: $[M + H]^+$ Calcd for $C_{24}H_{23}ClNO^+$ 376.1463; Found 376.1467.

(2*S*,3*R*,5*S*)-1,1-Dichloro-2-phenyl-2-(p-tolyl)-5-(trifluoromethyl)-4-oxa-6-azaspiro[2.4]heptane (7)

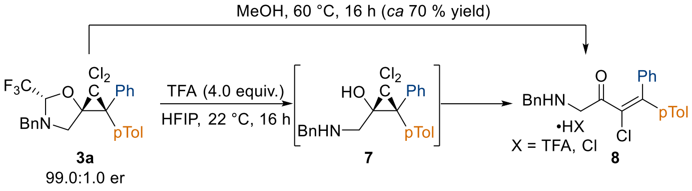

Scheme 14. Tether group removal from the dichlorocyclopropanation product and subsequent ring opening.

2 Step: an oven-dried 8 mL μW tube equipped with a Teflon coated stirring bar was charged with the dichlorocyclopropane **3a** (49 mg, 0.10 mmol, 1.0 equiv., 99.0:1.0 *er*), TFA (46 mg, 31 μL, 0.40 mmol, 4.0

equiv.) and HFIP (1.0 mL). The resulting solution was stirred at 22 °C for 16 hours. Then, the reaction mixture was concentrated in vacuo. The crude material was dissolved in a small amount of DCM, and excess pentane was added to induce precipitation. The resulting suspension was concentrated in vacuo with minimal heating, and the resulting residue was triturated with pentane (5 x 1 mL). The product **7** could be identified in the residue, but degradation was observed over time.

Representative signals:

^{1}H NMR (400 MHz, $CDCl_3$) δ 4.24 (d, *J* = 13.0 Hz, 1H, PhCH_aH$_b$N), 4.15 (d, *J* = 13.0 Hz, 1H, PhCH$_a$$H_b$N), 3.89 (d, *J* = 13.6 Hz, 1H, NCH_aH$_b$C), 3.12 (d, *J* = 13.6 Hz, 1H, NCH$_a$$H_b$C), 2.24 (s, 3H, ArC$H_3$).

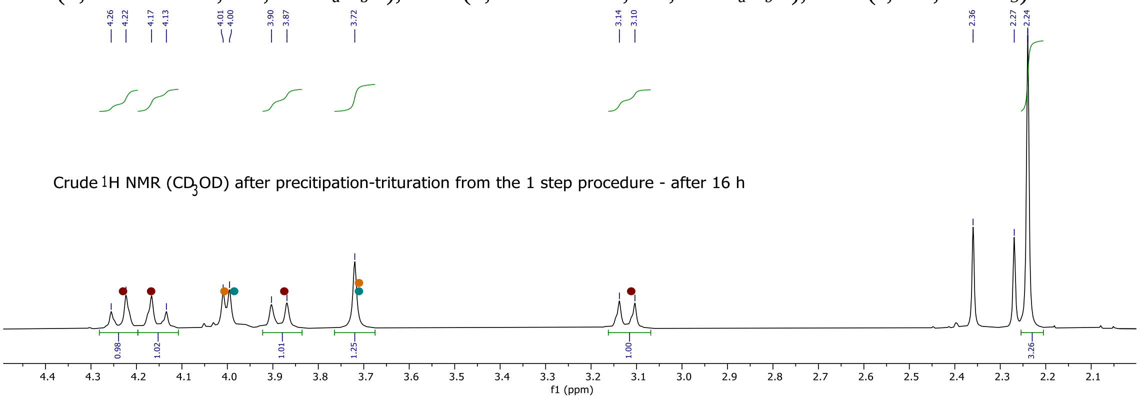

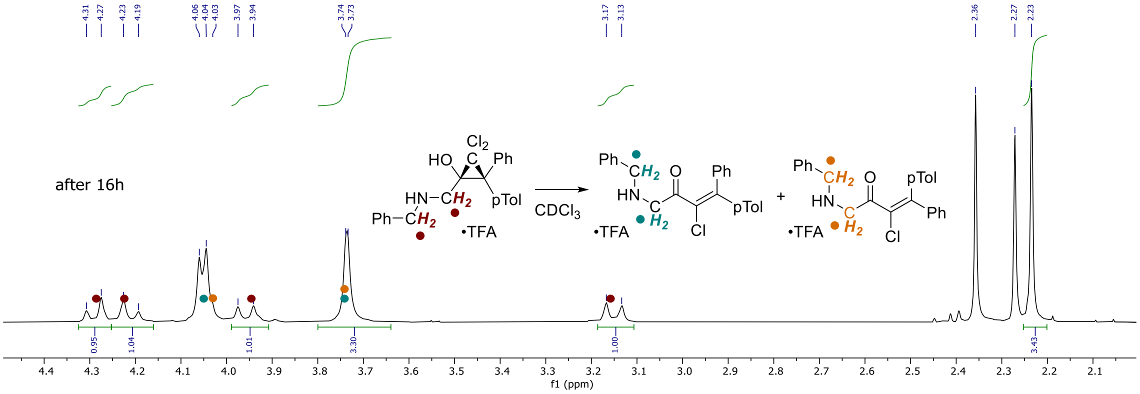

1 Step: an oven-dried 8 mL µW tube equipped with a Teflon coated stirring bar was charged with the dichlorocyclopropane **3a** (74 mg, 0.15 mmol, 1.0 equiv., 99.0:1.0 *er*) and MeOH (1.5 mL). The resulting solution was stirred at 60 °C for 16 hours. Then, the reaction mixture was concentrated in vacuo. The crude material was dissolved in small amount of DCM, and excess pentane was added to induce precipitation. The resulting suspension was concentrated in vacuo with minimal heating, and the resulting residue was triturated with pentane (5 x 1 mL). The product **8** (43 mg, 0.10 mmol, 70 % yield, **impure**) was obtained as a gray solid.

In order to make direct spectroscopic comparison with the TFA salt obtained in the previous experiment, a small sample was dissolved in methanol, filtered through a pad of $NaHCO_3$ directly into DCM containing a few drops of TFA. The resulting solution was concentrated in vacuo.

^{1}H NMR (400 MHz, CD_3OD) δ 7.46 – 7.37 (m, 6H, Ar*H*), 7.36 – 7.31 (m, 2H, Ar*H*), 7.31 – 7.27 (m, 2H, Ar*H*), 7.22 – 7.17 (m, 2H, Ar*H*), 7.04 (d, *J* = 8.2 Hz, 2H, Ar*H*), 4.08 (s, 2H, NCH_2C-O), 3.91 (s, 2H, PhCH_2N), 2.36 (s, 3H, ArCH_3).

^{13}C{^{1}H} NMR (101 MHz, CD_3OD) δ 191.2, 154.4, 141.7, 140.7, 137.6, 131.6, 131.1, 130.9, 130.8, 130.7, 130.52, 130.47, 130.4, 129.4, 125.4, 54.5, 51.8, 21.5.

IR (cm^{-1}) 3417 (br.), 3032 (s), 2931 (s), 2765 (s), 1697 (s), 1562 (s), 1450 (s).

HRMS (nanochip-ESI/LTQ-Orbitrap) m/z: $[M + H]^+$ Calcd for $C_{24}H_{23}ClNO^+$ 376.1463; Found 376.1467.

Then NMR of the crude product obtained from the **2 step** procedure was measured in CD_3OD and compared with the NMR of the **8** TFA salt from the **1 step** procedure:

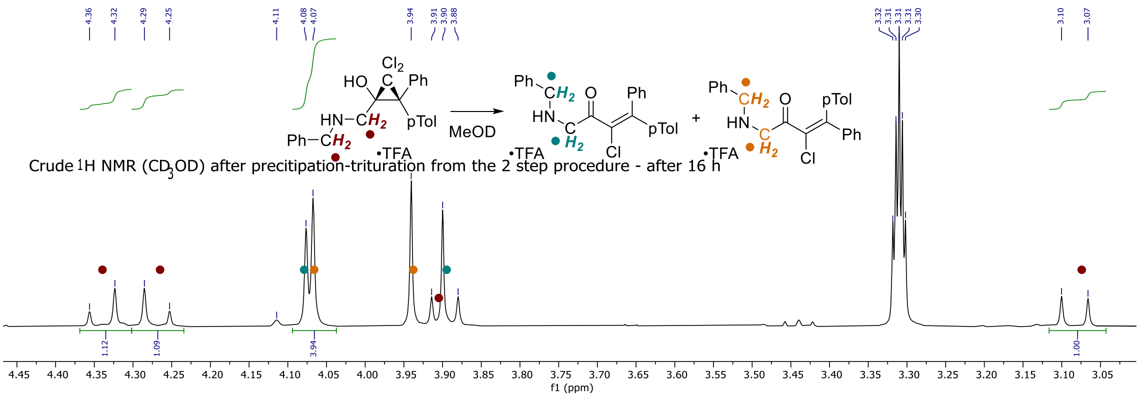

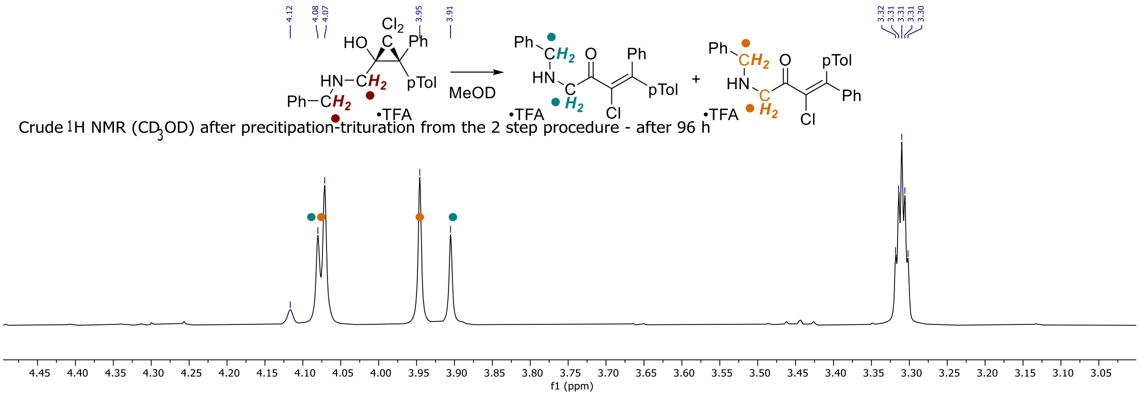

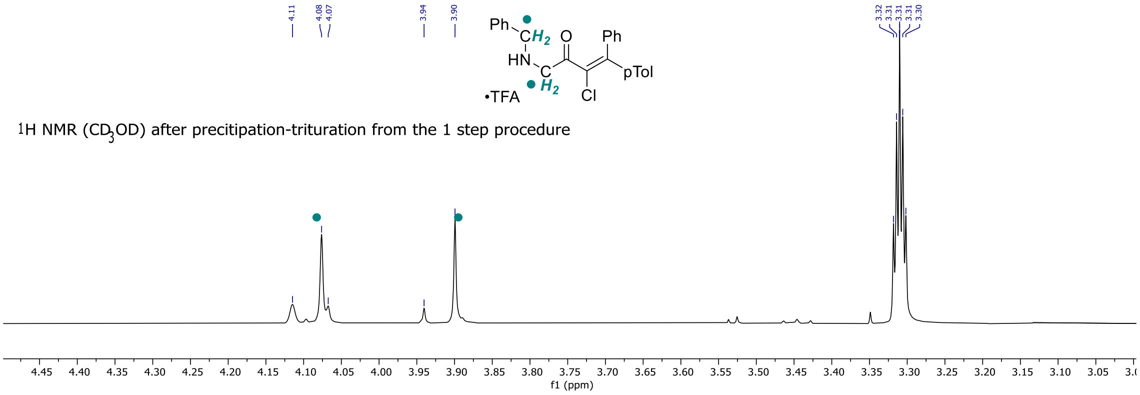

(1*S*)-3-(Benzylamino)-1-phenyl-1-(p-tolyl)propane-1,2-diol (9)

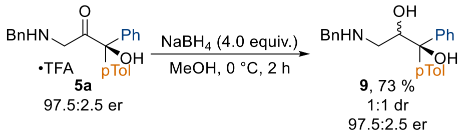

Scheme 14. Reduction of the aminoketone.

An oven-dried 10 mL round-bottom flask equipped with a Teflon coated stirring bar was charged with aminoketone **5a** (69 mg, 0.15 mmol, 1.0 equiv., 97.5:2.5 *er*) and MeOH (1.5 mL). The resulting solution was cooled down to 0 °C. Then, $NaBH_4$ (23 mg, 0.60 mmol, 4.0 equiv.) was added as a solid portion-wise. Evolution of gas is observed. Then, the reaction mixture was stirred at 0 °C for 2 h. The reaction mixture was then quenched by the addition of H_2O (3 mL). The resulting mixture was extracted with EtOAc (3 x 5 mL). The combined organic layers were dried over anhydrous Na_2SO_4, filtered and concentrated *in vacuo*. The crude material was purified by preparative TLC (5 % MeOH/DCM) to afford the corresponding product **9** (38 mg, 0.11 mmol, 73 % yield, 1:1 dr) as an off-white amorphous solid.

For asymmetric assessment, the product was protected with an *N*-Cbz group – an oven-dried 8 mL round bottom µW vial equipped with a Teflon coated stirring bar was charged with aminodiol 9 (5 mg, 0.01 mmol, 1.0 equiv.), benzyl chloroformiate (4 µL, 5 mg, 0.03 mmol, 2.0 equiv.), triethyl amine (5 µL, 4 mg, 0.03

mmol, 2.0 equiv.) and DCM (0.5 mL). The resulting mixture was stirred for 2 h at room temperature. Then, the reaction mixture was concentrated in vacuo and purified by preparative TLC (20 % EtOAc/Pentane). The enantiomeric ratio was determined to be 97.5:2.5 by HPLC analysis on a Daicel Chiralpak IB N-5 column: 80:20 hexane/IPA, flow rate 1 mL/min, λ = 210 nm: τ_{Major} = 11.2 and 13.2 min, τ_{Minor} = 9.0 and 15.4 min. Absolute and relative configuration were assigned based on the absolute configuration of the starting material.

The NMR spectra are reported as observed without attempts to identify each diastereoisomer due to the heavy overlap of the peaks.

Rf (5 % MeOH/DCM) = 0.15.

[α]D^{20} = -8.4 (c = 0.50, $CHCl_3$, 1:1 *dr*, 97.5:2.5 *er*).

^{1}H NMR (400 MHz, $CDCl_3$) δ 7.59 (app. dd, *J* = 8.4, 1.3 Hz, 2H, Ar*H*), 7.48 (app. d, *J* = 8.3 Hz, 2H, Ar*H*), 7.45 – 7.40 (m, 2H, Ar*H*), 7.36 – 7.29 (m, 8H, Ar*H*), 7.29 – 7.22 (m, 8H, Ar*H*), 7.18 (app. d, *J* = 17.4 Hz, 2H, Ar*H*), 7.14 (app. d, *J* = 8.1 Hz, 2H, Ar*H*), 7.07 (app. d, *J* = 8.0 Hz, 2H, Ar*H*), 4.58 (app. td, *J* = 4.1, 1.1 Hz, 2H, C*H*-OH), 3.70 (app. dd, *J* = 12.9, 1.5 Hz, 2H, $PhCH_aH_bN$), 3.60 (app. dd, *J* = 13.0, 5.4 Hz, 2H, $PhCH_a\mathit{H_b}N$), 3.40 (br. s, 6H, all OH and NH), 2.87 (app. ddd, *J* = 12.5, 6.9, 3.8 Hz, 2H, $NHC\mathit{H_a}H_bCH$-OH), 2.67 (app. td, *J* = 12.7, 4.3 Hz, 2H, $NHCH_a\mathit{H_b}CH$-OH), 2.30 (s, 3H, ArC*H*$_3$), 2.29 (s, 3H, ArC*H*$_3$).
^{13}C{^{1}H} NMR (101 MHz, $CDCl_3$) δ 145.6, 145.34, 142.5, 142.2, 139.1, 136.8, 136.4, 129.3, 129.1, 128.7, 128.5, 128.4, 127.5, 127.1, 126.8, 126.30, 126.25, 125.5, 125.4, 80.8, 77.5, 77.2, 76.8, 72.2, 54.0, 49.72, 49.66, 21.13, 21.11.
IR (cm^{-1}) 3305 (br.), 3059 (m), 3024 (m), 2920 (m), 2858 (m), 1658 (w), 1450 (m), 1215 (w), 1103 (m).
HRMS (ESI/QTOF) m/z: [M + H]$^+$ Calcd for $C_{23}H_{26}NO_2^+$ 348.1958; Found 348.1955.

(1*S*)-2-((Benzylamino)methyl)-1,4-diphenyl-1-(p-tolyl)but-3-yne-1,2-diol (10)

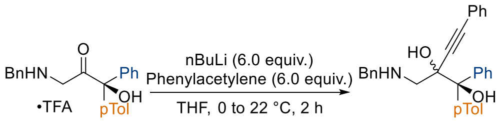

5a
97.5:2.5 er

10
1:1 dr
97.5:2.5 er

Scheme 14. Alkynylation of the aminoketone.

An oven-dried 10 mL round-bottom flask equipped with a Teflon coated stirring bar was charged with phenylacetylene (132 µL, 123 mg, 1.20 mmol, 6.0 equiv.) and THF (2.0 mL). Then, the resulting solution was cooled down to 0 °C and nBuLi (2.5 M, 0.48 mL, 1.2 mmol, 6.0 equiv.) was added dropwise. The resulting mixture was stirred at 0 °C for 10 minutes. Then, a solution of the aminoketone **5a** (92 mg, 0.20 mmol, 1.0 equiv., 97.5:2.5 *er*) in THF (2.0 mL) was added dropwise. The flask was warmed to 22 °C and stirred for 2 hours. The, reaction mixture was then cooled down to 0 °C and quenched by the addition of sat. aq. NH_4Cl (4 mL). Then the resulting mixture was basified with 1 M NaOH (8 mL) and extracted with EtOAc (3 x 5 mL) The combined organic layer was dried over sodium sulfate, filtered and concentrated in vacuo. The crude material was purified by flash column chromatography on silica (0 - 5 % MeOH/DCM) to afford the corresponding product **10** (80 mg, 0.18 mmol, 89 % yield, 1:1 dr) as a brown oil. The enantiomeric ratio was determined to be 97.5:2.5 by HPLC analysis on a Daicel Chiralpak IB N-5 column: 80:20 hexane/IPA, flow rate 1 mL/min, λ = 210 nm: τ_{Major} = 8.7 and 11.6 min, τ_{Minor} = 8.1 and 10.9 min. Absolute and relative configuration were assigned based on the absolute configuration of the starting material.

The NMR spectra are reported as observed without attempts to identify each diastereomer due to the heavy overlap of the peaks.

Rf (5 % MeOH/DCM) = 0.19 – 0.62.

[α]D^{20} = -8.5 (c = 0.50, $CHCl_3$, 1:1 *dr*, 97.5:2.5 *er*).

^{1}H NMR (400 MHz, $CDCl_3$) δ 7.83 – 7.76 (m, 2H, Ar*H*), 7.76 – 7.70 (m, 2H, Ar*H*), 7.66 (app. d, *J* = 8.4 Hz, 2H, Ar*H*), 7.61 (app. d, *J* = 8.2 Hz, 2H, Ar*H*), 7.33 – 7.17 (m, 26H, Ar*H*), 7.07 (app. d, *J* = 8.1 Hz, 4H, Ar*H*), 3.77 (app. d, *J* = 13.1 Hz, 2H, $PhC\mathit{H_a}H_bN$), 3.67 (app. d, *J* = 13.0 Hz, 2H, $PhCH_a\mathit{H_b}N$), 3.06 (app. dd, *J* = 12.3, 2.9 Hz, 2H, $NC\mathit{H_a}H_bC$-O), 2.94 (app. dd, *J* = 12.3, 5.0 Hz, 2H, $NCH_a\mathit{H_b}C$-O), 2.30 (s, 3H, ArC*H*$_3$), 2.28 (s, 3H, ArC*H*$_3$).
^{13}C{^{1}H} NMR (101 MHz, $CDCl_3$) δ 144.5, 143.7, 141.4, 140.6, 139.2, 136.87, 136.85, 131.6, 128.72, 128.67, 128.6, 128.43, 128.38, 128.31, 128.29, 128.0, 127.9, 127.7, 127.6, 127.5, 127.22, 127.20, 122.7, 91.1, 88.3, 82.4, 73.8, 55.82, 55.8, 53.9, 21.20, 21.17.
IR (cm^{-1}) 3541 (br.), 3062 (w), 3024 (w), 2924 (w), 2858 (w), 1662 (w), 1489 (w), 1450 (m), 1157 (m)

HRMS (ESI/QTOF) m/z: $[M + H]^+$ Calcd for $C_{31}H_{30}NO_2^+$ 448.2271; Found 448.2276.

E. X-Ray Crystallographic Data

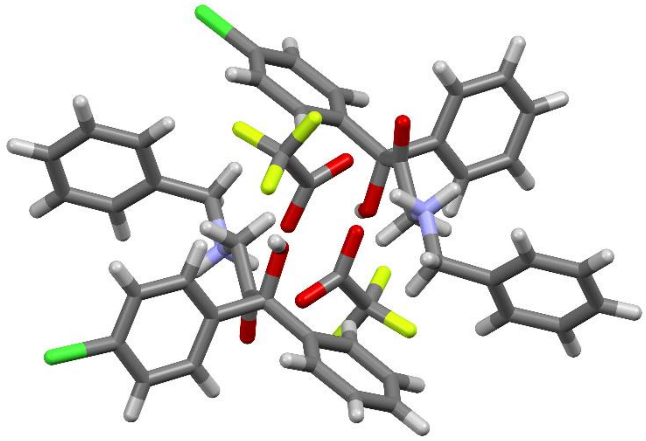

Experimental. Single clear pale colourless prism-shaped crystals of **5l** were used as supplied. A suitable crystal with dimensions 0.14 × 0.10 × 0.09 mm^3 was selected and mounted on a XtaLAB Synergy R, DW system, HyPix-Arc 150 diffractometer. The crystal was kept at a steady *T* = 140.00(10) K during data collection. The structure was solved with the **ShelXT** (Sheldrick, 2015) solution program using dual methods and by using **Olex2** 1.3 (Dolomanov et al., 2009) as the graphical interface. The model was refined with **ShelXL** 2018/3 (Sheldrick, 2015) using full matrix least squares minimisation on $\boldsymbol{F^2}$.

Crystal Data. $C_{24}H_{21}ClF_3NO_4$, M_r = 479.87, triclinic, *P*1 (No. 1), a = 9.3806(5) Å, b = 11.1506(4) Å, c = 12.1007(3) Å, *a* = 82.342(3)°, *b* = 68.997(4)°, *g* = 74.273(4)°, *V* = 1136.48(9) $Å^3$, *T* = 140.00(10) K, *Z* = 2, *Z'* = 2, *m*(Cu K_a) = 1.988, 28074 reflections measured, 8400 unique (R_{int} = 0.0663) which were used in all calculations. The final wR_2 was 0.2162 (all data) and R_1 was 0.0672 (I≥2 *s*(I)).

Table S1. Single crystal X-Ray parameters.

**Compound**	**5l**
Formula	$C_{24}H_{21}ClF_3NO_4$
$D_{calc.}$/ g cm^{-3}	1.402
m/mm^{-1}	1.988
Formula Weight	479.87
Colour	clear pale colourless
Shape	prism-shaped
Size/mm^3	0.14×0.10×0.09
T/K	140.00(10)
Crystal System	triclinic
Flack Parameter	0.18(3)
Hooft Parameter	0.141(9)
Space Group	*P*1
a/Å	9.3806(5)
b/Å	11.1506(4)
c/Å	12.1007(3)
a/°	82.342(3)
b/°	68.997(4)
g/°	74.273(4)
V/$Å^3$	1136.48(9)
Z	2
Z'	2
Wavelength/Å	1.54184
Radiation type	Cu K_a
Q_{min}/°	3.916
Q_{max}/°	75.578
Measured Refl's.	28074
Indep't Refl's	8400
Refl's I≥2 *s*(I)	5255
R_{int}	0.0663
Parameters	608
Restraints	3
Largest Peak	0.314
Deepest Hole	-0.367
GooF	1.047
wR_2 (all data)	0.2162
wR_2	0.1904
R_1 (all data)	0.1035
R_1	0.0672

F. References

1. G. M. Sheldrick, SHELXT – Integrated space-group and crystal-structure determination. *Acta Cryst A* **2015**, *71*, 3–8.
2. G. M. Sheldrick, Crystal structure refinement with SHELXL. *Acta Cryst C* **2015**, *71*, 3–8.
3. S. S. Zalesskiy, V. P. Ananikov, $Pd_2(dba)_3$ as a Precursor of Soluble Metal Complexes and Nanoparticles: Determination of Palladium Active Species for Catalysis and Synthesis. *Organometallics* **2012**, *31*, 2302–2309.
4. L. Buzzetti, M. Puriņš, P. D. G. Greenwood, J. Waser, Enantioselective Carboetherification/Hydrogenation for the Synthesis of Amino Alcohols via a Catalytically Formed Chiral Auxiliary. *J. Am. Chem. Soc.* **2020**, *142*, 17334-17339.
5. D. Althuon, F. Rönicke, D. Fürniss, J. Quan, I. Wellhöfer, M. Jung, U. Schepers, S. Bräse, Functionalized triazolopeptoids – a novel class for mitochondrial targeted delivery. *Org. Biomol. Chem.* **2015**, *13*, 4226–4230.
6. Y. Shao, F. Zhang, J. Zhang, X. Zhou, Lanthanide-Catalyzed Reversible Alkynyl Exchange by Carbon–Carbon Single-Bond Cleavage Assisted by a Secondary Amino Group. *Angew. Chem. Int. Ed.* **2016**, *55*, 11485–11489.
7. García-Domínguez, P.; Fehr, L.; Rusconi, G.; Nevado, C., Palladium-catalyzed incorporation of atmospheric CO2: Efficient synthesis of functionalized oxazolidinones. *Chem. Sci.* **2016**, *7*, 3914–3918.
8. P. D. G. Greenwood, E. Grenet, J. Waser, Palladium-Catalyzed Carbo-Oxygenation of Propargylic Amines using in Situ Tether Formation. *Chem. Eur. J.* **2019**, *25*, 3010–3013.
9. G. Suez, V. Bloch, G. Nisnevich, M. Gandelman, Design and Development of Bioinspired Guanine-Based Organic Catalyst for Asymmetric Catalysis. *Eur. J. Org. Chem.* **2012**, *2012*, 2118–2122.
10. S. E. Denmark, J. P. Edwards, A Comparison of (Chloromethyl)- and (Iodomethyl)Zinc Cyclopropanation Reagents. *J. Org. Chem.* **1991**, *56*, 6974–6981.
11. J. C. Lorenz, J. Long, Z. Yang, S. Xue, Y. Xie, Y. Shi, A Novel Class of Tunable Zinc Reagents (RXZnCH2Y) for Efficient Cyclopropanation of Olefins. *J. Org. Chem.* **2004**, *69*, 327–334.
12. F. Wang, T. Luo, J. Hu, Y. Wang, H. S. Krishnan, P. V. Jog, S. K. Ganesh, G. K. S. Prakash, G. A. Olah, Synthesis of Gem-Difluorinated Cyclopropanes and Cyclopropenes:

Trifluoromethyltrimethylsilane as a Difluorocarbene Source. *Angewandte Chemie International Edition* **2011**, *50*, 7153–7157.

13. T. N. Grant, F. G. West, A New Approach to the Nazarov Reaction via Sequential Electrocyclic Ring Opening and Ring Closure. *J. Am. Chem. Soc.* **2006**, *128*, 9348–9349.

G. NMR Spectra

G.1.Cyclopropanation products

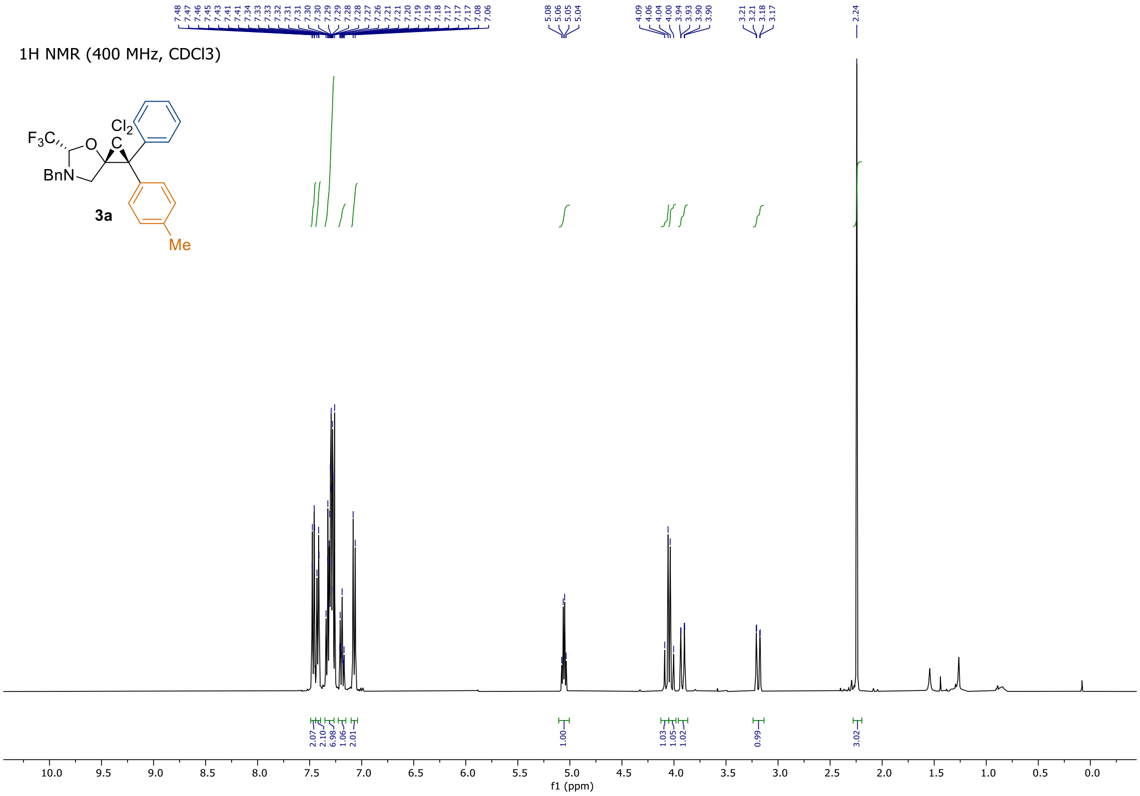

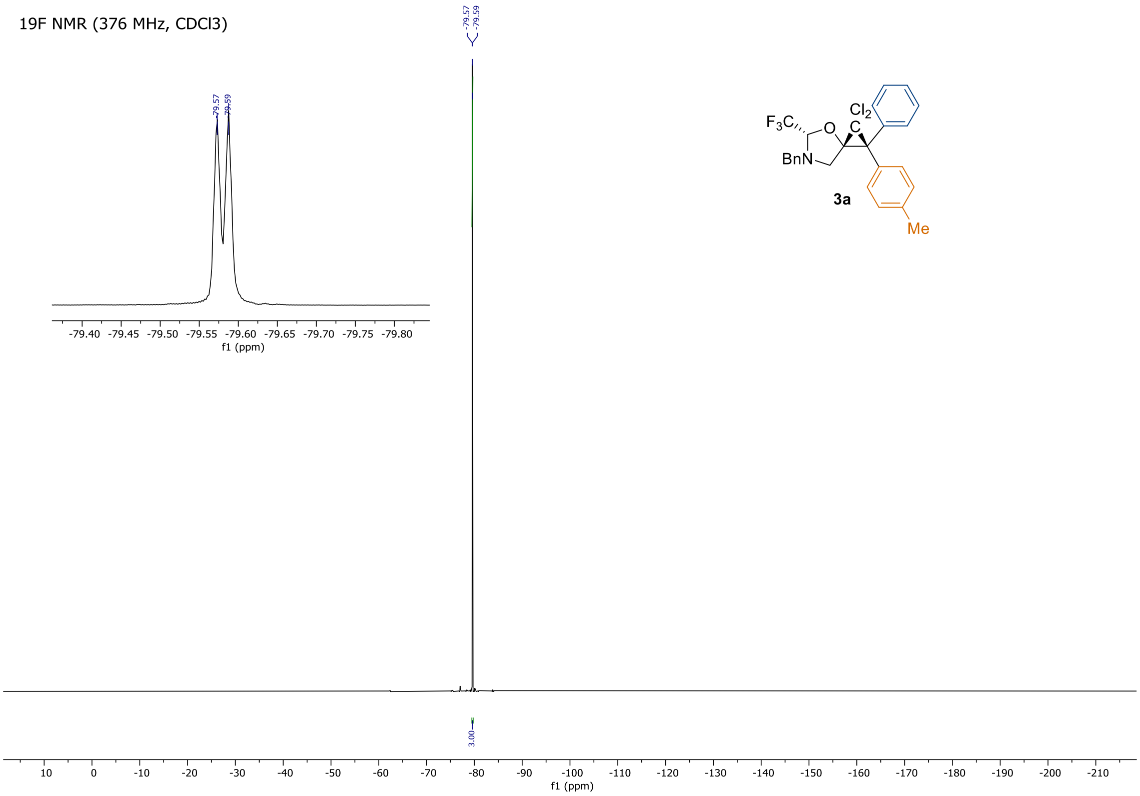

13C{1H} NMR (101 MHz, CDCl3)

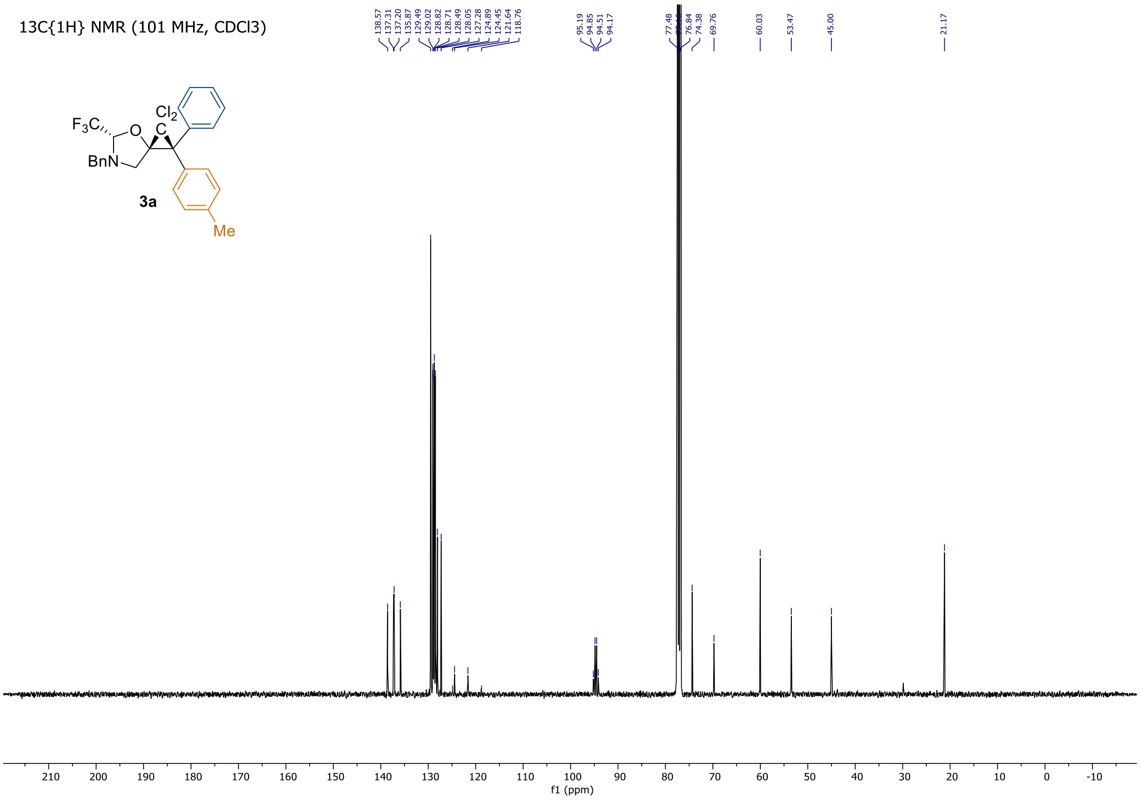

1H NMR (400 MHz, CDCl3)

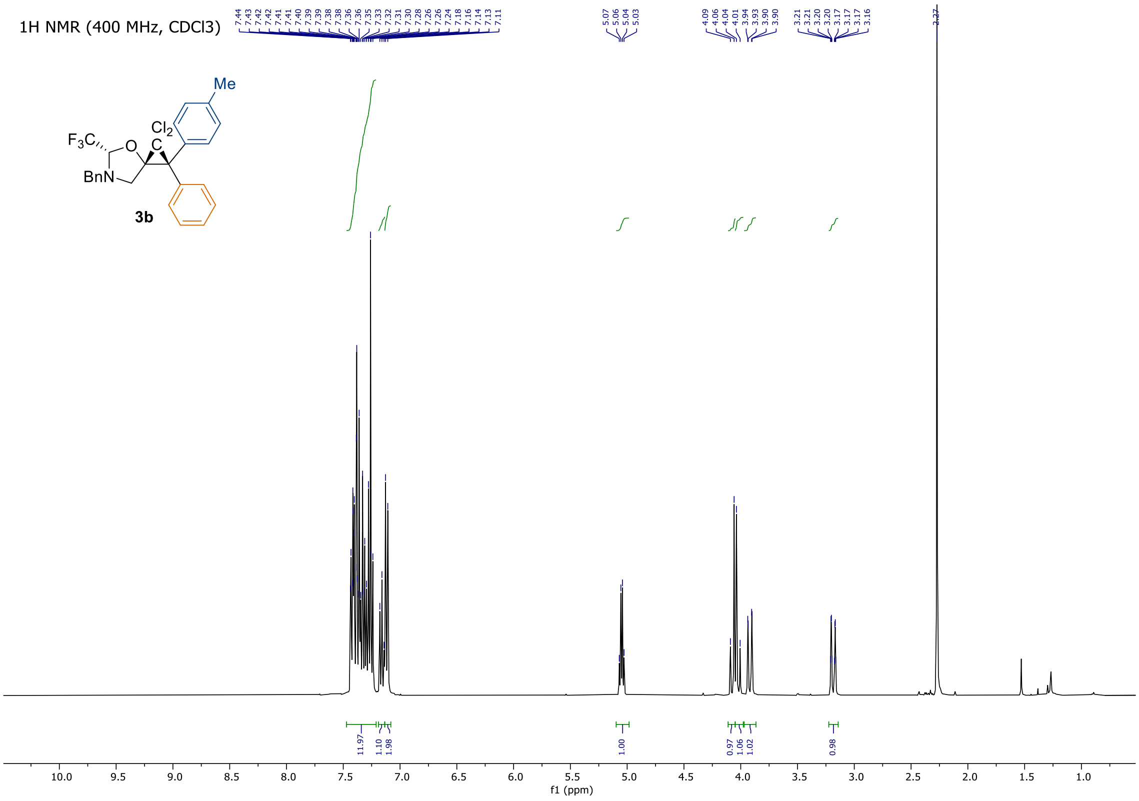

19F NMR (376 MHz, CDCl3)

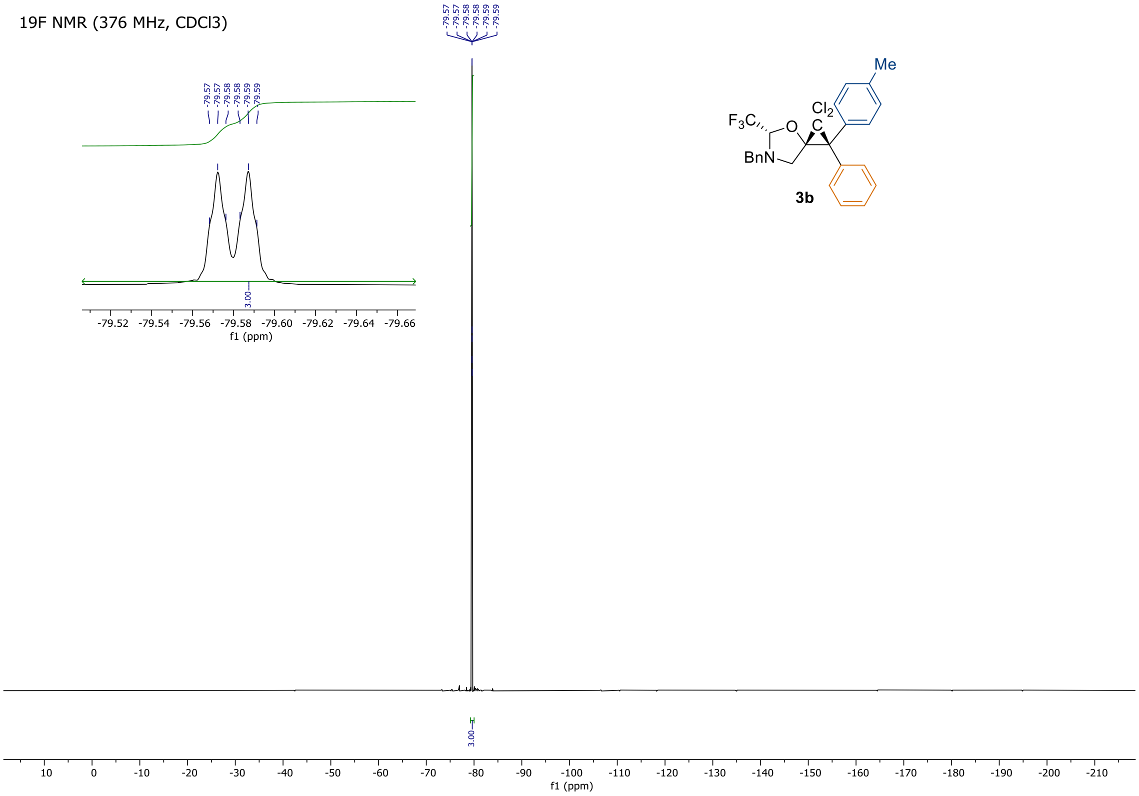

13C{1H} NMR (101 MHz, CDCl3)

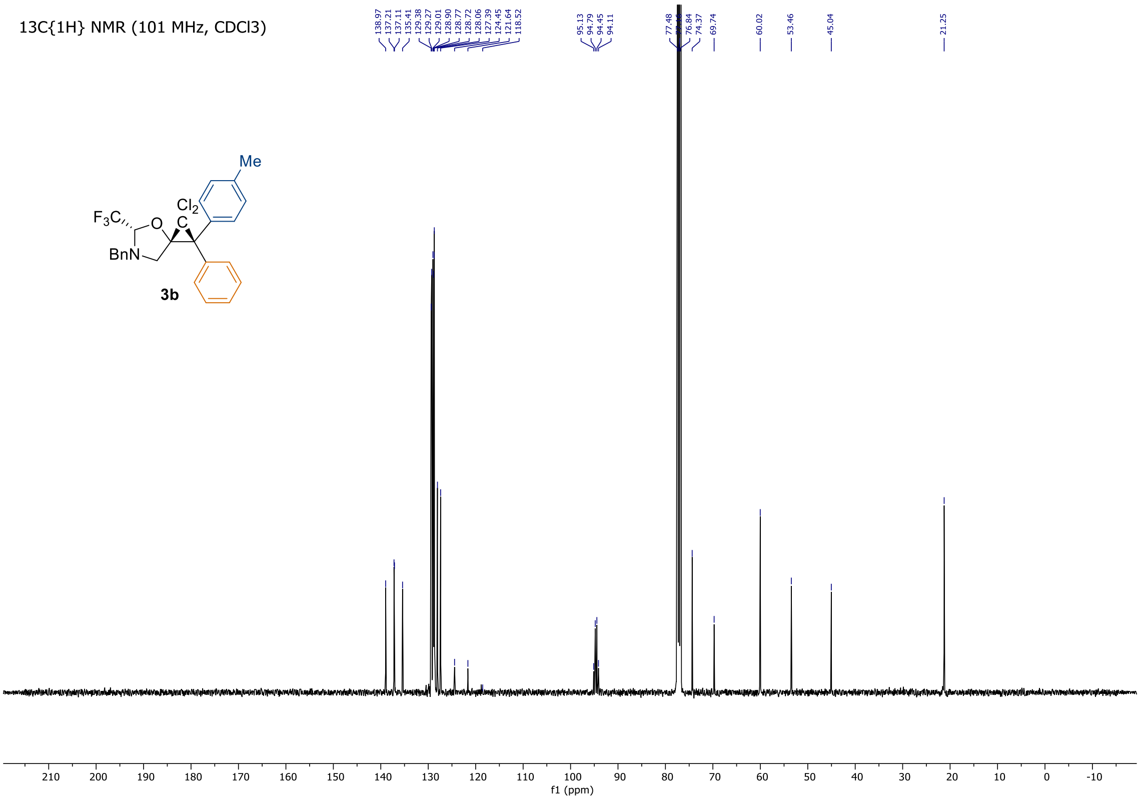

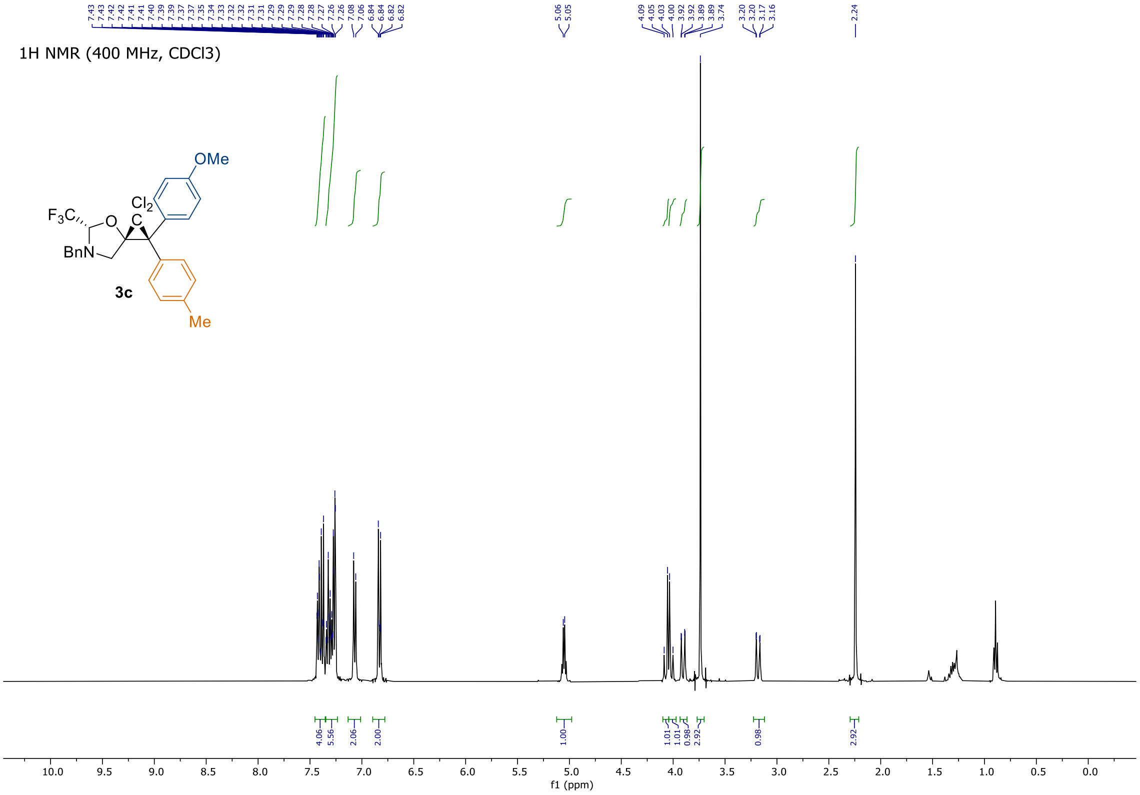
1H NMR (400 MHz, CDCl3)
OMe
Cl2
F3C
O
C
BnN
3c
Me
f1 (ppm)

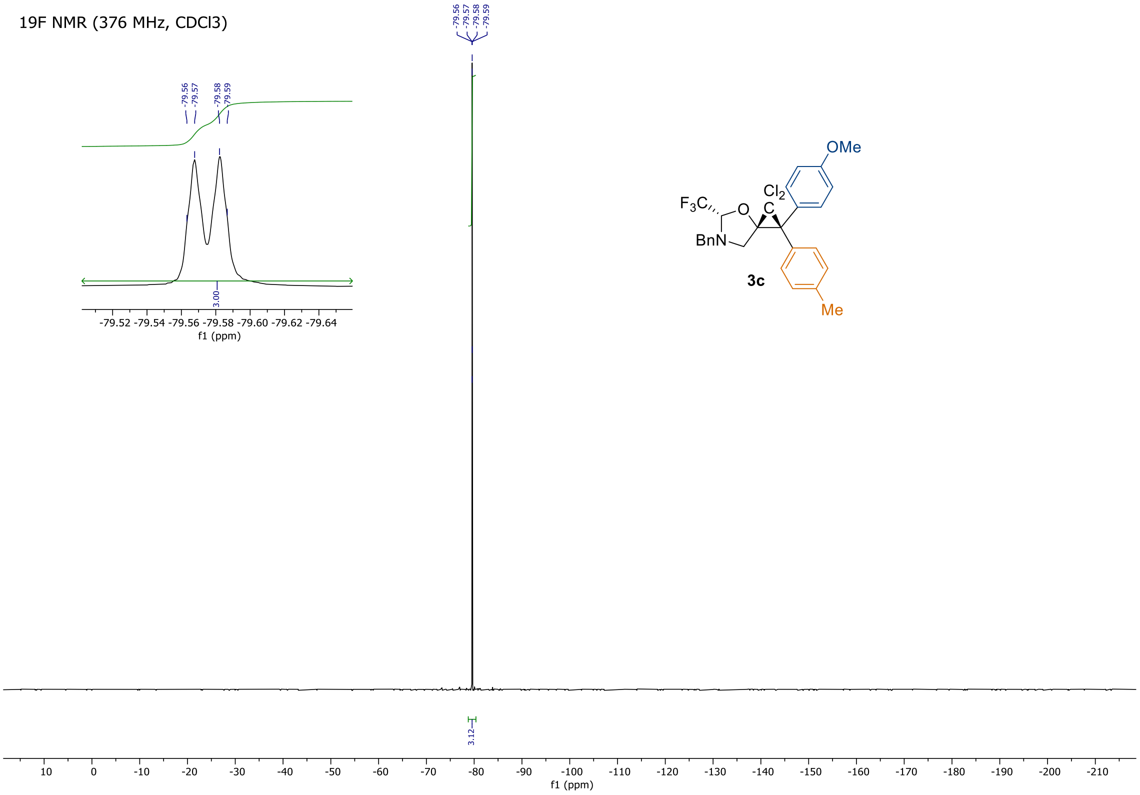
19F NMR (376 MHz, CDCl3)
OMe
Cl2
F3C
O
C
BnN
3c
Me
f1 (ppm)

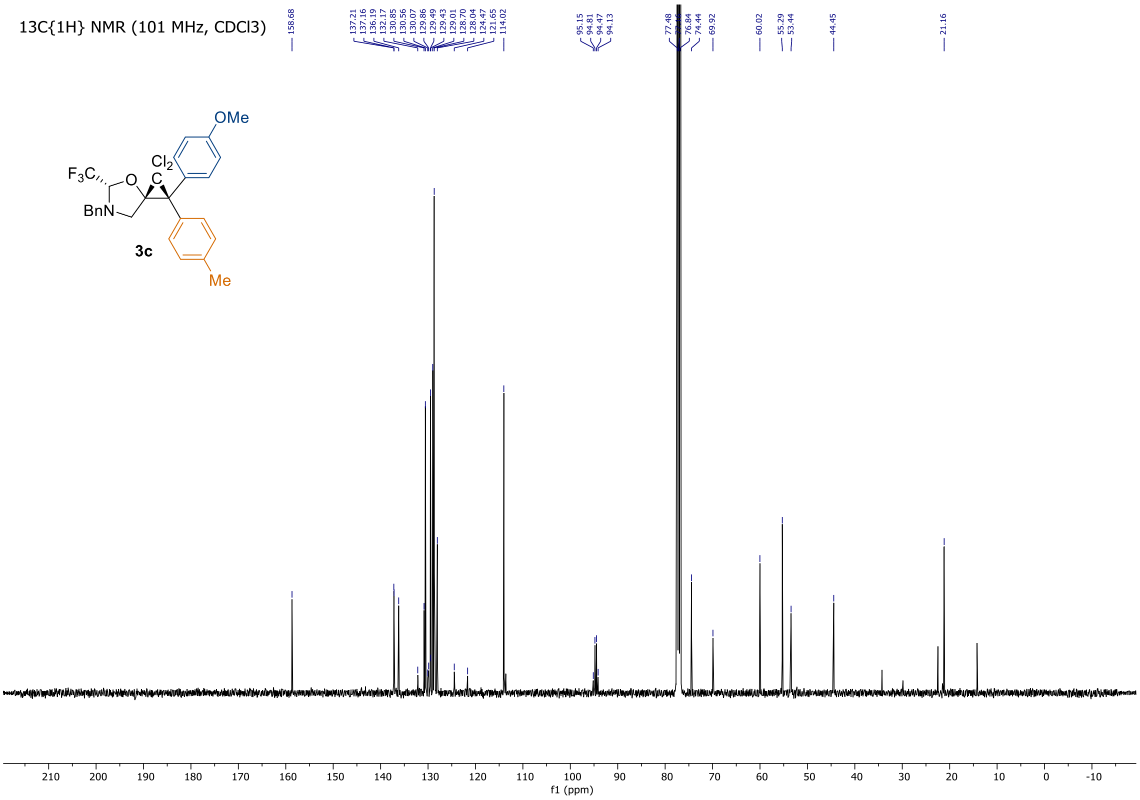

13C{1H} NMR (101 MHz, CDCl3)
158.68
137.21
137.16
136.19
132.17
130.85
130.56
130.07
129.86
129.49
129.43
129.01
128.70
128.04
124.47
121.65
114.02
95.15
94.81
94.47
94.13
77.48
76.84
74.44
69.92
60.02
55.29
53.44
44.45
21.16
OMe
F_3C
Cl_2
C
O
BnN
3c
Me
f1 (ppm)

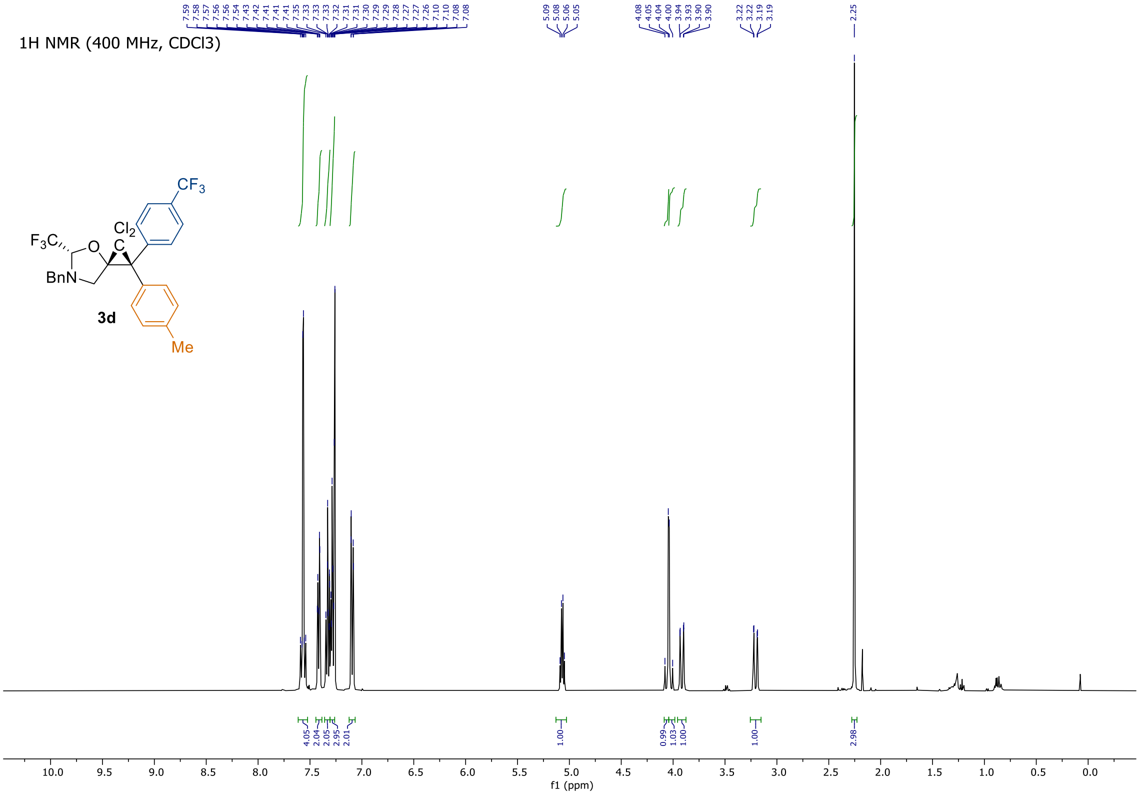

1H NMR (400 MHz, CDCl3)
7.59 7.58 7.57 7.56 7.54 7.43 7.42 7.41 7.41 7.35 7.33 7.33 7.33 7.32 7.31 7.30 7.29 7.29 7.28 7.27 7.27 7.26 7.10 7.10 7.08 7.08
5.09 5.08 5.06 5.05
4.08 4.05 4.04 4.00 3.94 3.93 3.90 3.90
3.22 3.22 3.19 3.19
2.25
CF_3
F_3C
Cl_2
C
O
BnN
3d
Me
4.05 2.04 2.05 2.95 2.01
1.00
0.99 1.03 1.00
1.00
2.98
f1 (ppm)

19F NMR (376 MHz, CDCl3)

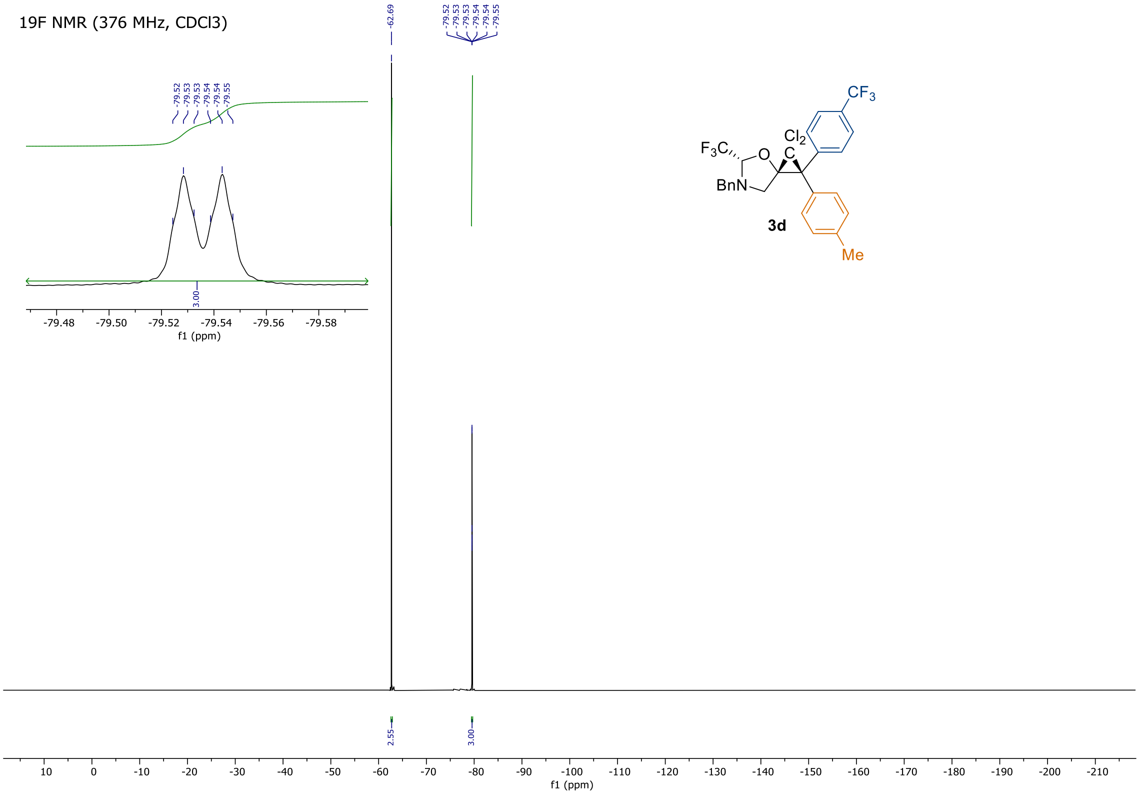

13C{1H} NMR (101 MHz, CDCl3)

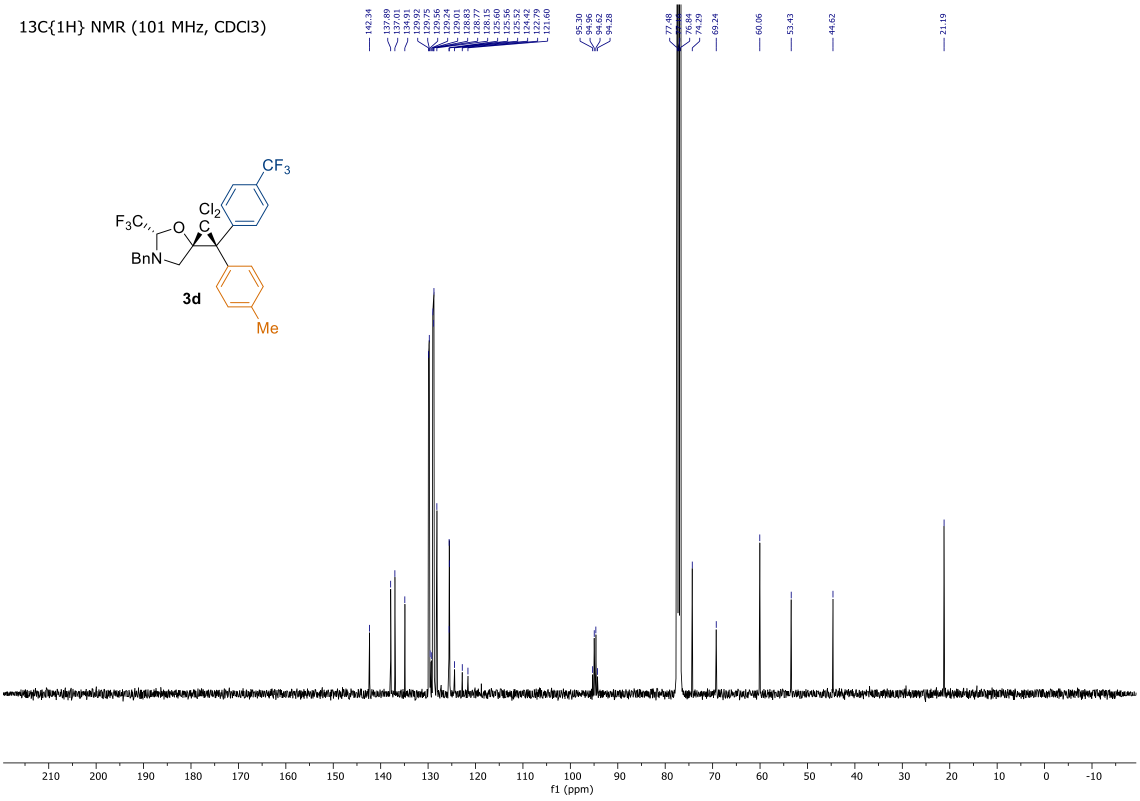

1H NMR (400 MHz, CDCl3)

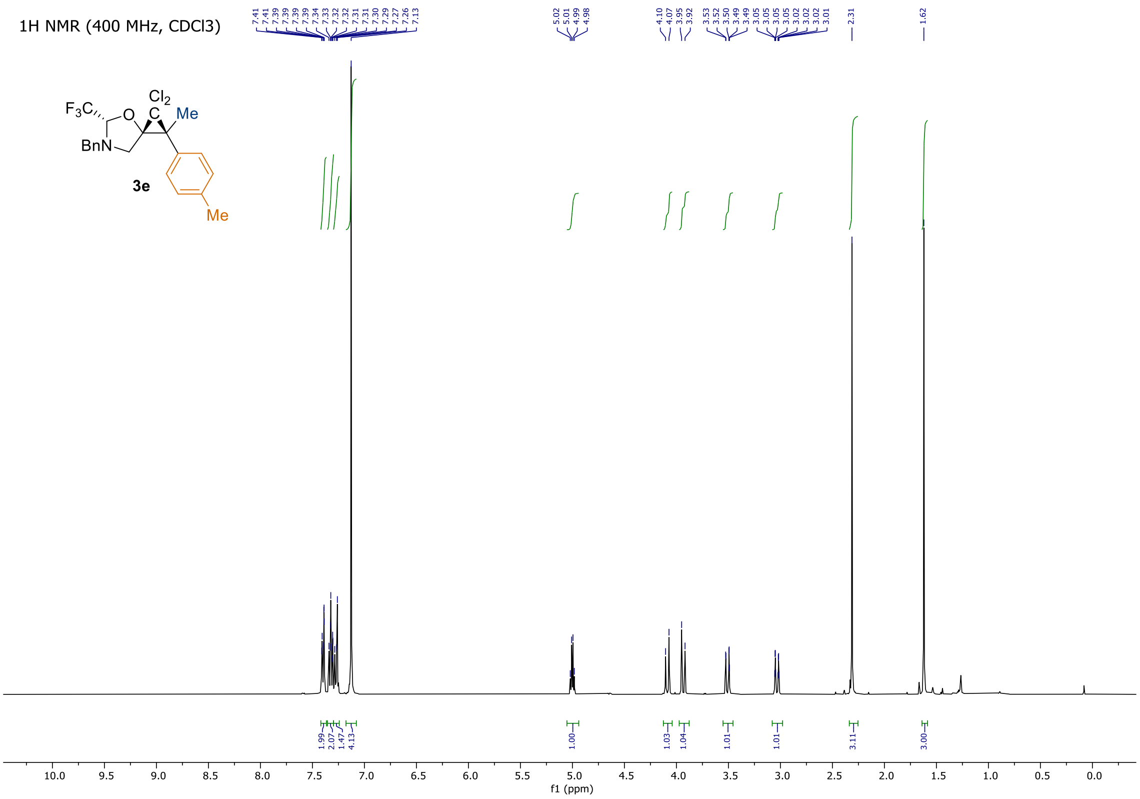

19F NMR (376 MHz, CDCl3)

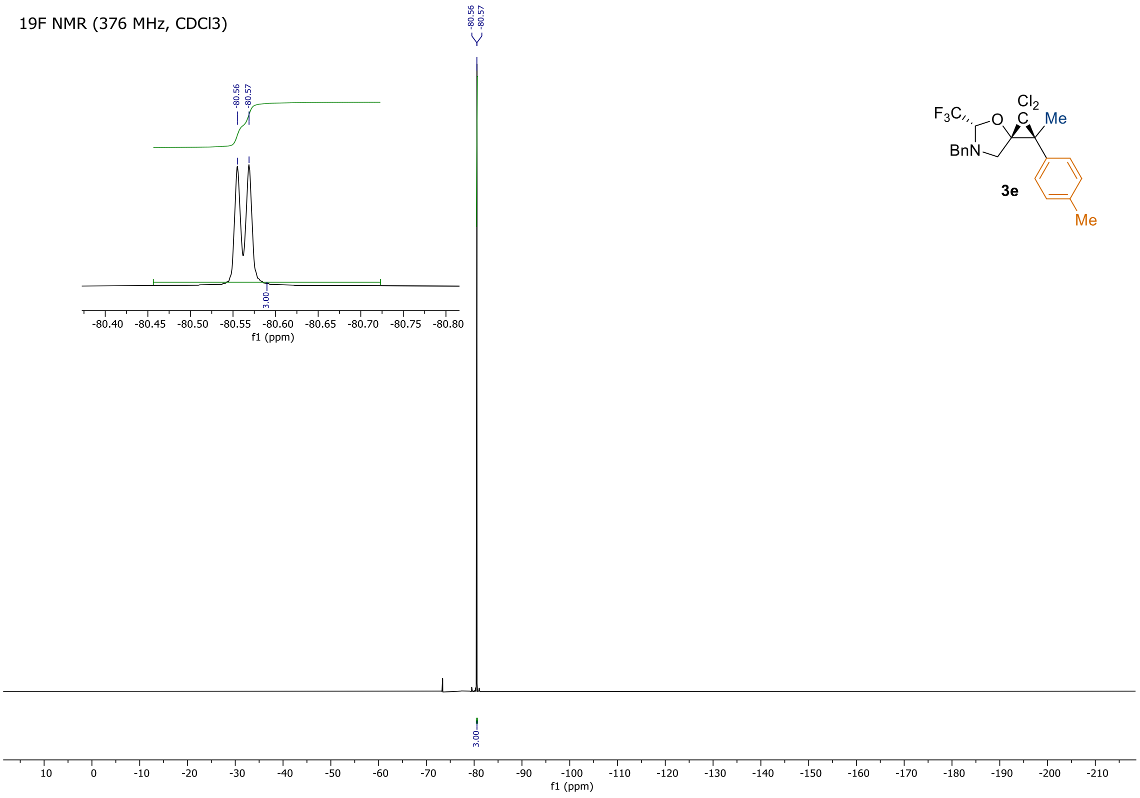

13C{1H} NMR (101 MHz, CDCl3)

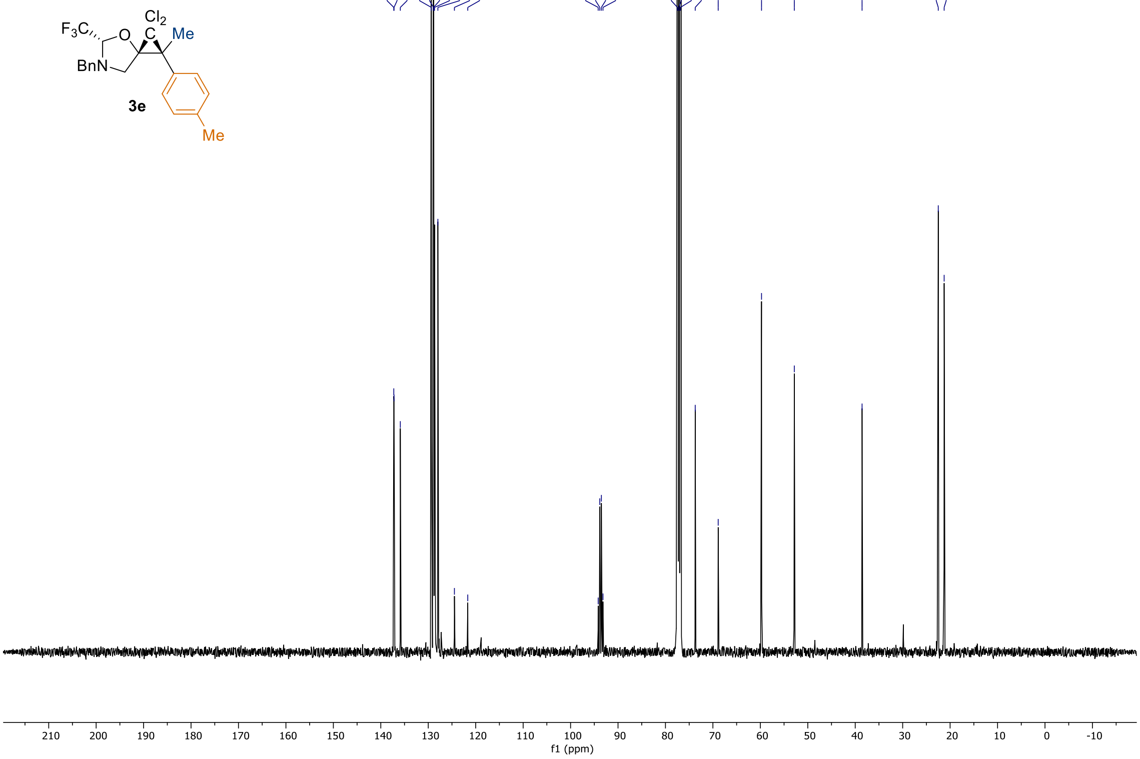

1H NMR (400 MHz, CDCl3)

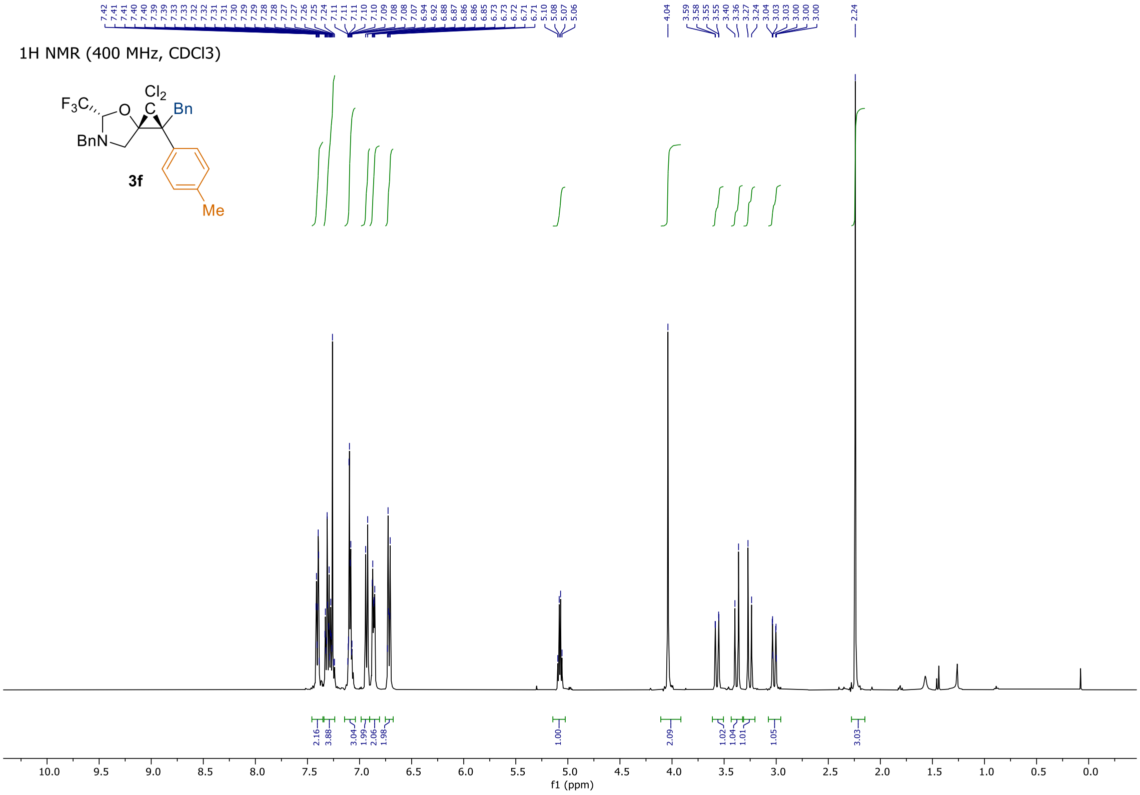

19F NMR (376 MHz, CDCl3)

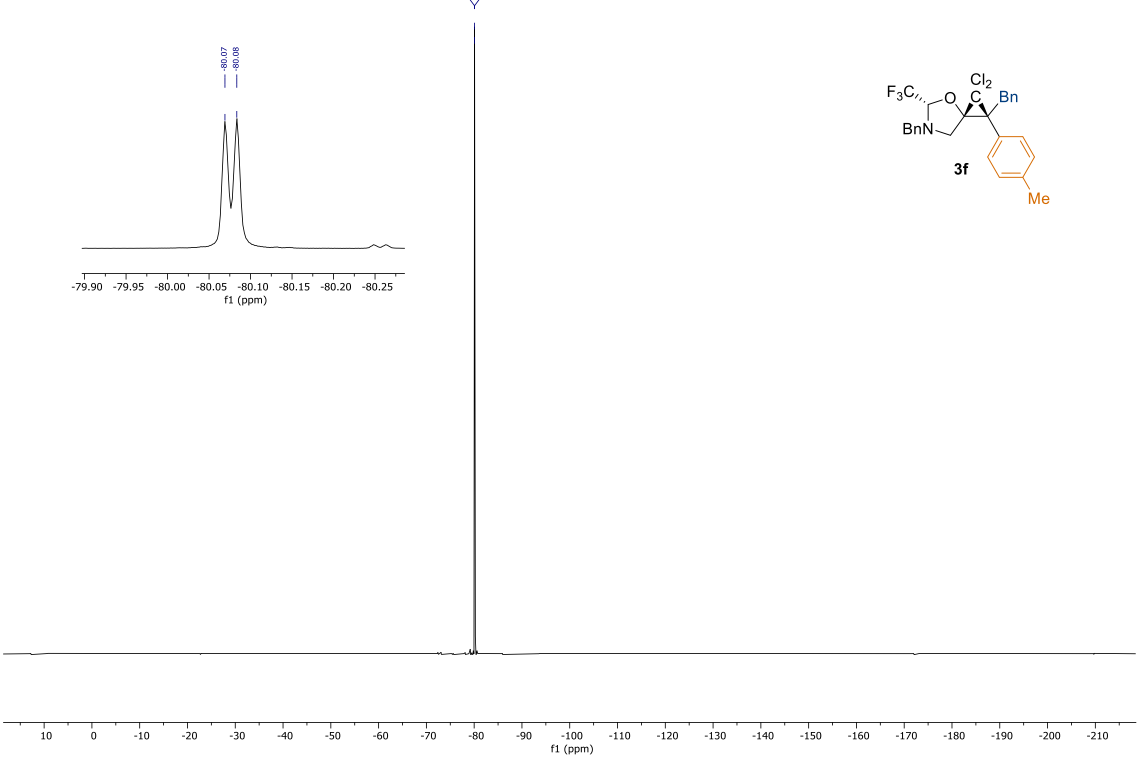

13C{1H} NMR (101 MHz, CDCl3)

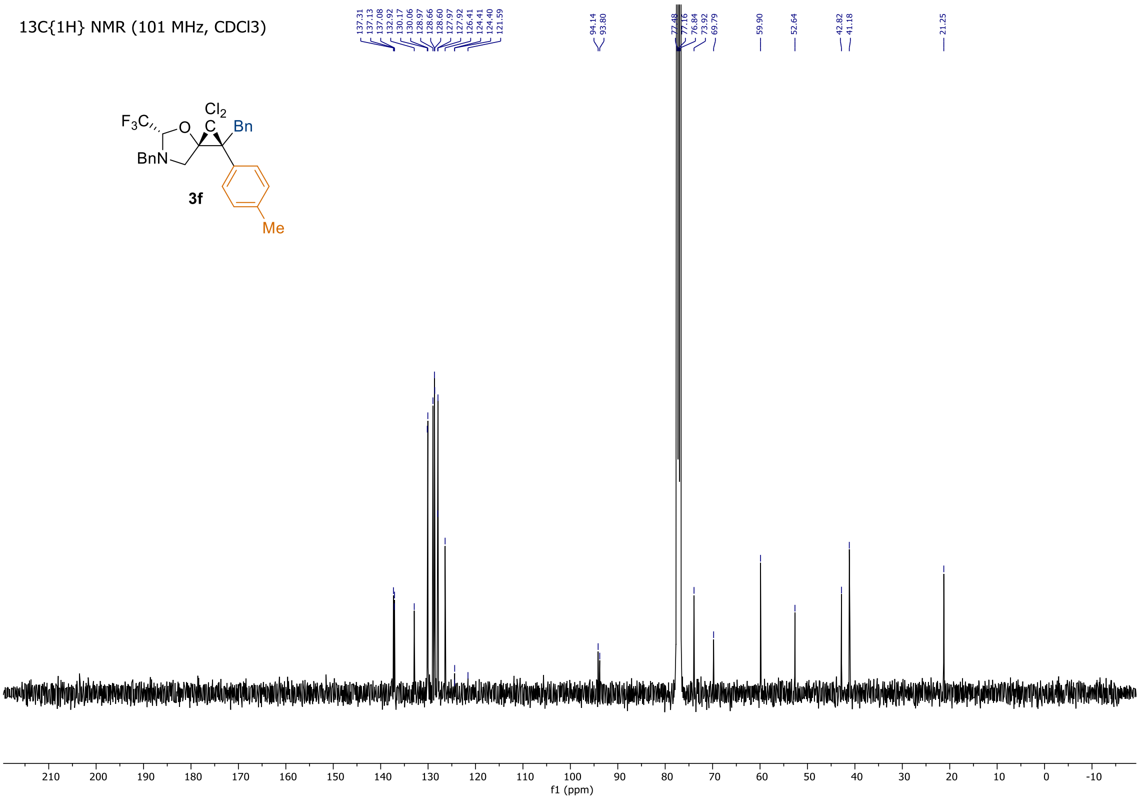

1H NMR (400 MHz, CDCl3)

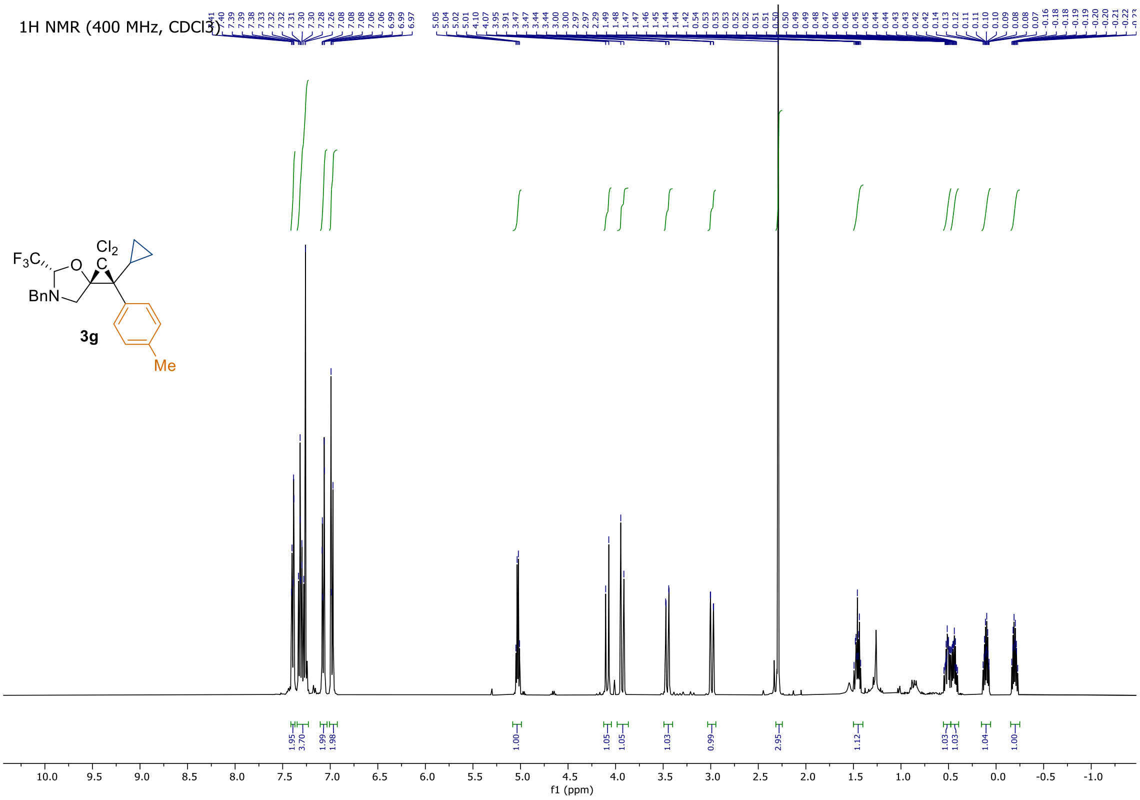

19F NMR (376 MHz, CDCl3)

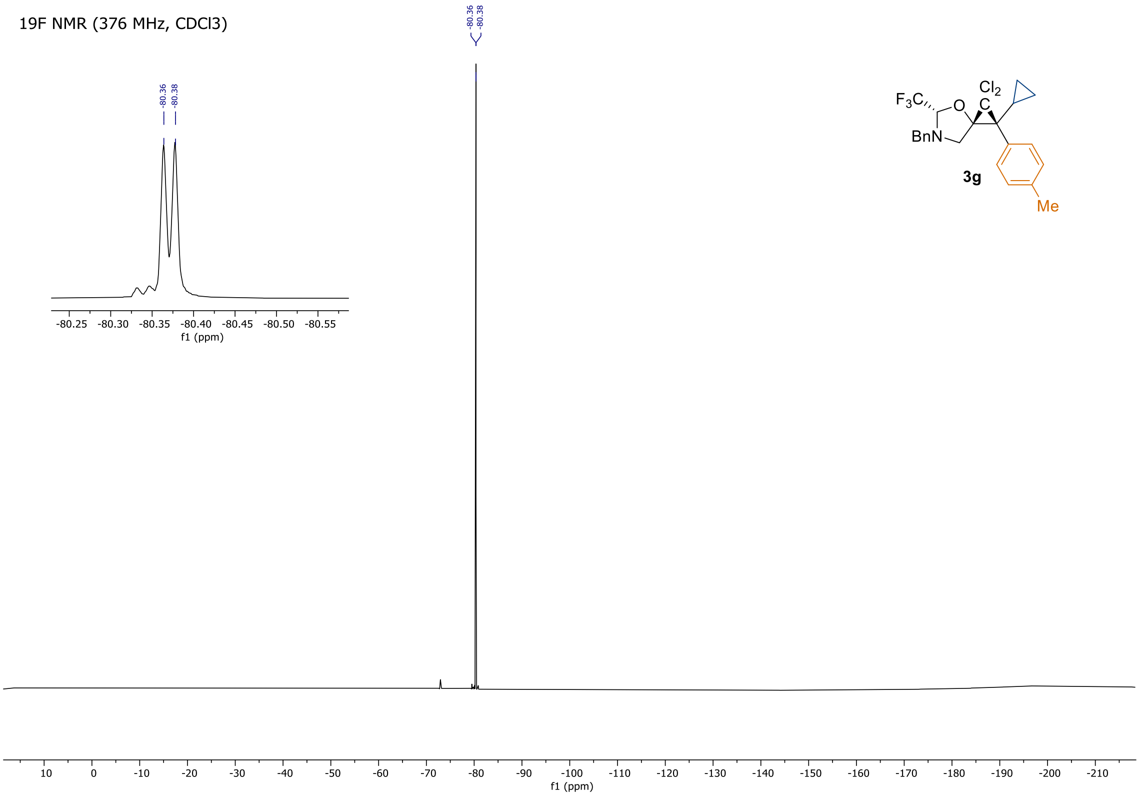

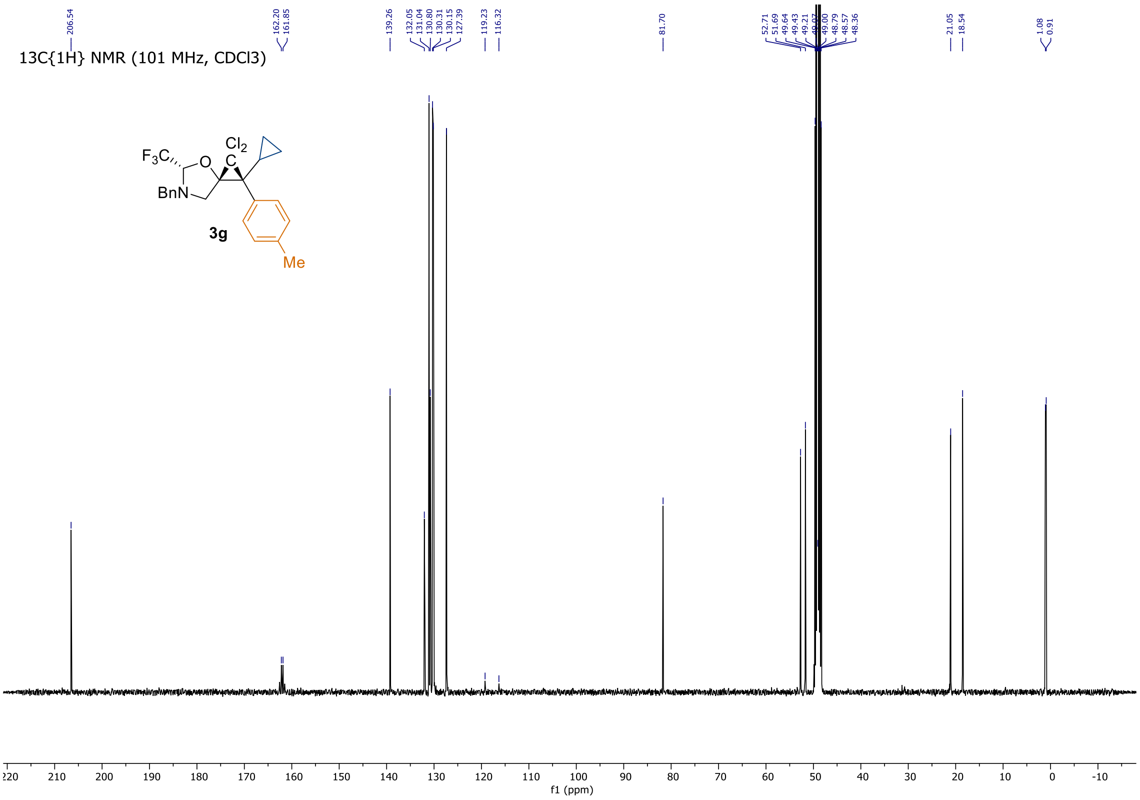
13C{1H} NMR (101 MHz, CDCl3)
F3C
O
Cl2
C
BnN
3g
Me
f1 (ppm)

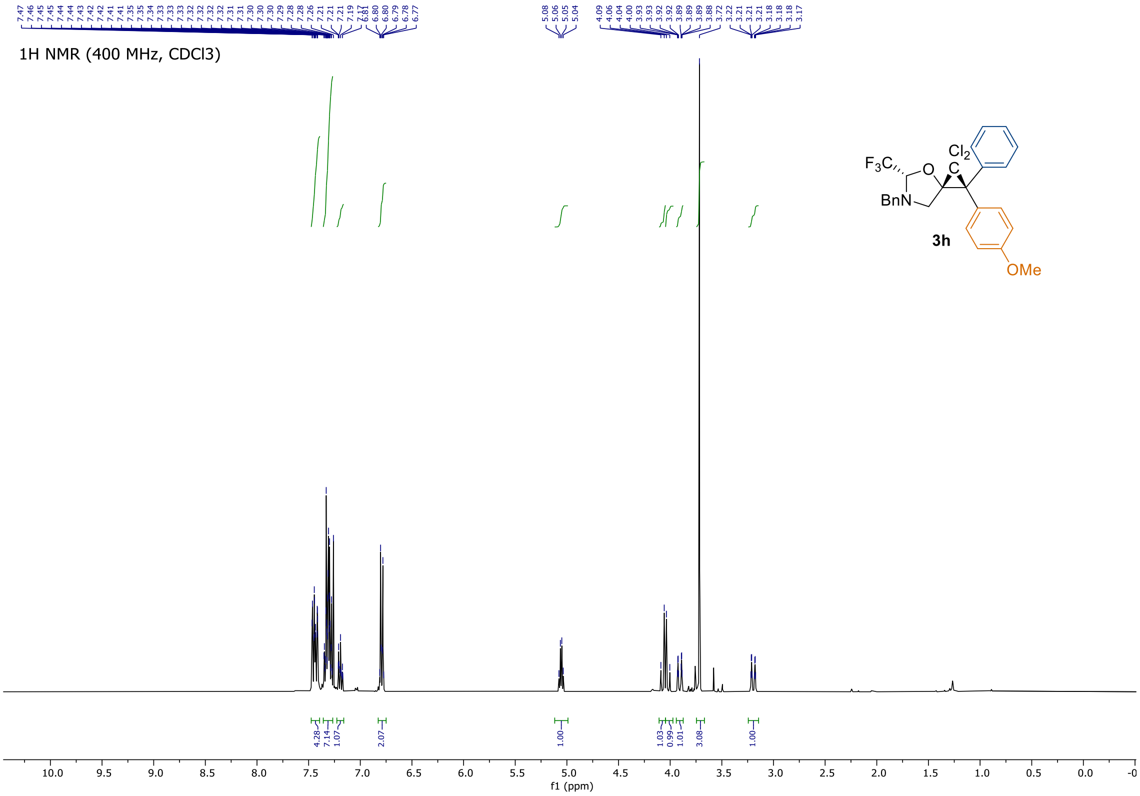
1H NMR (400 MHz, CDCl3)
F3C
O
Cl2
C
BnN
3h
OMe
f1 (ppm)

19F NMR (376 MHz, CDCl3)

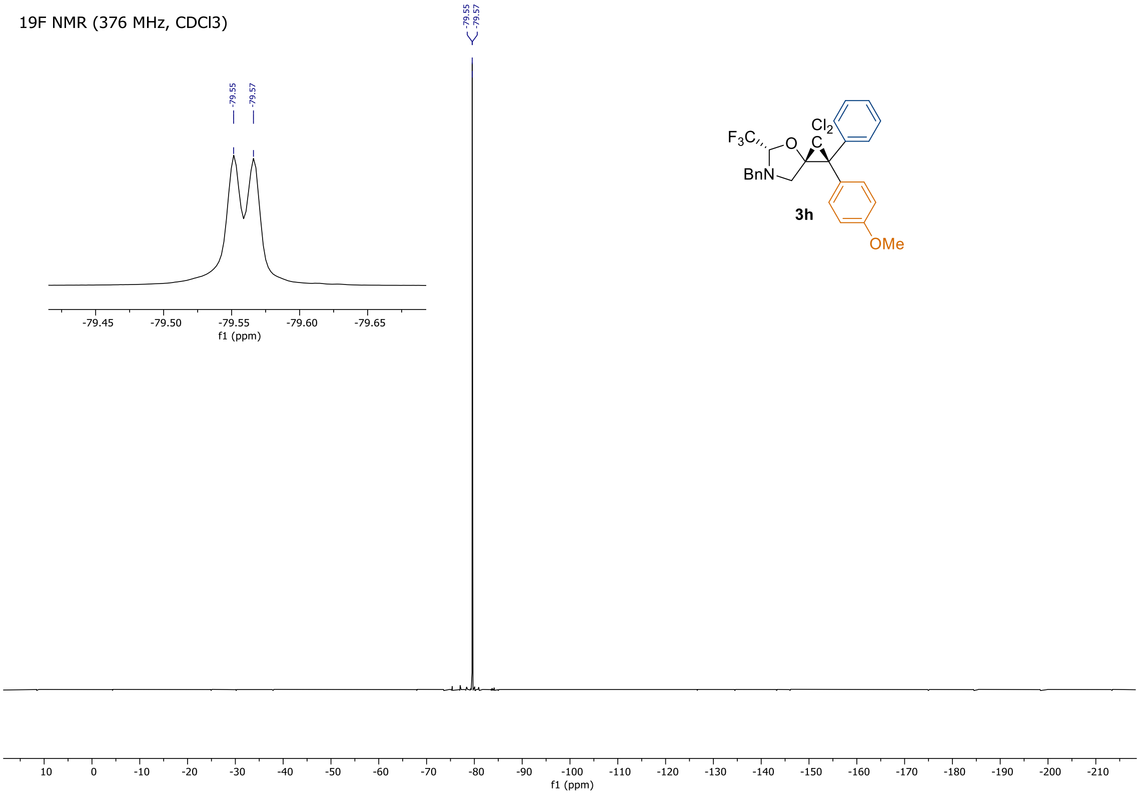

13C{1H} NMR (101 MHz, CDCl3)

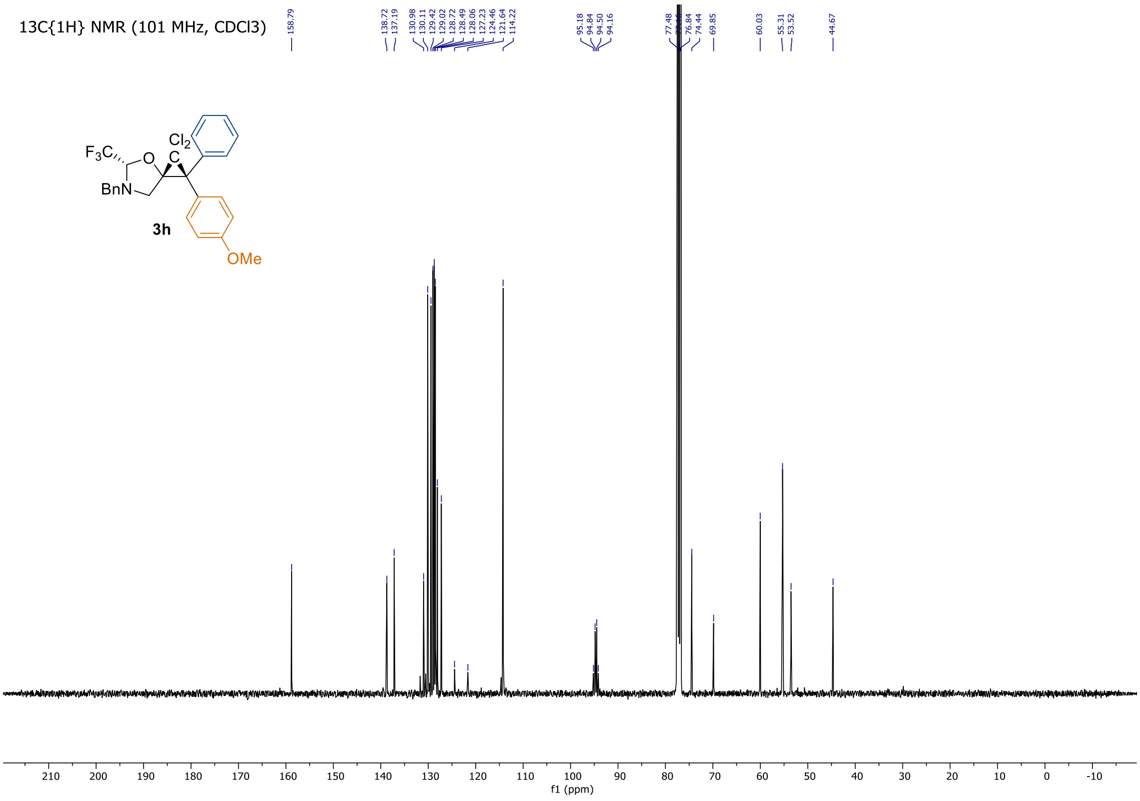

1H NMR (400 MHz, CDCl3)

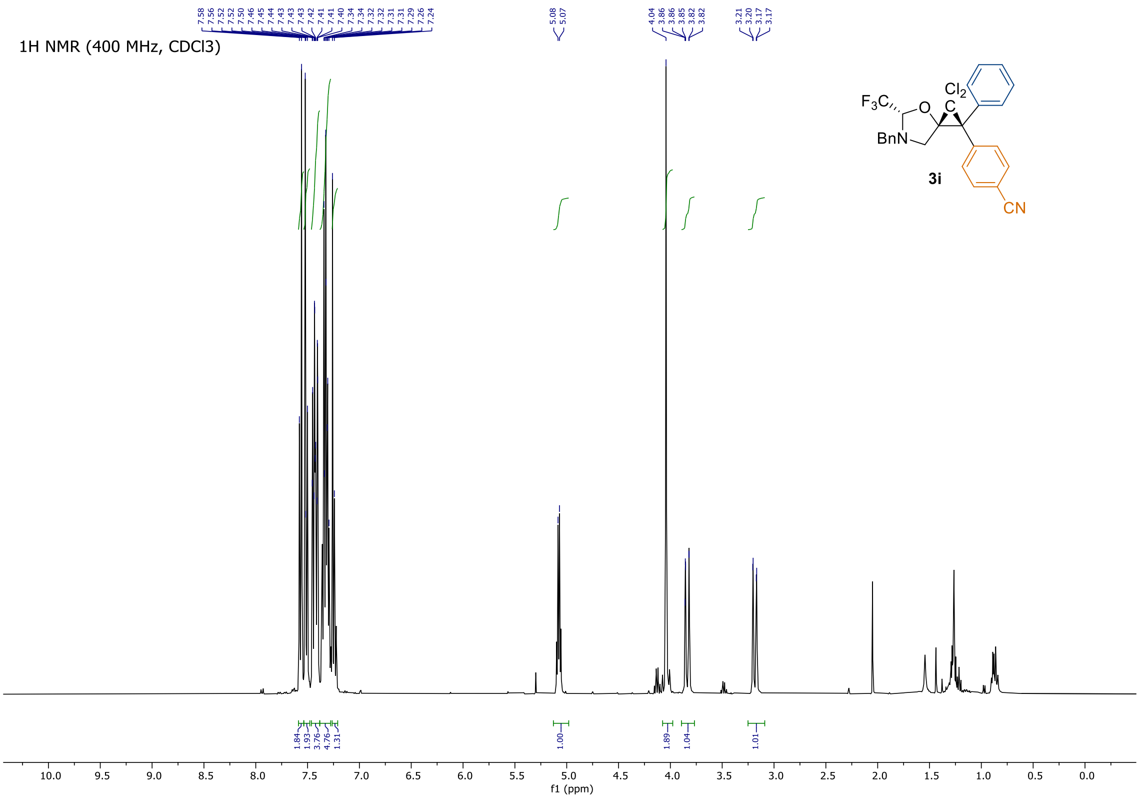

19F NMR (376 MHz, CDCl3)

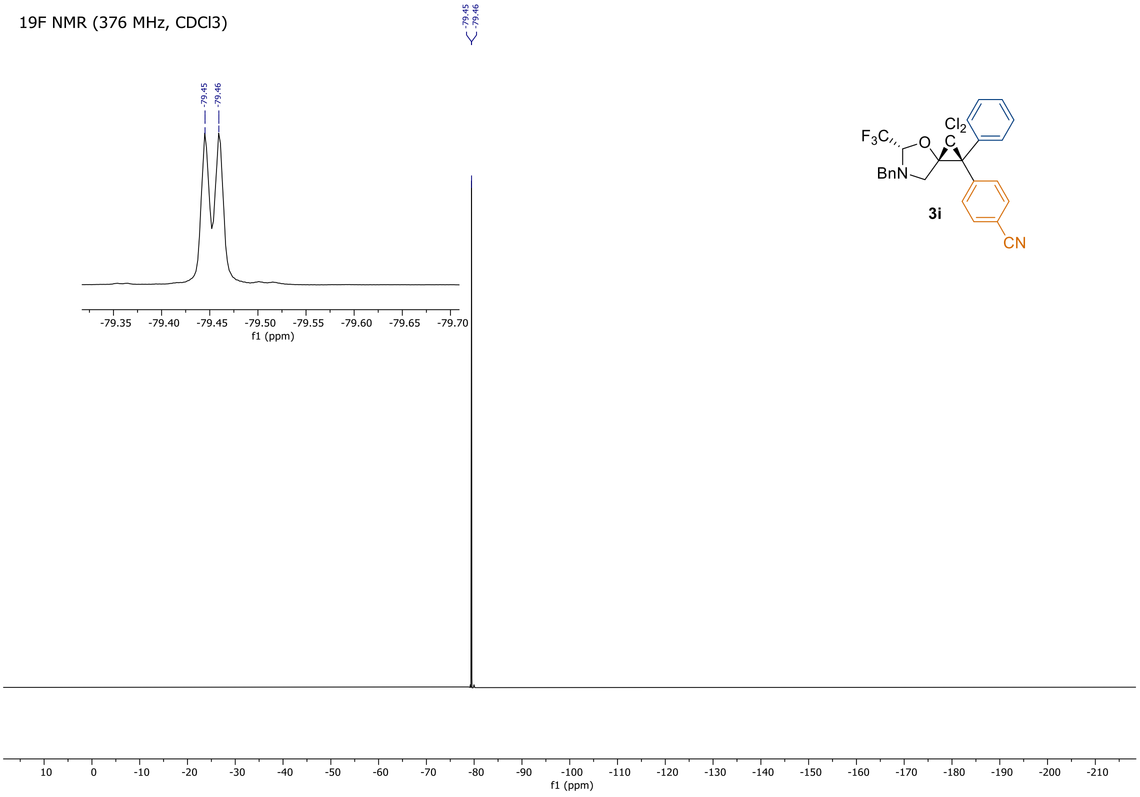

13C{1H} NMR (101 MHz, CDCl3)

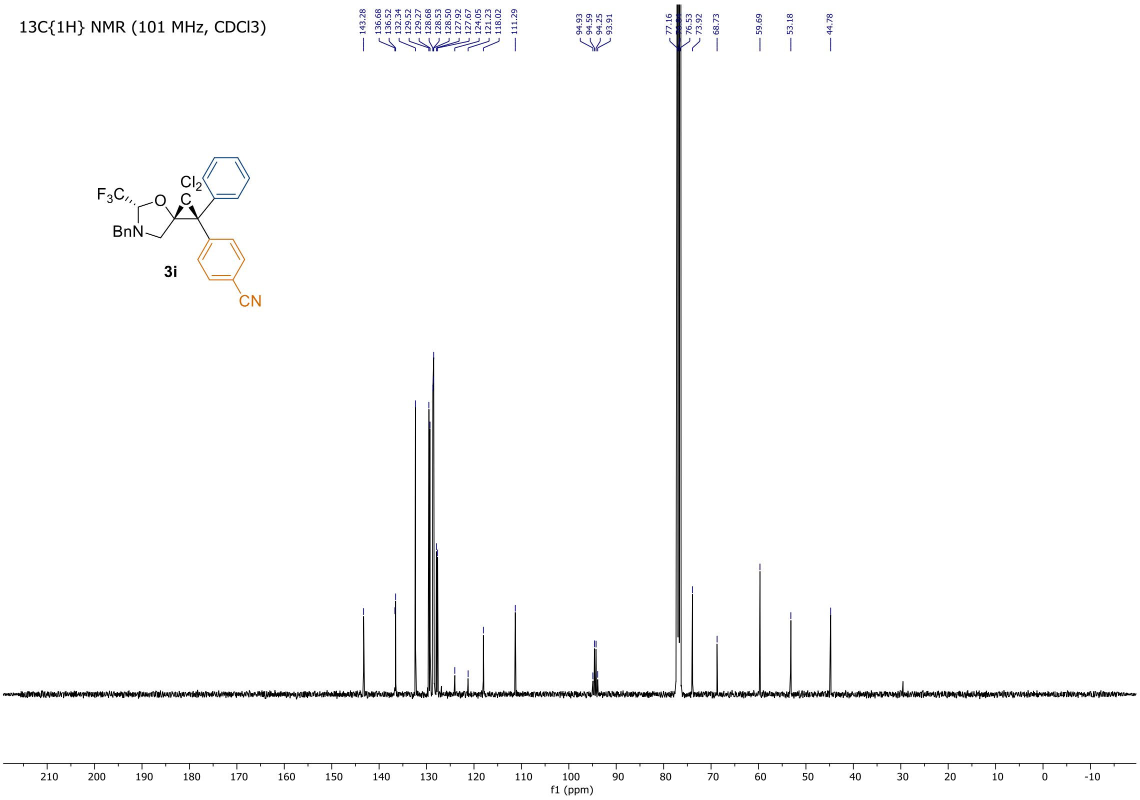

1H NMR (400 MHz, CDCl3)

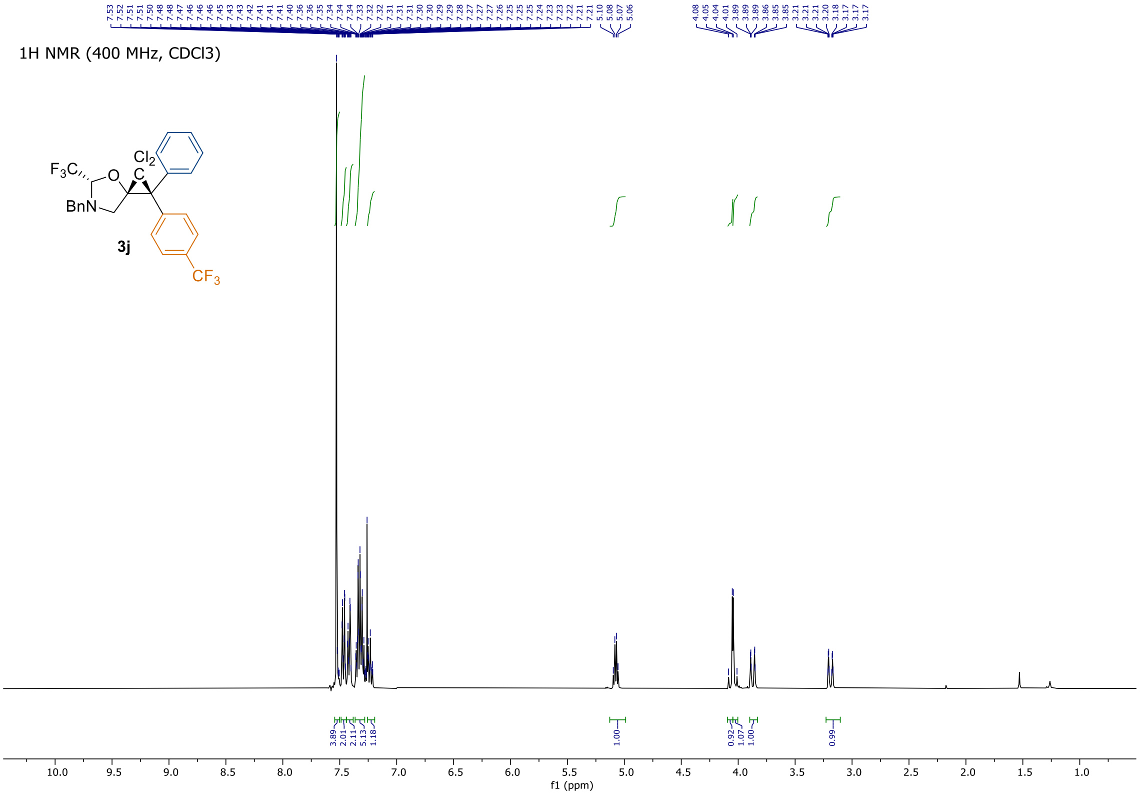

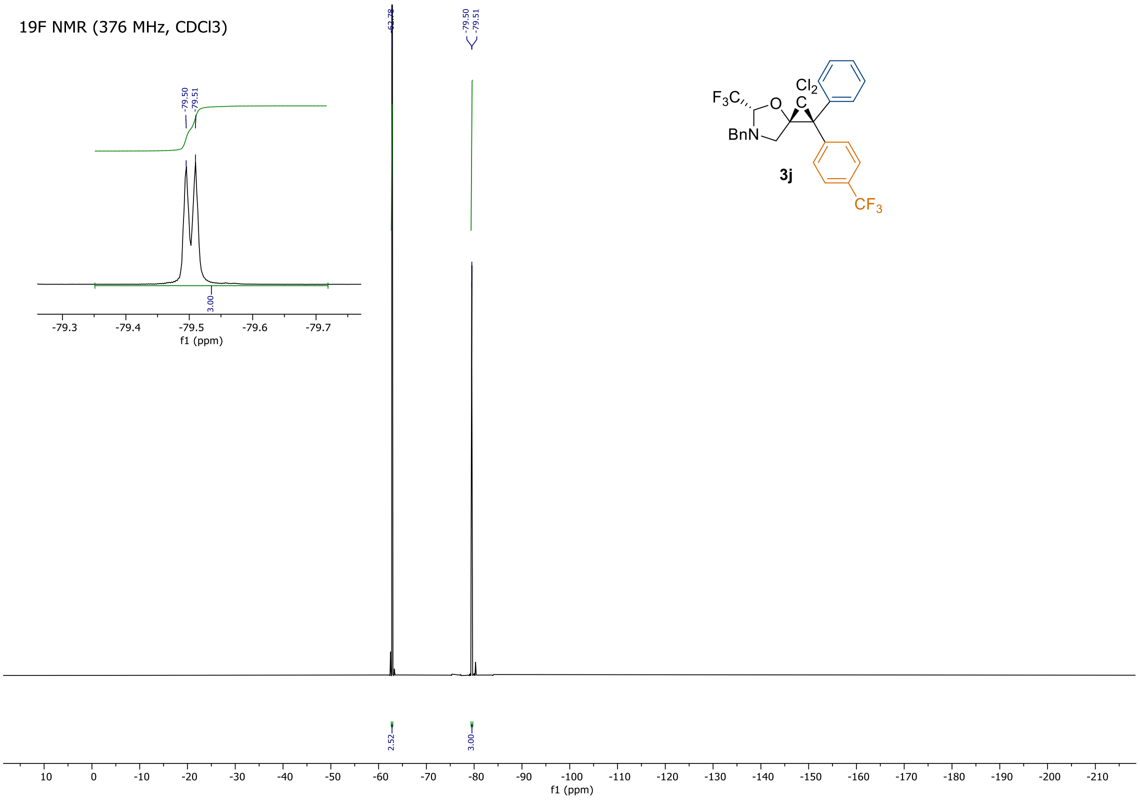
19F NMR (376 MHz, CDCl3)
-62.78
-79.50
-79.51
3.00
-79.3
-79.4
-79.5
-79.6
-79.7
f1 (ppm)
F3C
O
Cl2
C
BnN
3j
CF3
2.52
3.00
f1 (ppm)

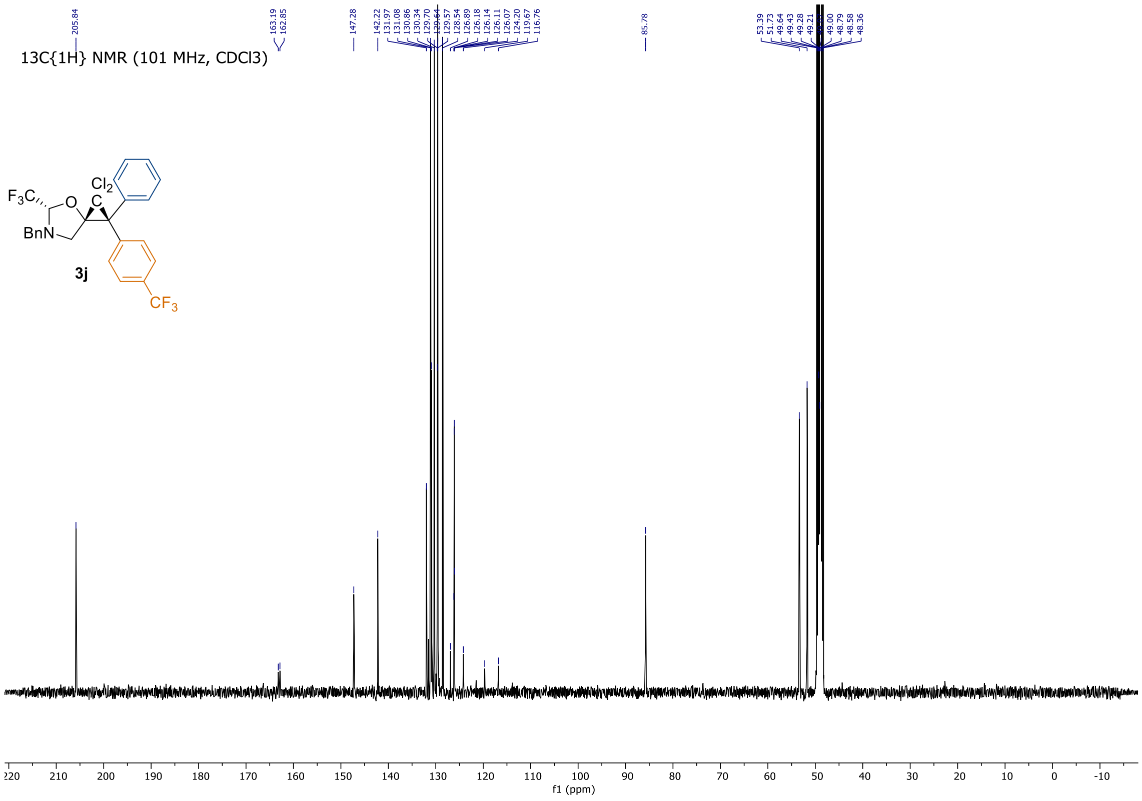
13C{1H} NMR (101 MHz, CDCl3)
205.84
163.19
162.85
147.28
142.22
131.97
131.08
130.86
130.34
129.70
129.57
128.54
126.89
126.18
126.14
126.11
126.07
124.20
119.67
116.76
85.78
53.39
51.73
49.64
49.43
49.28
49.21
49.00
48.79
48.58
48.36
F3C
O
Cl2
C
BnN
3j
CF3
f1 (ppm)

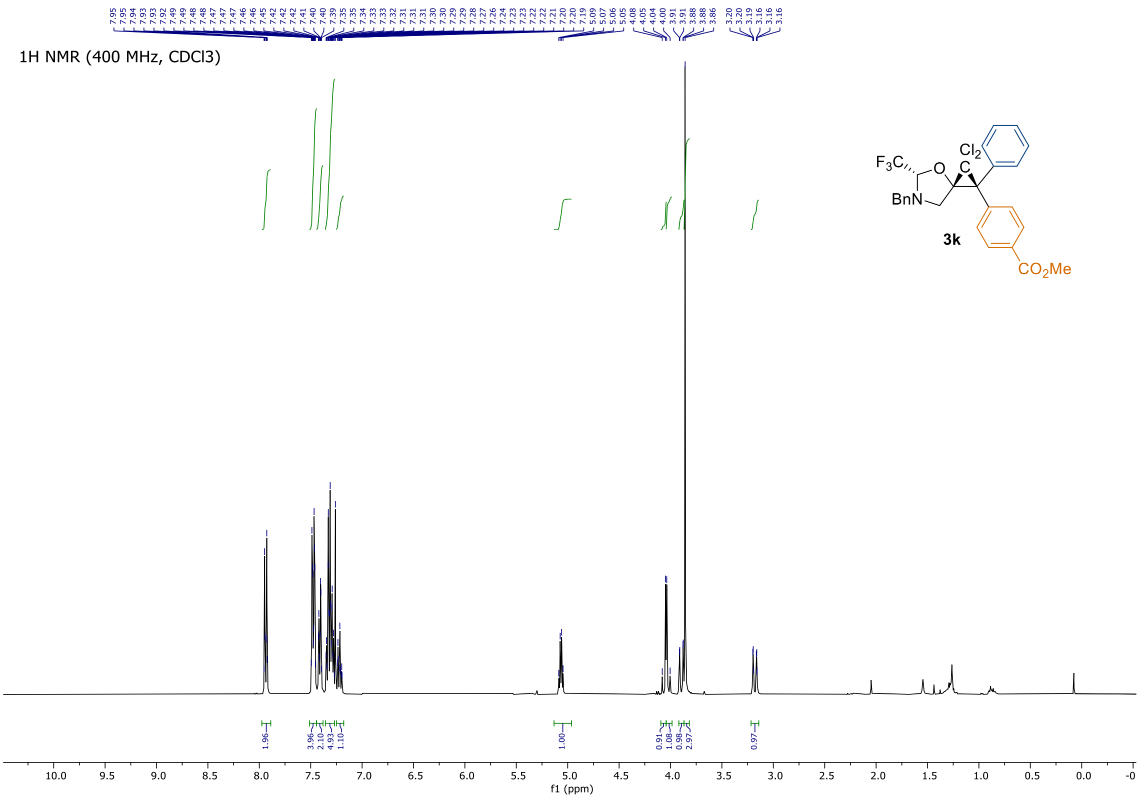
1H NMR (400 MHz, CDCl3)
3k
f1 (ppm)

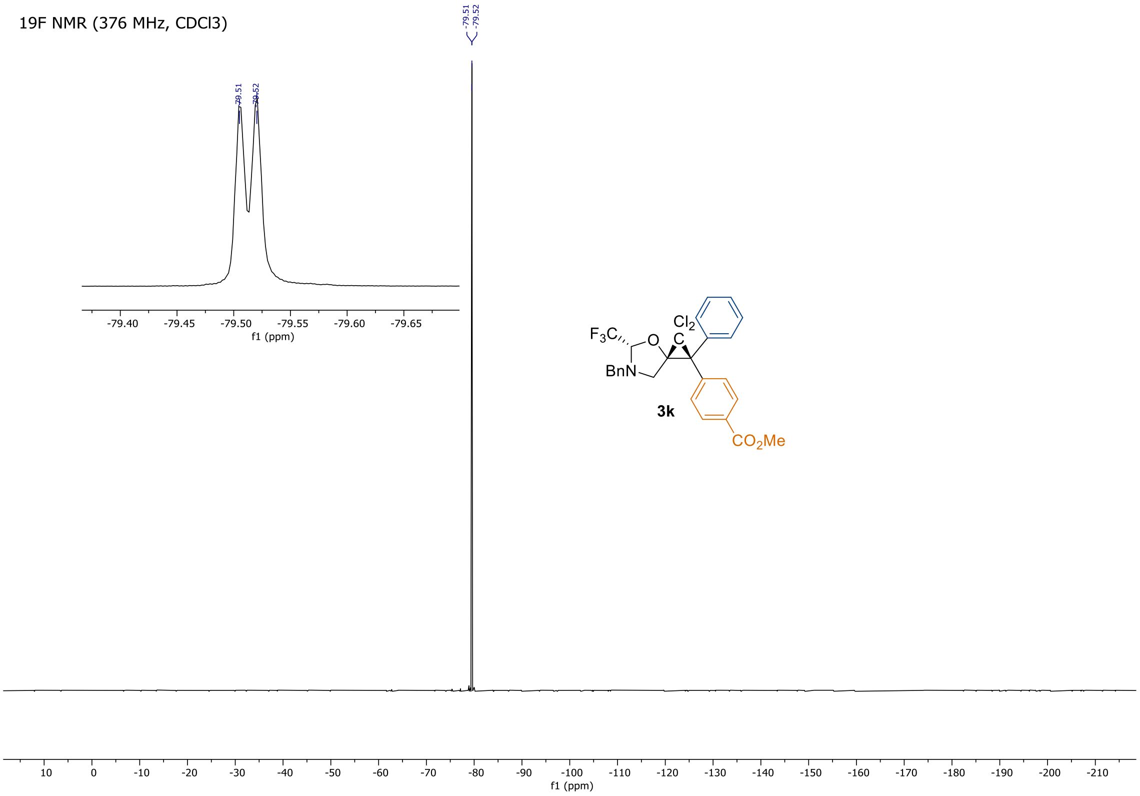
19F NMR (376 MHz, CDCl3)
3k
f1 (ppm)

13C{1H} NMR (101 MHz, CDCl3)

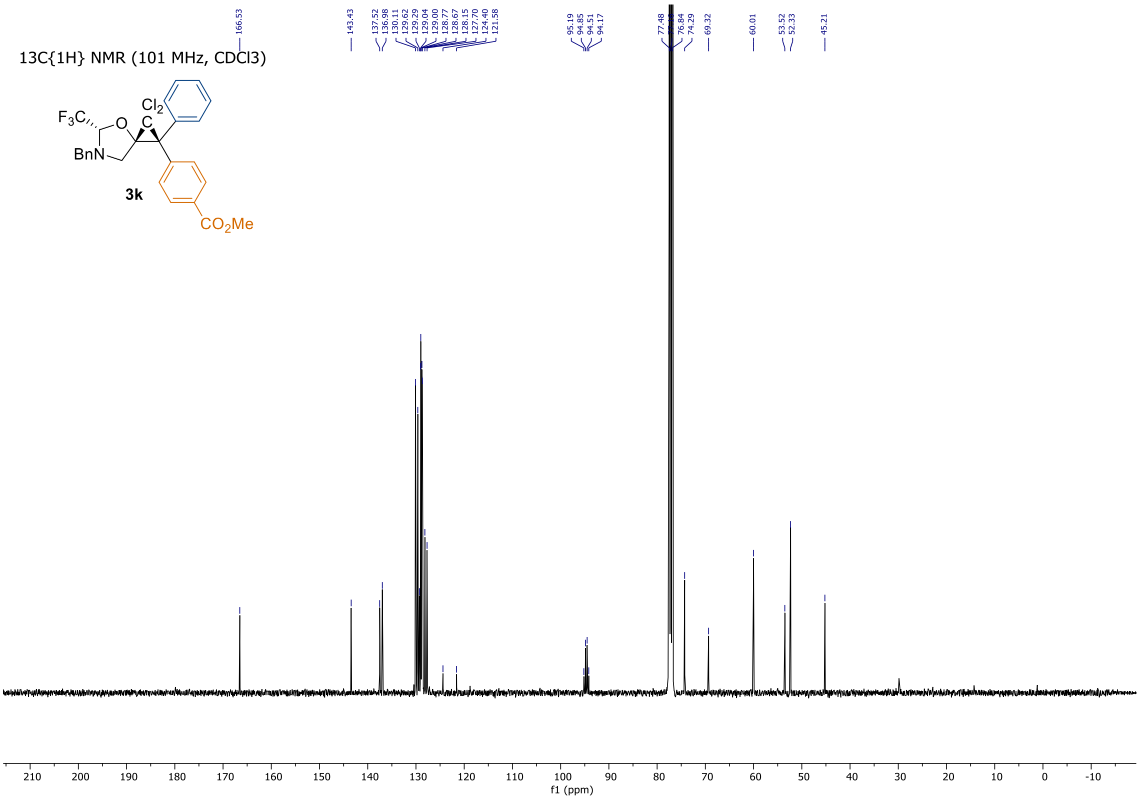

1H NMR (400 MHz, CDCl3)

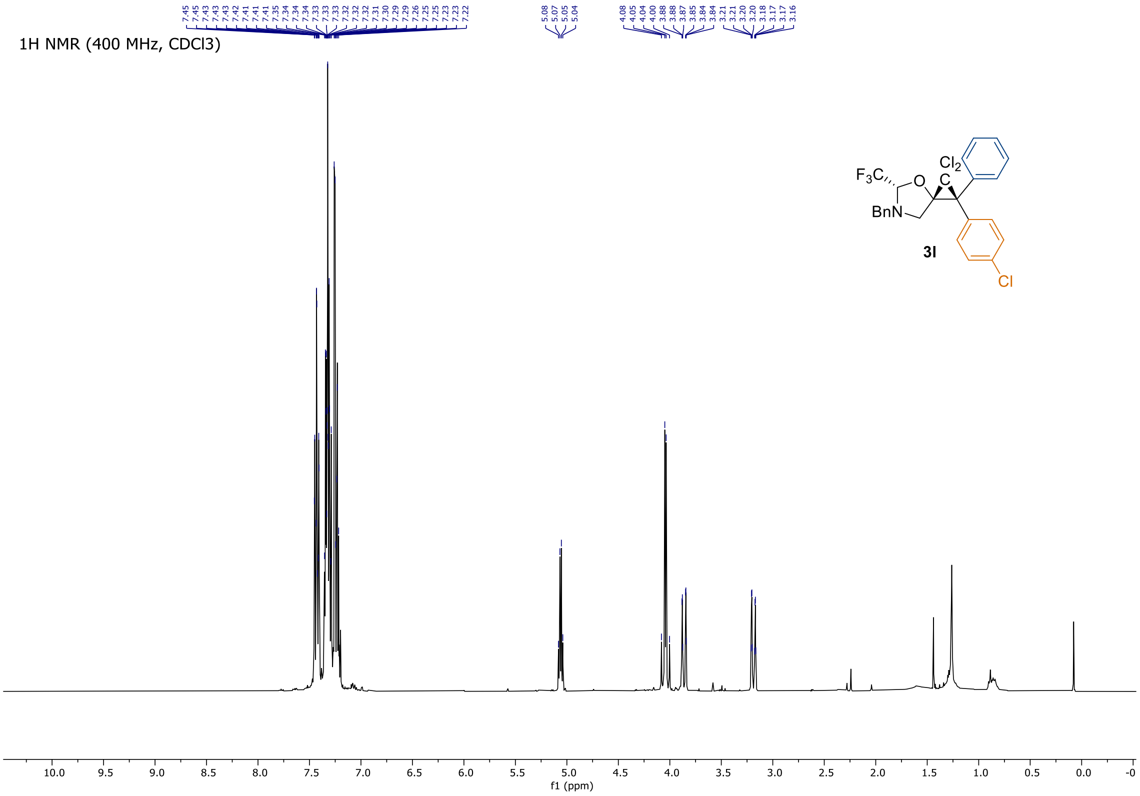

19F NMR (376 MHz, CDCl3)

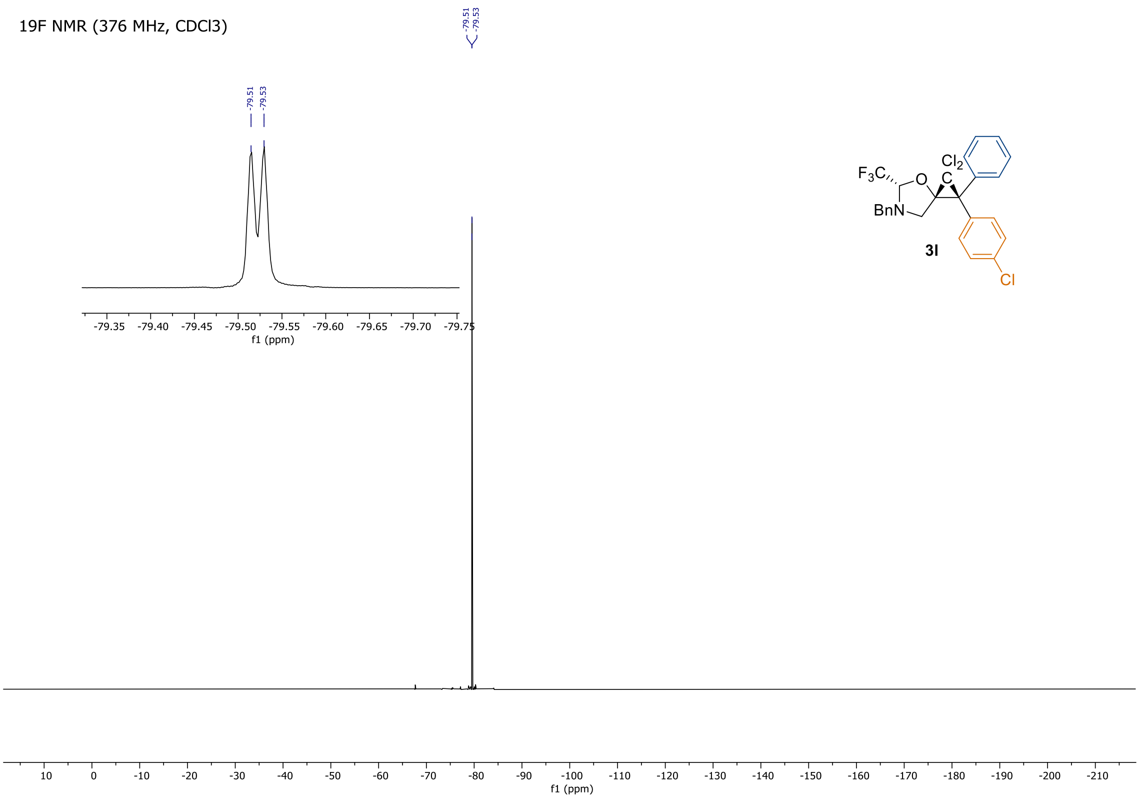

13C{1H} NMR (101 MHz, CDCl3)

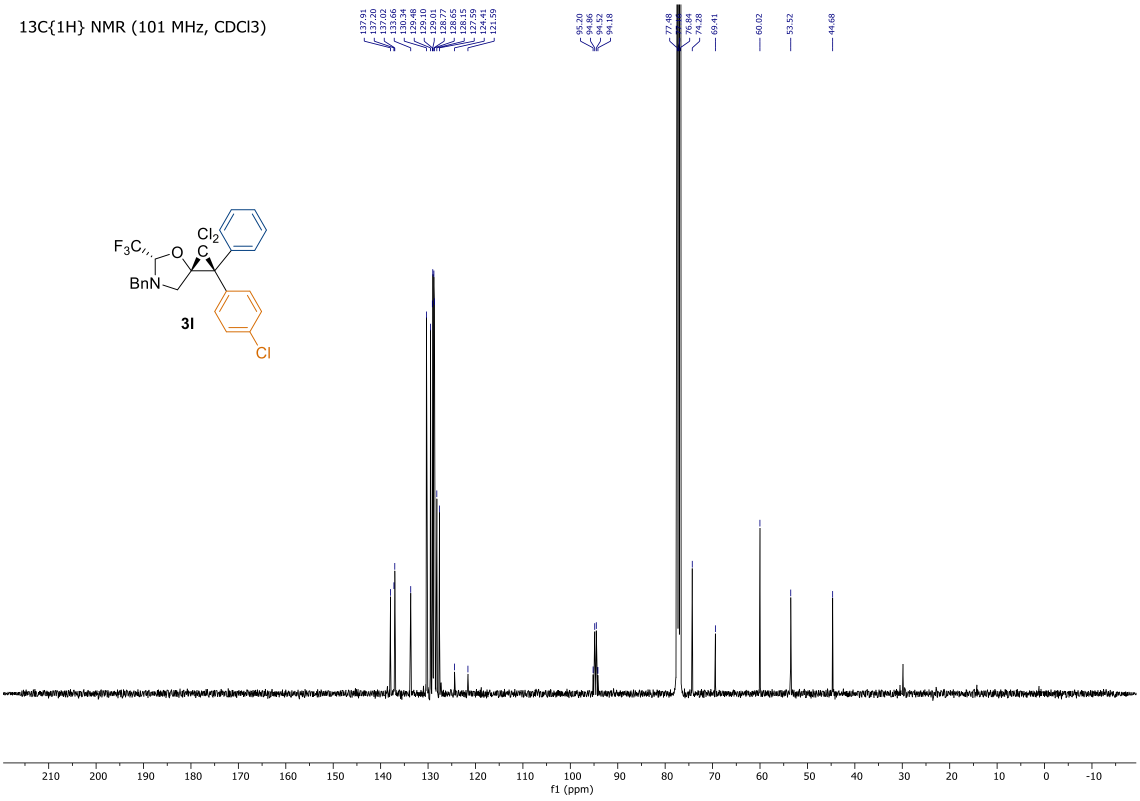

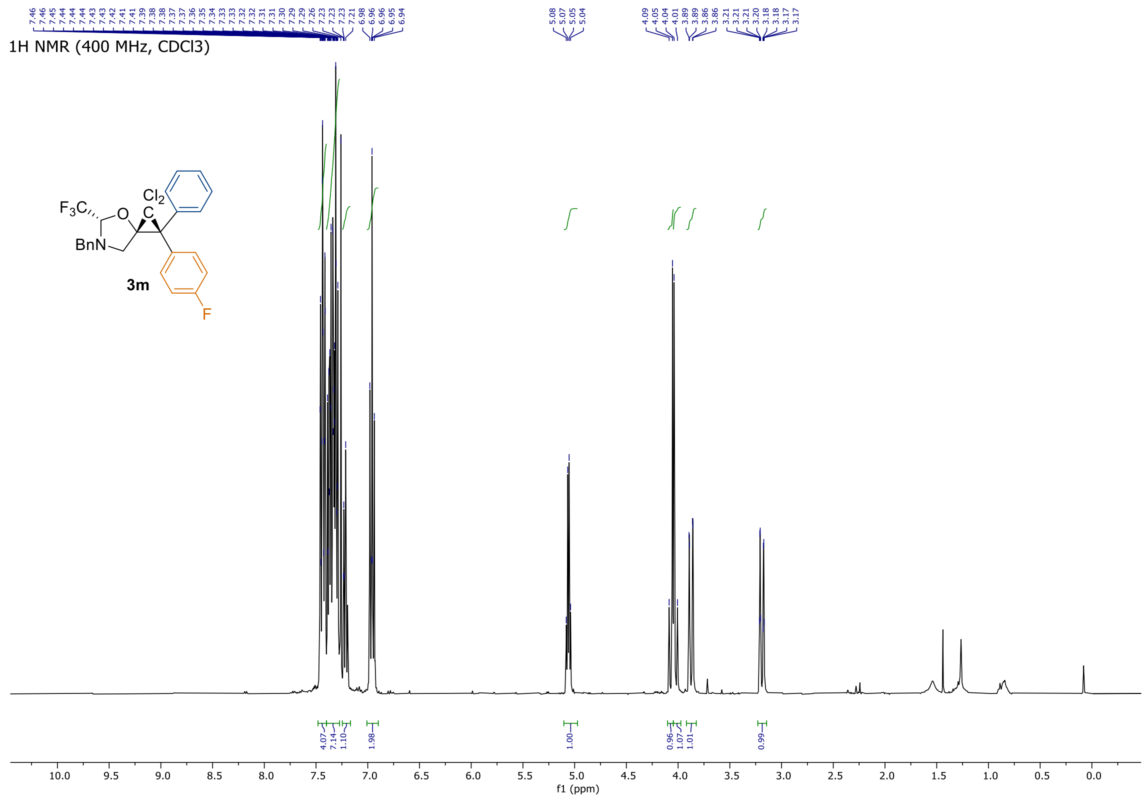
1H NMR (400 MHz, CDCl3)
3m
f1 (ppm)

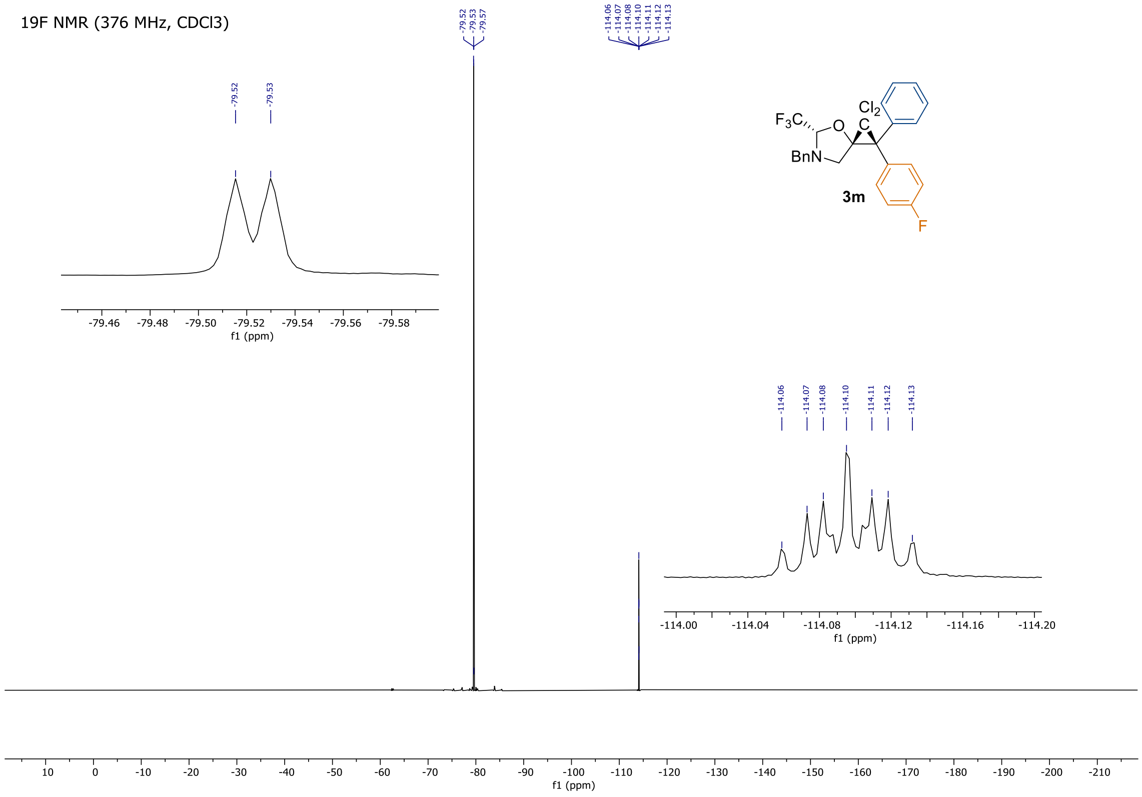
19F NMR (376 MHz, CDCl3)
3m
f1 (ppm)

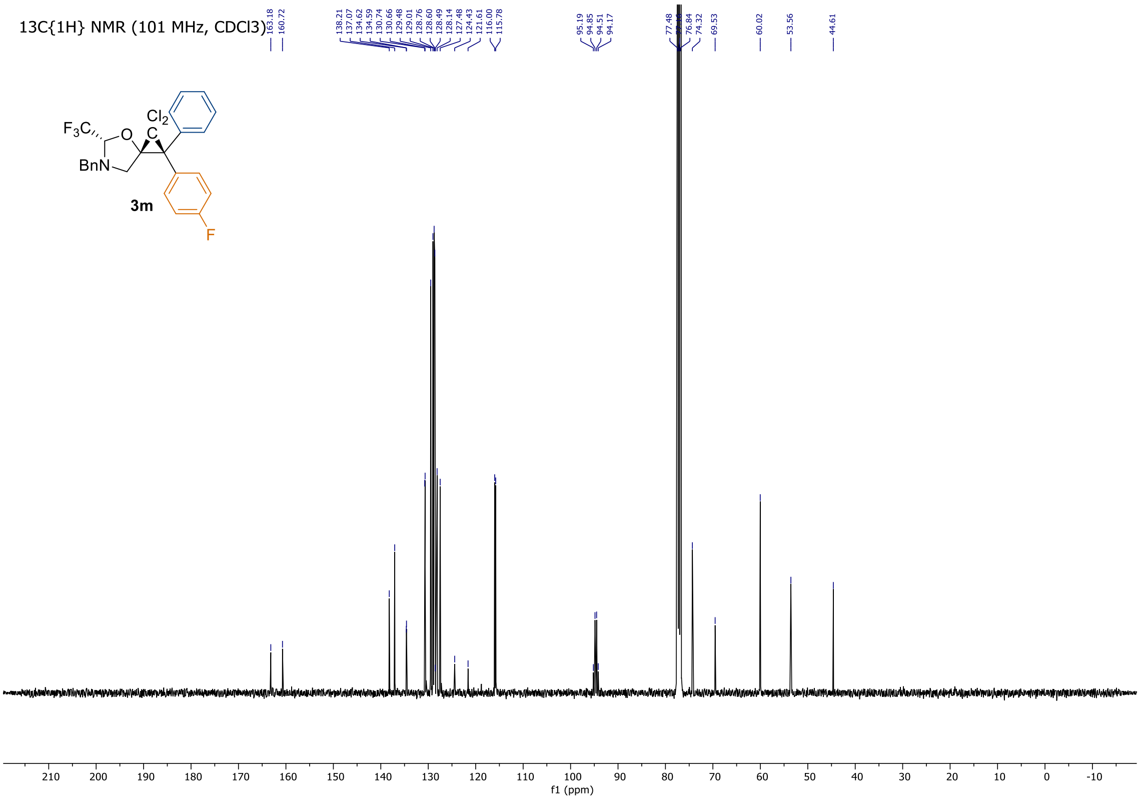
13C{1H} NMR (101 MHz, CDCl3)
163.18
160.72
138.21
137.07
134.62
134.59
130.74
130.66
129.48
129.01
128.76
128.60
128.49
128.14
127.48
124.43
121.61
116.00
115.78
95.19
94.85
94.51
94.17
77.48
76.84
74.32
69.53
60.02
53.56
44.61
F_3C
O
Cl_2
C
BnN
3m
F
210 200 190 180 170 160 150 140 130 120 110 100 90 80 70 60 50 40 30 20 10 0 -10
f1 (ppm)

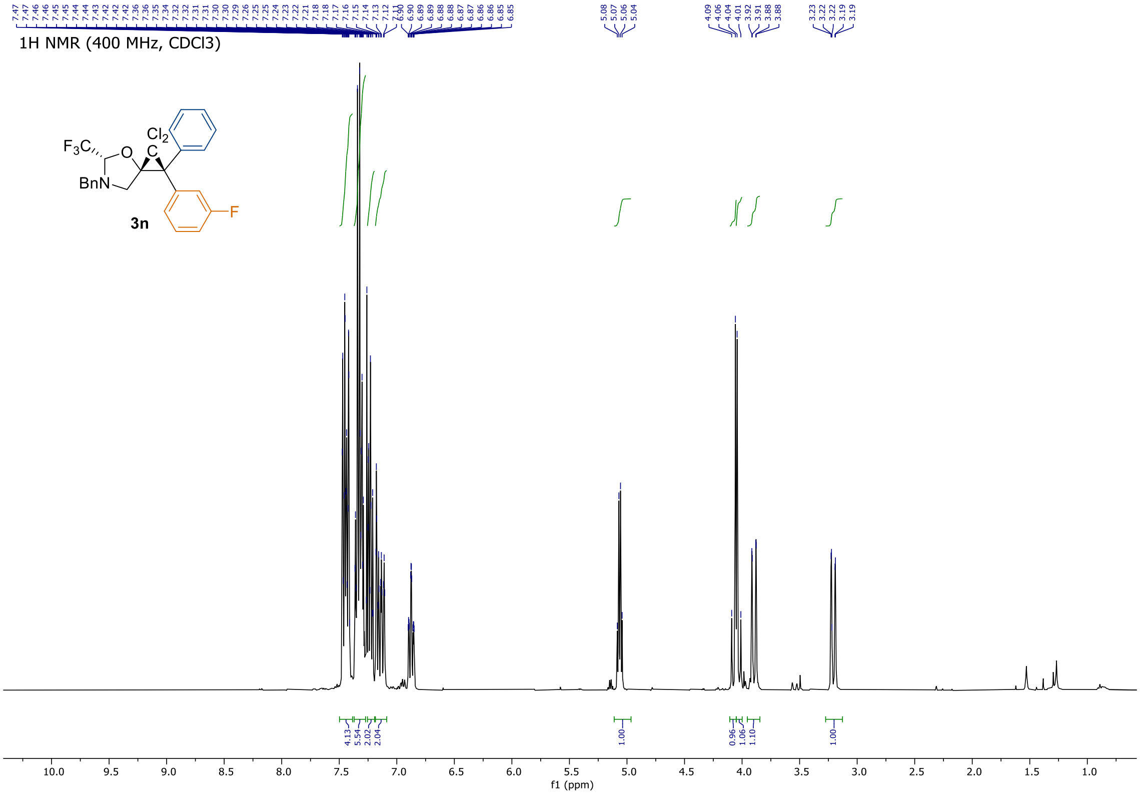
1H NMR (400 MHz, CDCl3)
7.47 7.47 7.46 7.46 7.45 7.45 7.44 7.44 7.43 7.42 7.42 7.42 7.36 7.36 7.35 7.34 7.32 7.32 7.31 7.31 7.30 7.30 7.29 7.26 7.25 7.25 7.24 7.23 7.22 7.21 7.18 7.18 7.17 7.16 7.15 7.14 7.13 7.12 7.11 6.90 6.90 6.89 6.89 6.88 6.88 6.87 6.87 6.86 6.86 6.85 6.85
5.08 5.07 5.06 5.04
4.09 4.06 4.04 4.01 3.92 3.91 3.88 3.88
3.23 3.22 3.22 3.19 3.19
F_3C
O
Cl_2
C
BnN
3n
F
4.13 5.54 2.02 2.04
1.00
0.96 1.06 1.10
1.00
10.0 9.5 9.0 8.5 8.0 7.5 7.0 6.5 6.0 5.5 5.0 4.5 4.0 3.5 3.0 2.5 2.0 1.5 1.0
f1 (ppm)

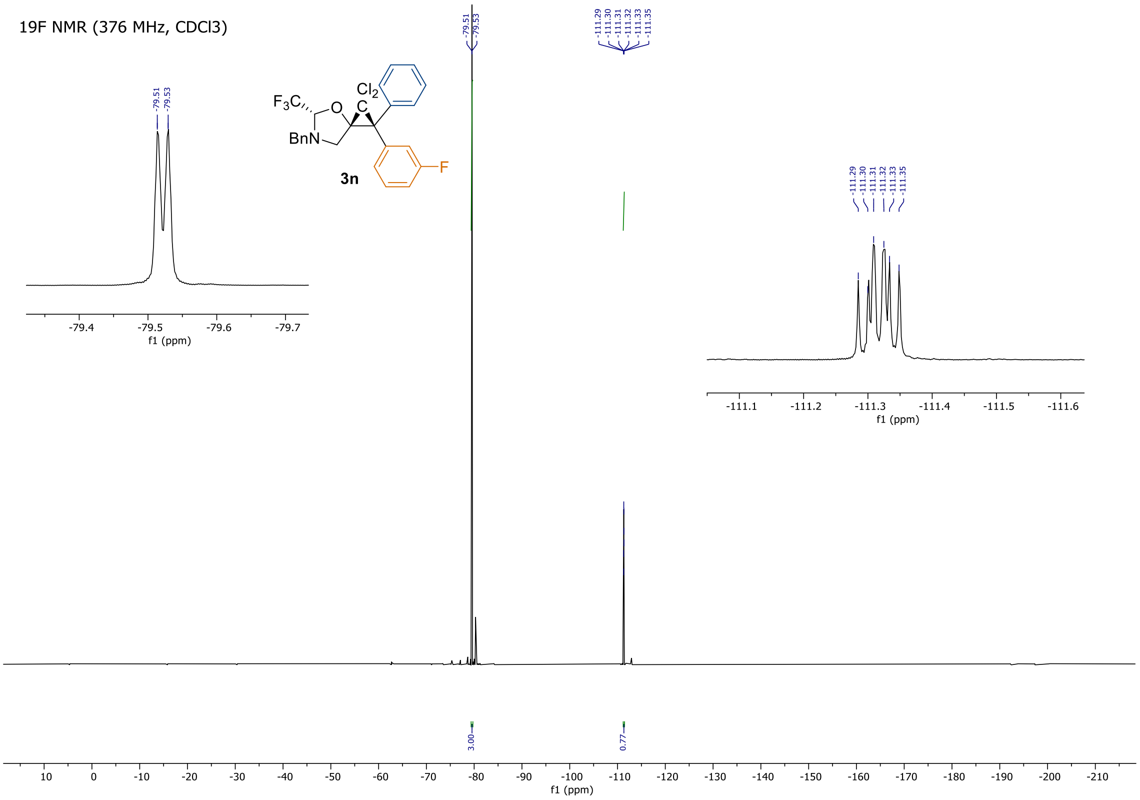
19F NMR (376 MHz, CDCl3)
3n
f1 (ppm)

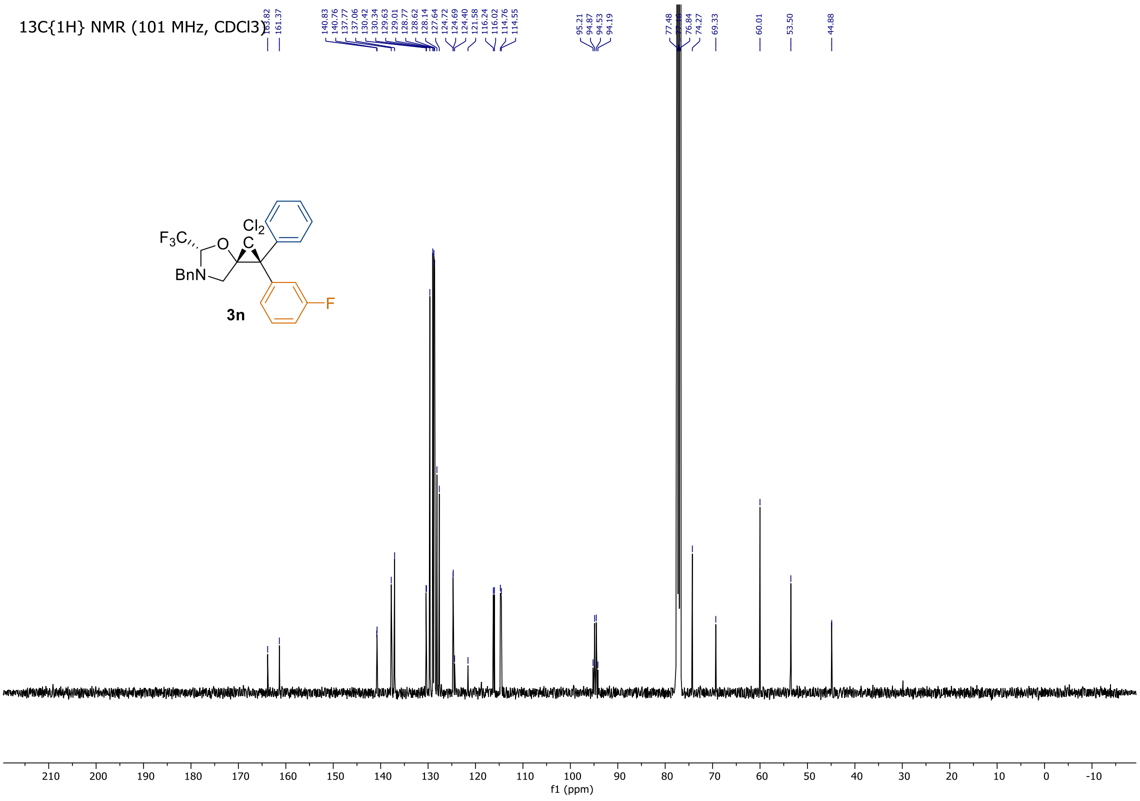
13C{1H} NMR (101 MHz, CDCl3)
3n
f1 (ppm)

G.2.Epoxidation products

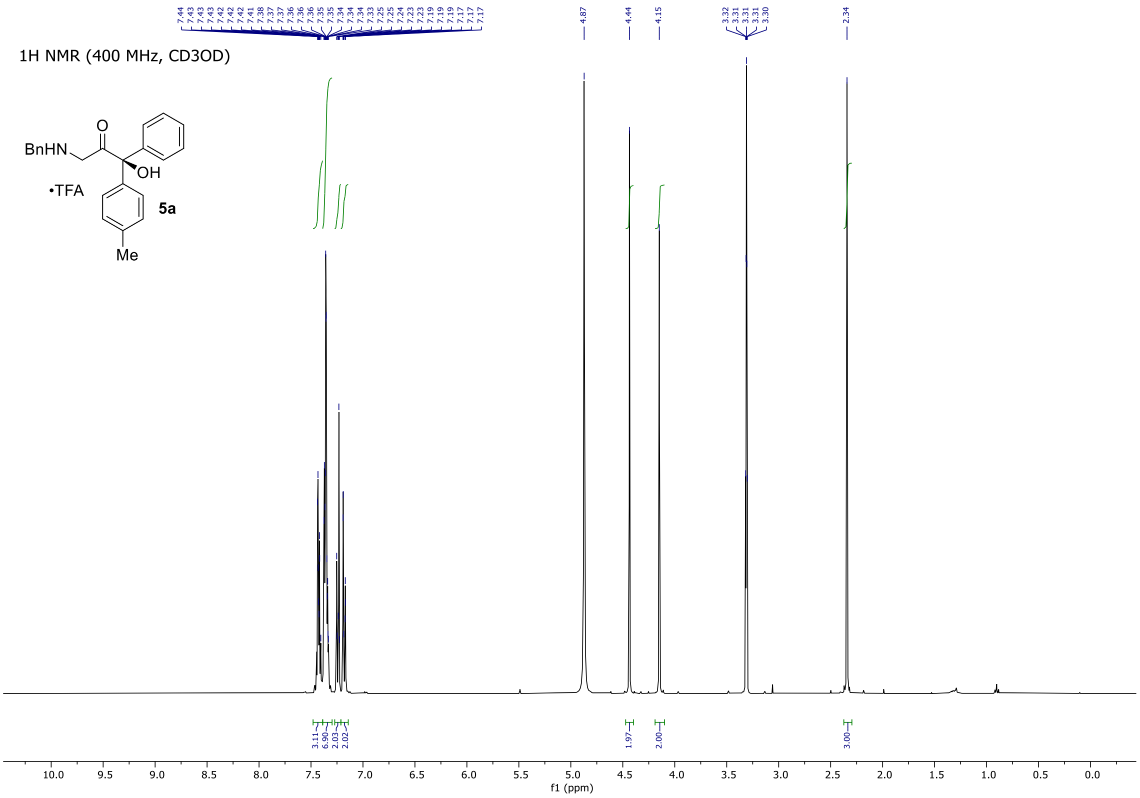

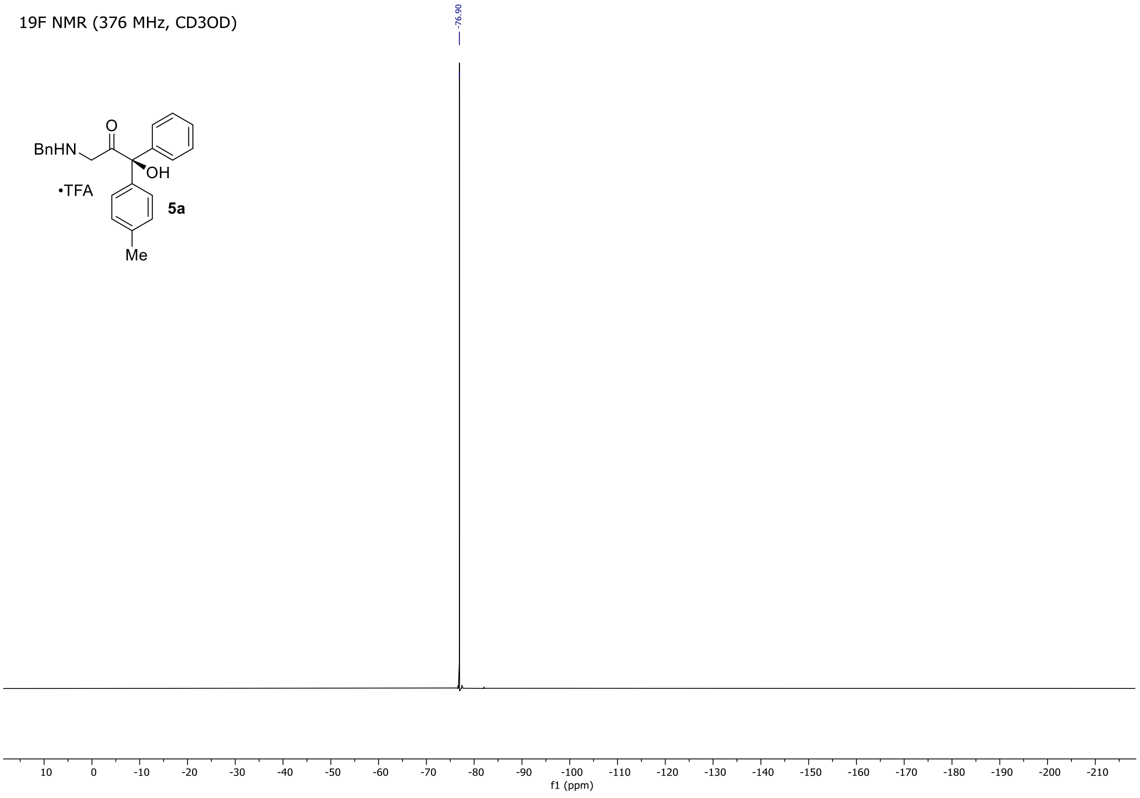

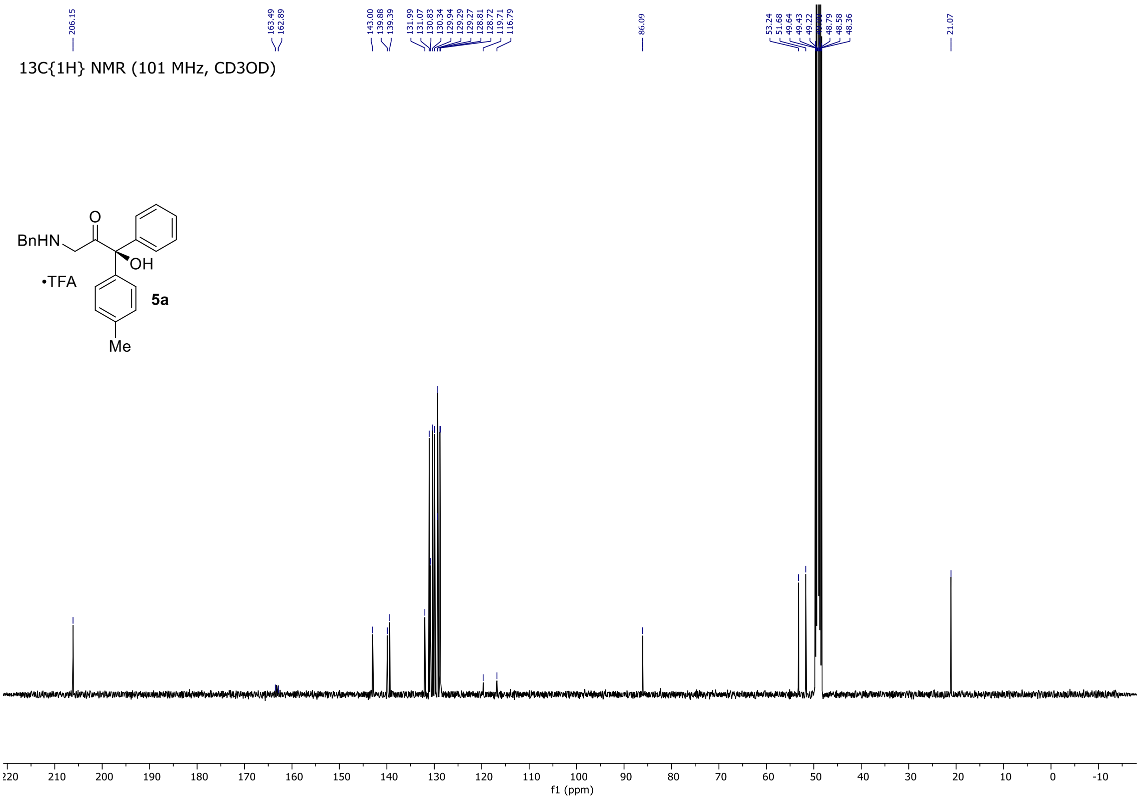
13C{1H} NMR (101 MHz, CD3OD)
BnHN
O
OH
•TFA
5a
Me
f1 (ppm)

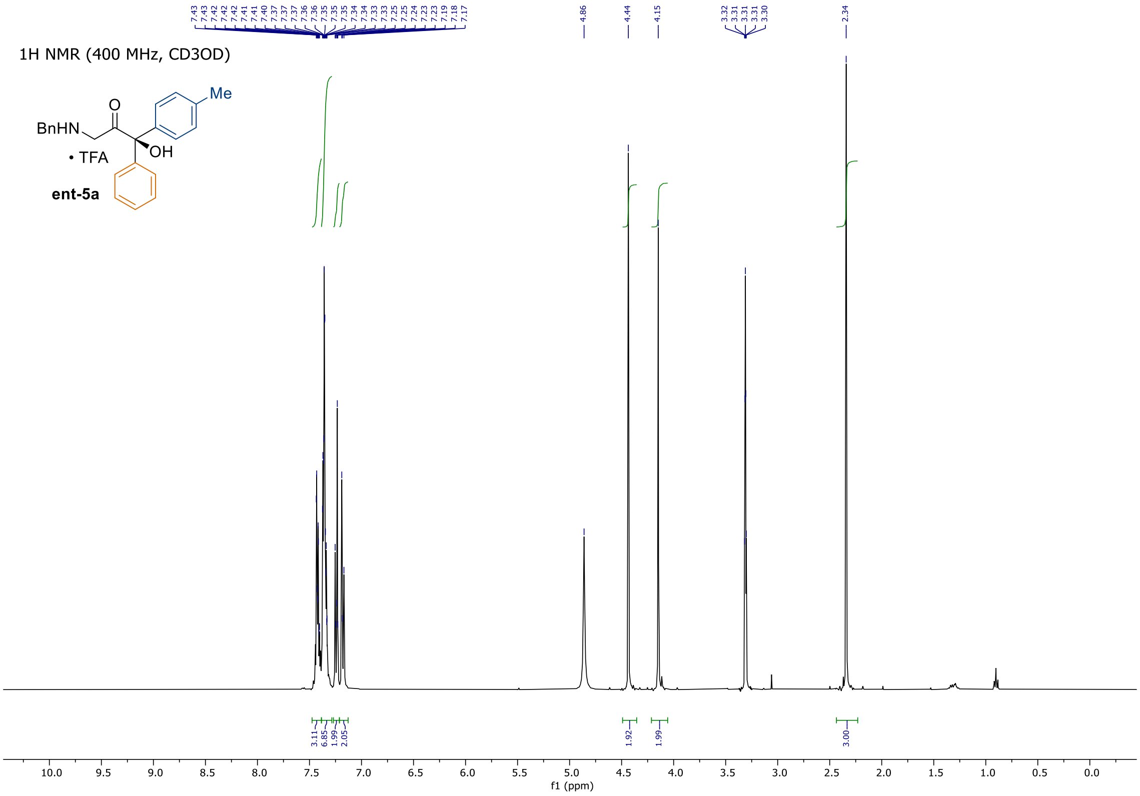
1H NMR (400 MHz, CD3OD)
BnHN
O
Me
OH
• TFA
ent-5a
f1 (ppm)

19F NMR (376 MHz, CD3OD)

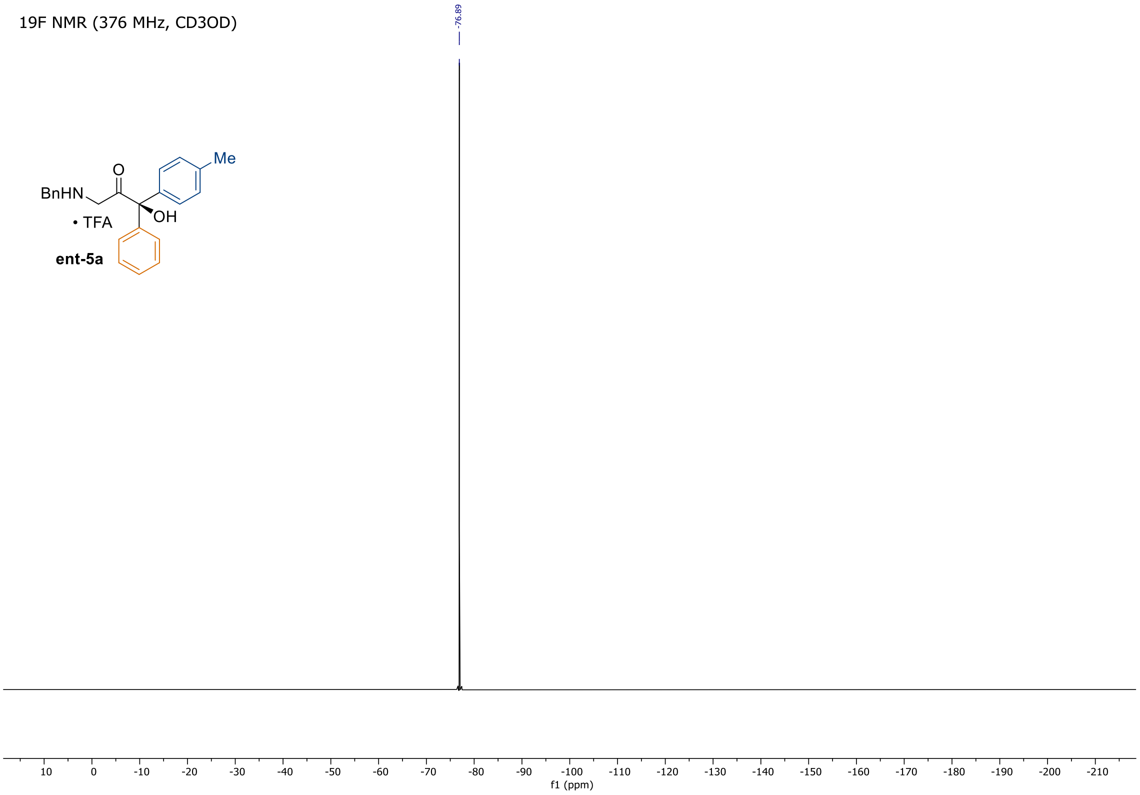

13C{1H} NMR (101 MHz, CD3OD)

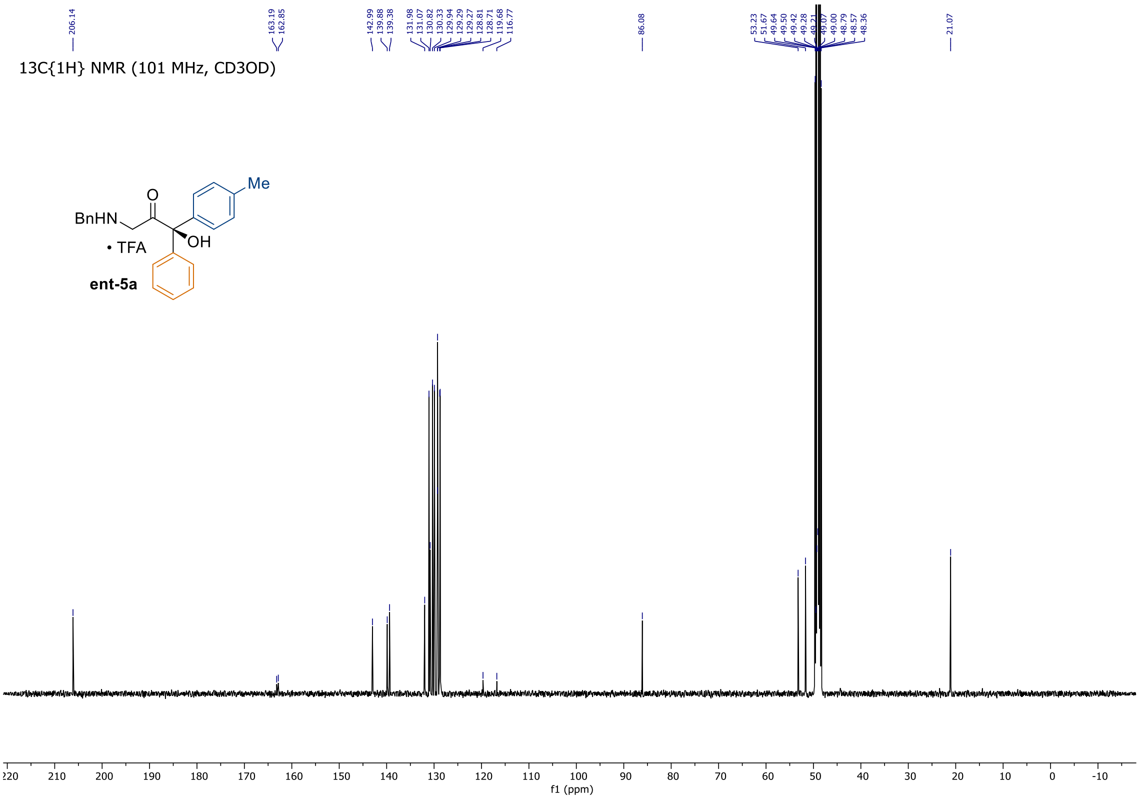

1H NMR (400 MHz, CD3OD)

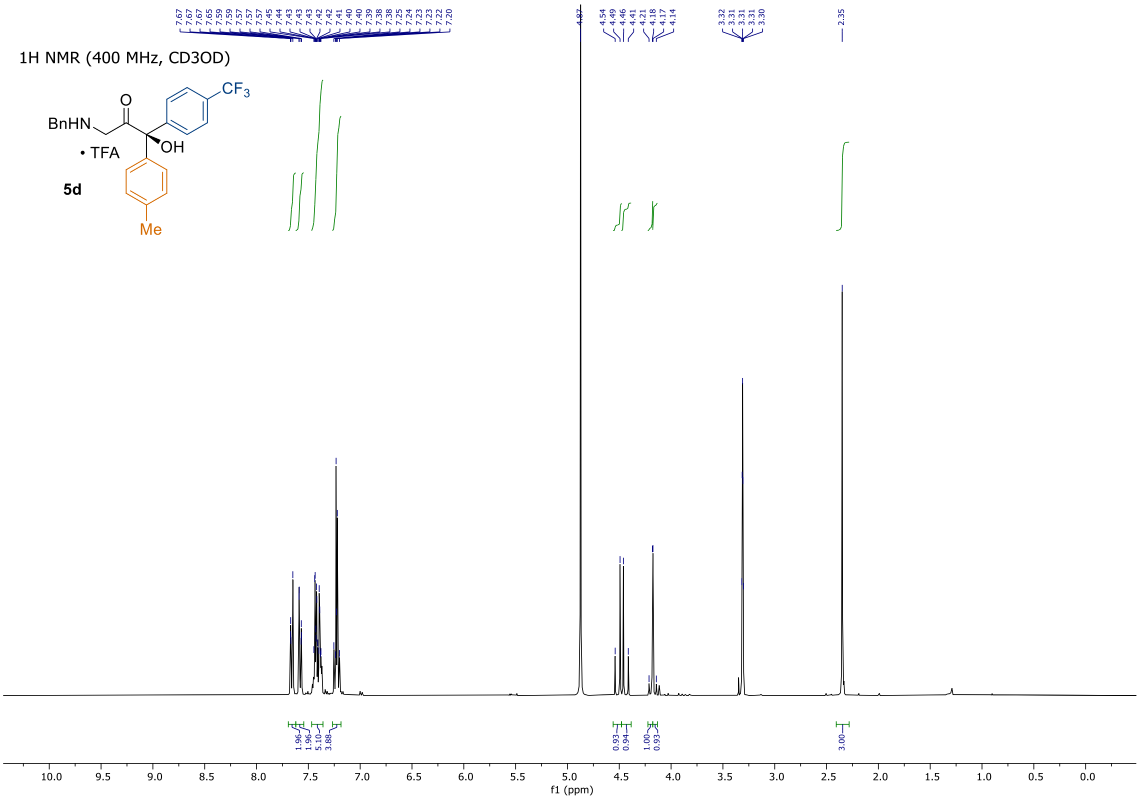

19F NMR (376 MHz, CD3OD)

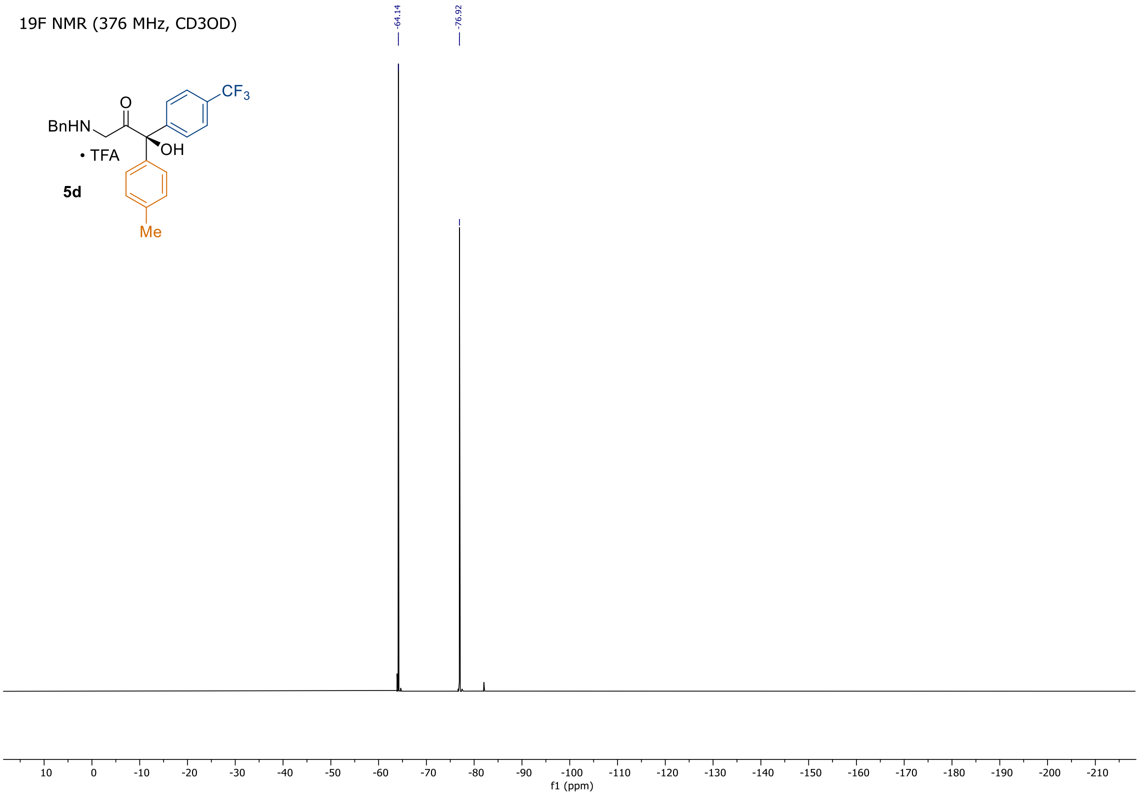

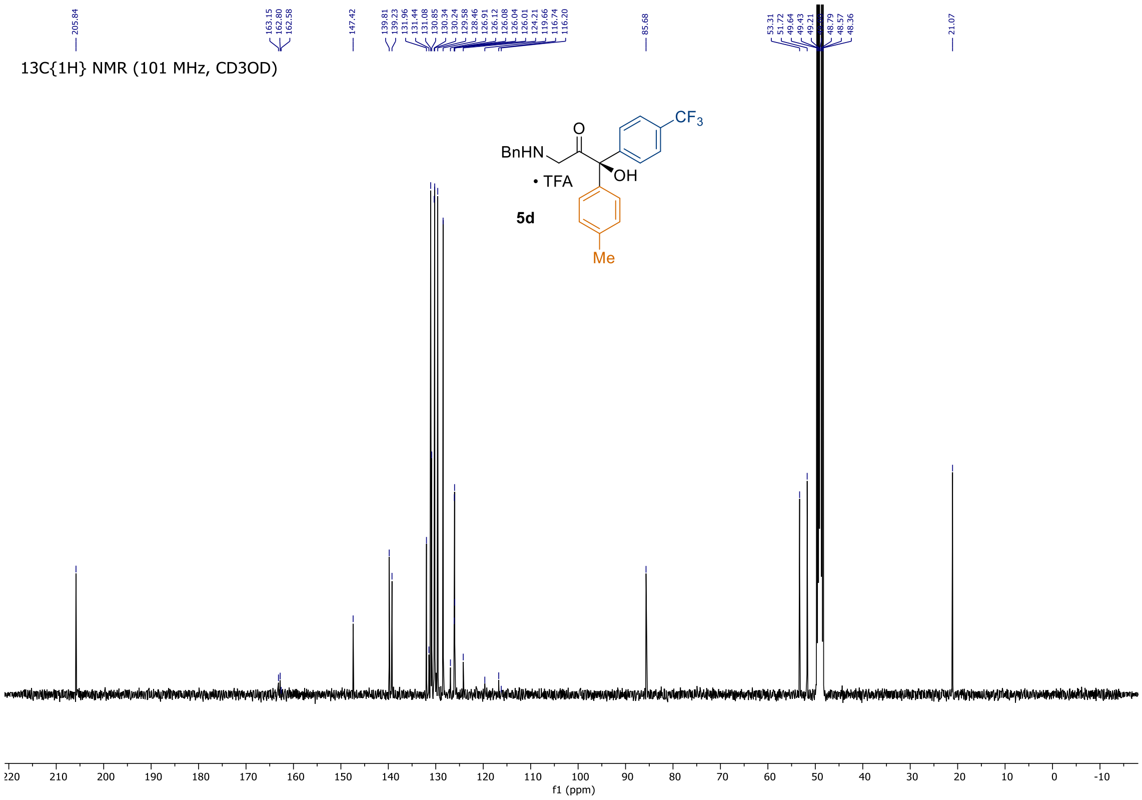
205.84
163.15
162.80
162.58
147.42
139.81
139.23
131.96
131.44
131.08
130.85
130.34
130.24
129.58
128.46
126.91
126.12
126.08
126.04
126.01
124.21
119.66
116.74
116.20
85.68
53.31
51.72
49.64
49.43
49.21
48.79
48.57
48.36
21.07
13C{1H} NMR (101 MHz, CD3OD)
BnHN
O
CF3
OH
• TFA
5d
Me
f1 (ppm)

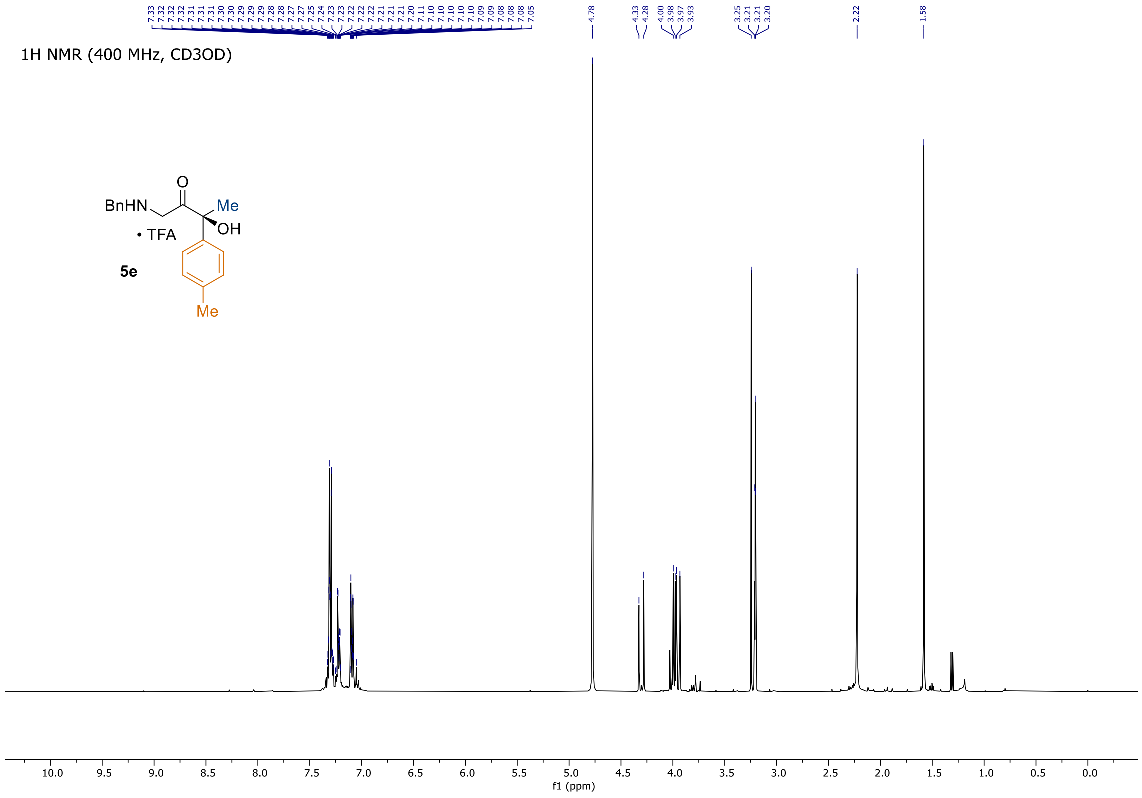
7.33
7.32
7.32
7.32
7.31
7.31
7.30
7.30
7.29
7.29
7.29
7.28
7.27
7.27
7.25
7.24
7.23
7.23
7.22
7.22
7.22
7.21
7.21
7.21
7.20
7.11
7.10
7.10
7.10
7.10
7.10
7.09
7.08
7.08
7.08
7.05
4.78
4.33
4.28
4.00
3.98
3.97
3.93
3.25
3.21
3.21
3.20
2.22
1.58
1H NMR (400 MHz, CD3OD)
BnHN
O
Me
OH
• TFA
5e
Me
f1 (ppm)

19F NMR (376 MHz, CD3OD)

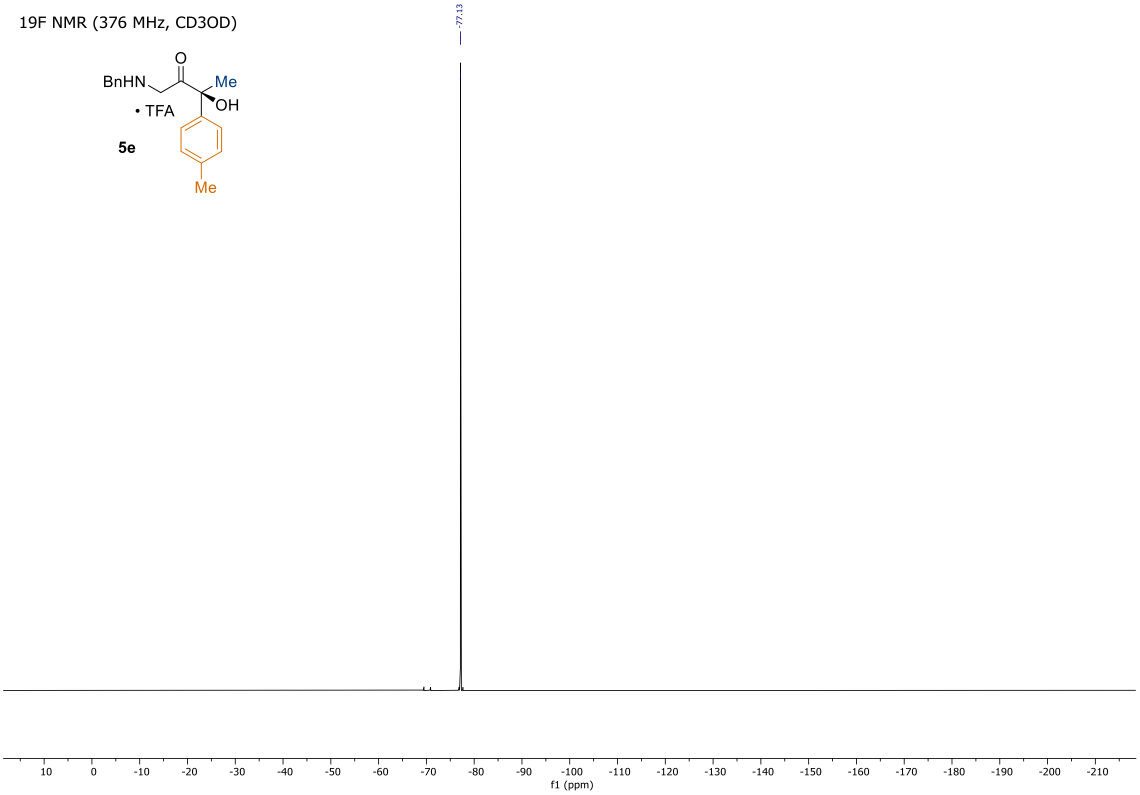

13C{1H} NMR (101 MHz, CD3OD)

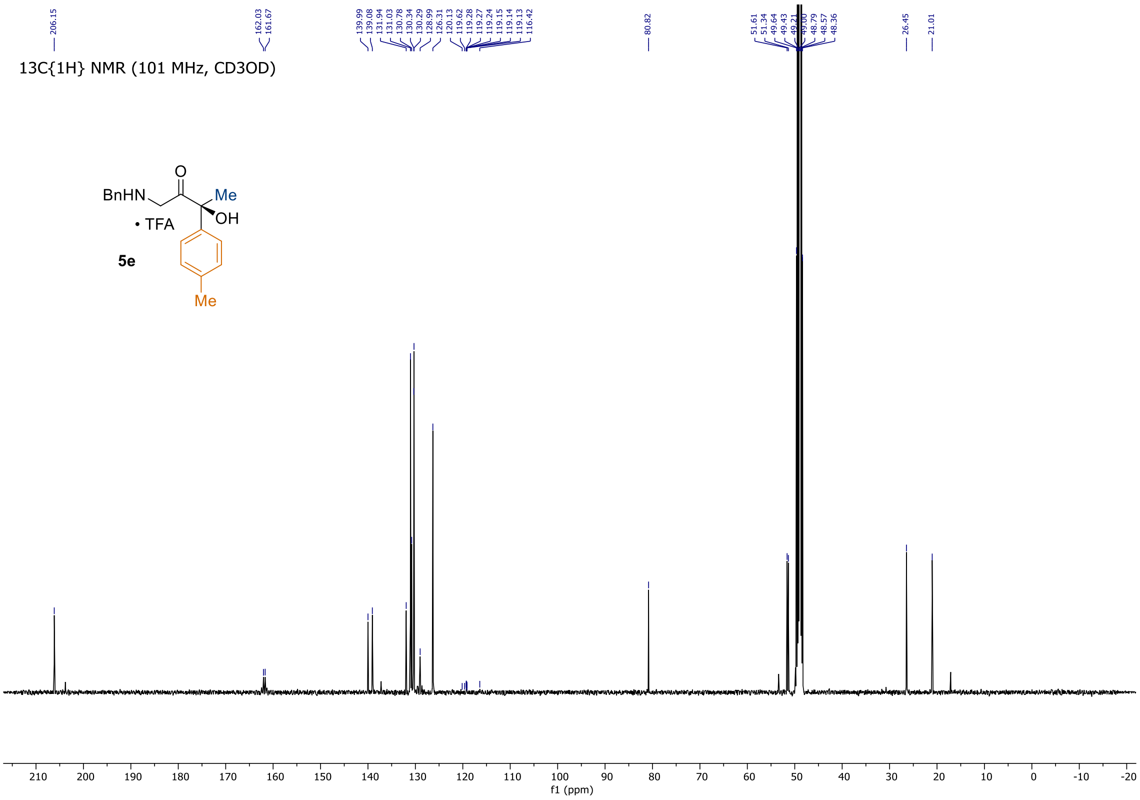

1H NMR (400 MHz, CD3OD)

5f

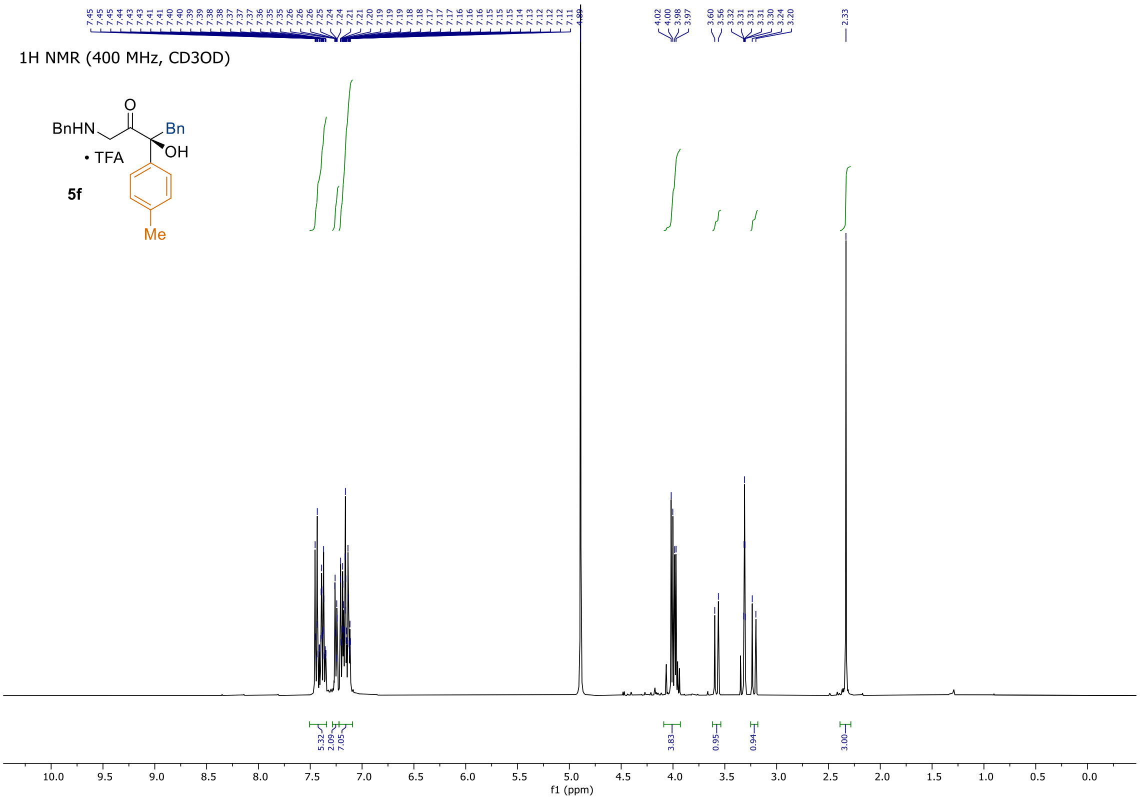

19F NMR (376 MHz, CD3OD)

5f

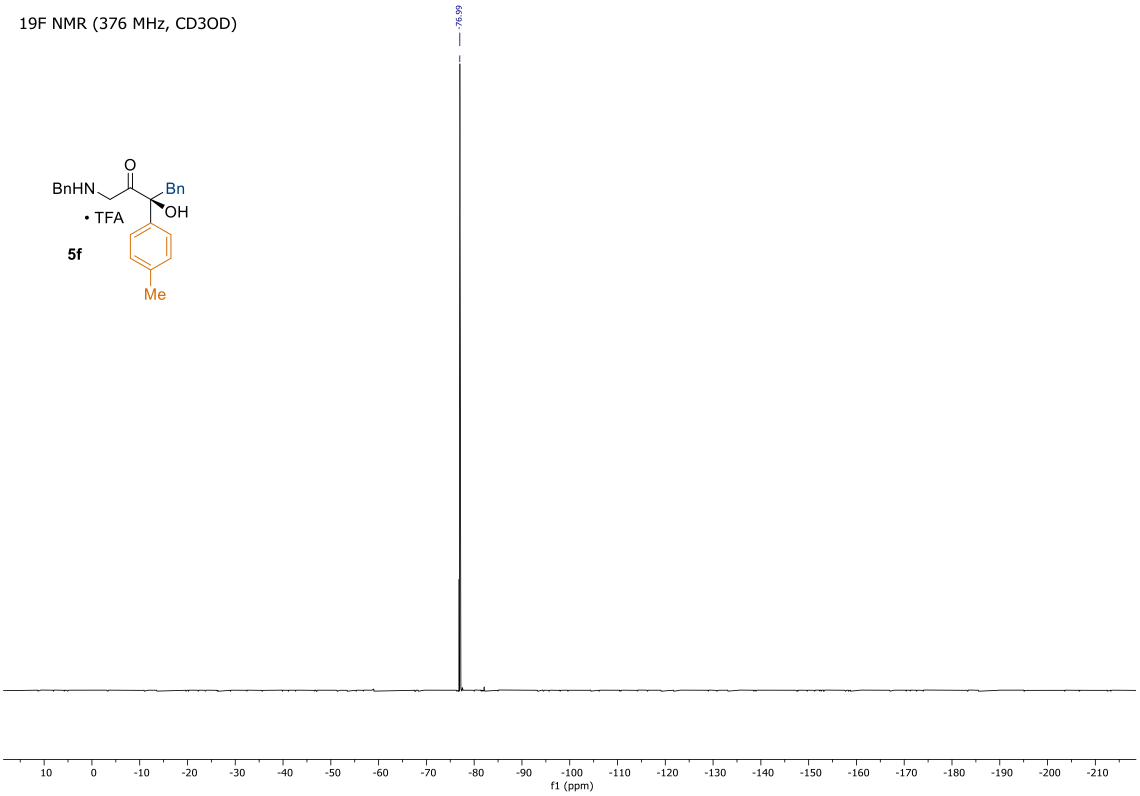

13C{1H} NMR (101 MHz, CD3OD)

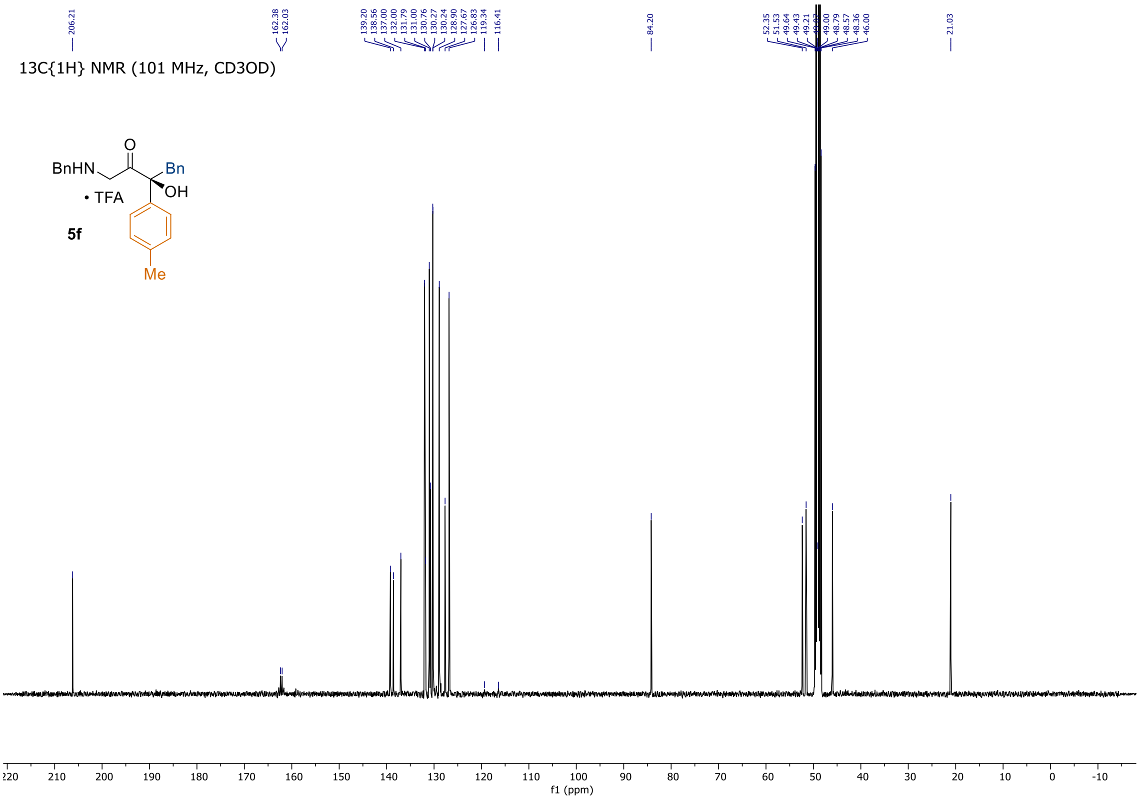

1H NMR (400 MHz, CD3OD)

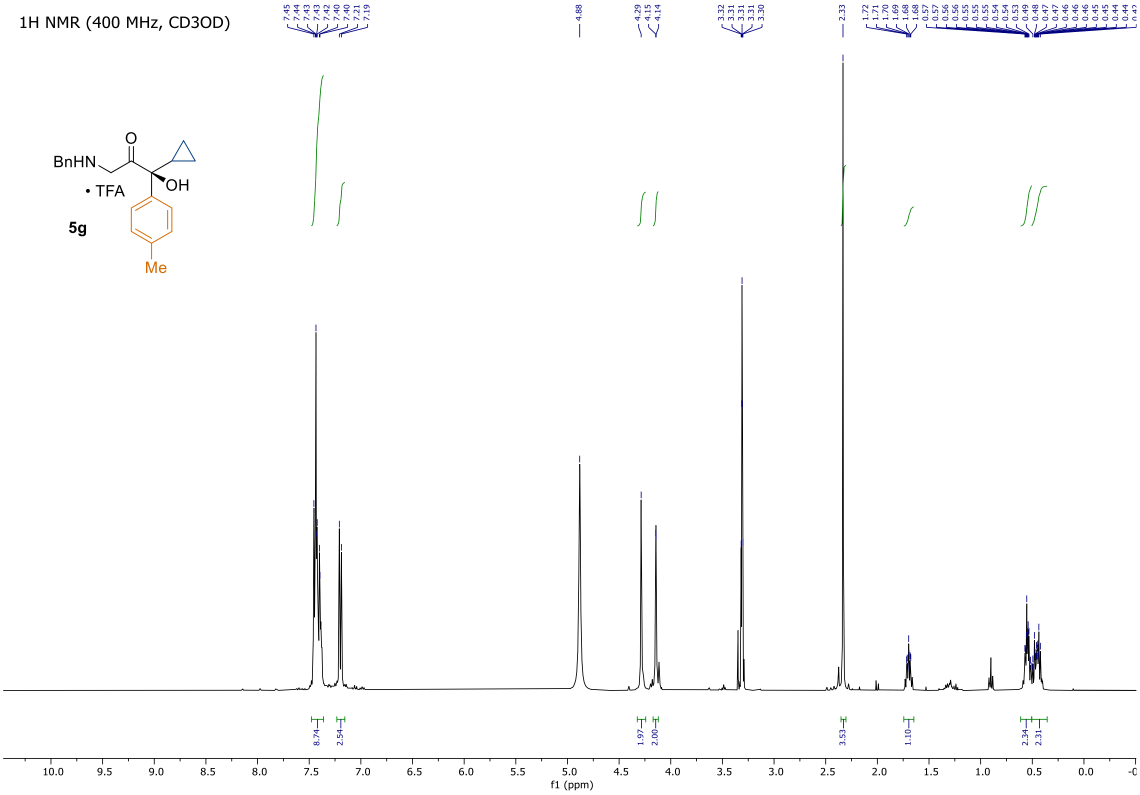

19F NMR (376 MHz, CD3OD)

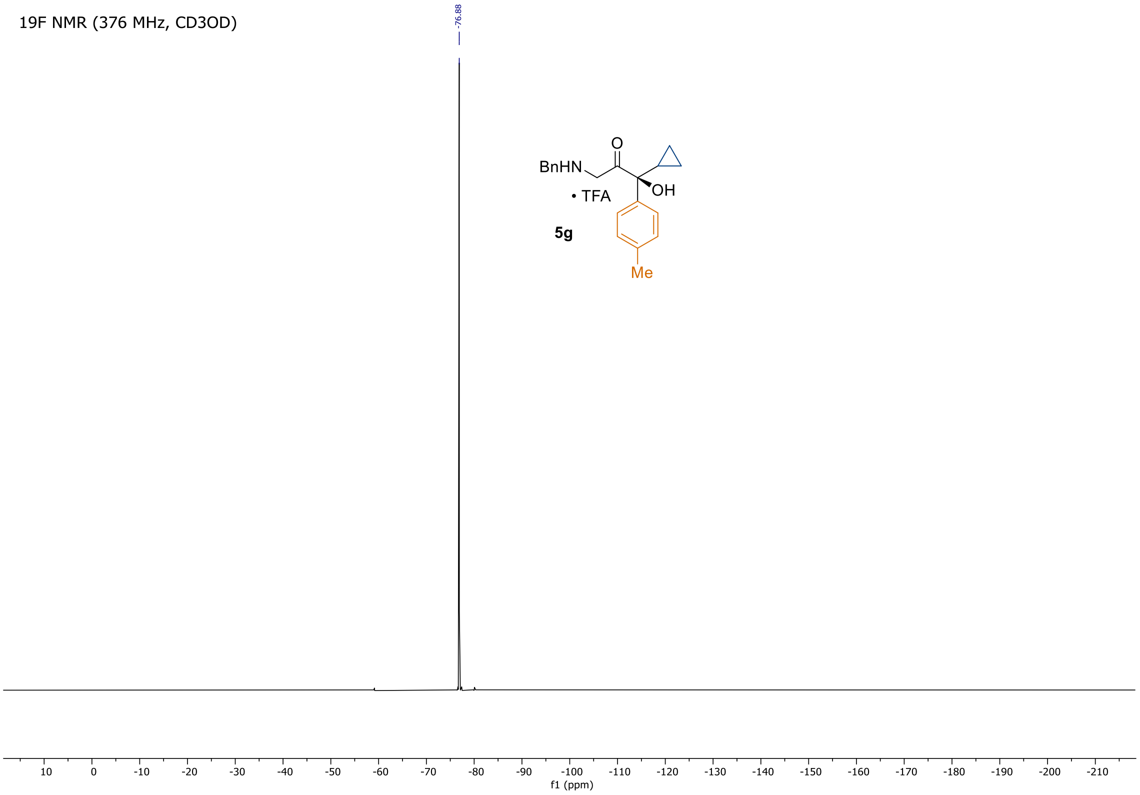

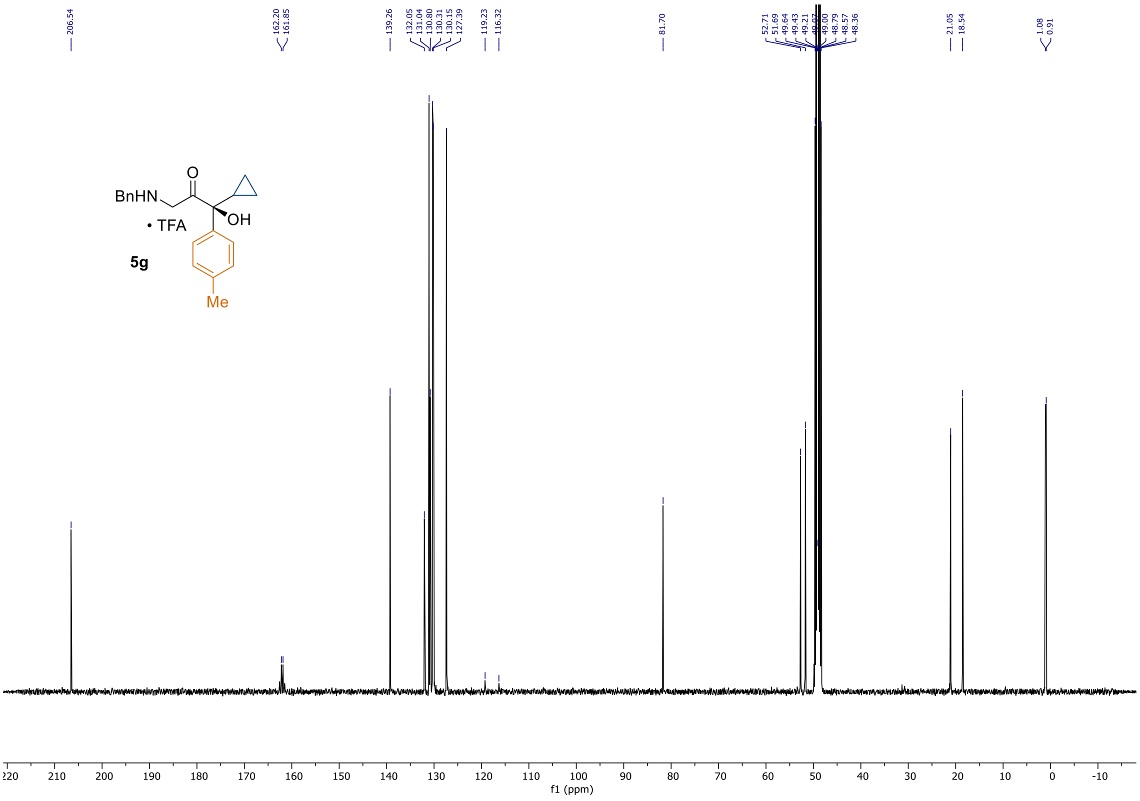

1H NMR (400 MHz, CD3OD)

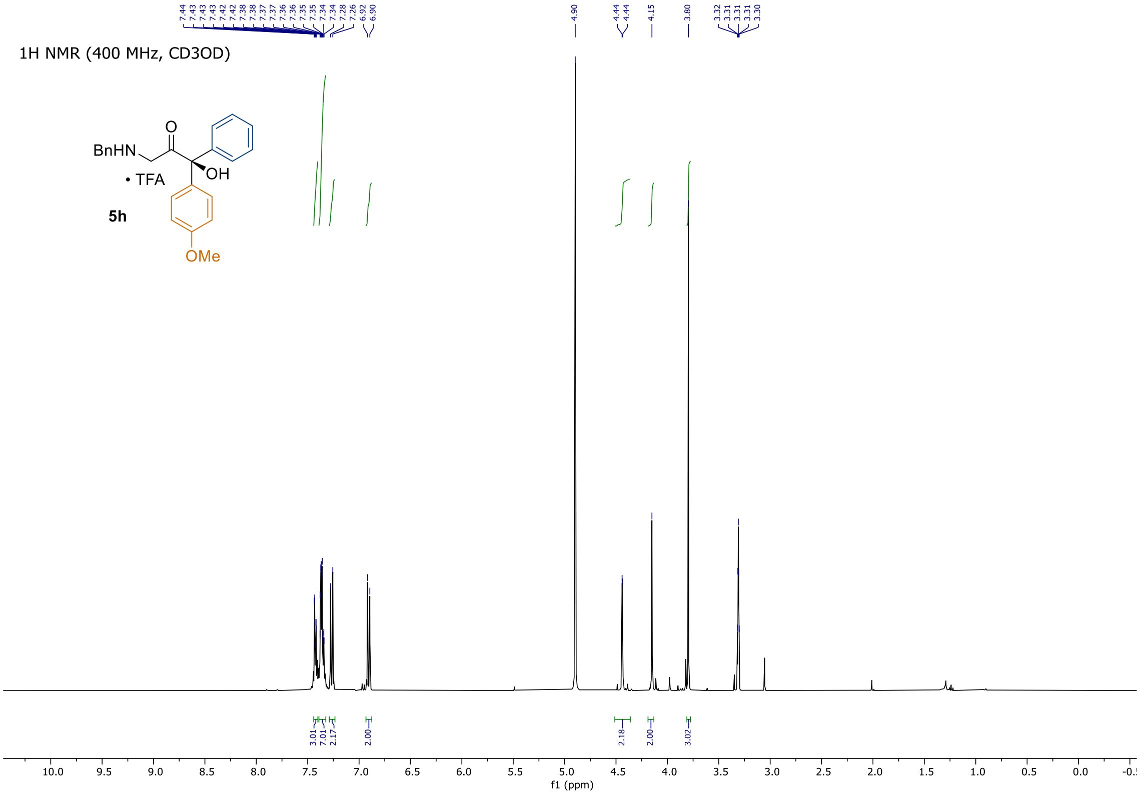

19F NMR (376 MHz, CD3OD)

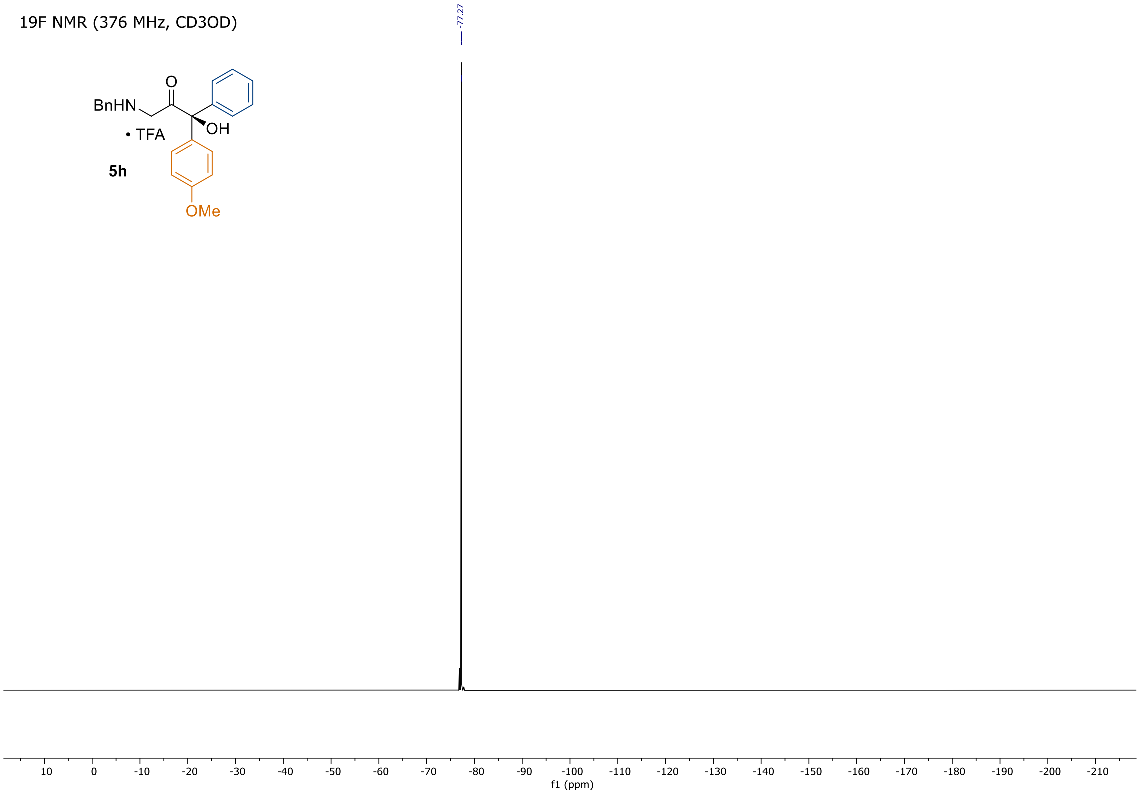

13C{1H} NMR (101 MHz, CD3OD)

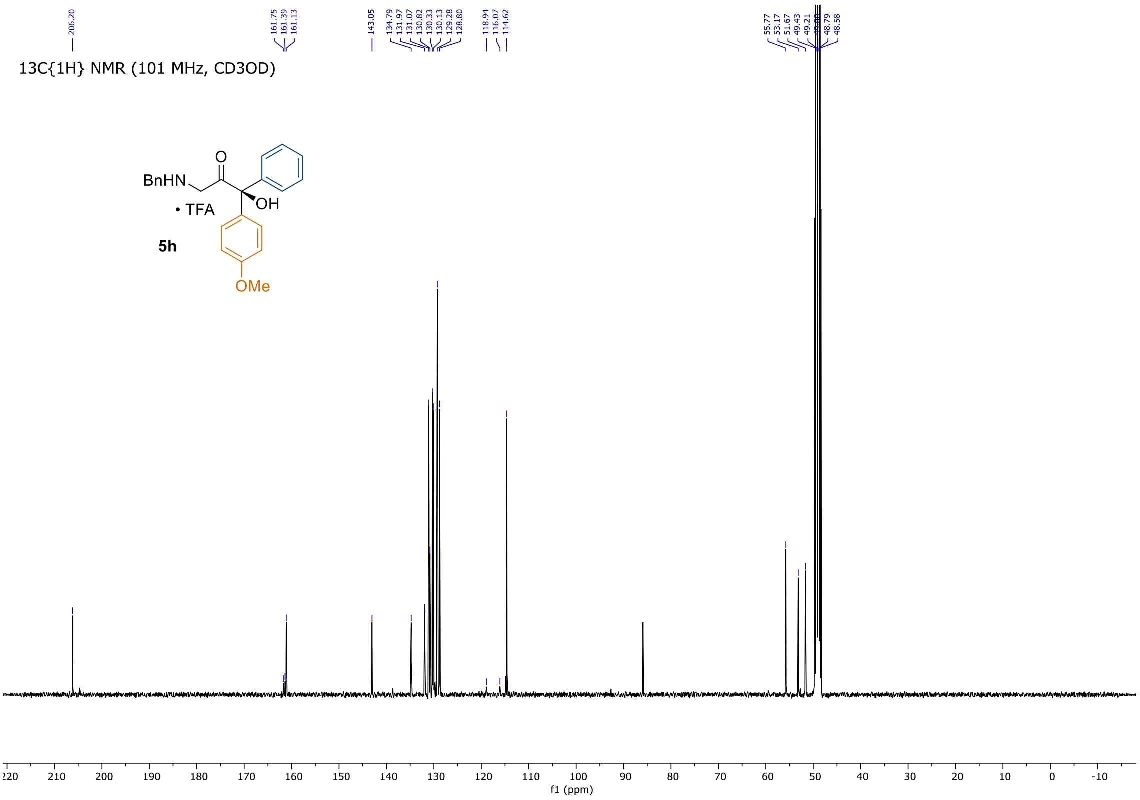

5h

1H NMR (400 MHz, CD3OD)

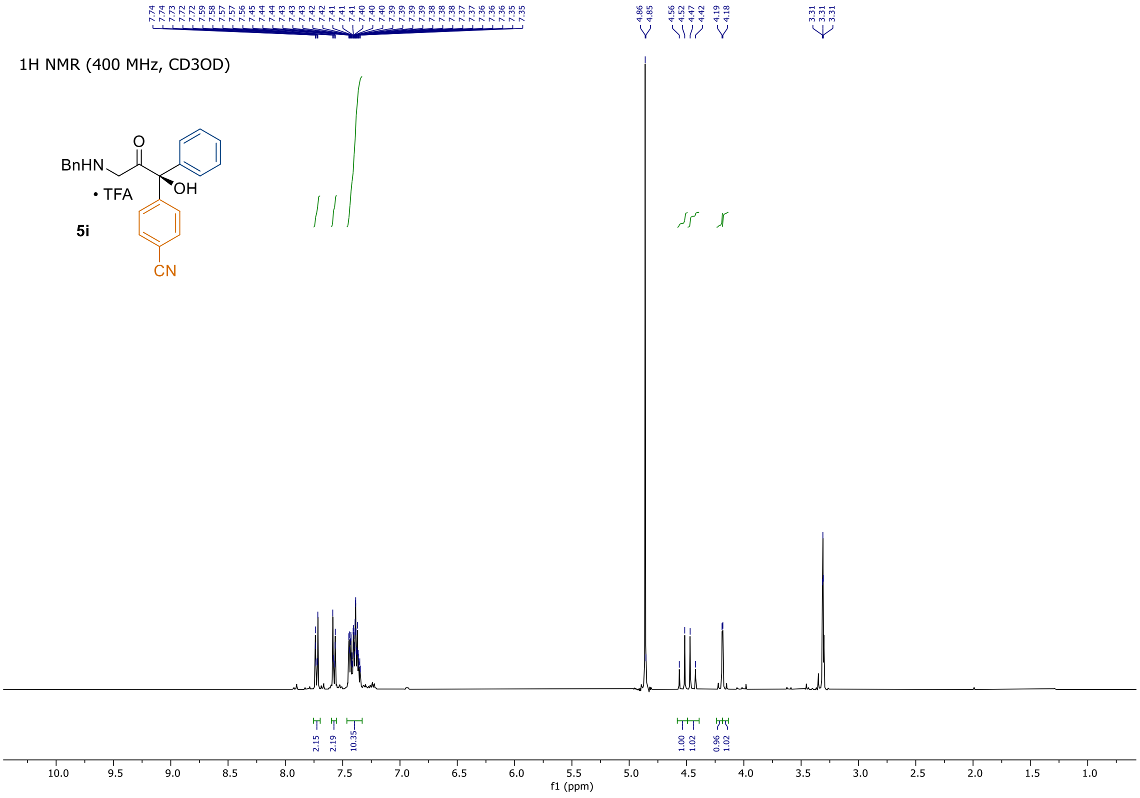

5i

19F NMR (376 MHz, CD3OD)

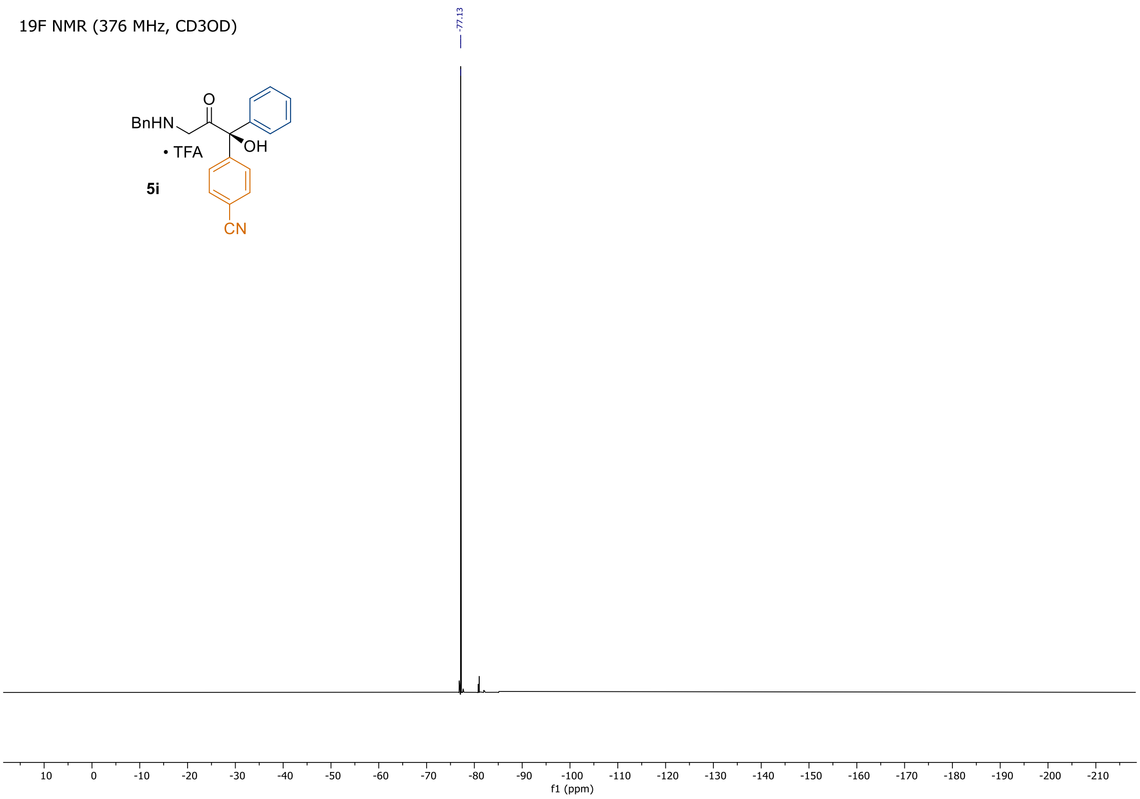

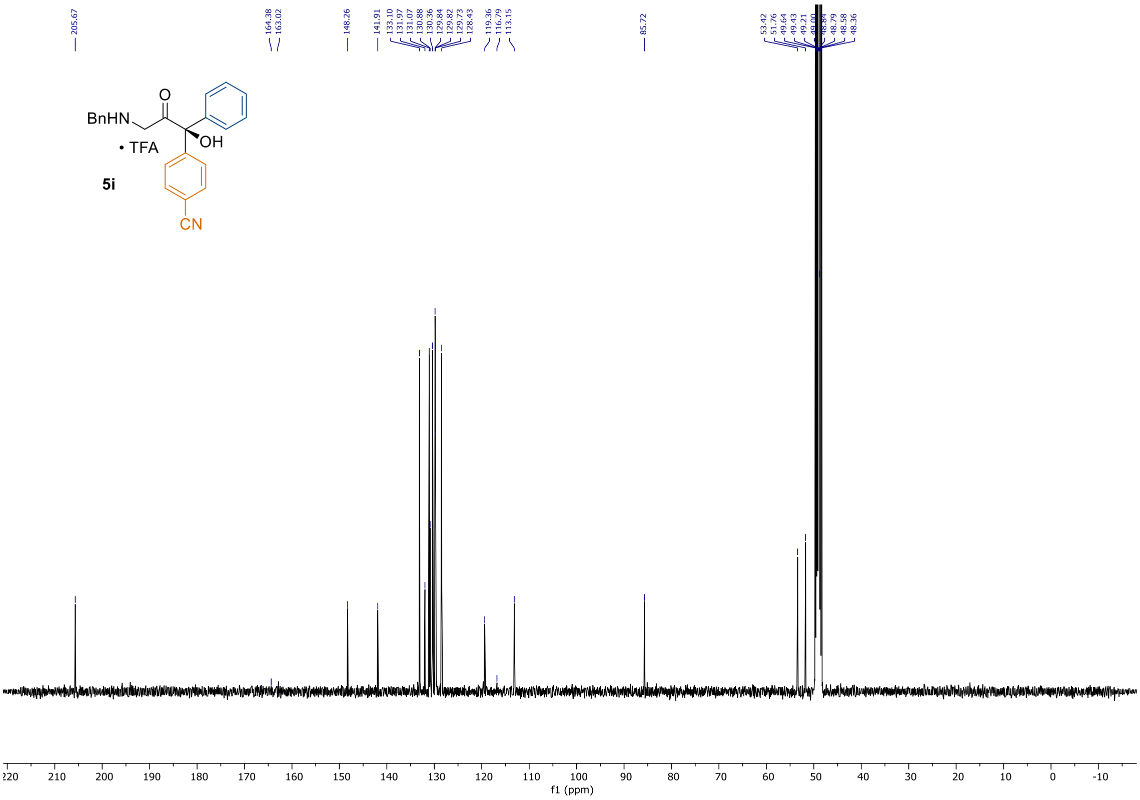

1H NMR (400 MHz, CD3OD)

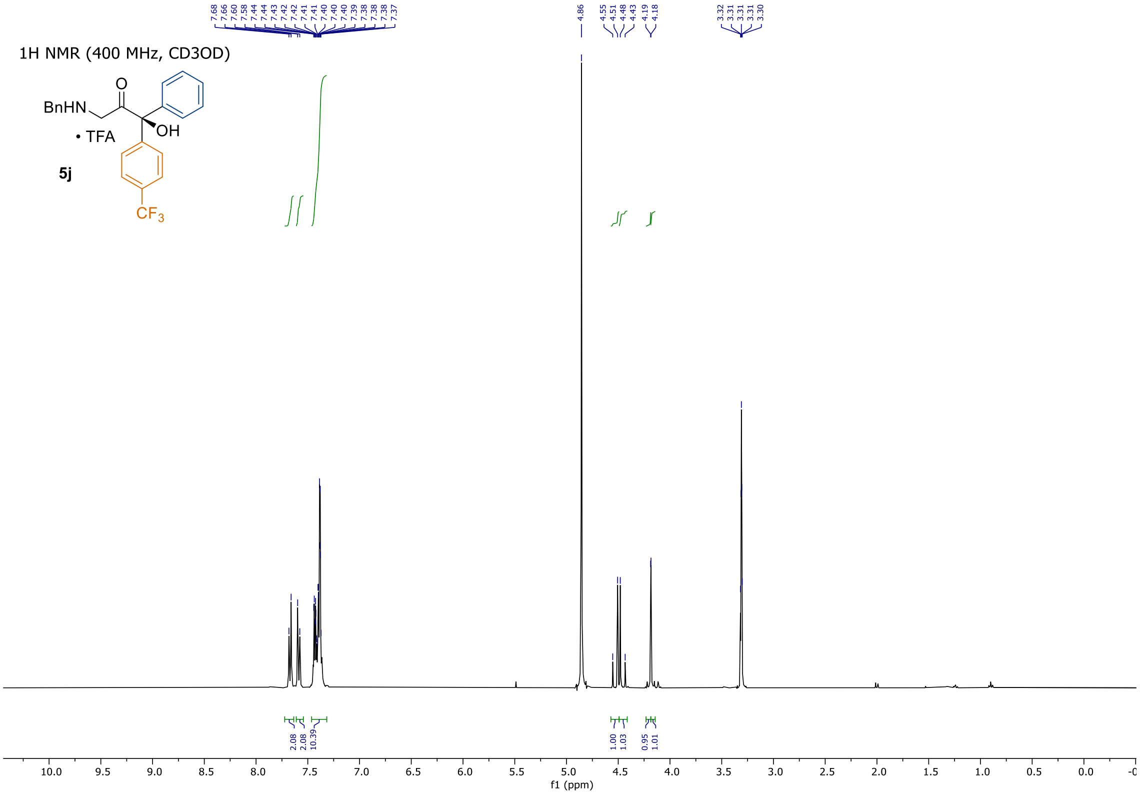

19F NMR (376 MHz, CD3OD)

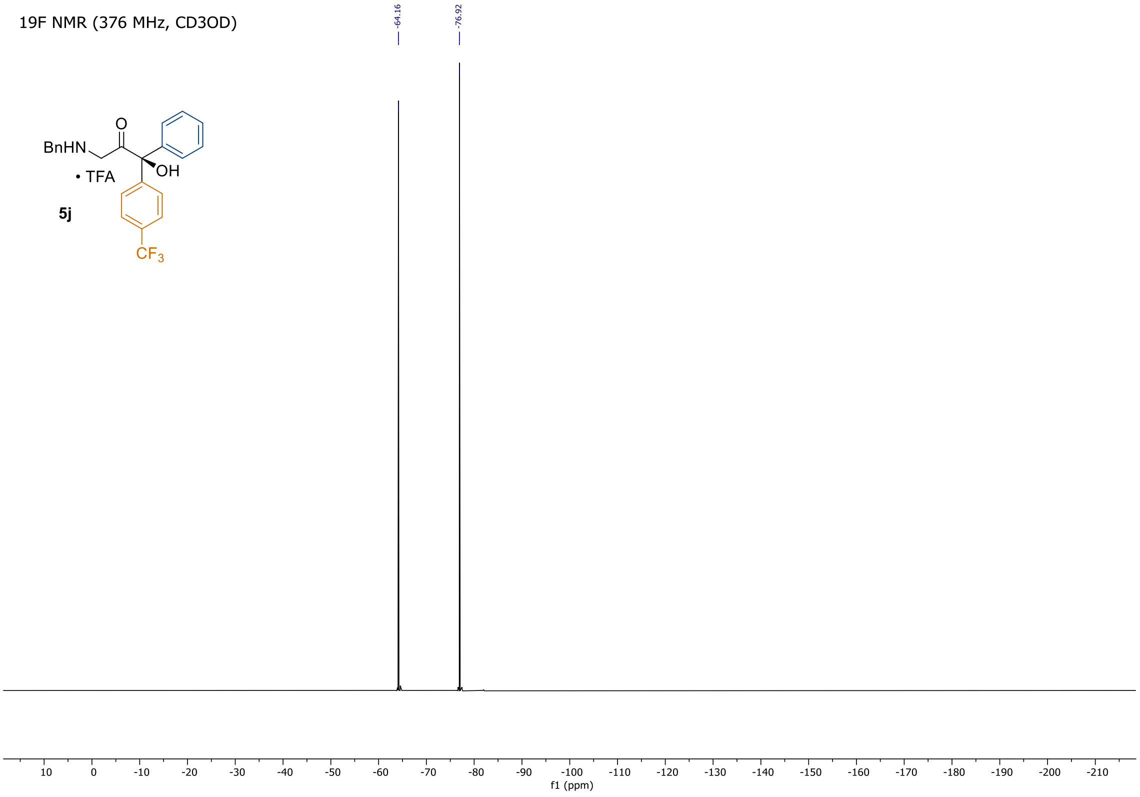

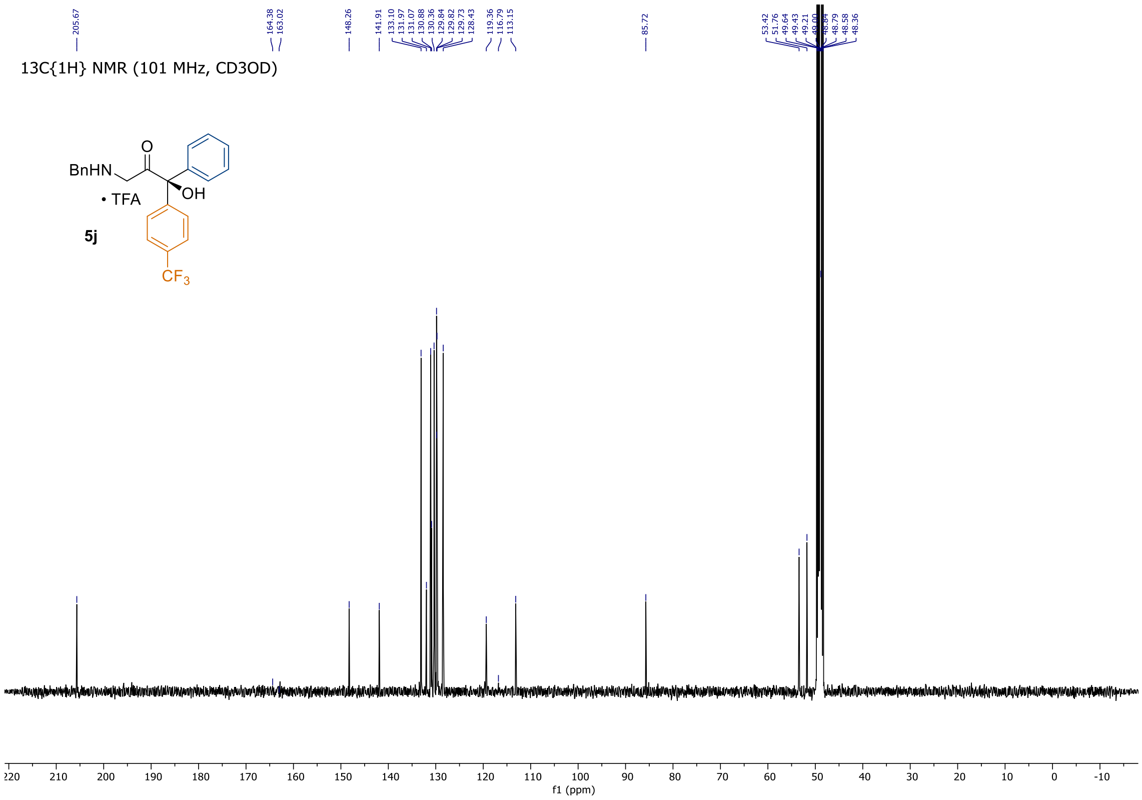
13C{1H} NMR (101 MHz, CD3OD)
BnHN
O
OH
• TFA
5j
CF_3
f1 (ppm)

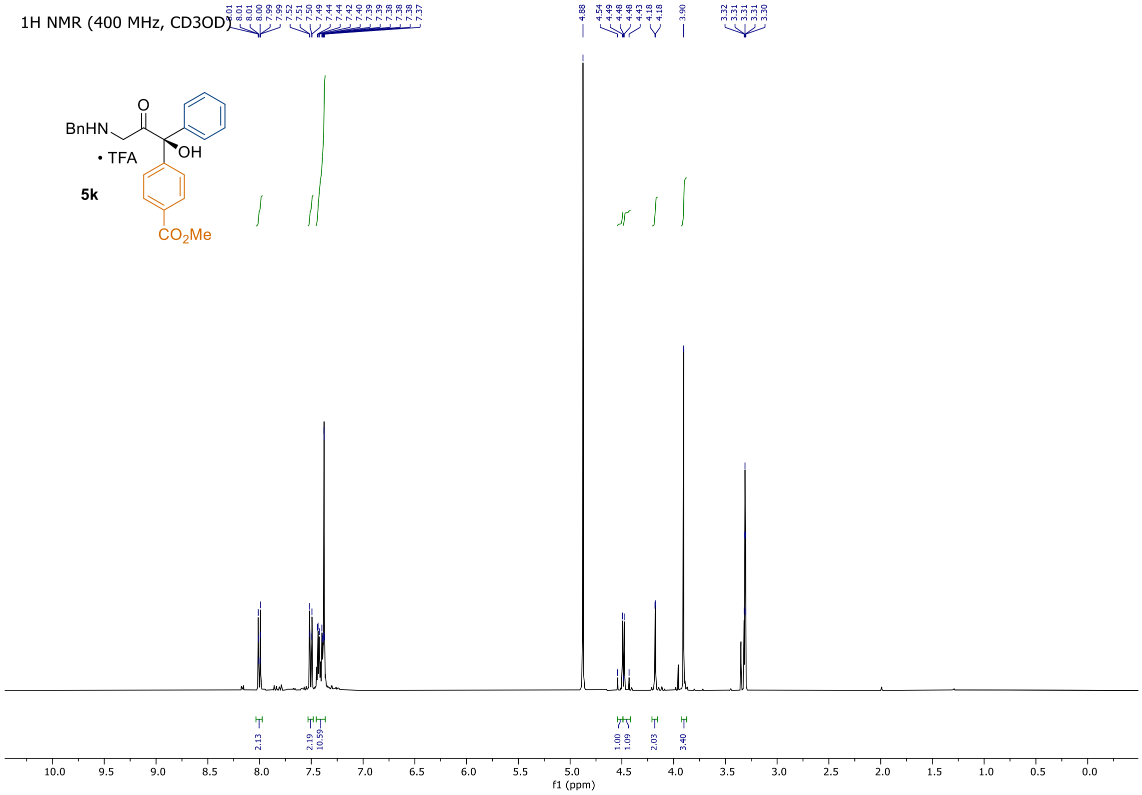
1H NMR (400 MHz, CD3OD)
BnHN
O
OH
• TFA
5k
CO_2Me
f1 (ppm)

19F NMR (376 MHz, CD3OD)

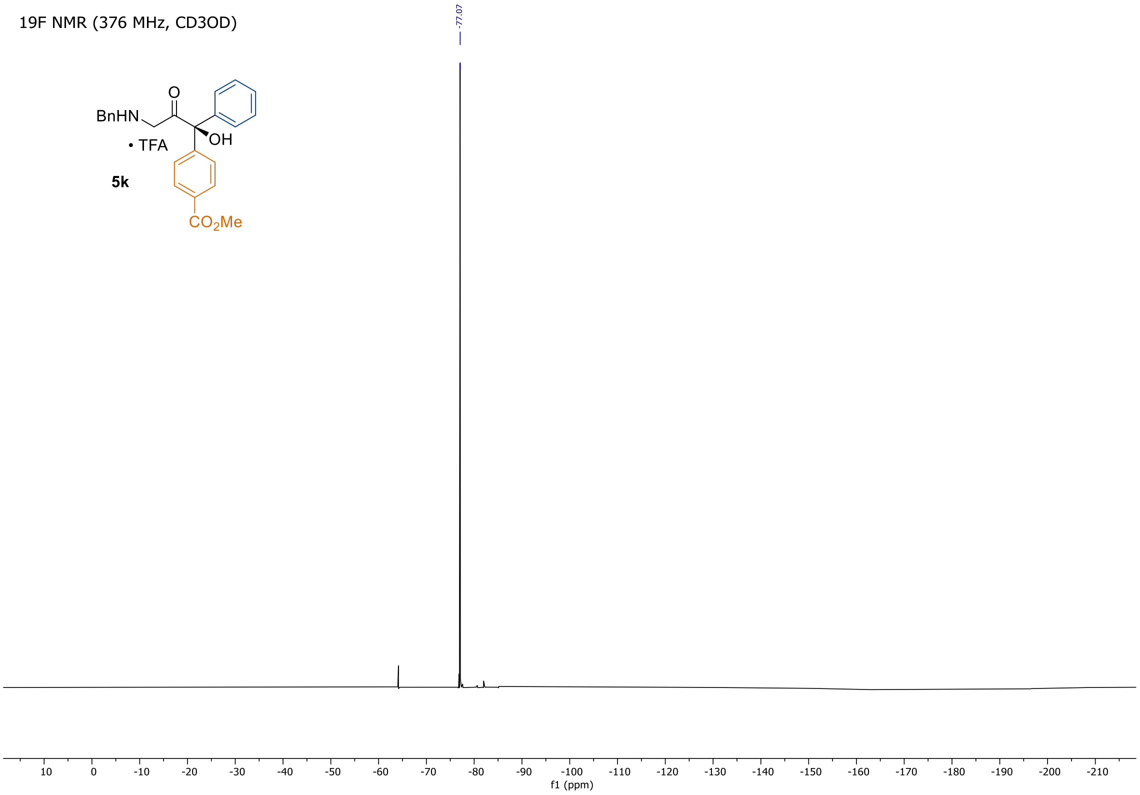

13C{1H} NMR (101 MHz, CD3OD)

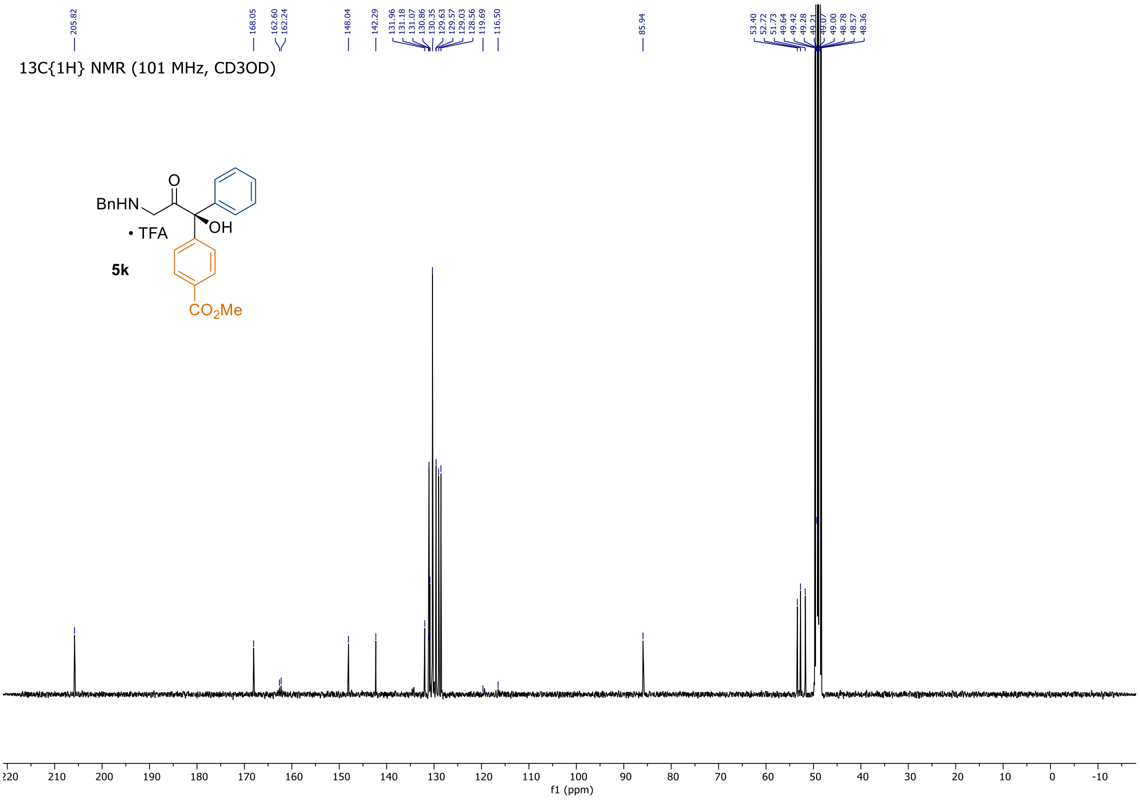

1H NMR (400 MHz, CD3OD)

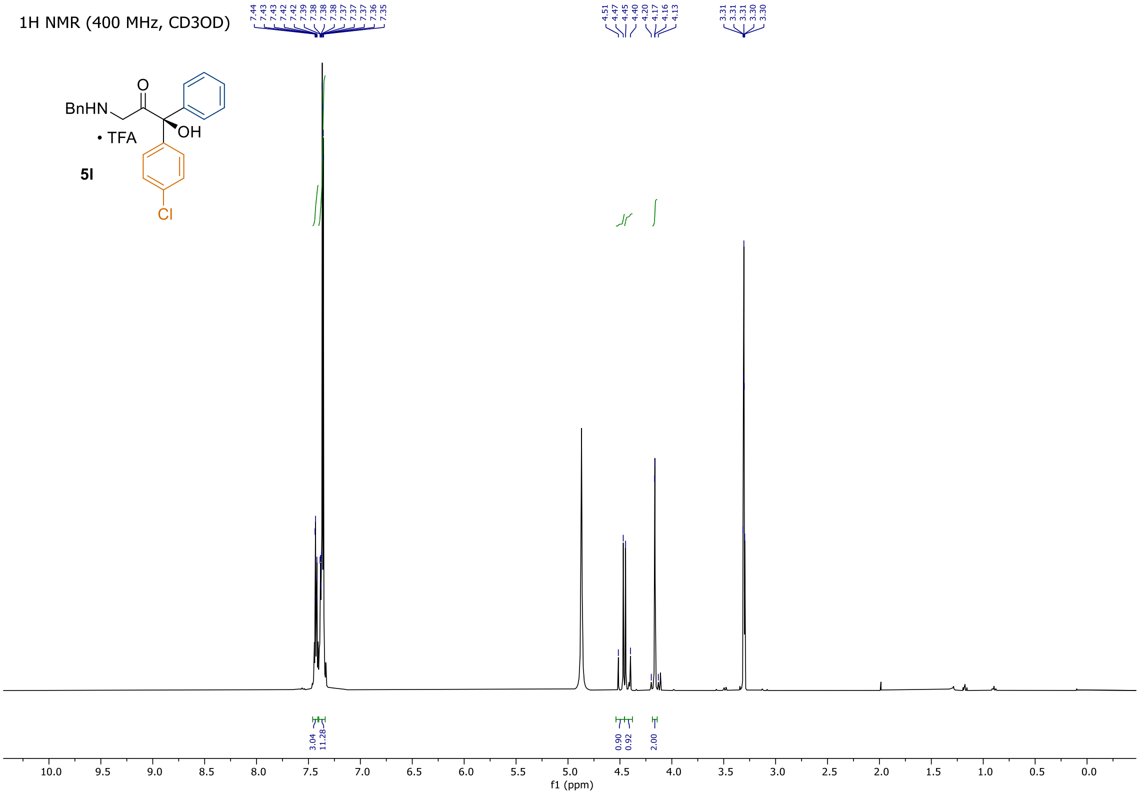

19F NMR (376 MHz, CD3OD)

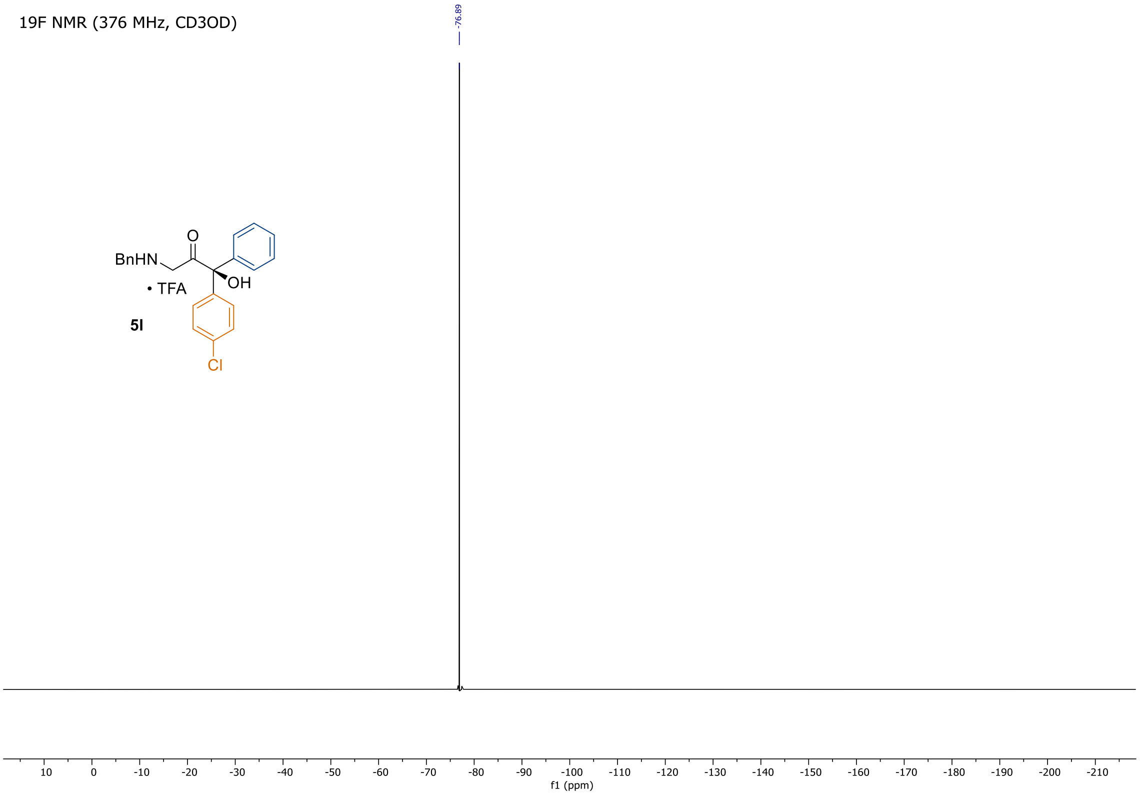

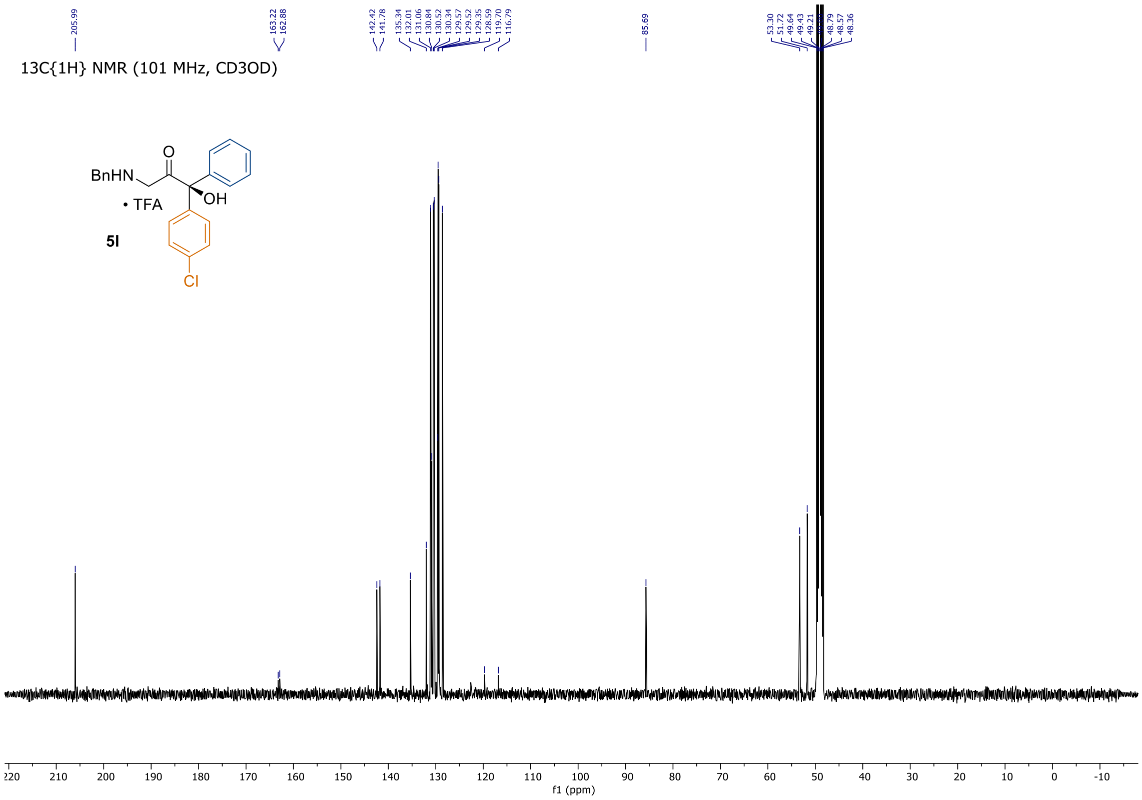
205.99
163.22
162.88
142.42
141.78
135.34
132.01
131.06
130.84
130.52
130.34
129.57
129.52
129.35
128.59
119.70
116.79
85.69
53.30
51.72
49.64
49.43
49.21
48.79
48.57
48.36
13C{1H} NMR (101 MHz, CD3OD)
BnHN
O
OH
• TFA
5l
Cl
f1 (ppm)

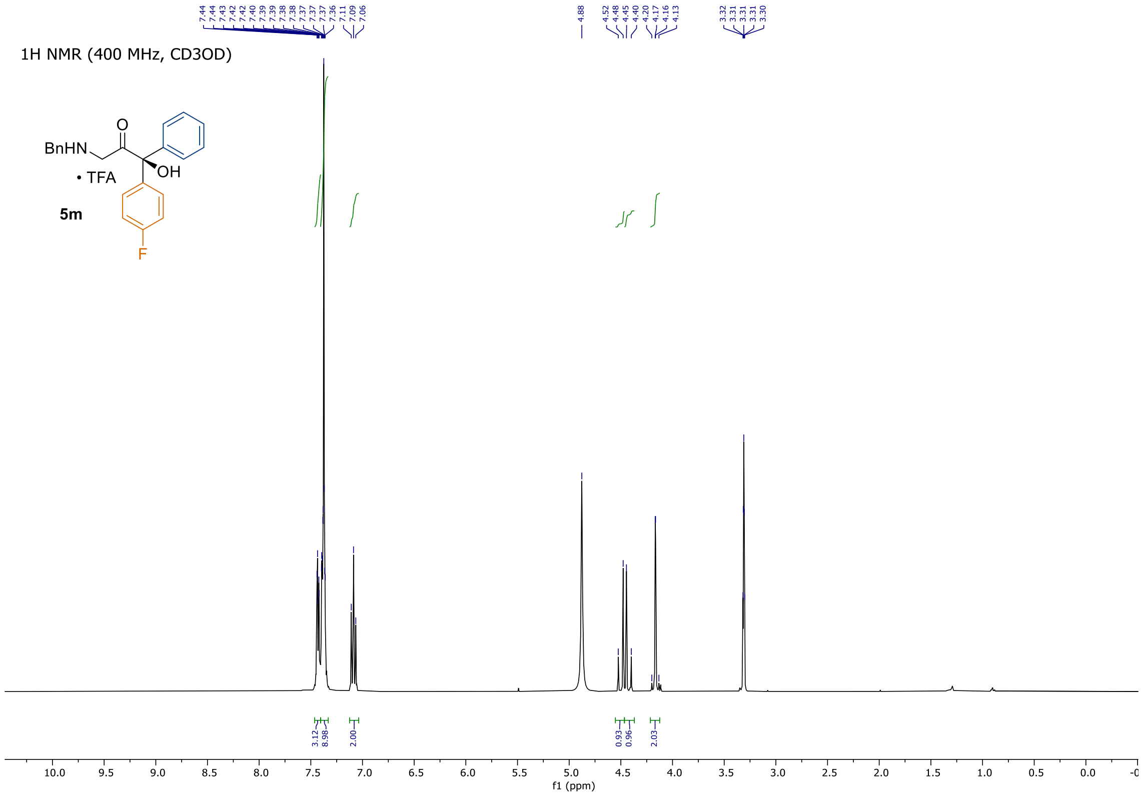
7.44
7.44
7.43
7.42
7.42
7.40
7.39
7.39
7.38
7.38
7.37
7.37
7.37
7.36
7.11
7.09
7.06
4.88
4.52
4.48
4.45
4.40
4.20
4.17
4.16
4.13
3.32
3.31
3.31
3.31
3.30
1H NMR (400 MHz, CD3OD)
BnHN
O
OH
• TFA
5m
F
3.12
8.98
2.00
0.93
0.96
2.03
f1 (ppm)

1H NMR (400 MHz, CD3OD)

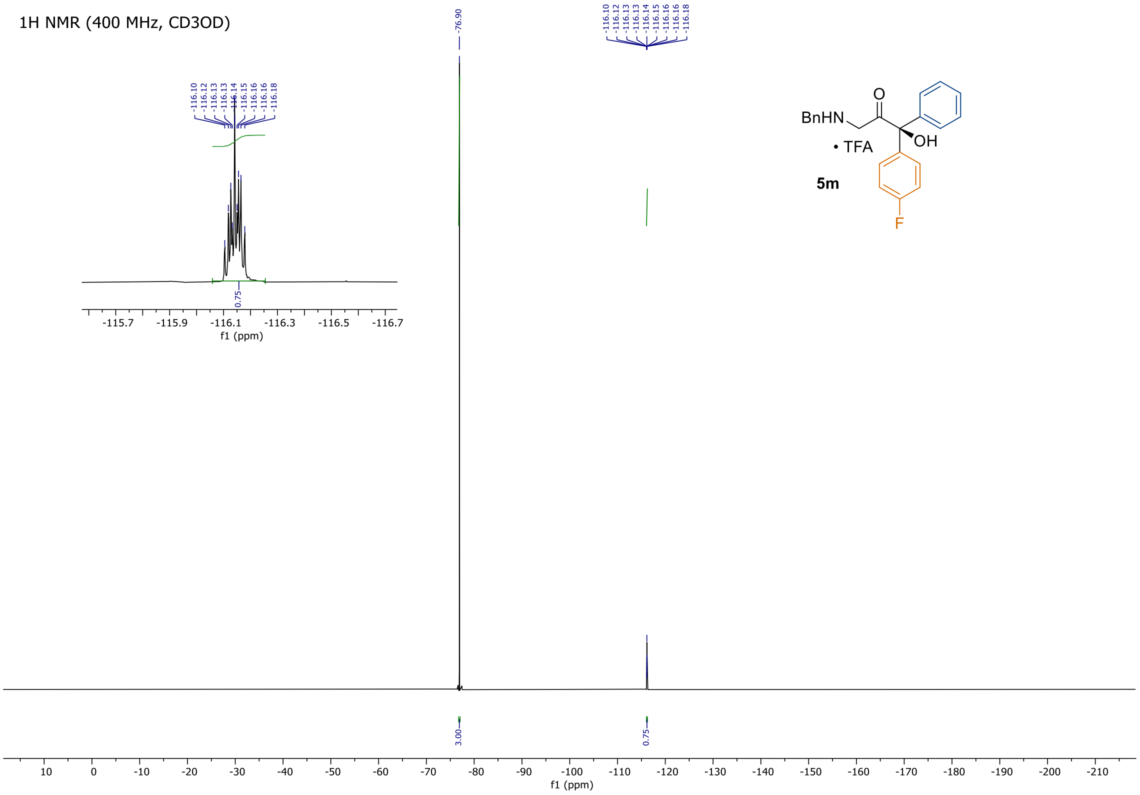

13C{1H} NMR (101 MHz, CD3OD)

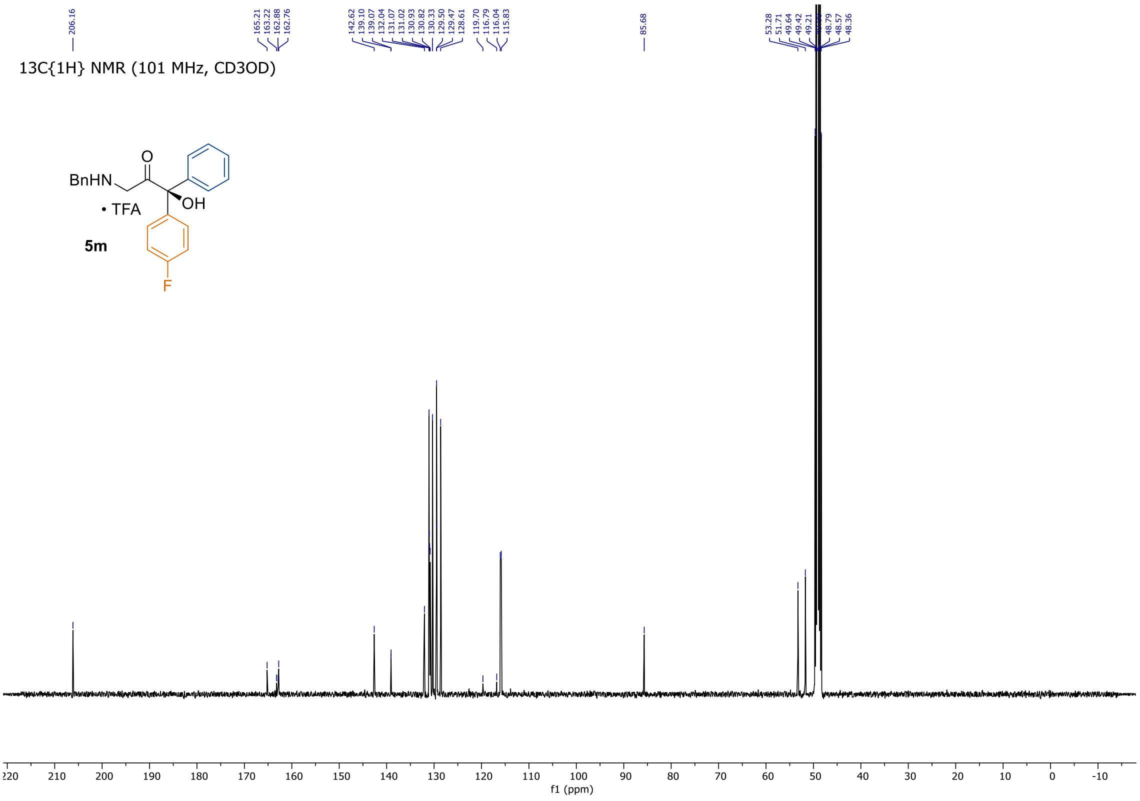

1H NMR (400 MHz, CD3OD)

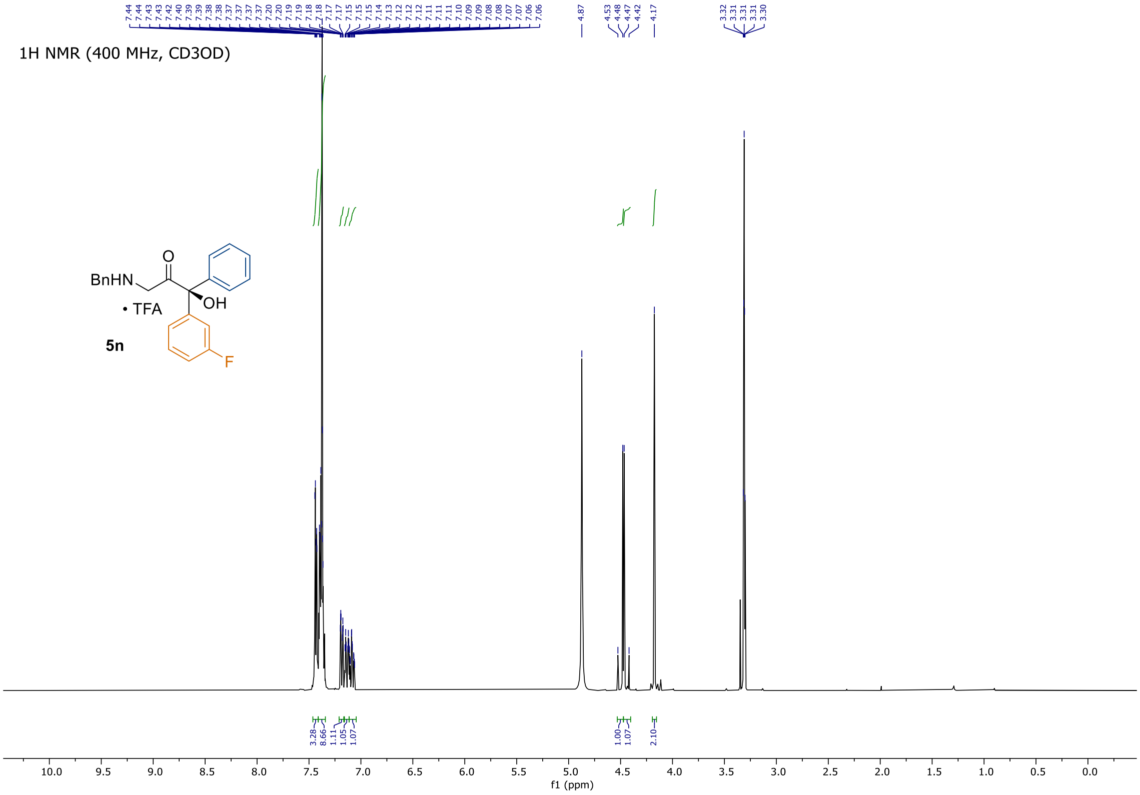

19F NMR (376 MHz, CD3OD)

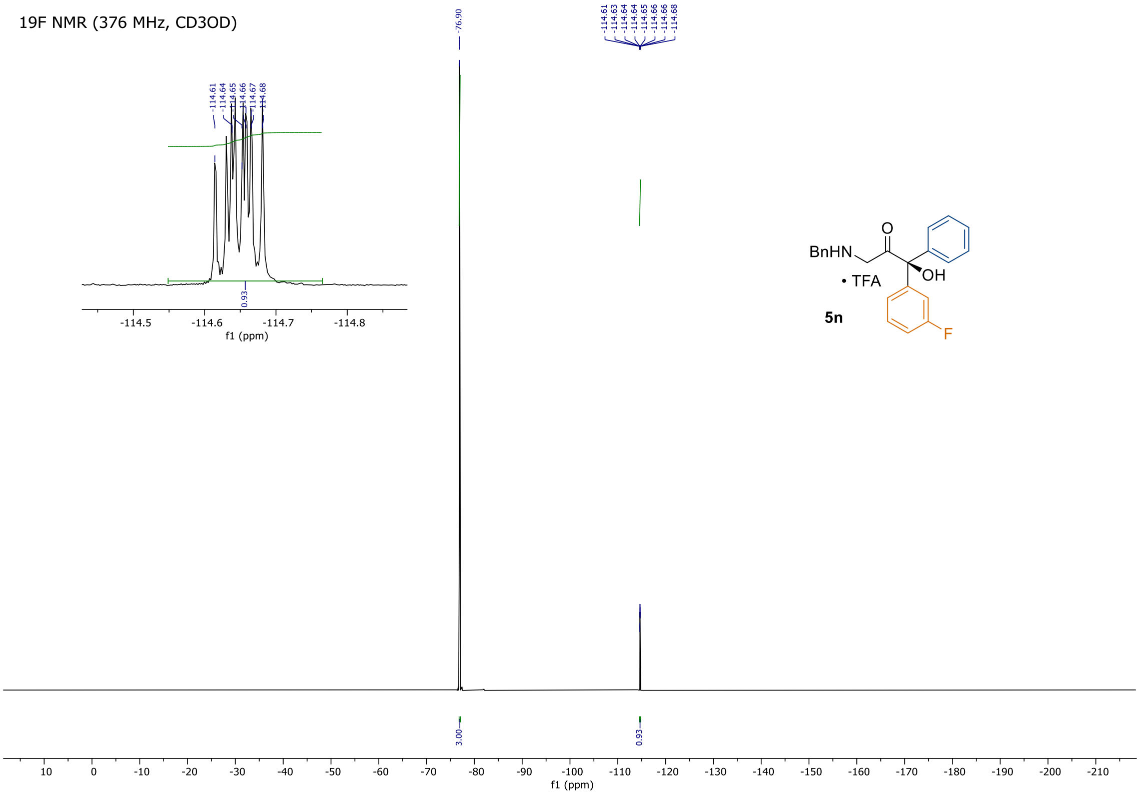

13C{1H} NMR (101 MHz, CD3OD)

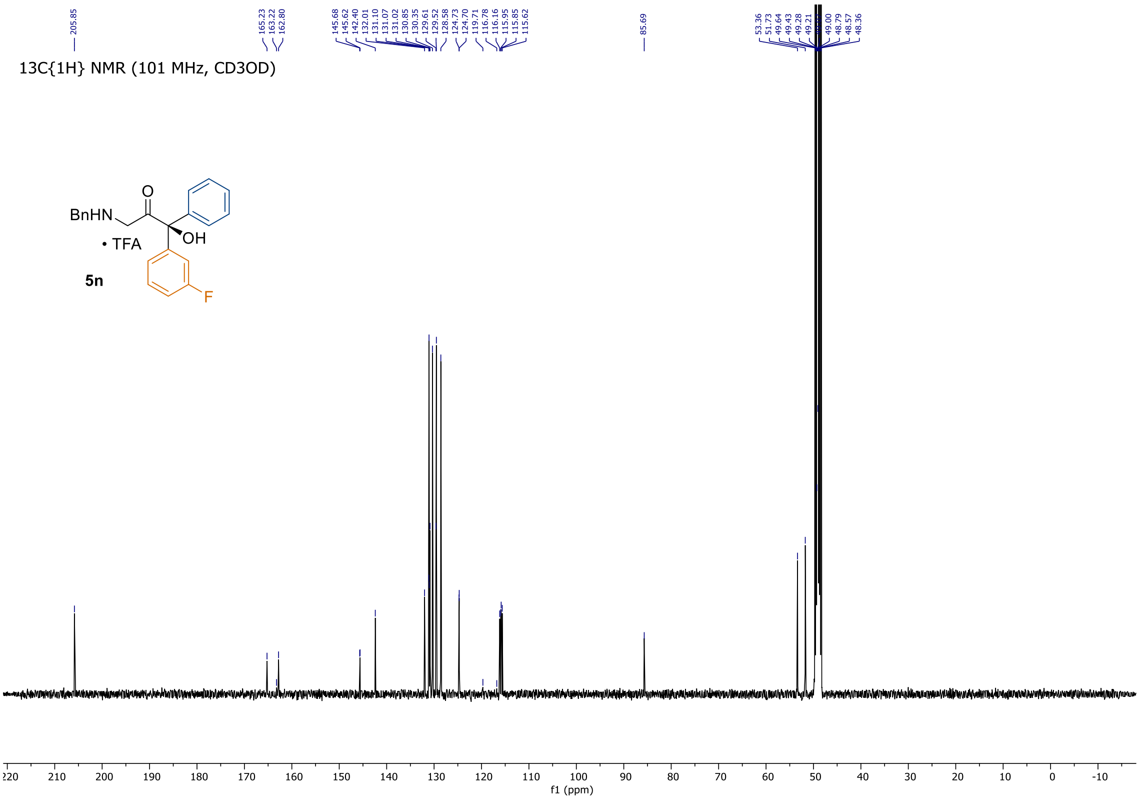

1H NMR (400 MHz, CD3OD)

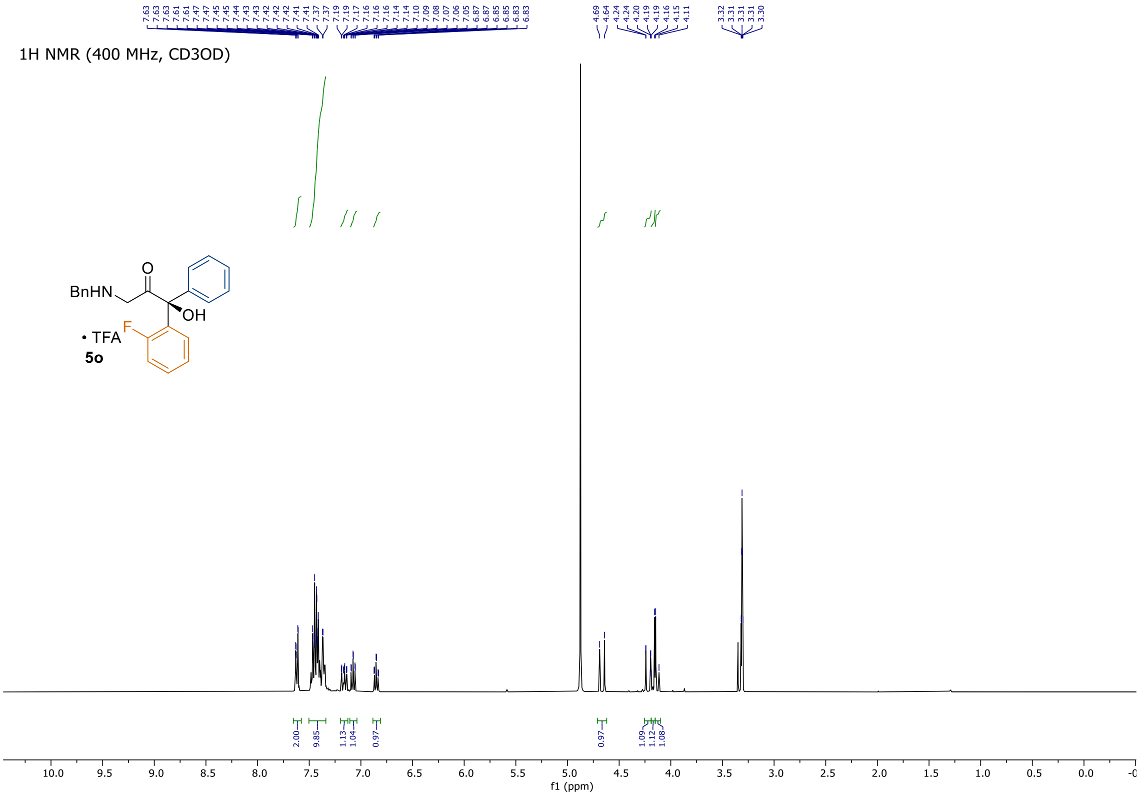

19F NMR (376 MHz, CD3OD)

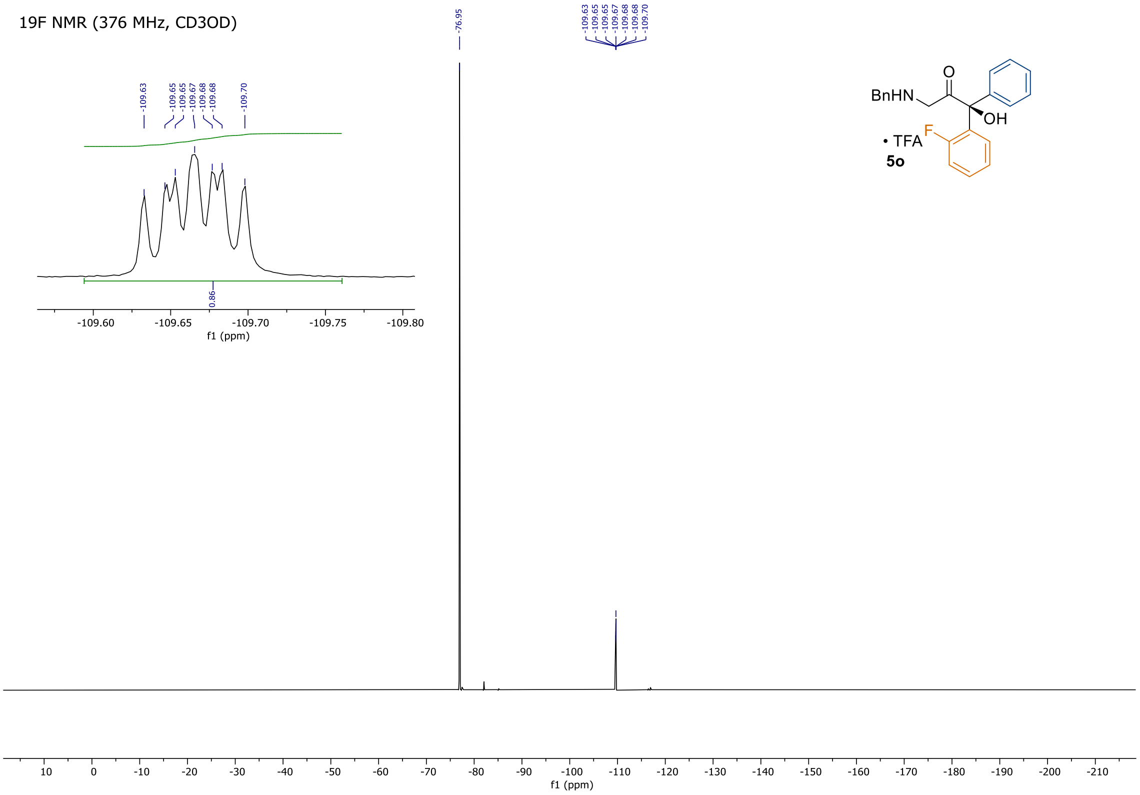

13C{1H} NMR (101 MHz, CD3OD)

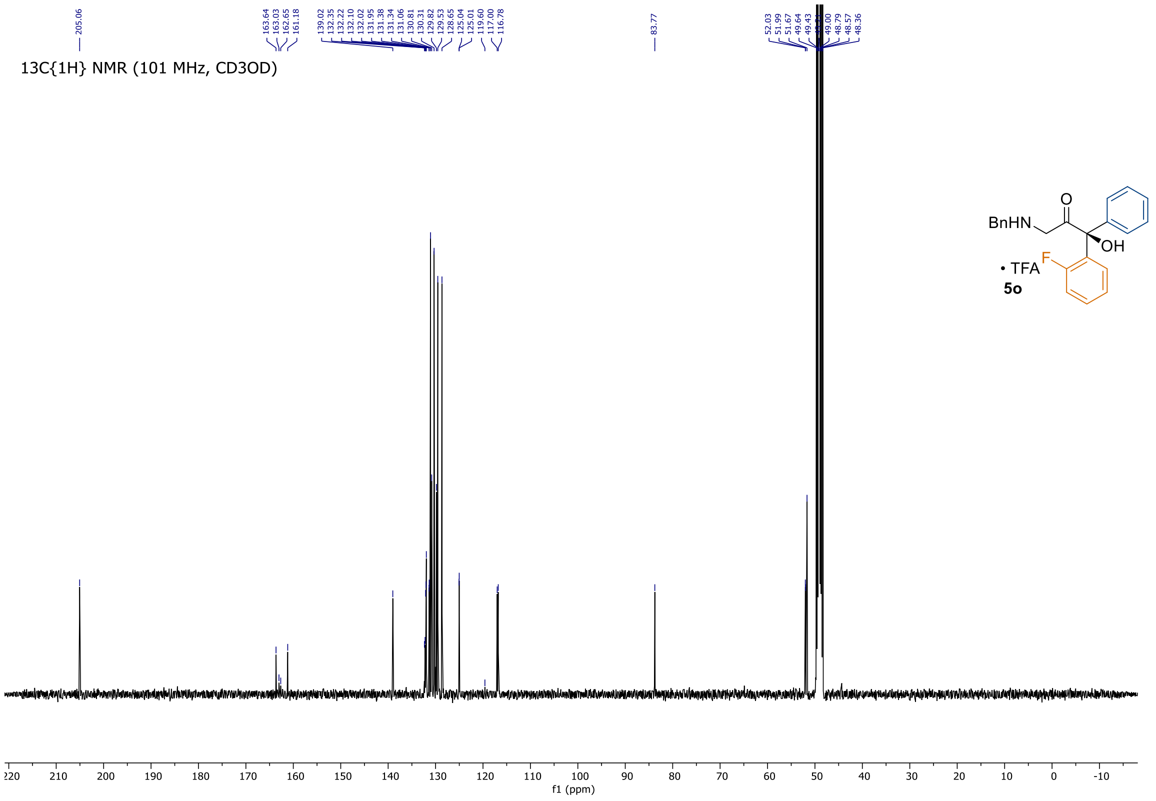

1H NMR (400 MHz, CD3OD)

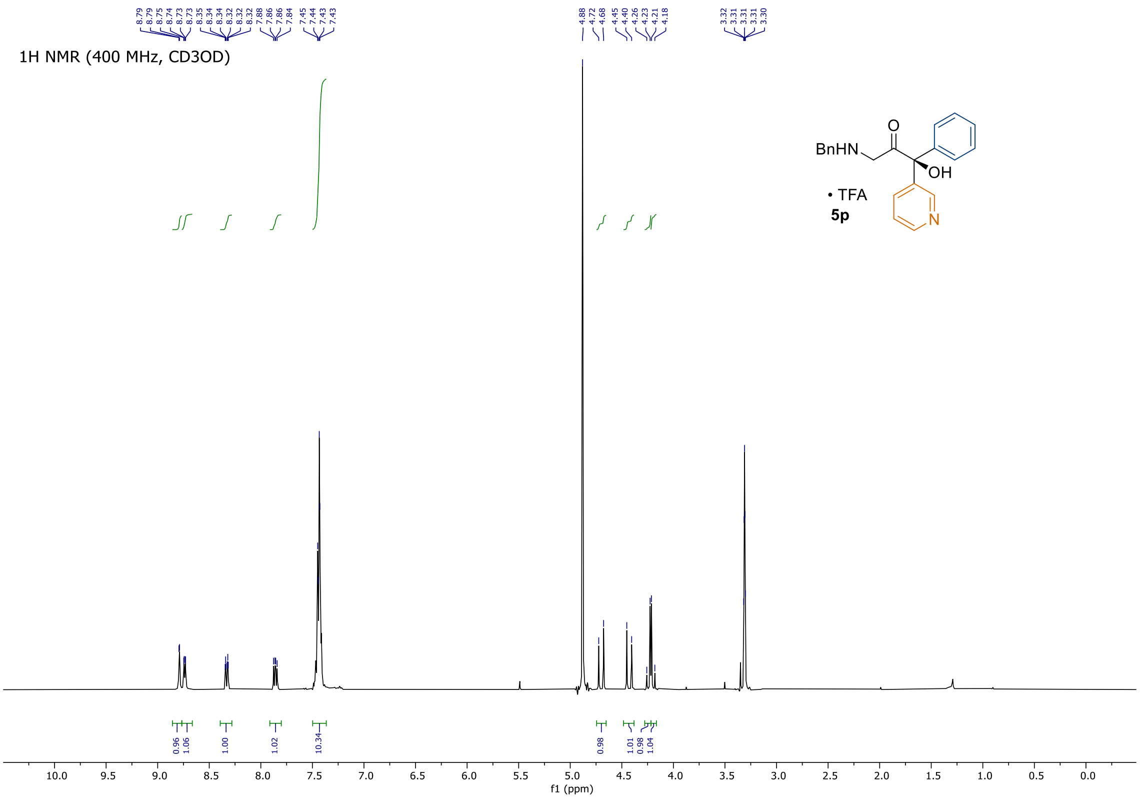

19F NMR (376 MHz, CD3OD)

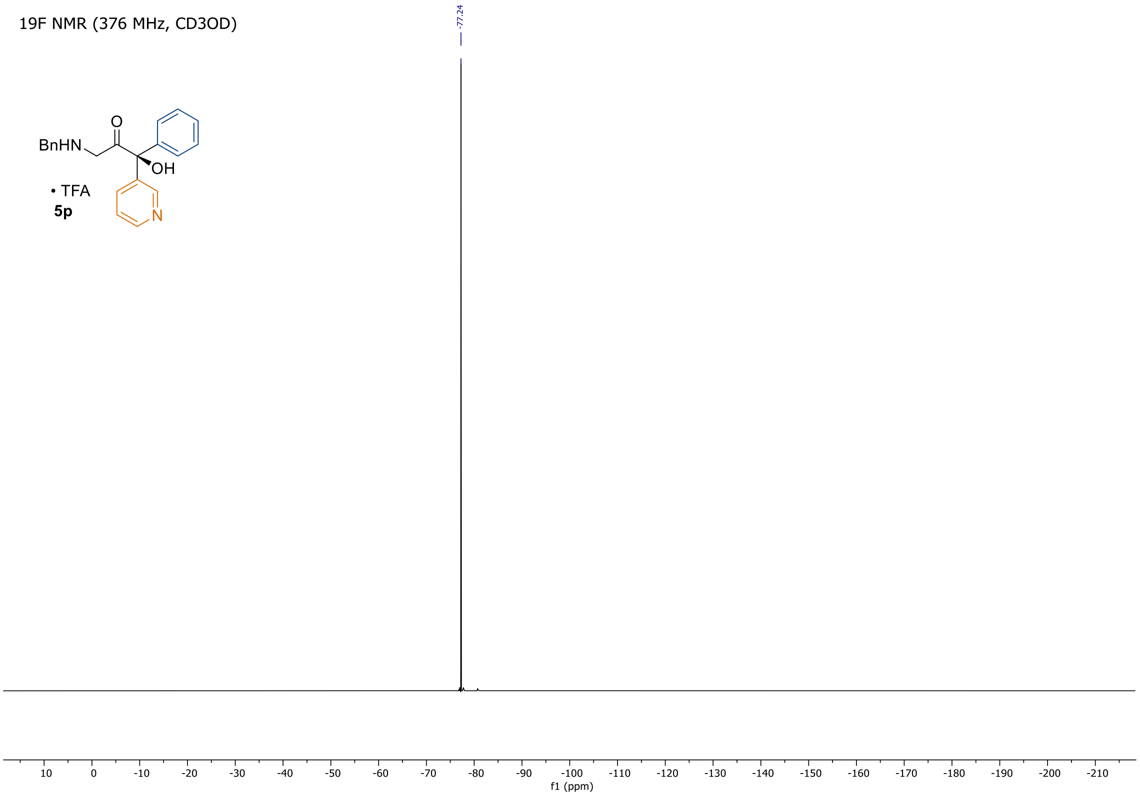

13C{1H} NMR (101 MHz, CD3OD)

• TFA
5p

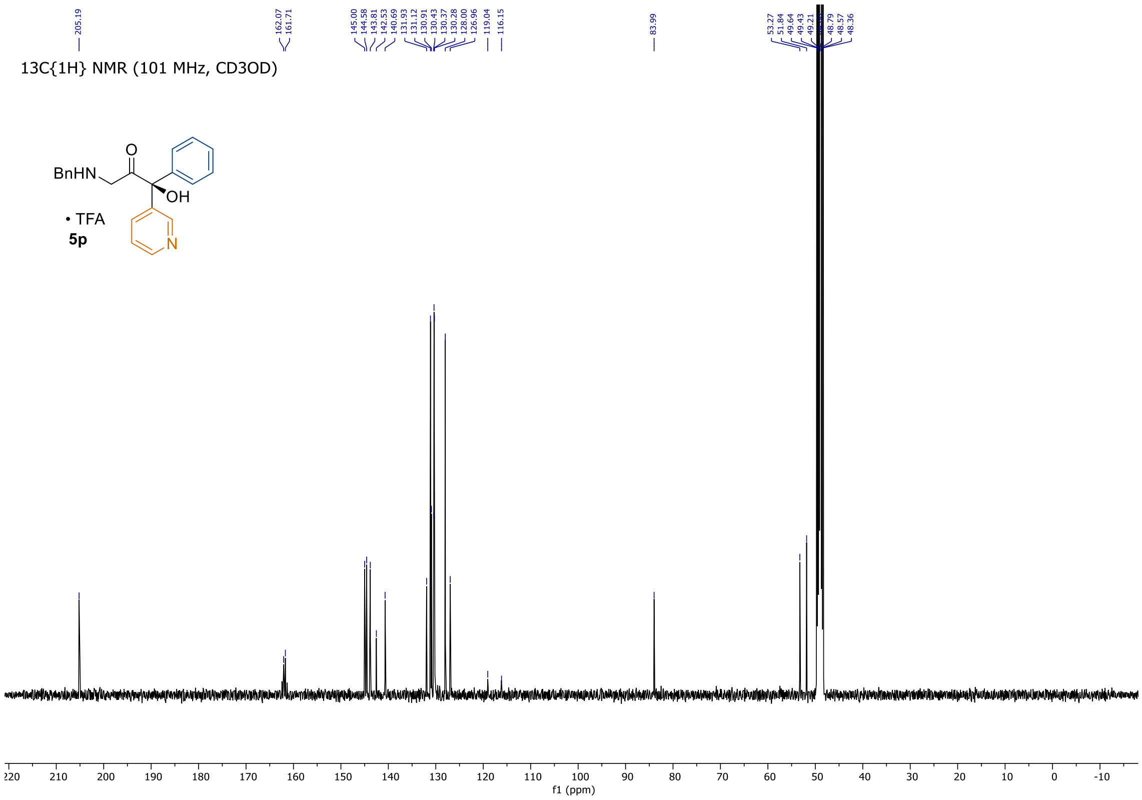

G.3.Product modifications

1H NMR (400 MHz, CDCl3)

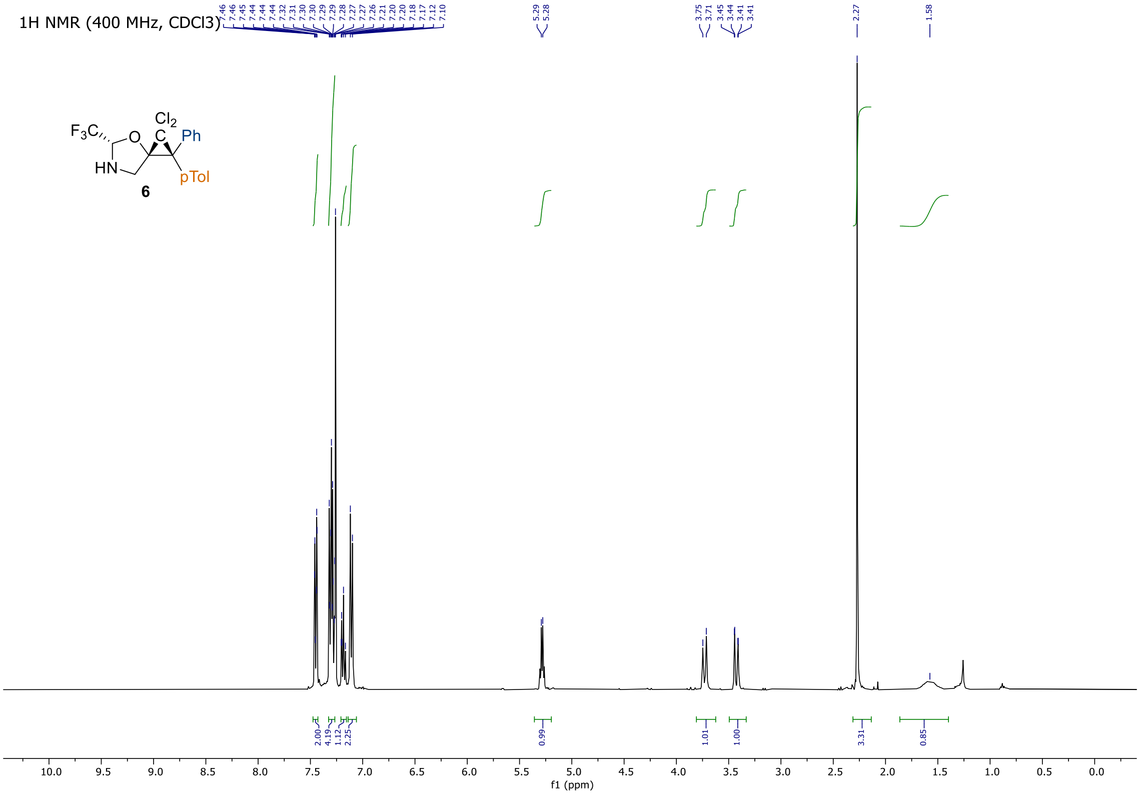

19F NMR (376 MHz, CDCl3)

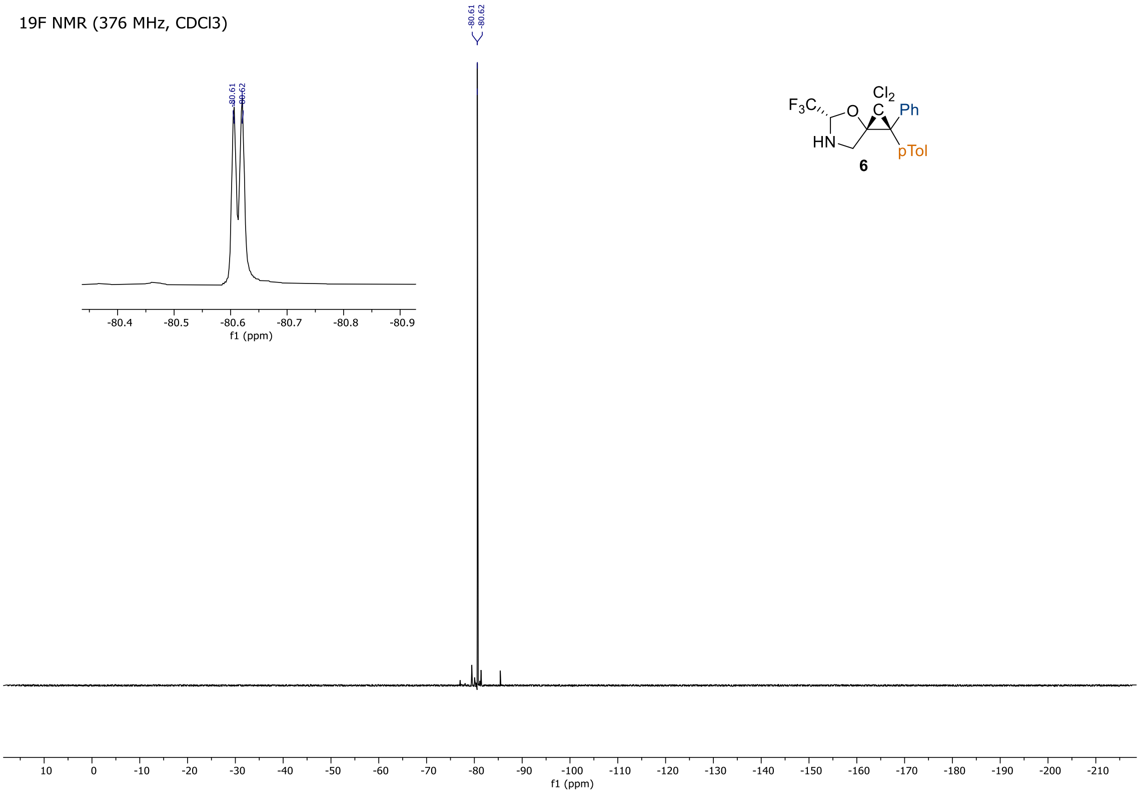

13C{1H} NMR (101 MHz, CDCl3)

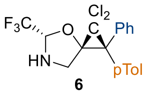

6

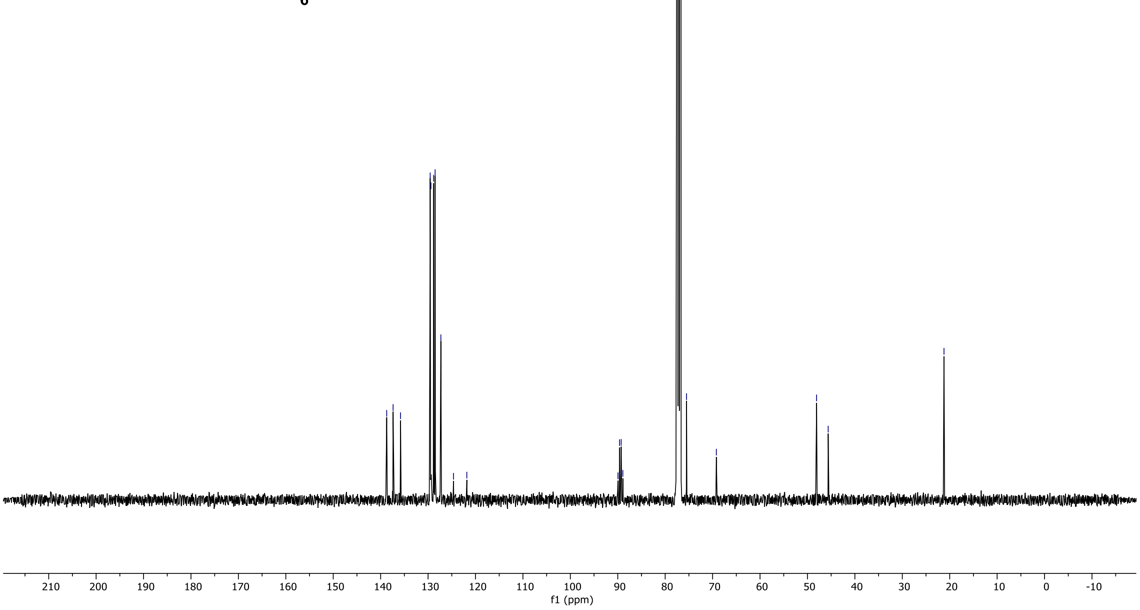

Product **7**•TFA after solvolysis with TFA/HFIP

1H NMR (400 MHz, CDCl3)

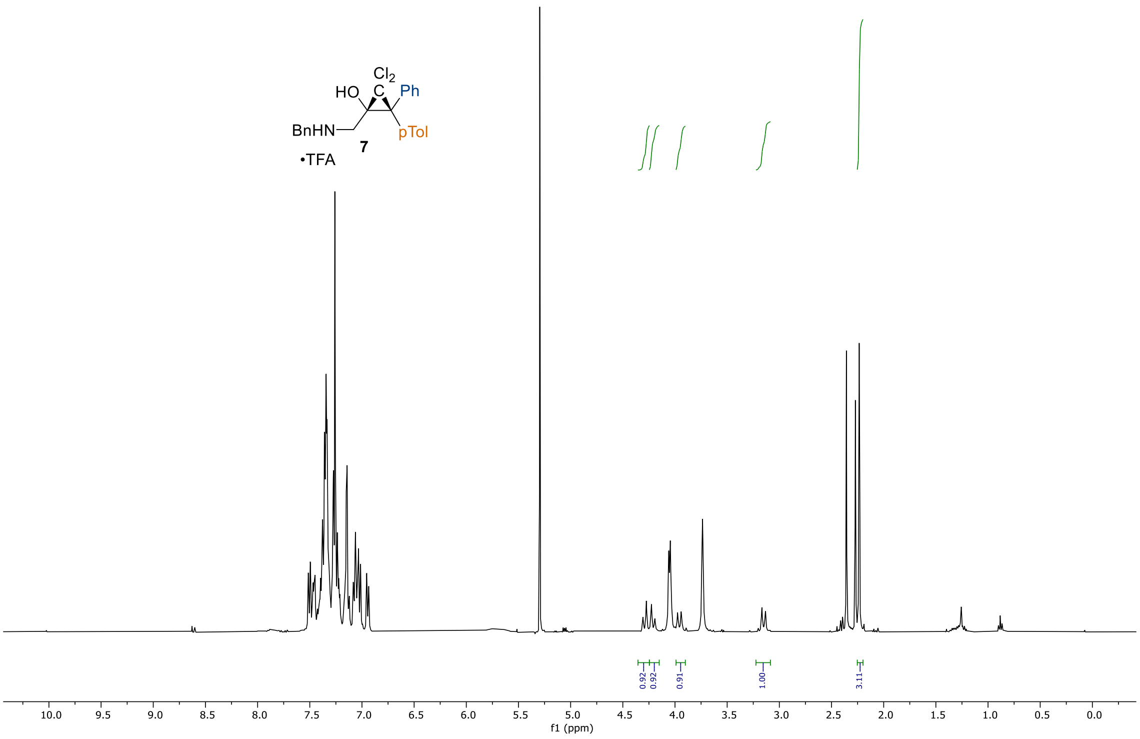

13C{1H} NMR (101 MHz, CDCl3)

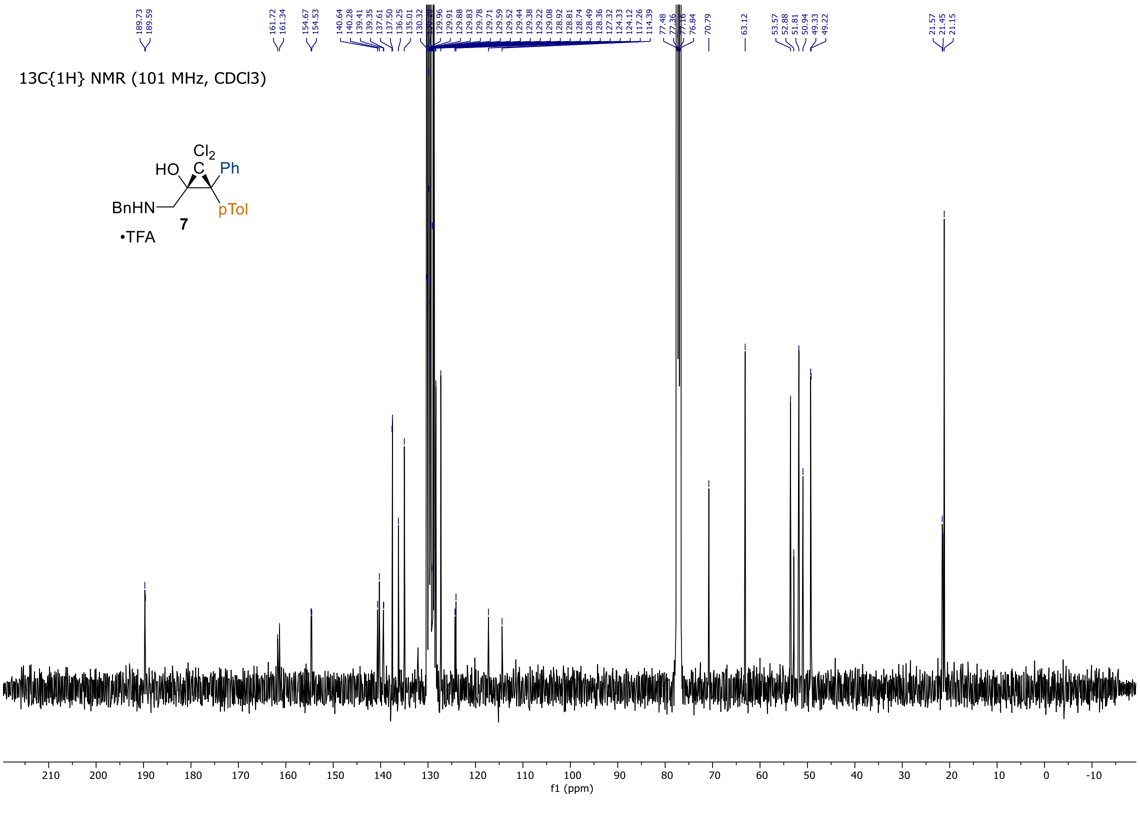

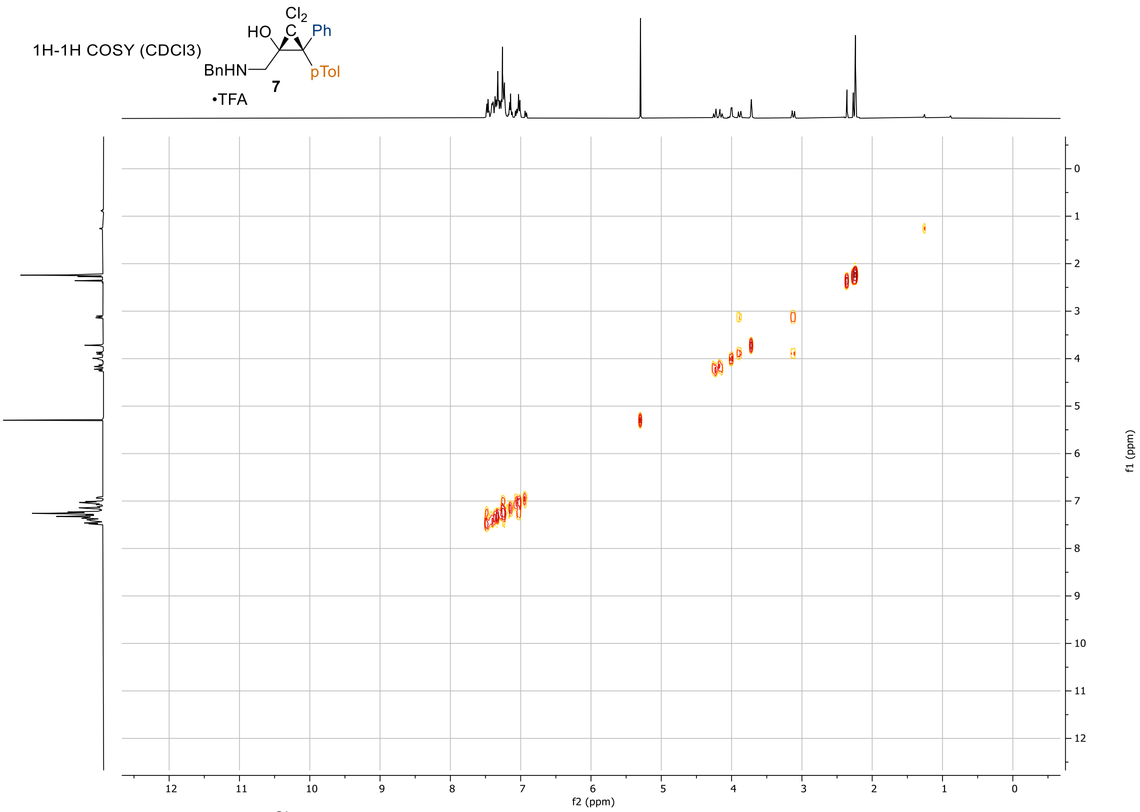
1H-1H COSY (CDCl3)
Cl2
HO
C
Ph
BnHN
pTol
7
•TFA
f2 (ppm)
f1 (ppm)

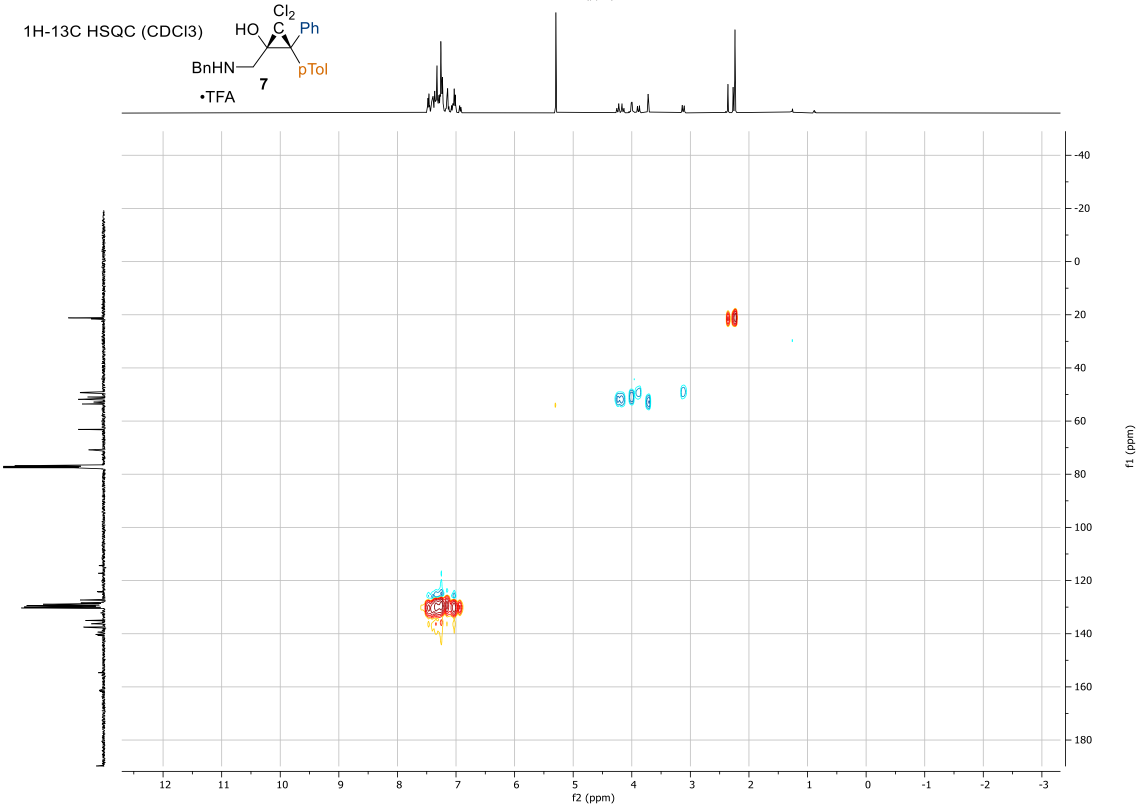
1H-13C HSQC (CDCl3)
Cl2
HO
C
Ph
BnHN
pTol
7
•TFA
f2 (ppm)
f1 (ppm)

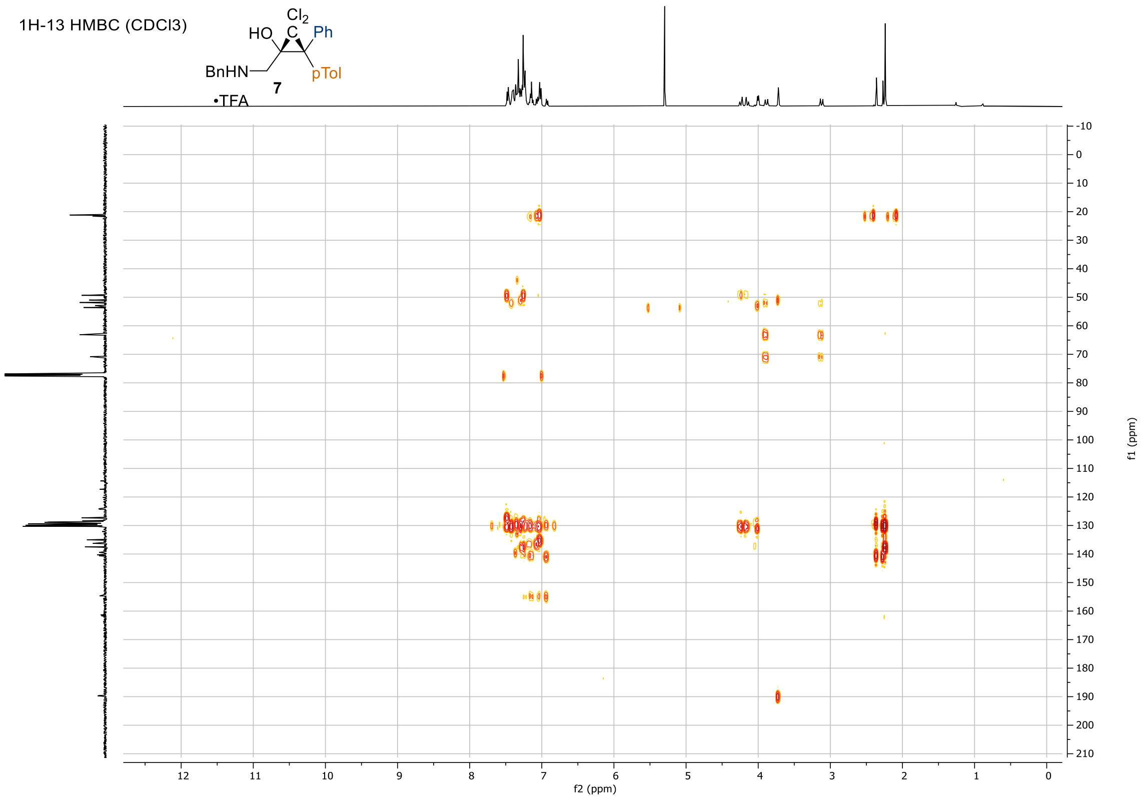

Product **7**•TFA after solvolysis with TFA/HFIP after 16h in CD3OD

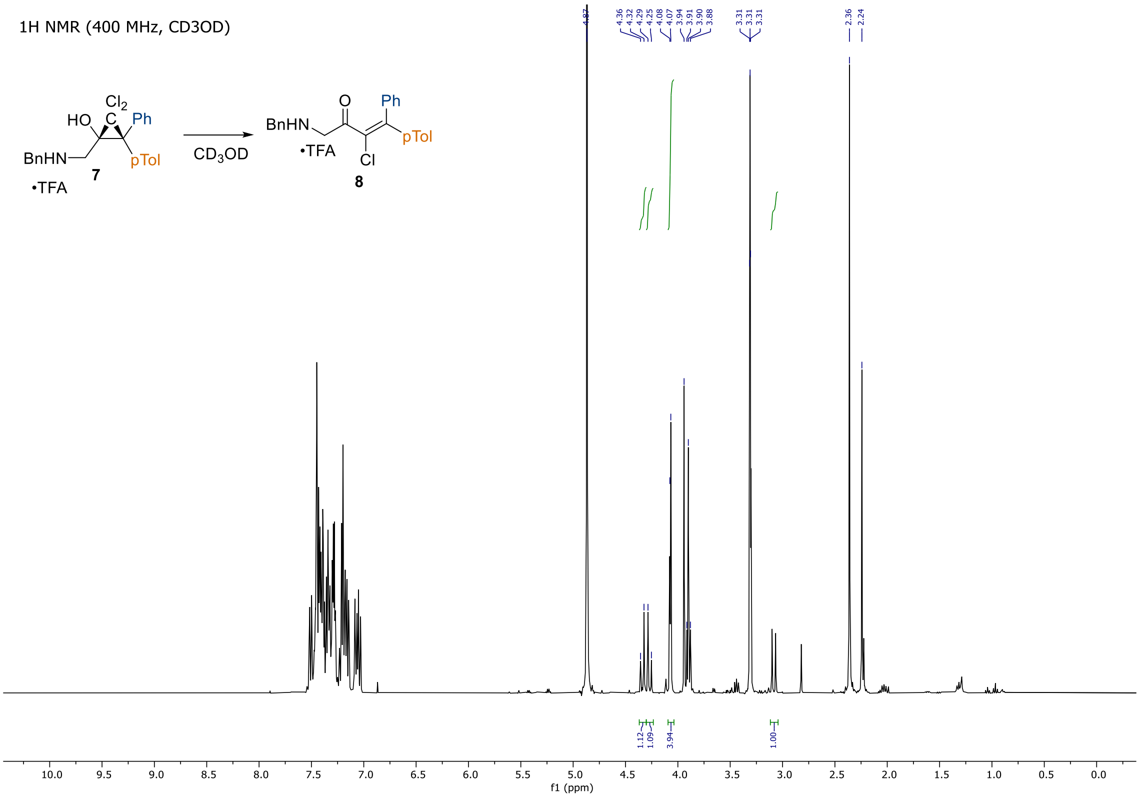

Product **7**•TFA→**8•TFA** after solvolysis with TFA/HFIP after 96h in CD_3OD

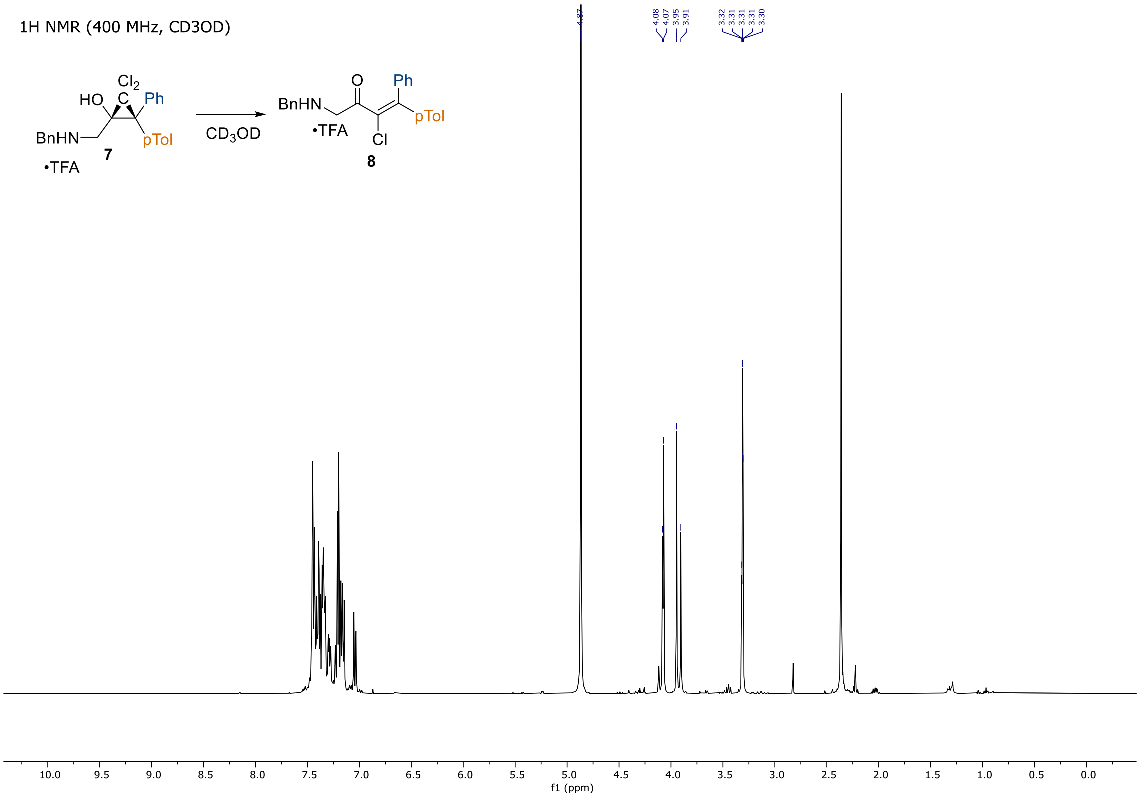

Product **8•TFA** obtained from 8•HCl (from the 1 step procedure) by filtration through $NaHCO_3$ with direct quenching with TFA

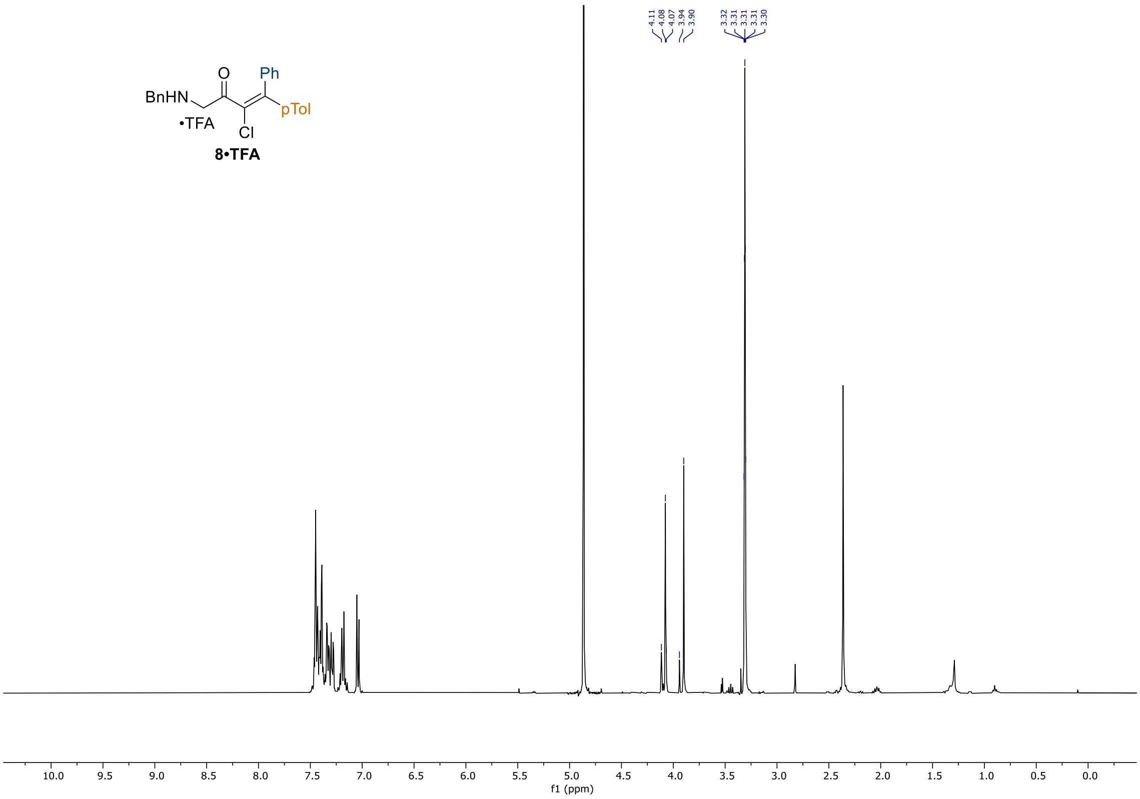

Product **8•HCl** obtained from the 1 step procedure.

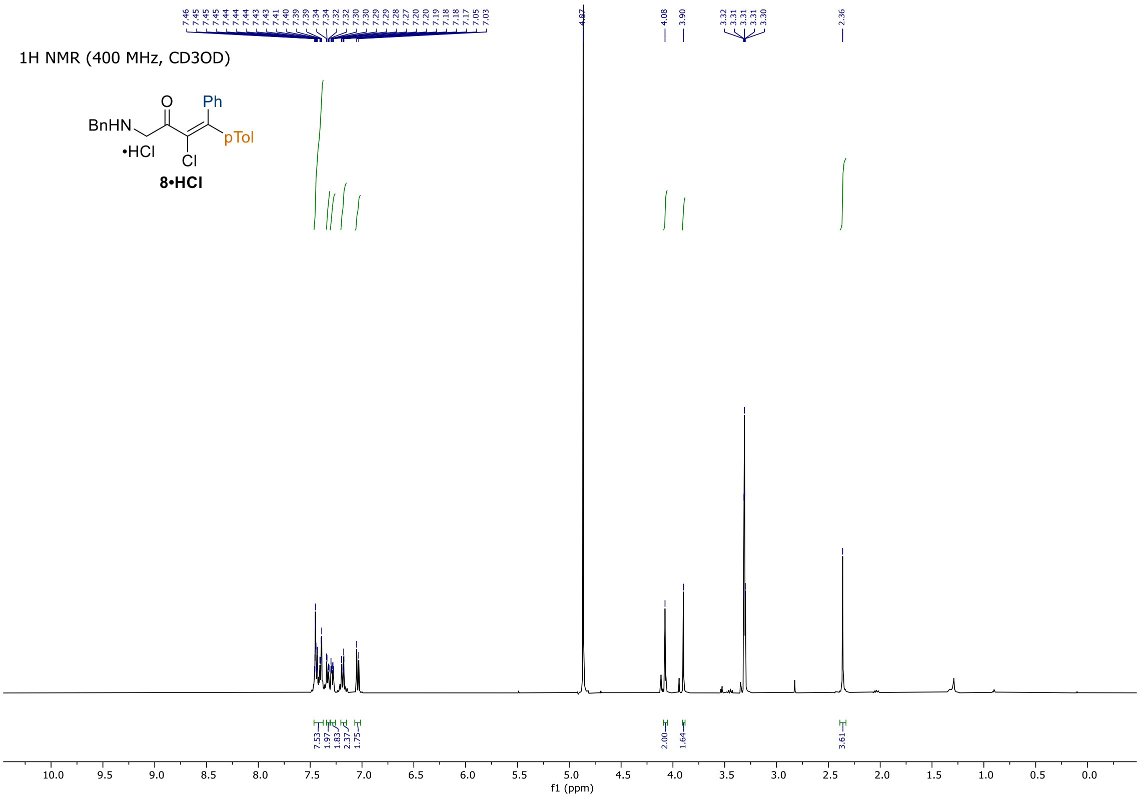

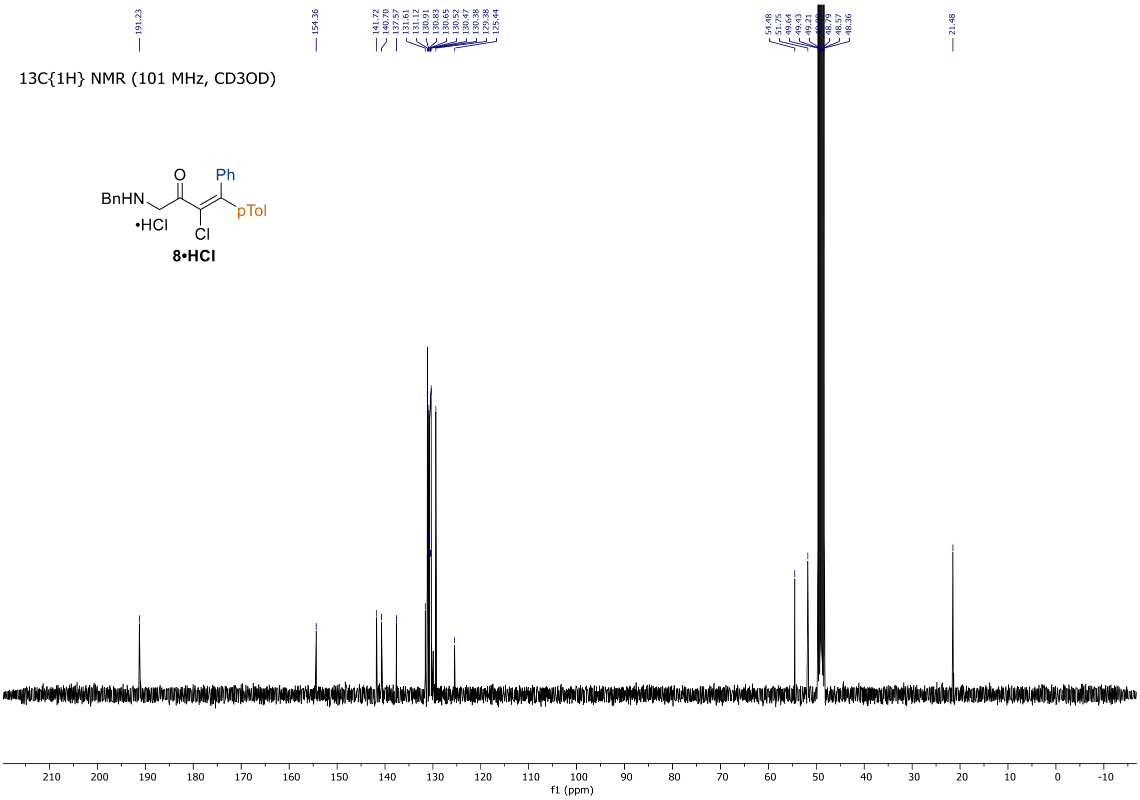

1H-1H COSY (CDCl3)

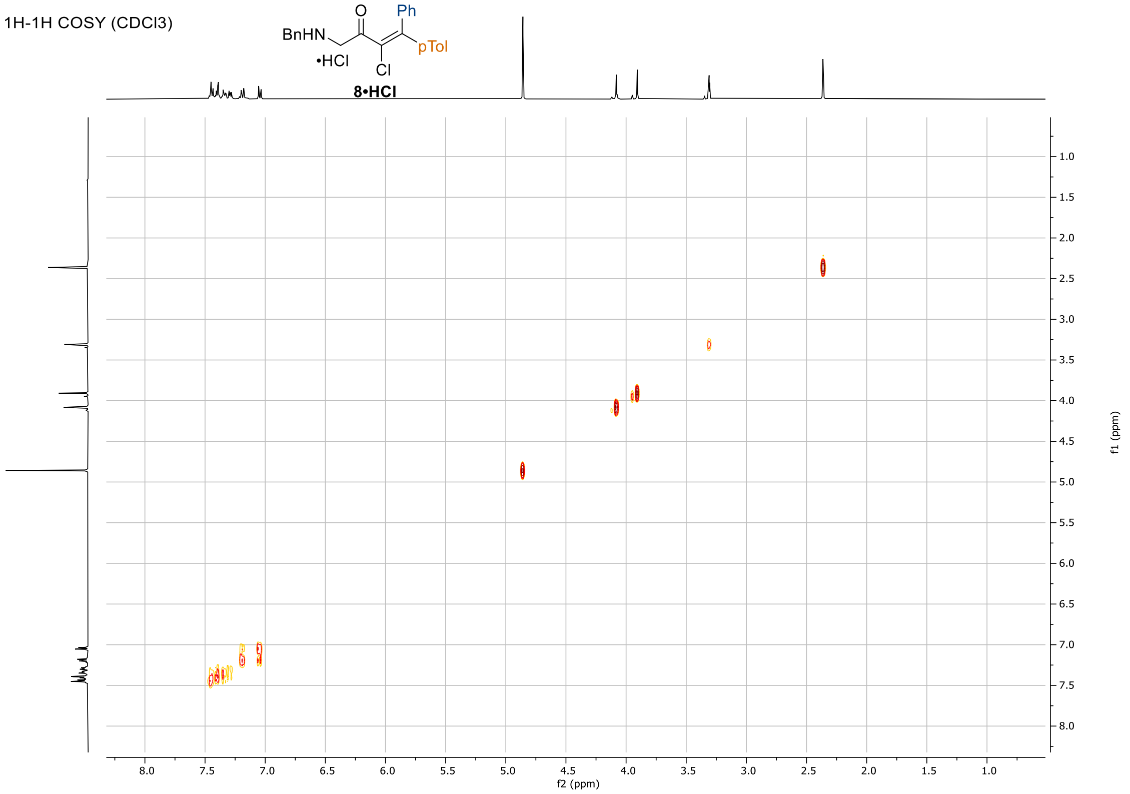

1H-13C HSQC (CDCl3)

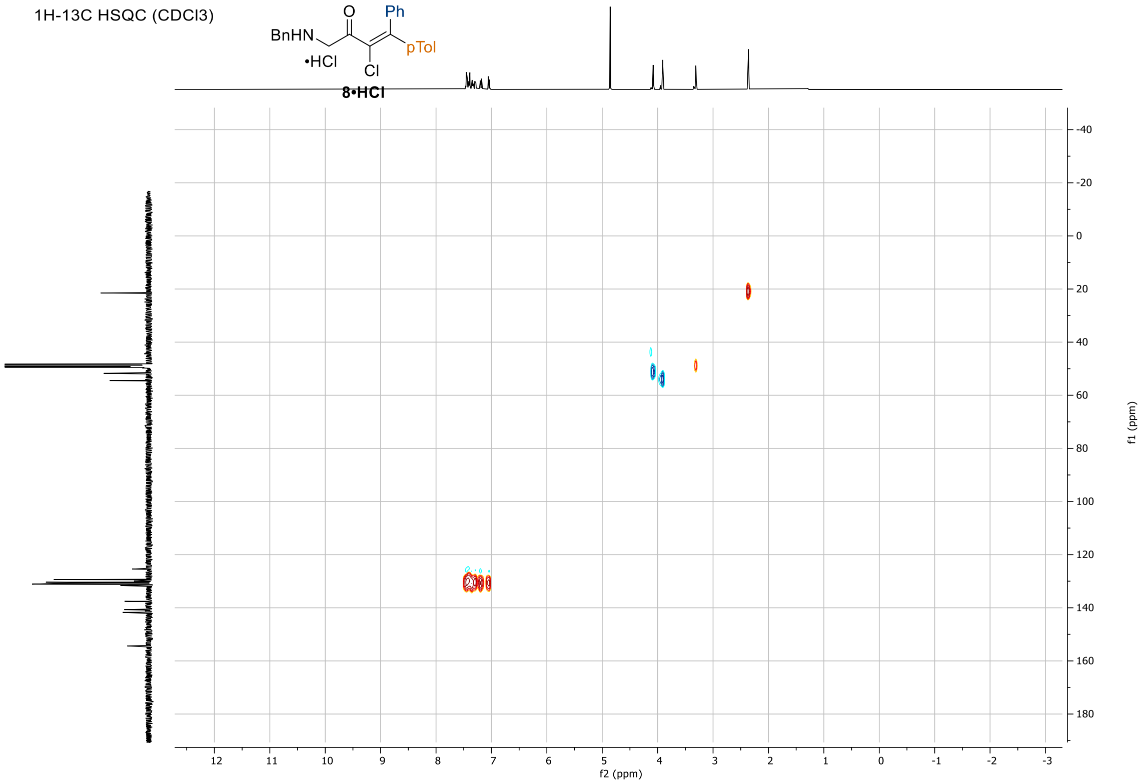

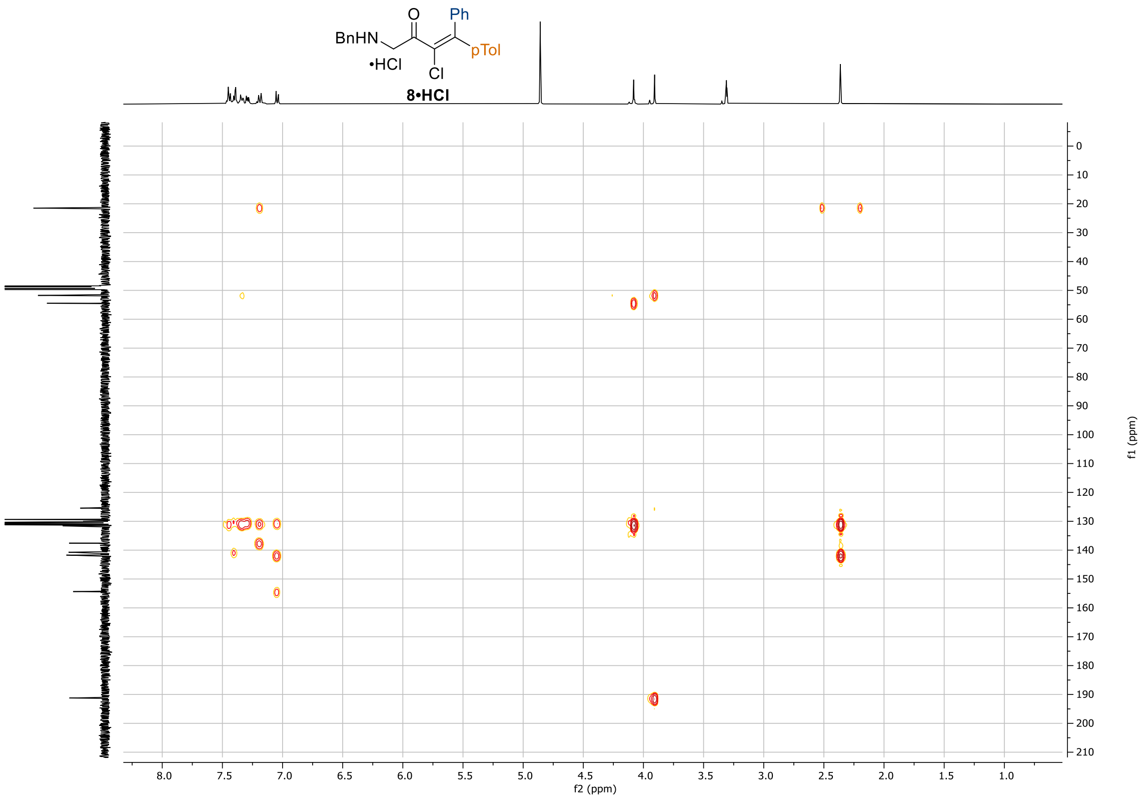
BnHN
O
Ph
pTol
•HCl
Cl
8•HCl
f2 (ppm)
f1 (ppm)

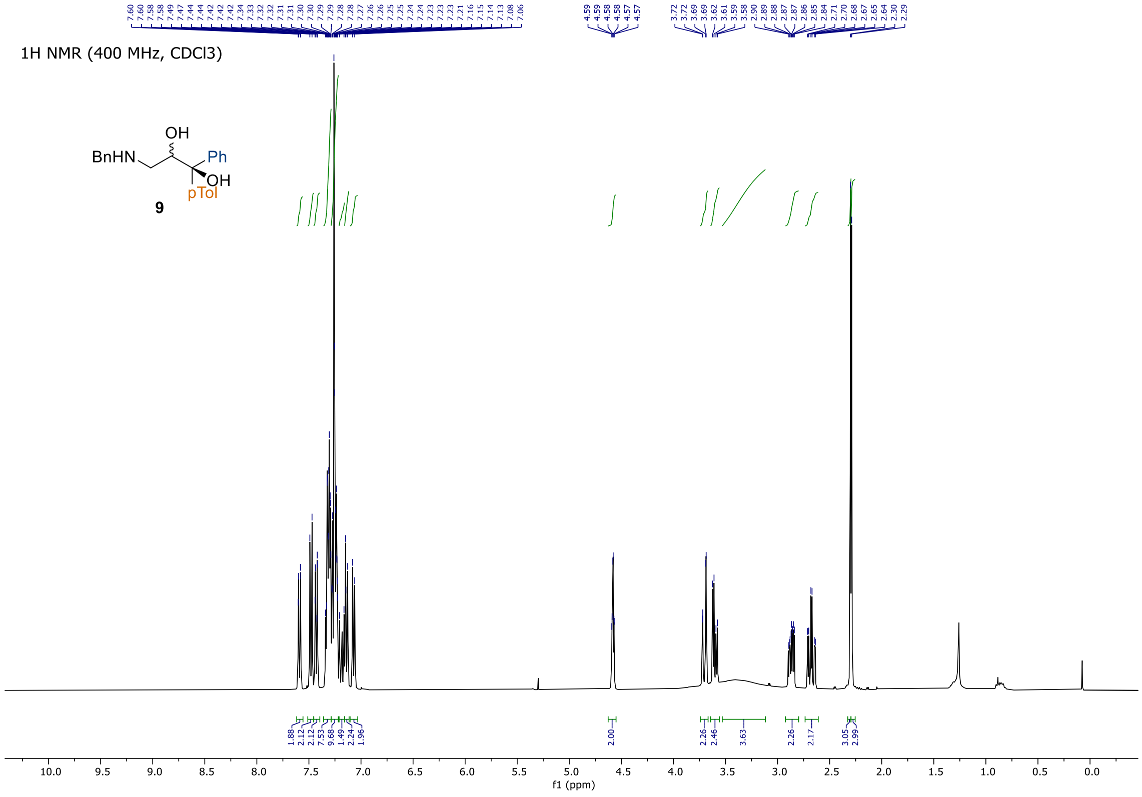
1H NMR (400 MHz, CDCl3)
BnHN
OH
Ph
OH
pTol
9
f1 (ppm)

13C{1H} NMR (101 MHz, CDCl3)

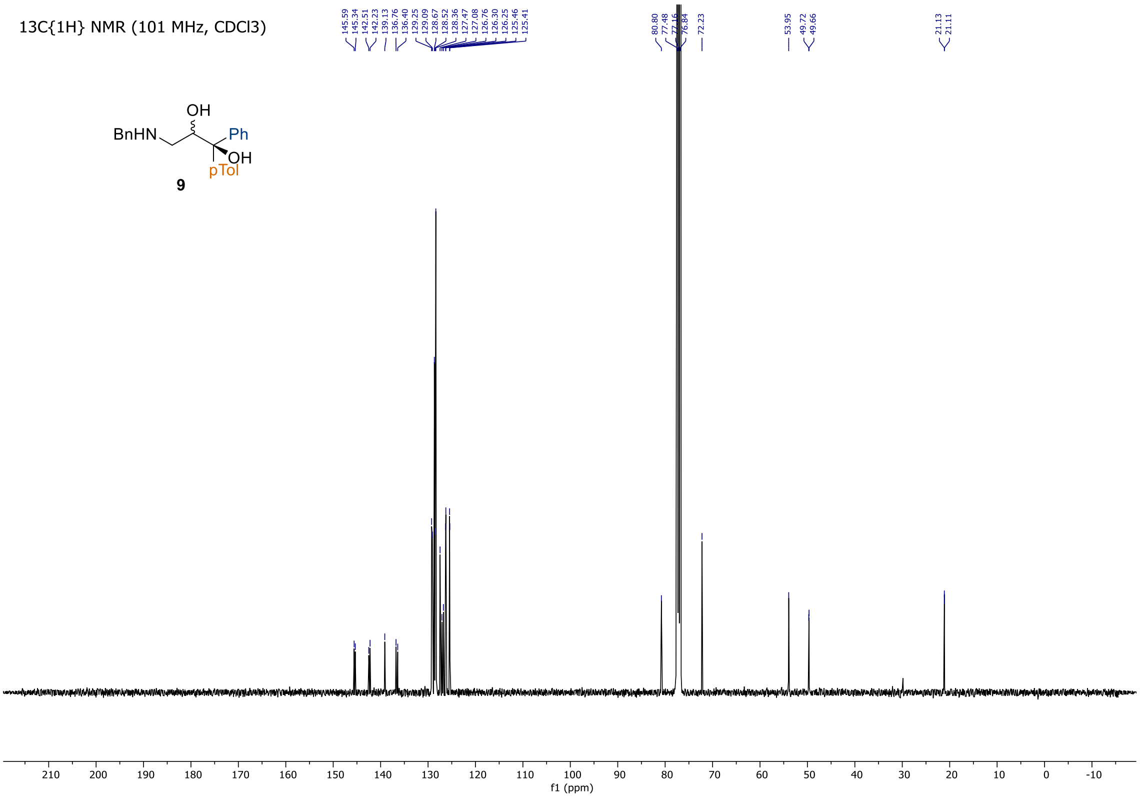

1H-1H COSY (CDCl3)

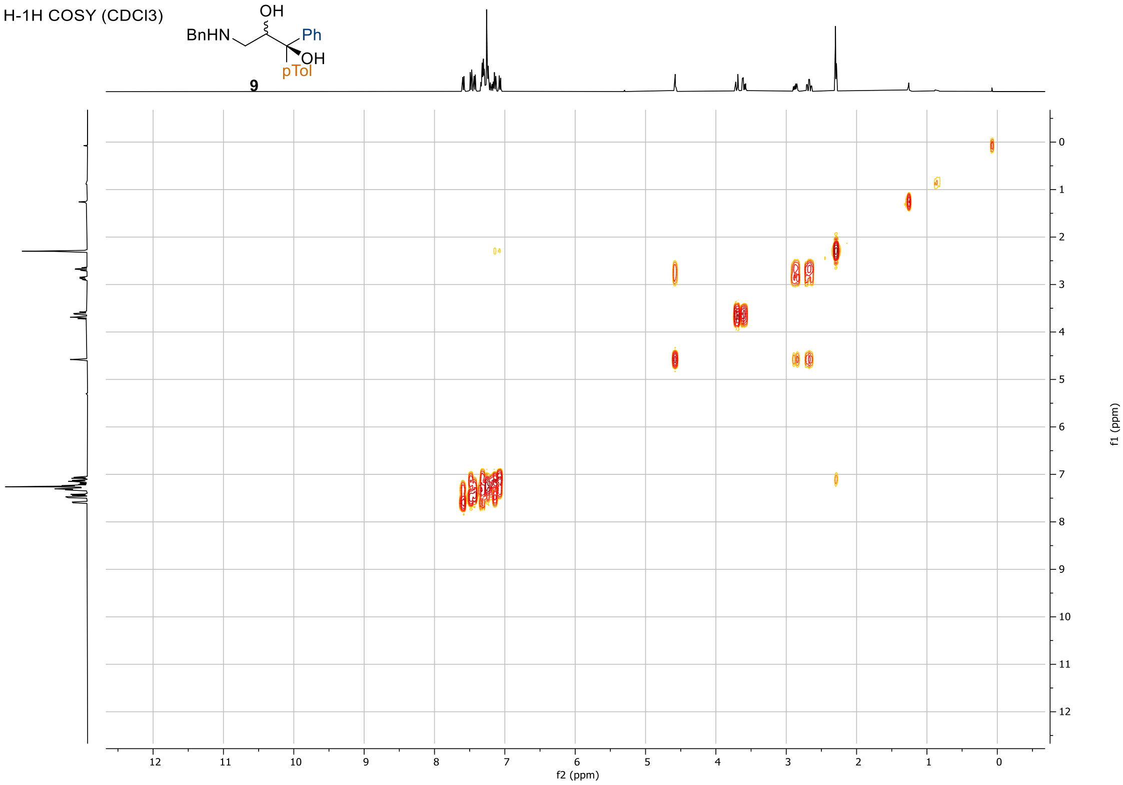

1H-13C HSQC (CDCl3)

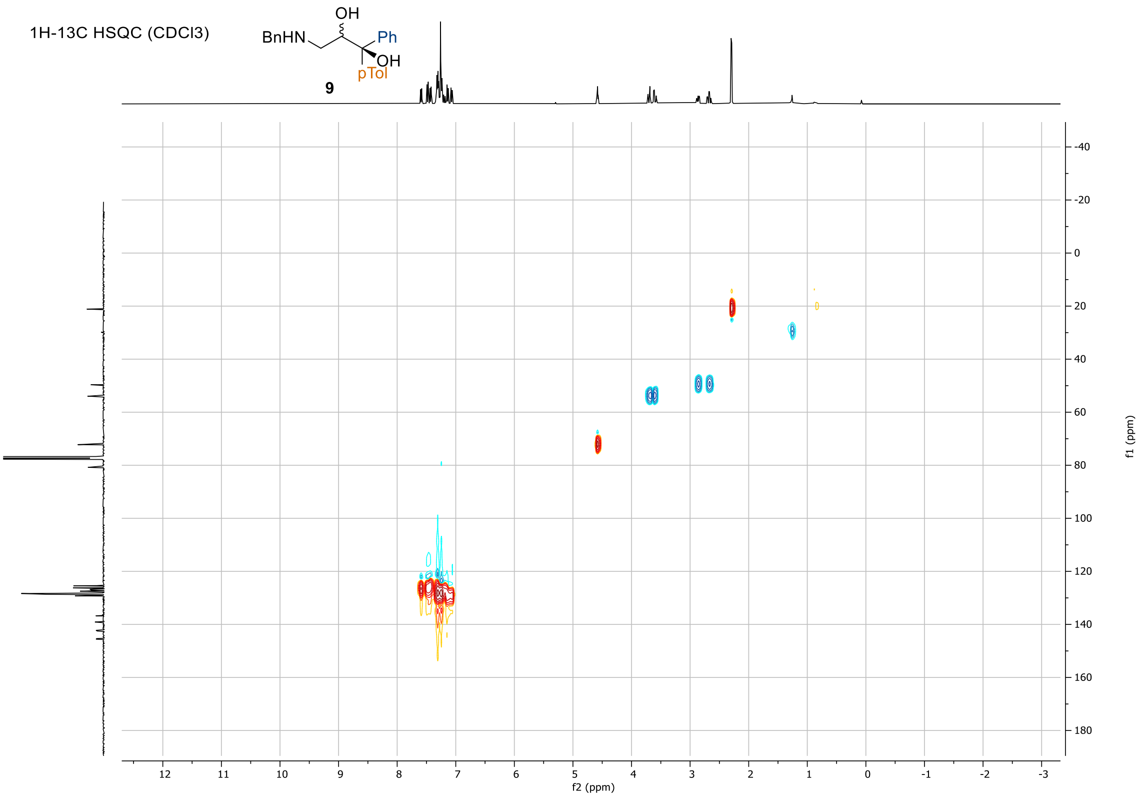

1H-13C HMBC (CDCl3)

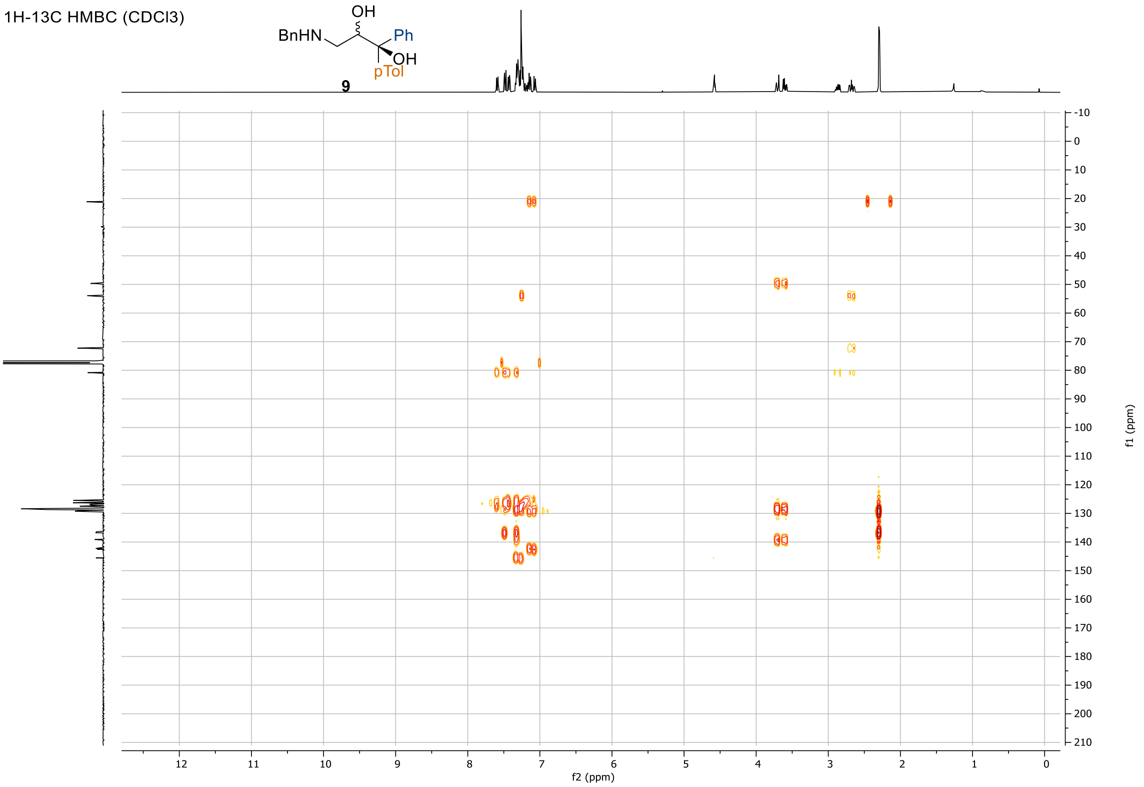

1H NMR (400 MHz, CDCl3)

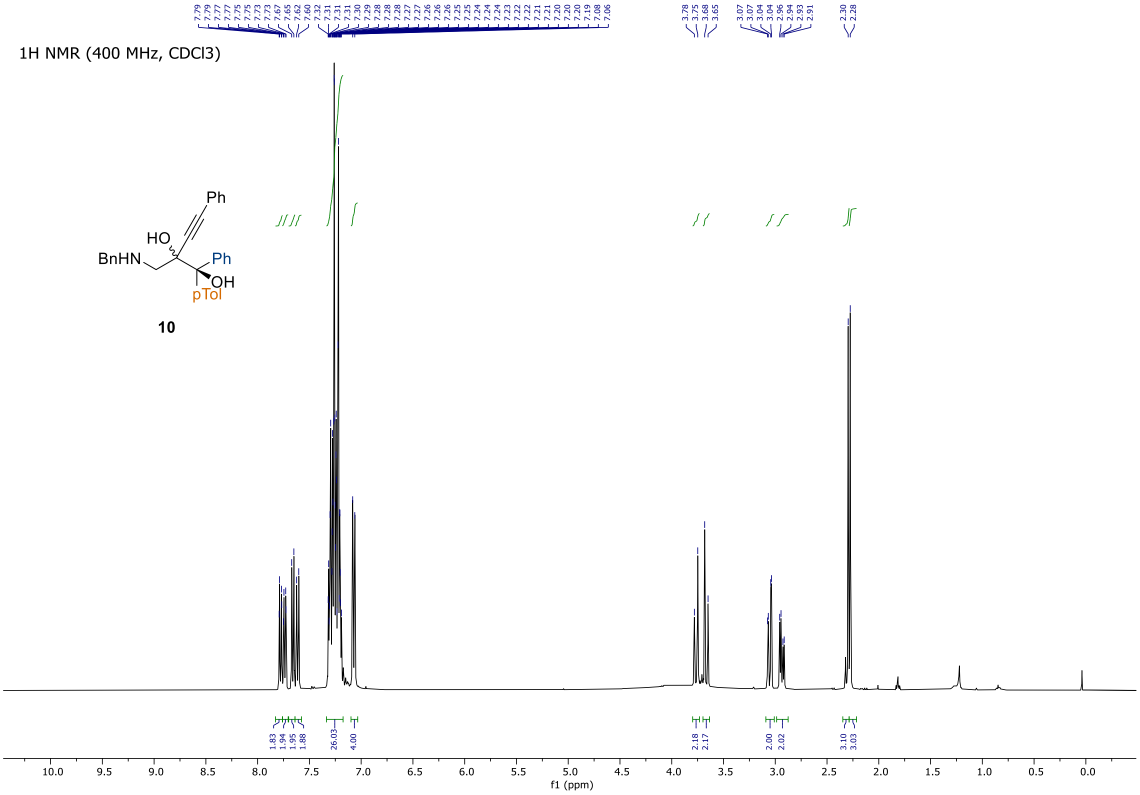

13C{1H} NMR (101 MHz, CDCl3)

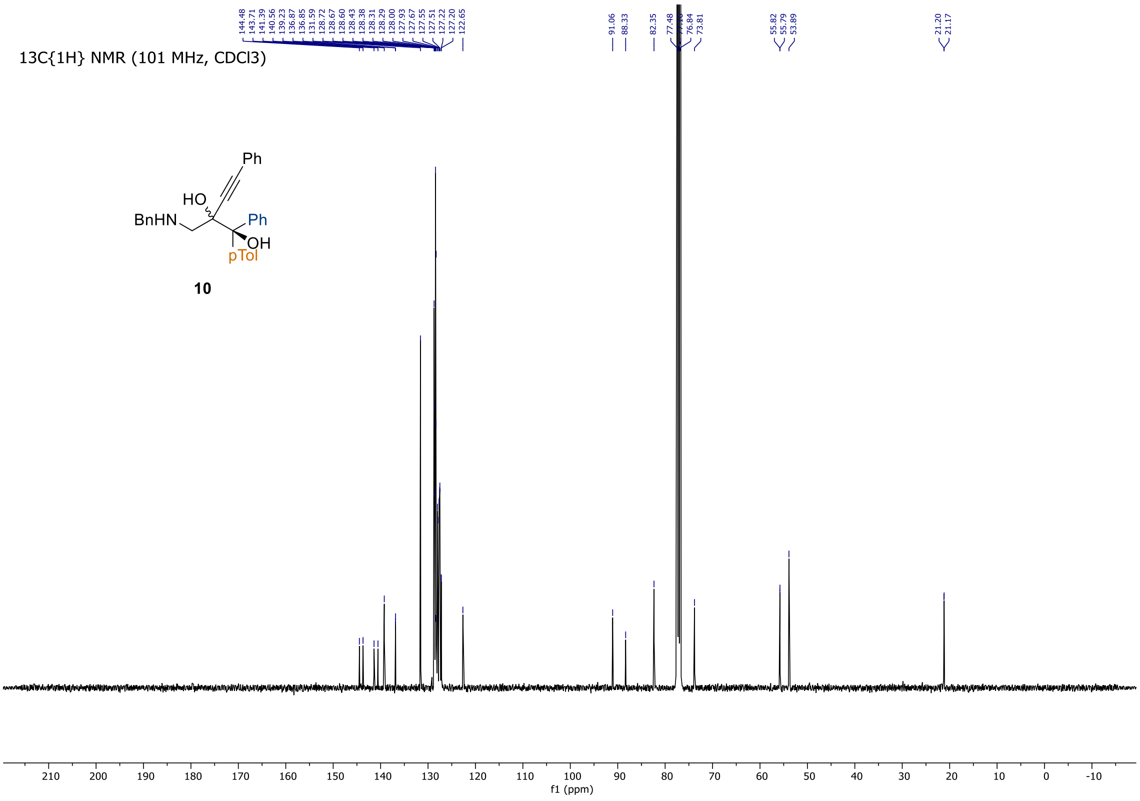

1H-1H COSY (CDCl3)

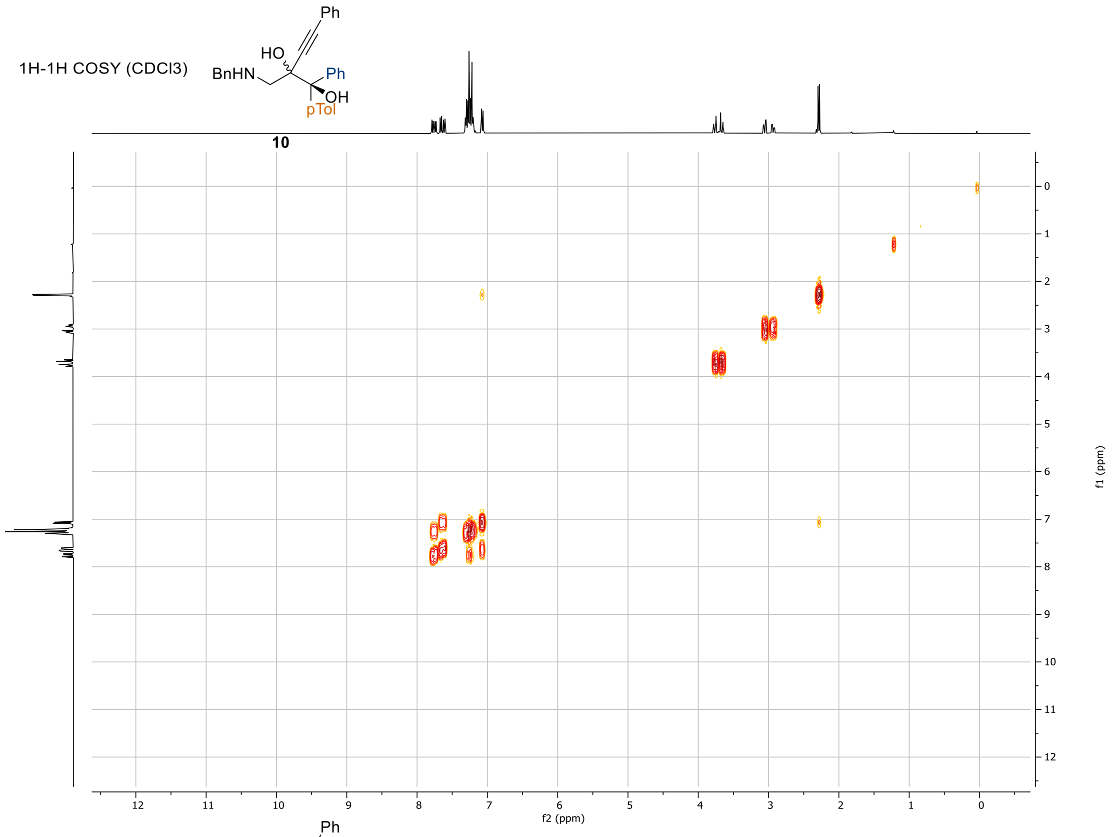

1H-13C HSQC (CDCl3)

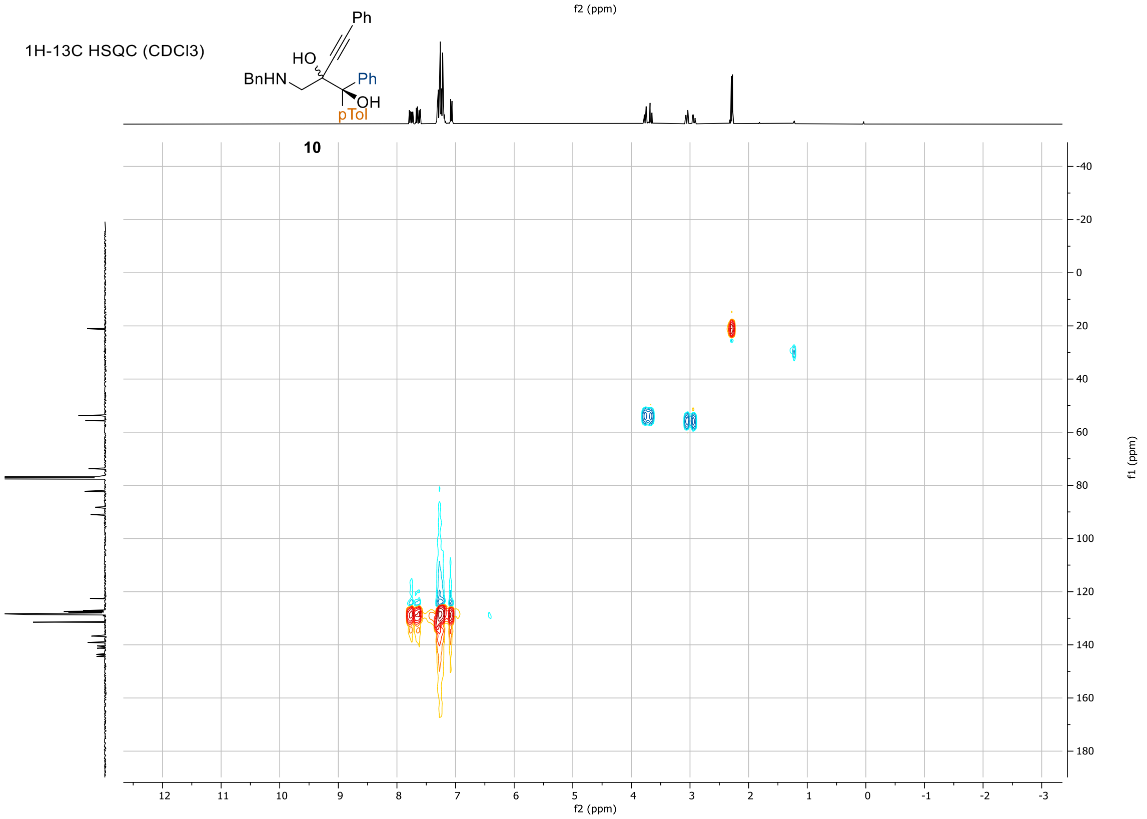

1H-13C HMBC (CDCl3)

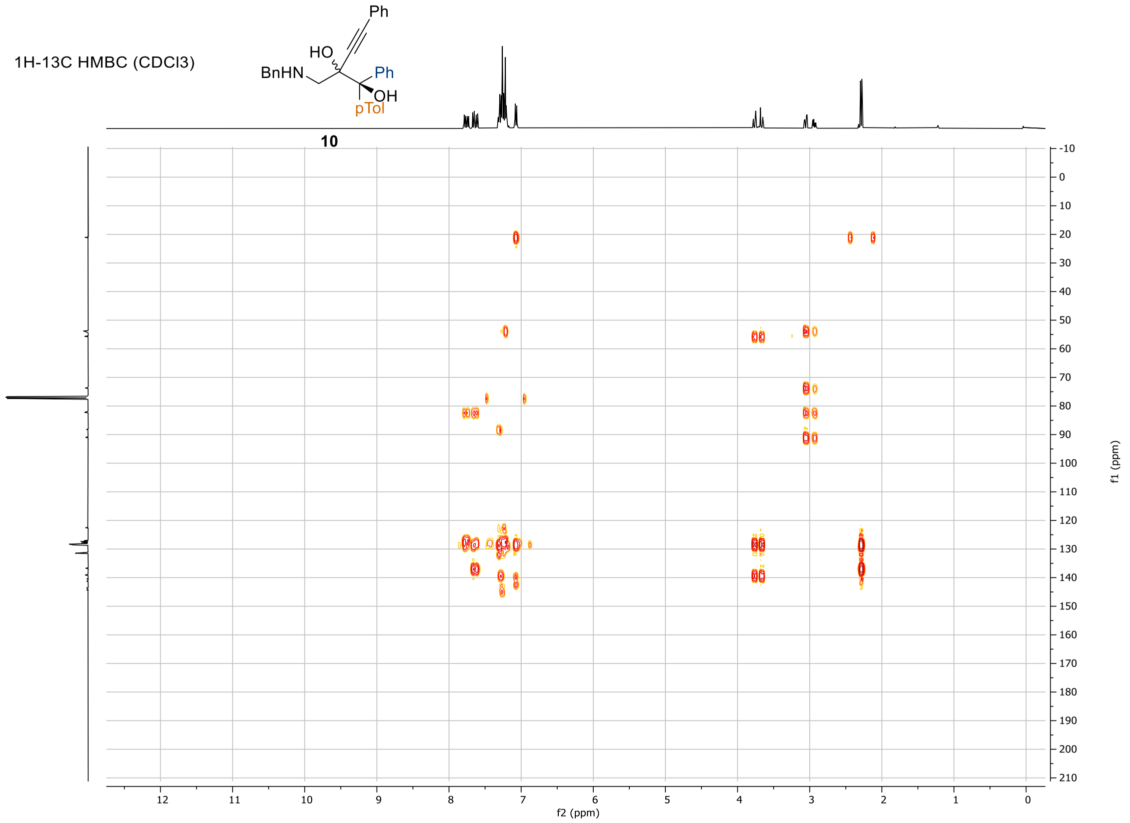

H. HPLC Traces

H.1.Starting materials

Chiral HPLC Daicel Chiralpak IB N-5 column: 99:1 hexane/IPA, flow rate 1 mL/min, λ = 230 nm

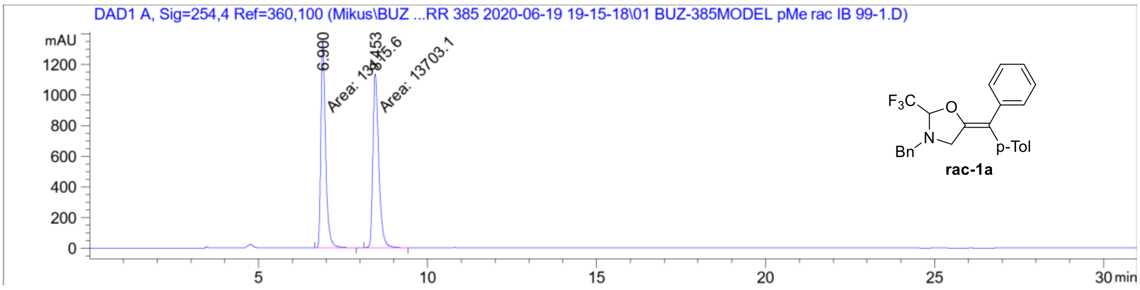

Peak #	RetTime [min]	Type	Width [min]	Area [mAU*s]	Height [mAU]	Area %
1	6.900	MM	0.1627	1.31156e4	1343.72034	48.9047
2	8.453	MM	0.2014	1.37031e4	1133.90173	51.0953

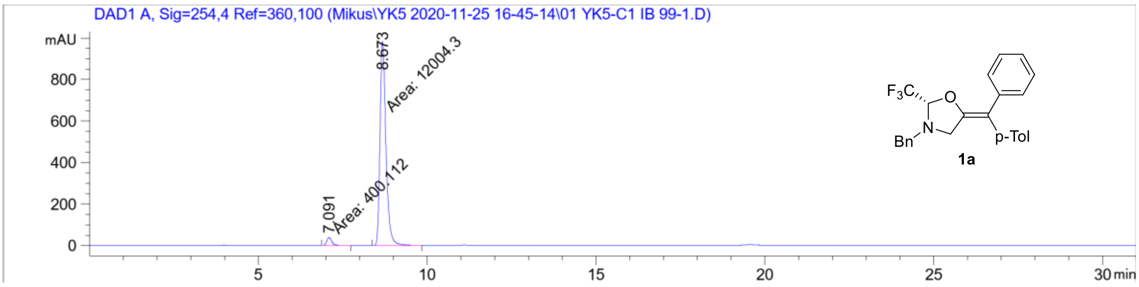

Peak #	RetTime [min]	Type	Width [min]	Area [mAU*s]	Height [mAU]	Area %
1	7.091	MM	0.1685	400.11215	39.57809	3.2256
2	8.673	MM	0.2035	1.20043e4	983.03345	96.7744

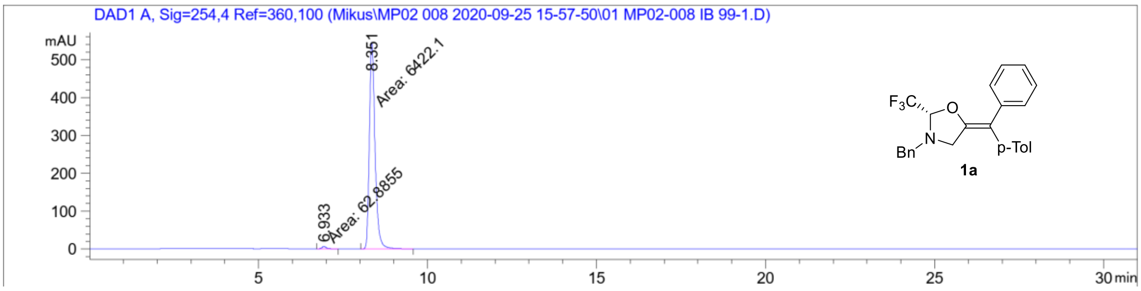

Peak #	RetTime [min]	Type	Width [min]	Area [mAU*s]	Height [mAU]	Area %
1	6.933	MM	0.1694	62.88552	6.18570	0.9697
2	8.351	MM	0.1963	6422.09912	545.20538	99.0303

Chiral HPLC Daicel Chiralpak IB N-5 column: 99:1 hexane/IPA, flow rate 1 mL/min, λ = 254 nm

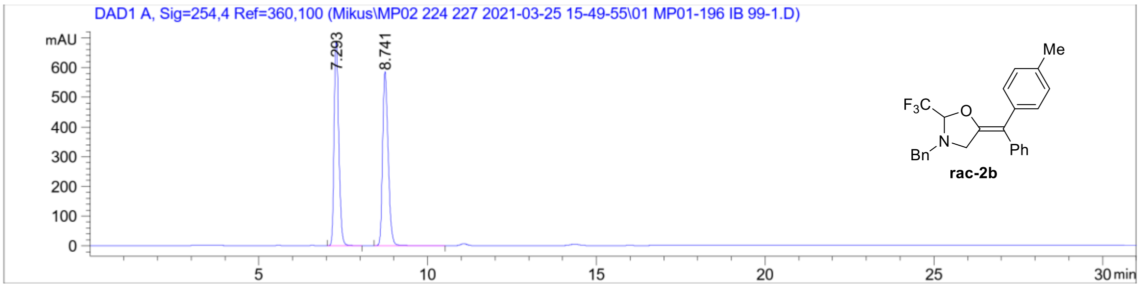

Peak #	RetTime [min]	Type	Width [min]	Area [mAU*s]	Height [mAU]	Area %
1	7.293	BB	0.1499	6591.32129	687.27197	49.6918
2	8.741	BB	0.1769	6673.09521	585.69263	50.3082

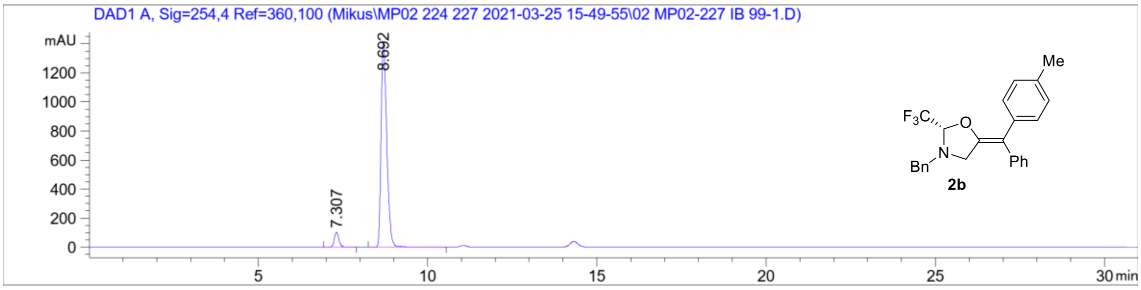

Peak #	RetTime [min]	Type	Width [min]	Area [mAU*s]	Height [mAU]	Area %
1	7.307	BB	0.1478	970.13416	101.24650	5.5612
2	8.692	BB	0.1782	1.64745e4	1411.45386	94.4388

Chiral HPLC Daicel Chiralpak IB N-5 column: 99:1 hexane/IPA, flow rate 1 mL/min, λ = 254 nm

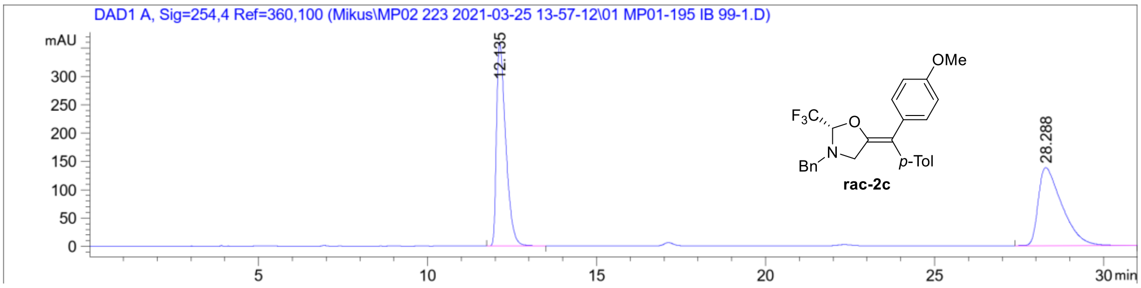

Peak #	RetTime [min]	Type	Width [min]	Area [mAU*s]	Height [mAU]	Area %
1	12.135	BB	0.2867	6804.98145	359.76126	50.3359
2	28.288	BBA	0.7251	6714.17236	137.65378	49.6641

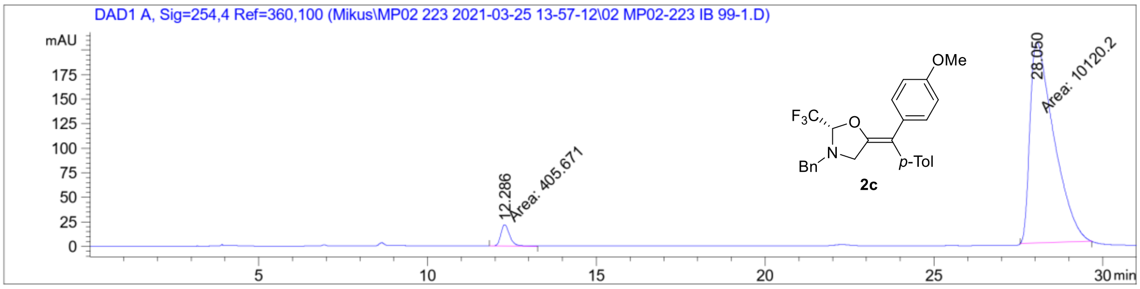

Peak #	RetTime [min]	Type	Width [min]	Area [mAU*s]	Height [mAU]	Area %
1	12.286	MM	0.3087	405.67117	21.90416	3.8540
2	28.050	MM	0.8243	1.01202e4	204.61443	96.1460

Chiral HPLC Daicel Chiralpak IB N-5 column: 99:1 hexane/IPA, flow rate 1 mL/min, λ = 254 nm

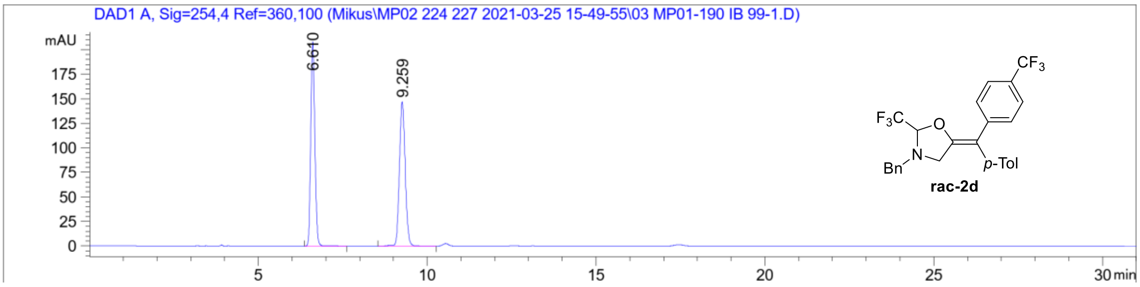

Peak #	RetTime [min]	Type	Width [min]	Area [mAU*s]	Height [mAU]	Area %
1	6.610	BB	0.1296	1740.68494	208.13171	49.7015
2	9.259	BB	0.1858	1761.59131	146.98993	50.2985

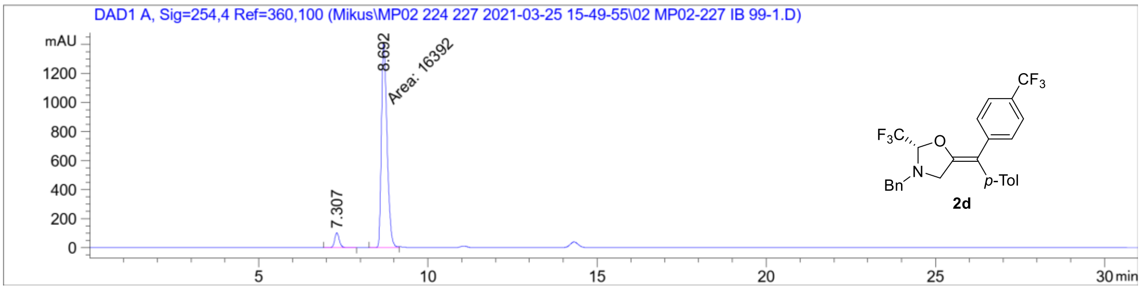

Peak #	RetTime [min]	Type	Width [min]	Area [mAU*s]	Height [mAU]	Area %
1	7.307	BB	0.1478	970.13416	101.24650	5.5876
2	8.692	MM	0.1935	1.63920e4	1411.75427	94.4124

Chiral HPLC Daicel Chiralpak IB N-5 column: 99:1 hexane/IPA, flow rate 1 mL/min, λ = 254 nm

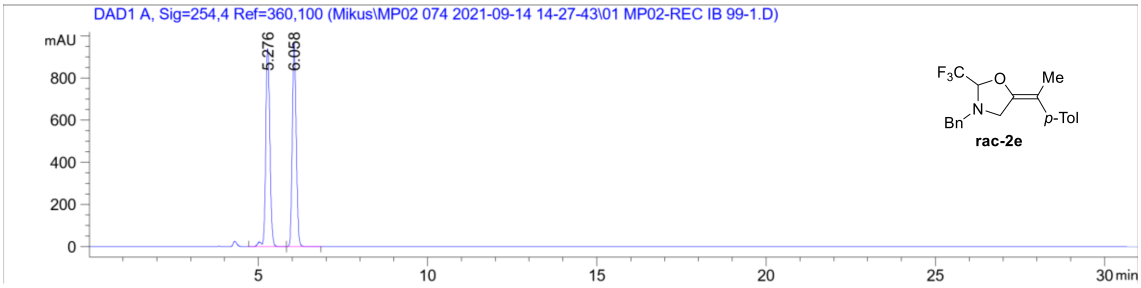

Peak #	RetTime [min]	Type	Width [min]	Area [mAU*s]	Height [mAU]	Area %
1	5.276	VB R	0.1290	7987.86572	940.72534	50.6769
2	6.058	BB	0.1234	7774.46729	971.61096	49.3231

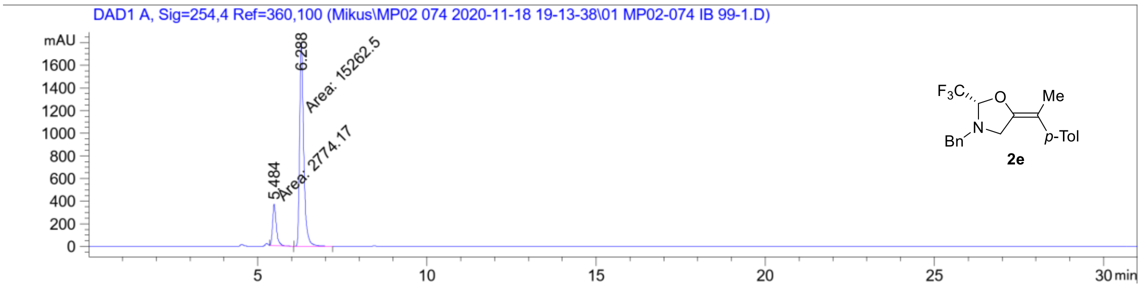

Peak #	RetTime [min]	Type	Width [min]	Area [mAU*s]	Height [mAU]	Area %
1	5.484	MM	0.1266	2774.17139	365.25504	15.3807
2	6.288	MM	0.1409	1.52625e4	1804.83618	84.6193

Chiral HPLC Daicel Chiralpak IB N-5 column: 99:1 hexane/IPA, flow rate 1 mL/min, λ = 254 nm

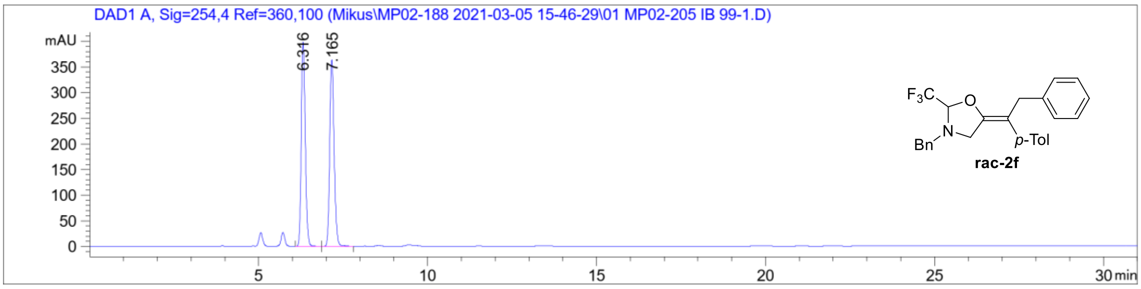

Peak #	RetTime [min]	Type	Width [min]	Area [mAU*s]	Height [mAU]	Area %
1	6.316	BB	0.1274	3258.40723	398.37305	49.8192
2	7.165	BB	0.1393	3282.05151	363.51822	50.1808

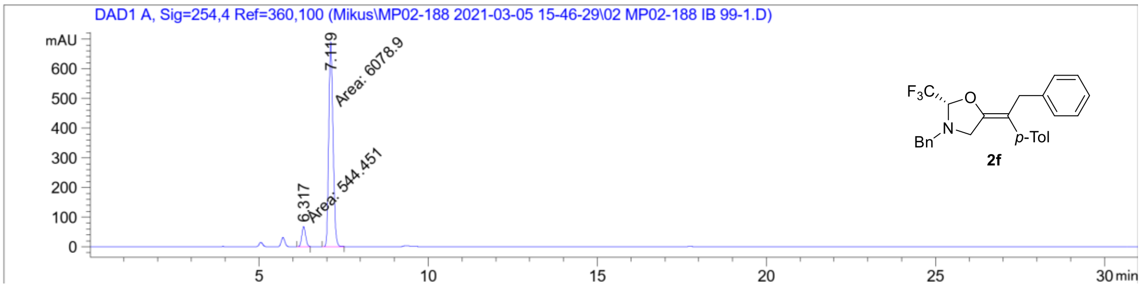

Peak #	RetTime [min]	Type	Width [min]	Area [mAU*s]	Height [mAU]	Area %
1	6.317	MM	0.1327	544.45074	68.36325	8.2202
2	7.119	MM	0.1470	6078.90381	689.19690	91.7798

Chiral HPLC Daicel Chiralpak IB N-5 column: 99:1 hexane/IPA, flow rate 1 mL/min, λ = 254 nm

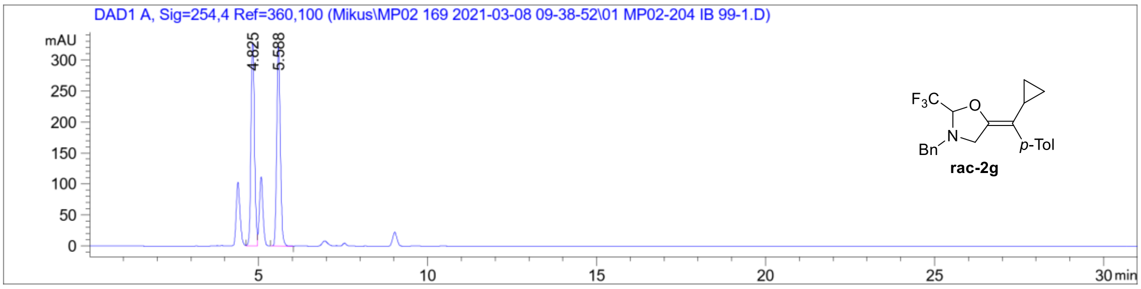

Peak #	RetTime [min]	Type	Width [min]	Area [mAU*s]	Height [mAU]	Area %
1	4.825	VV	0.1131	2396.88330	328.71405	49.9493
2	5.588	BB	0.1138	2401.74707	326.45248	50.0507

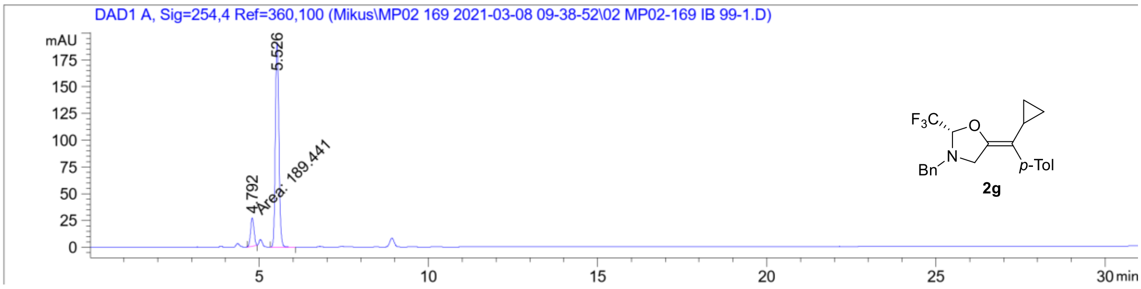

Peak #	RetTime [min]	Type	Width [min]	Area [mAU*s]	Height [mAU]	Area %
1	4.792	MM	0.1191	189.44070	26.49929	12.0151
2	5.526	BB	0.1125	1387.24268	191.48364	87.9849

Chiral HPLC Daicel Chiralpak IB N-5 column: 99:1 hexane/IPA, flow rate 1 mL/min, λ = 254 nm

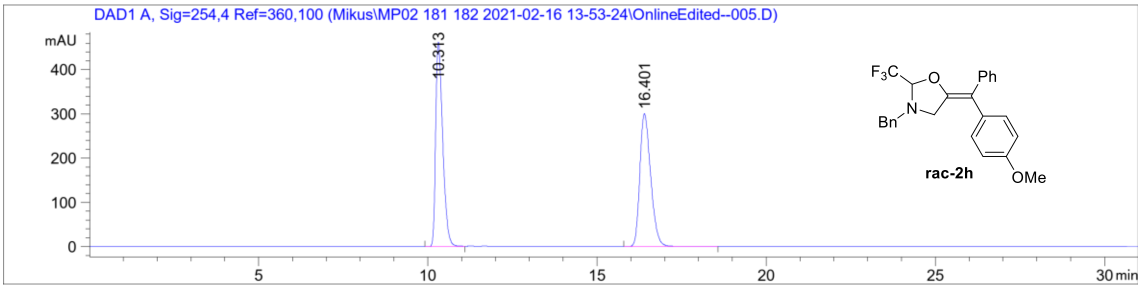

Peak #	RetTime [min]	Type	Width [min]	Area [mAU*s]	Height [mAU]	Area %
1	10.313	BB	0.2198	6638.86914	461.66302	49.8470
2	16.401	BB	0.3436	6679.63379	300.24307	50.1530

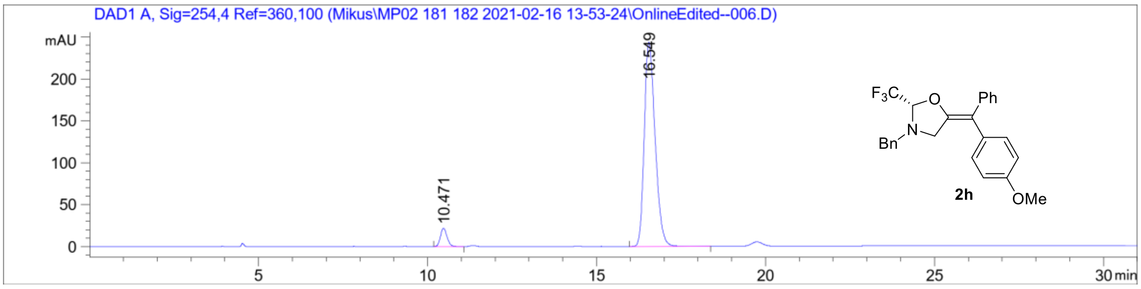

Peak #	RetTime [min]	Type	Width [min]	Area [mAU*s]	Height [mAU]	Area %
1	10.471	BB	0.2109	295.13519	21.66521	5.1588
2	16.549	BB	0.3420	5425.82861	243.43414	94.8412

Chiral HPLC Daicel Chiralpak IA column: 95:5 hexane/IPA, flow rate 1 mL/min, λ = 254 nm

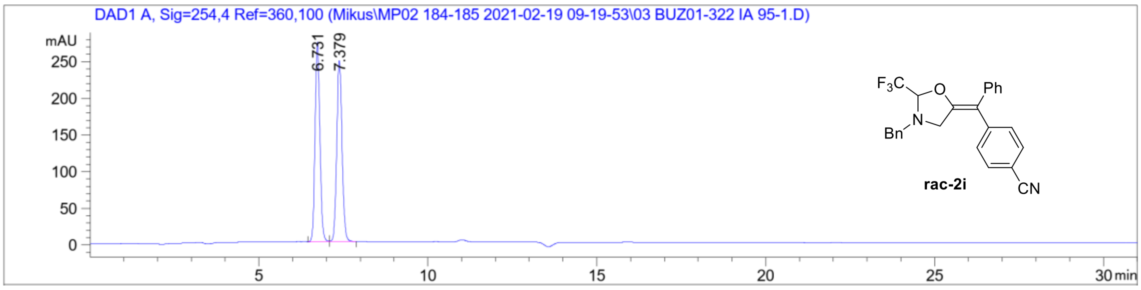

Peak #	RetTime [min]	Type	Width [min]	Area [mAU*s]	Height [mAU]	Area %
1	6.731	BV	0.1538	2696.47900	271.63037	50.0155
2	7.379	VB	0.1695	2694.80371	246.61102	49.9845

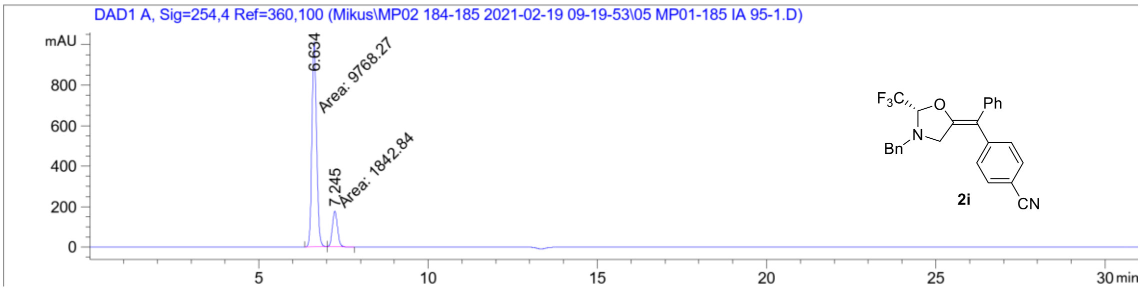

Peak #	RetTime [min]	Type	Width [min]	Area [mAU*s]	Height [mAU]	Area %
1	6.634	MM	0.1612	9768.26758	1010.17139	84.1286
2	7.245	MM	0.1740	1842.84033	176.55234	15.8714

Chiral HPLC Daicel Chiralpak IB N-5 column: 99:1 hexane/IPA, flow rate 1 mL/min, λ = 254 nm

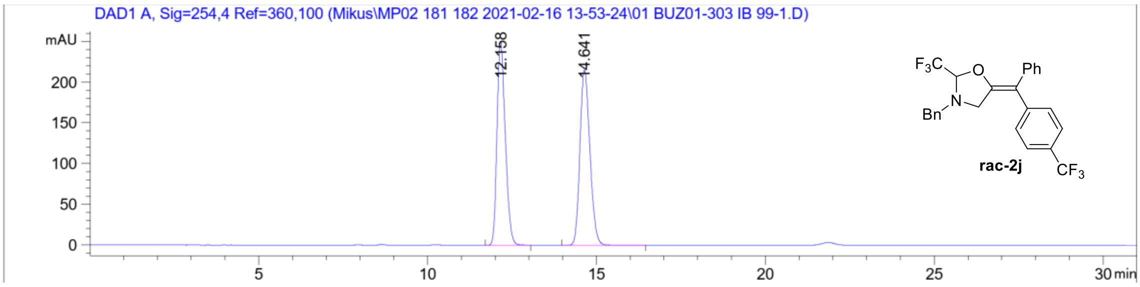

Peak #	RetTime [min]	Type	Width [min]	Area [mAU*s]	Height [mAU]	Area %
1	12.158	BB	0.2637	4286.57520	250.42015	49.1977
2	14.641	BB	0.3179	4426.37891	215.29947	50.8023

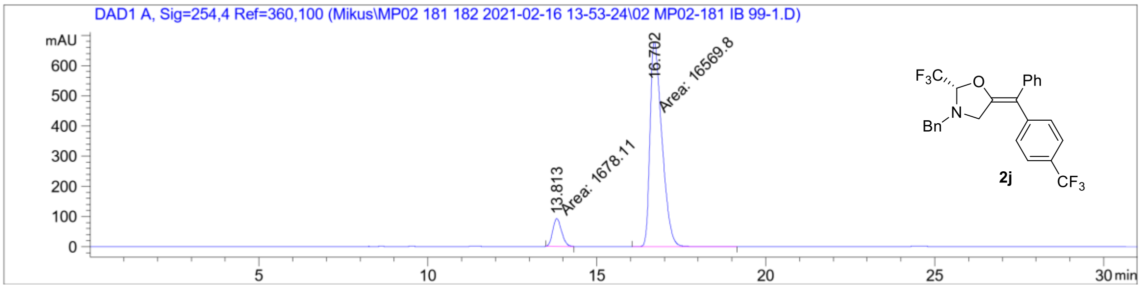

Peak #	RetTime [min]	Type	Width [min]	Area [mAU*s]	Height [mAU]	Area %
1	13.813	MM	0.3042	1678.11475	91.94987	9.1962
2	16.702	MM	0.4076	1.65698e4	677.56830	90.8038

Chiral HPLC Daicel Chiralpak IA column: 95:5 hexane/IPA, flow rate 1 mL/min, λ = 254 nm

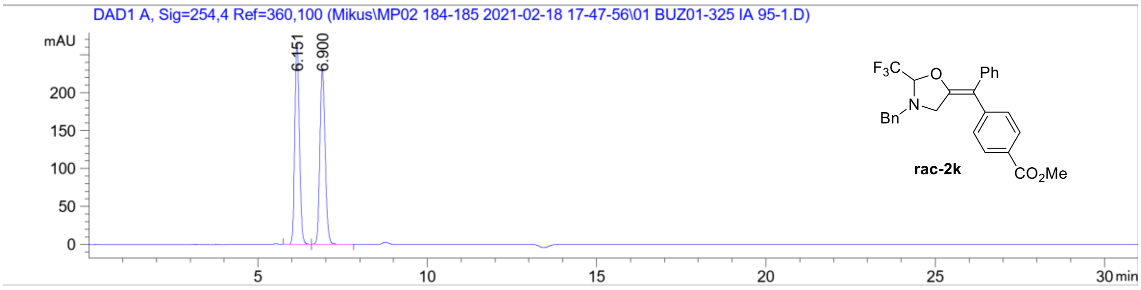

Peak #	RetTime [min]	Type	Width [min]	Area [mAU*s]	Height [mAU]	Area %
1	6.151	BB	0.1482	2508.97876	265.66409	49.9419
2	6.900	BB	0.1686	2514.81641	231.73305	50.0581

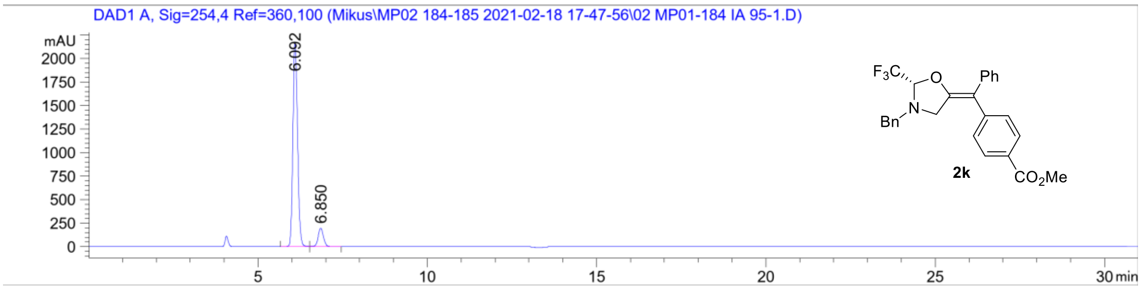

Peak #	RetTime [min]	Type	Width [min]	Area [mAU*s]	Height [mAU]	Area %
1	6.092	VV	0.1495	2.07435e4	2170.07031	90.3714
2	6.850	VB	0.1727	2210.10962	197.33310	9.6286

Chiral HPLC Daicel Chiralpak IB N-5 column: 99:1 hexane/IPA, flow rate 1 mL/min, λ = 254 nm

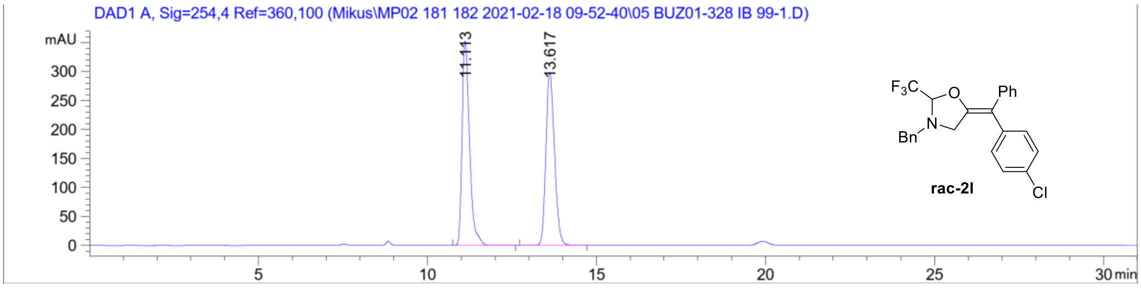

Peak #	RetTime [min]	Type	Width [min]	Area [mAU*s]	Height [mAU]	Area %
1	11.113	BB	0.2344	5391.71387	352.50629	50.5452
2	13.617	BB	0.2743	5275.40283	298.36484	49.4548

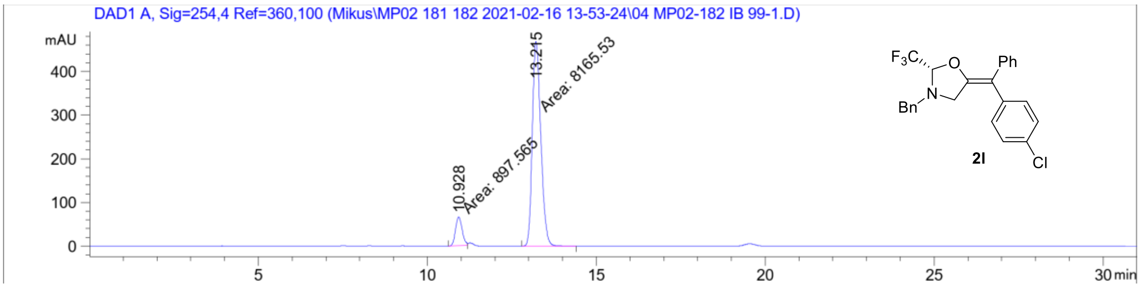

Peak #	RetTime [min]	Type	Width [min]	Area [mAU*s]	Height [mAU]	Area %
1	10.928	MM	0.2268	897.56451	65.96429	9.9035
2	13.215	MM	0.2906	8165.52979	468.32892	90.0965

Chiral HPLC Daicel Chiralpak IB N-5 column: 99:1 hexane/IPA, flow rate 1 mL/min, λ = 254 nm

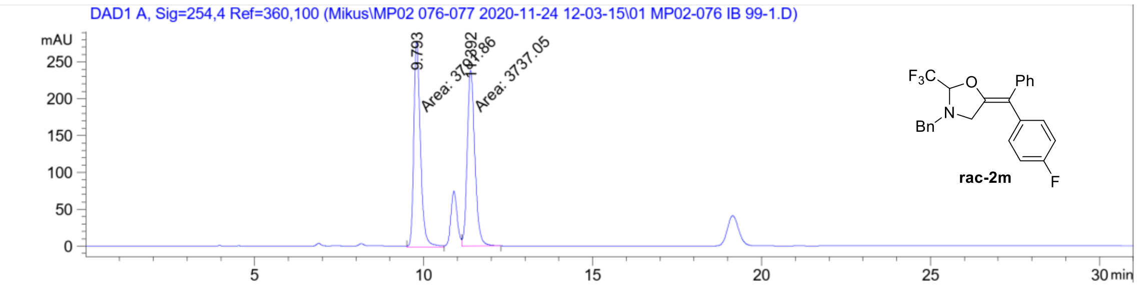

Peak #	RetTime [min]	Type	Width [min]	Area [mAU*s]	Height [mAU]	Area %
1	9.793	MM	0.2267	3791.85571	278.72601	50.3639
2	11.392	MM	0.2610	3737.05469	238.63475	49.6361

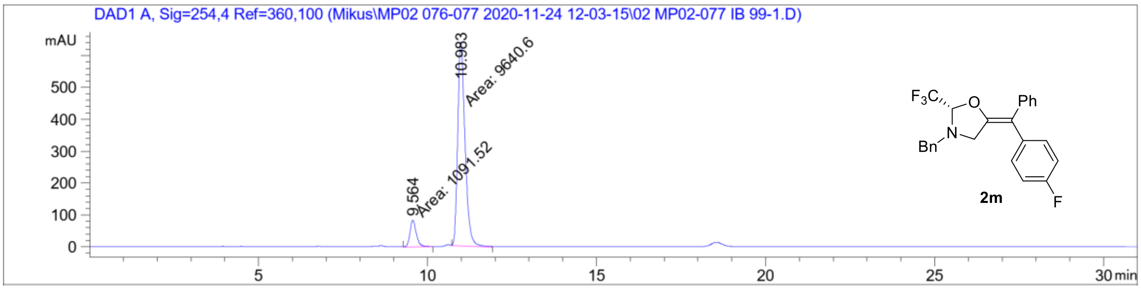

Peak #	RetTime [min]	Type	Width [min]	Area [mAU*s]	Height [mAU]	Area %
1	9.564	MM	0.2199	1091.51880	82.71938	10.1706
2	10.983	MM	0.2510	9640.59668	640.22076	89.8294

Chiral HPLC Daicel Chiralpak IB N-5 column: 99:1 hexane/IPA, flow rate 1 mL/min, λ = 254 nm

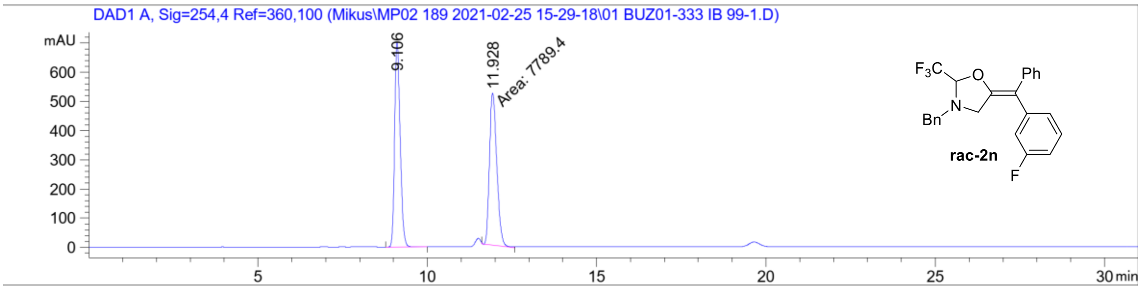

Peak #	RetTime [min]	Type	Width [min]	Area [mAU*s]	Height [mAU]	Area %
1	9.106	BB	0.1765	7972.14209	701.87134	50.5797
2	11.928	MM	0.2494	7789.40234	520.64459	49.4203

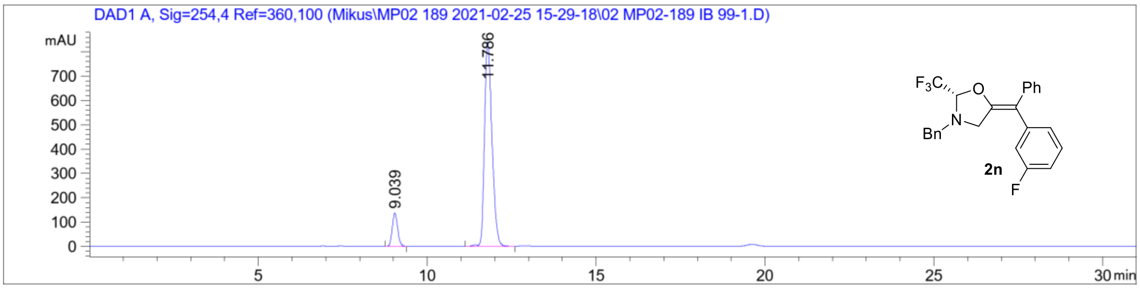

Peak #	RetTime [min]	Type	Width [min]	Area [mAU*s]	Height [mAU]	Area %
1	9.039	BB	0.1718	1507.11890	137.54398	10.4987
2	11.786	VB R	0.2337	1.28482e4	839.43896	89.5013

Chiral HPLC Daicel Chiralpak IB N-5 column: 99:1 hexane/IPA, flow rate 1 mL/min, λ = 254 nm

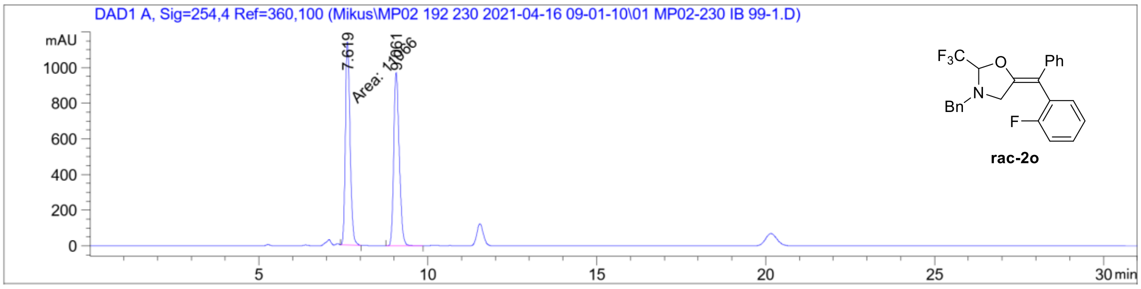

Peak #	RetTime [min]	Type	Width [min]	Area [mAU*s]	Height [mAU]	Area %
1	7.619	MM	0.1615	1.10660e4	1141.89709	50.0253
2	9.061	BB	0.1768	1.10548e4	970.95984	49.9747

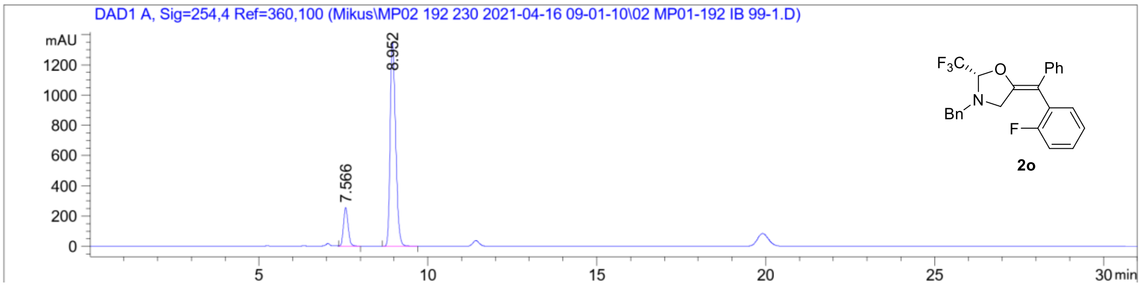

Peak #	RetTime [min]	Type	Width [min]	Area [mAU*s]	Height [mAU]	Area %
1	7.566	VB	0.1467	2445.81323	257.72256	13.7937
2	8.952	BB	0.1739	1.52855e4	1351.63513	86.2063

Chiral HPLC Daicel Chiralpak IB N-5 column: 90:10 hexane/IPA, flow rate 1 mL/min, λ = 254 nm

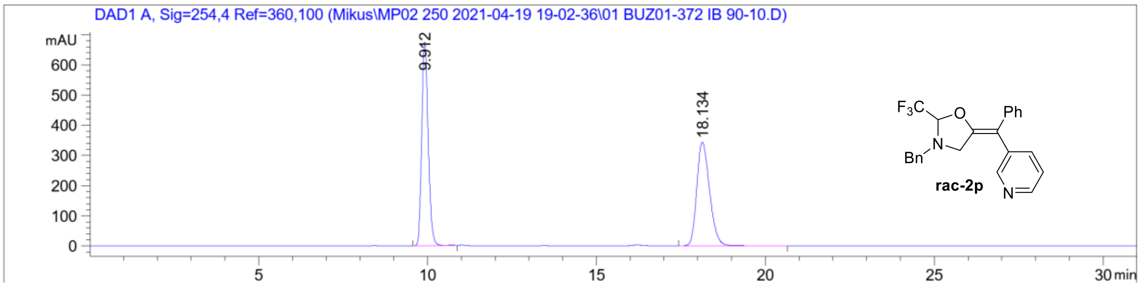

Peak #	RetTime [min]	Type	Width [min]	Area [mAU*s]	Height [mAU]	Area %
1	9.912	BV R	0.2050	8980.29297	674.70959	49.9513
2	18.134	BB	0.4058	8997.81641	343.28778	50.0487

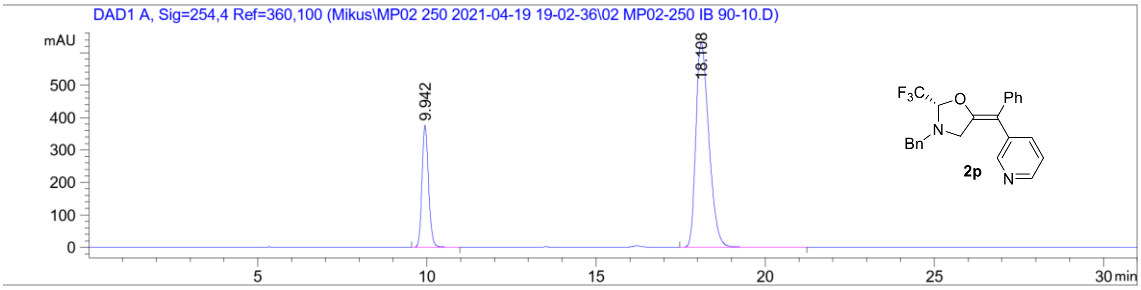

Peak #	RetTime [min]	Type	Width [min]	Area [mAU*s]	Height [mAU]	Area %
1	9.942	BB	0.2069	5062.79688	376.41376	23.0899
2	18.108	BB	0.4112	1.68636e4	632.16608	76.9101

Chiral HPLC Daicel Chiralpak IB N-5 column: 99:1 hexane/IPA, flow rate 1 mL/min, λ = 254 nm

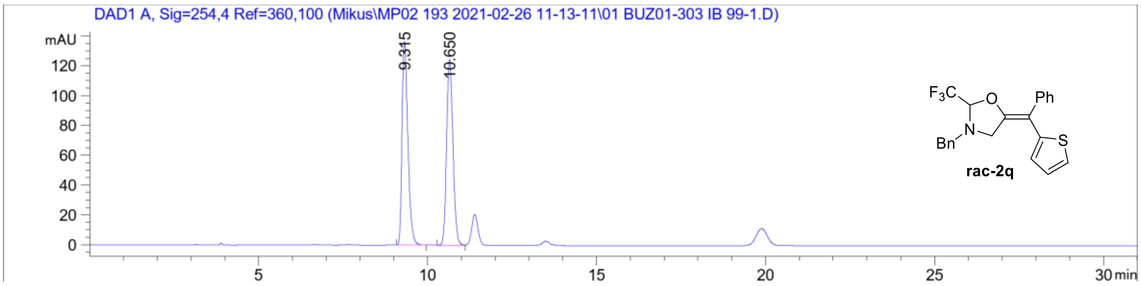

Peak #	RetTime [min]	Type	Width [min]	Area [mAU*s]	Height [mAU]	Area %
1	9.315	BB	0.1834	1630.20459	136.41965	50.0269
2	10.650	BV	0.2045	1628.44934	124.55177	49.9731

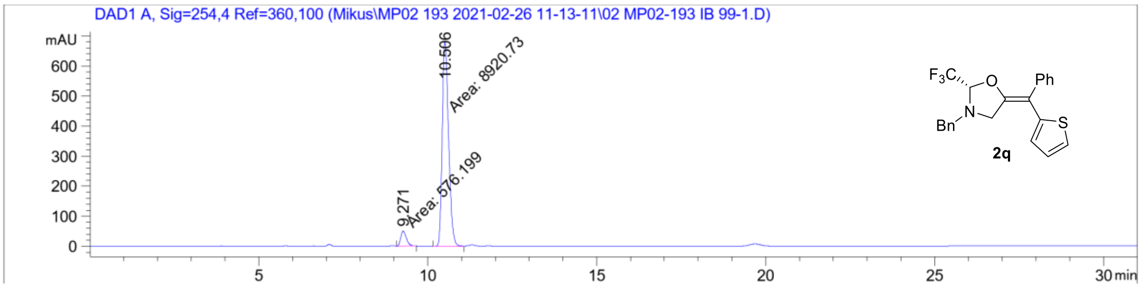

Peak #	RetTime [min]	Type	Width [min]	Area [mAU*s]	Height [mAU]	Area %
1	9.271	MM	0.1920	576.19910	50.02419	6.0672
2	10.506	MM	0.2184	8920.73242	680.80975	93.9328

H.2.Cyclopropanation products

Chiral HPLC Daicel Chiralpak IB N-5 column: 99:1 hexane/IPA, flow rate 1 mL/min, λ = 254 nm

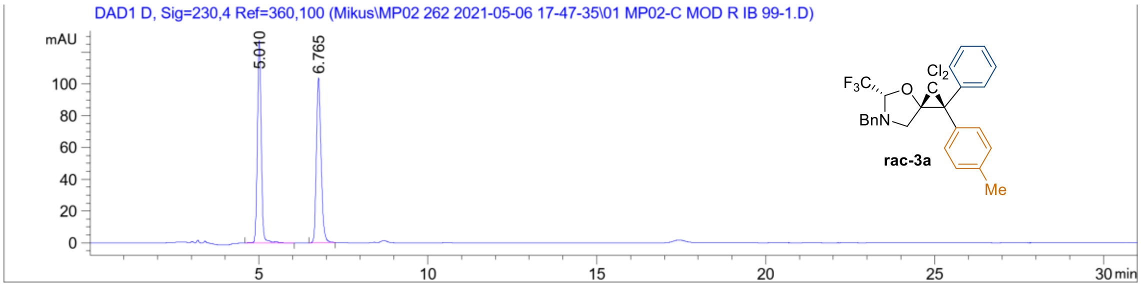

Peak #	RetTime [min]	Type	Width [min]	Area [mAU*s]	Height [mAU]	Area %
1	5.010	BV R	0.1228	1013.08618	127.31103	50.4800
2	6.765	BB	0.1458	993.81982	103.67456	49.5200

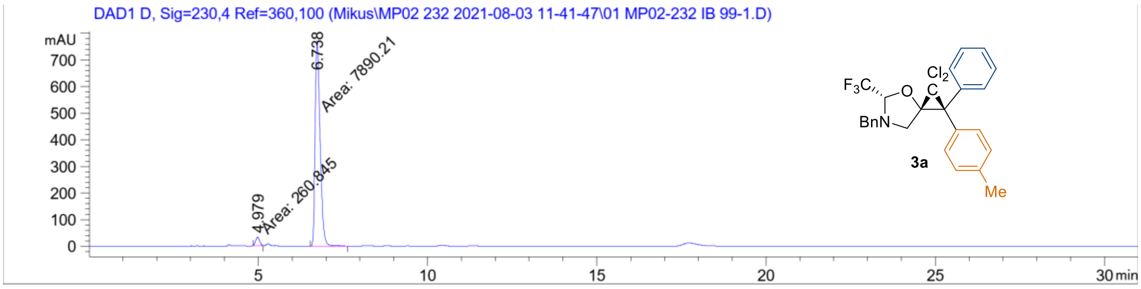

Peak #	RetTime [min]	Type	Width [min]	Area [mAU*s]	Height [mAU]	Area %
1	4.979	MM	0.1361	260.84515	31.94954	3.2001
2	6.738	MM	0.1707	7890.21240	770.22894	96.7999

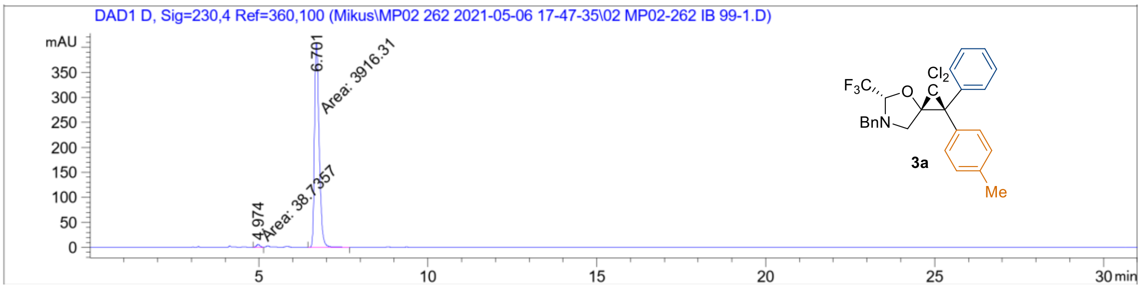

Peak #	RetTime [min]	Type	Width [min]	Area [mAU*s]	Height [mAU]	Area %
1	4.974	MM	0.1175	38.73566	5.49246	0.9794
2	6.701	MM	0.1600	3916.30957	407.95474	99.0206

Chiral HPLC Daicel Chiralpak IB N-5 column: 100:0 hexane/IPA, flow rate 1 mL/min, λ = 230 nm

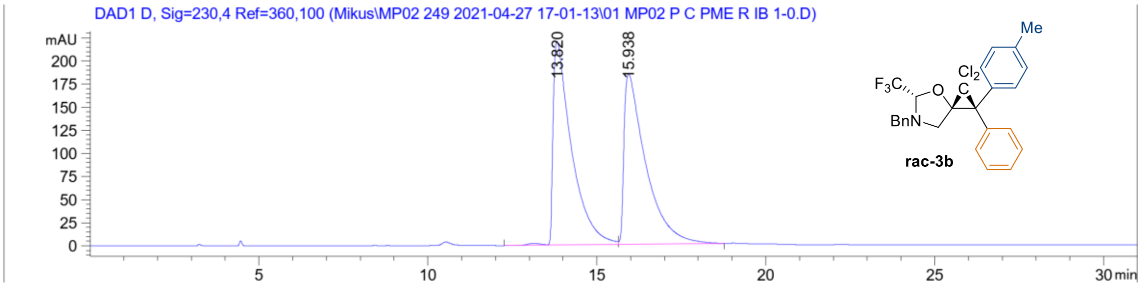

Peak #	RetTime [min]	Type	Width [min]	Area [mAU*s]	Height [mAU]	Area %
1	13.820	VV R	0.5367	8389.55762	220.50897	50.4563
2	15.938	VB	0.6367	8237.80664	184.57341	49.5437

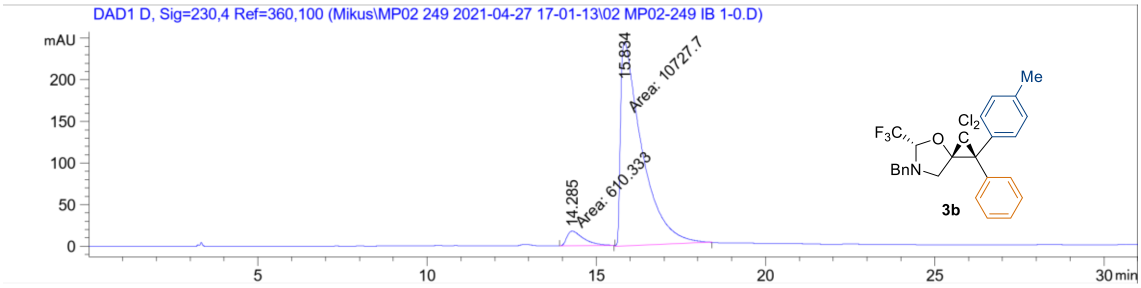

Peak #	RetTime [min]	Type	Width [min]	Area [mAU*s]	Height [mAU]	Area %
1	14.285	MM	0.5792	610.33301	17.56160	5.3831
2	15.834	MM	0.7296	1.07277e4	245.06920	94.6169

Chiral HPLC Daicel Chiralpak IB N-5 column: 99:1 hexane/IPA, flow rate 1 mL/min, λ = 230 nm

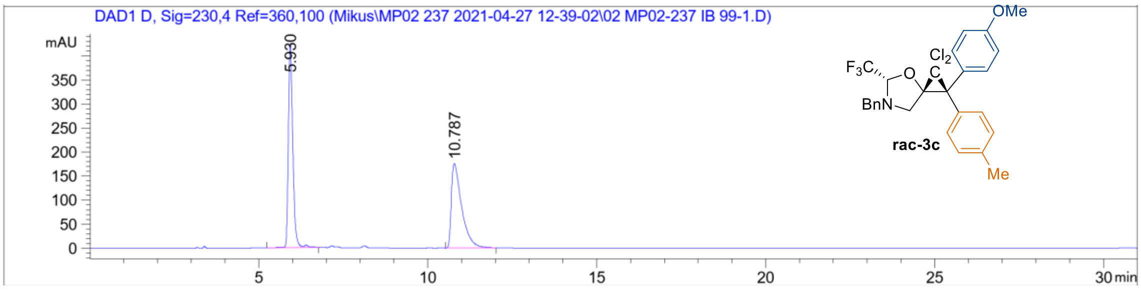

Peak #	RetTime [min]	Type	Width [min]	Area [mAU*s]	Height [mAU]	Area %
1	5.930	VV R	0.1364	3874.24658	424.99191	50.9385
2	10.787	BB	0.3114	3731.48218	175.96339	49.0615

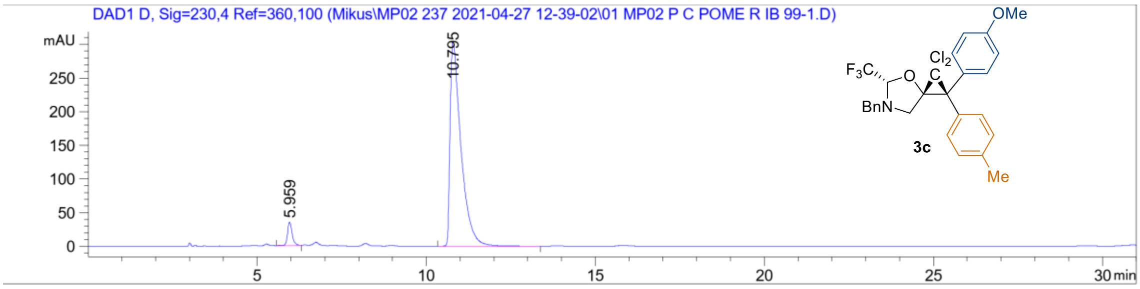

Peak #	RetTime [min]	Type	Width [min]	Area [mAU*s]	Height [mAU]	Area %
1	5.959	BB	0.1441	333.56717	34.70132	4.6709
2	10.795	BB	0.3273	6807.80615	303.96573	95.3291

Chiral HPLC Daicel Chiralpak IB N-5 column: 99:1 hexane/IPA, flow rate 1 mL/min, λ = 230 nm

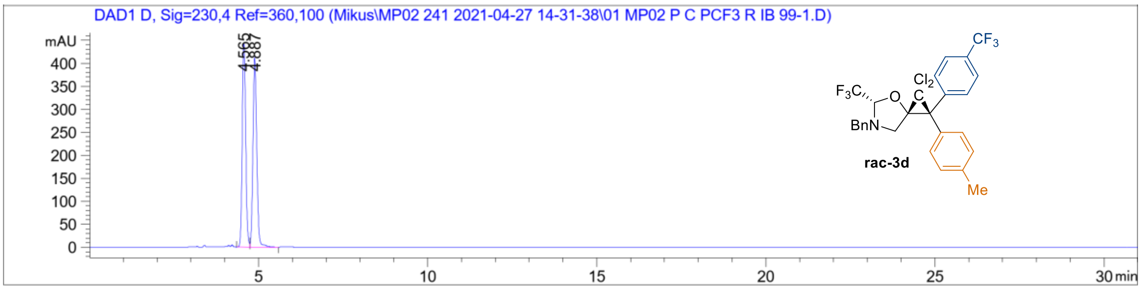

Peak #	RetTime [min]	Type	Width [min]	Area [mAU*s]	Height [mAU]	Area %
1	4.565	BV	0.1105	3138.40771	444.08136	49.3954
2	4.887	VB	0.1212	3215.23242	411.40179	50.6046

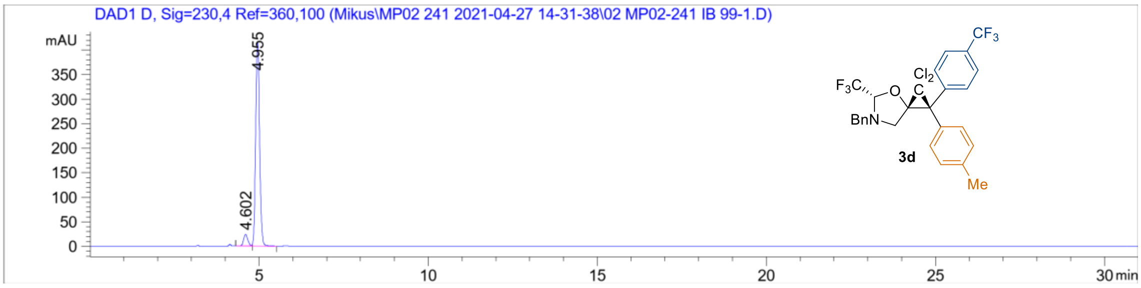

Peak #	RetTime [min]	Type	Width [min]	Area [mAU*s]	Height [mAU]	Area %
1	4.602	BV E	0.1367	208.85738	23.71952	5.8984
2	4.955	VB R	0.1233	3332.08301	416.66678	94.1016

Chiral HPLC Daicel Chiralpak IB N-5 column: 100:0 hexane/IPA, flow rate 1 mL/min, λ = 230 nm

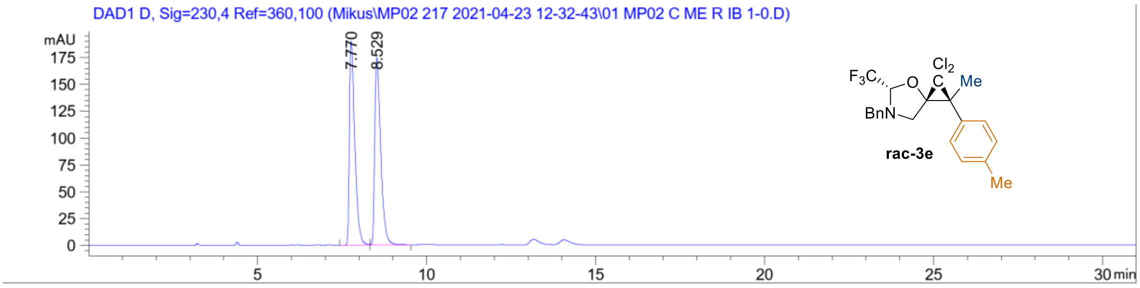

Peak #	RetTime [min]	Type	Width [min]	Area [mAU*s]	Height [mAU]	Area %
1	7.770	BV	0.1678	2113.37402	189.99213	49.6925
2	8.529	VB	0.1833	2139.53174	174.24231	50.3075

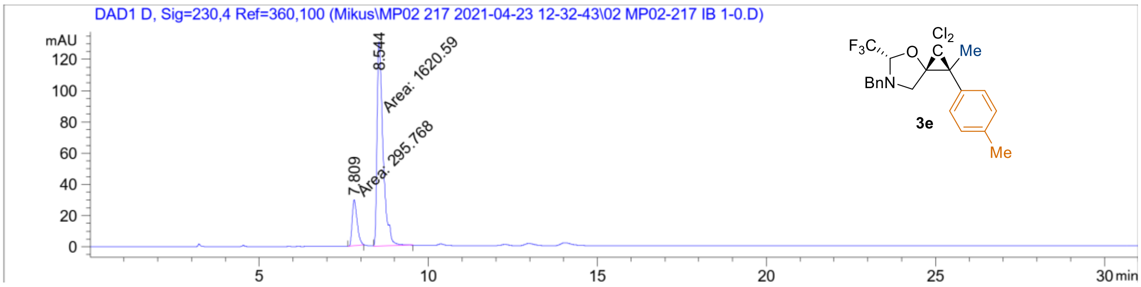

Peak #	RetTime [min]	Type	Width [min]	Area [mAU*s]	Height [mAU]	Area %
1	7.809	MM	0.1671	295.76758	29.50390	15.4338
2	8.544	MM	0.2062	1620.59058	131.01260	84.5662

Chiral HPLC Daicel Chiralpak IB N-5 column: 100:0 hexane/IPA, flow rate 1 mL/min, λ = 230 nm

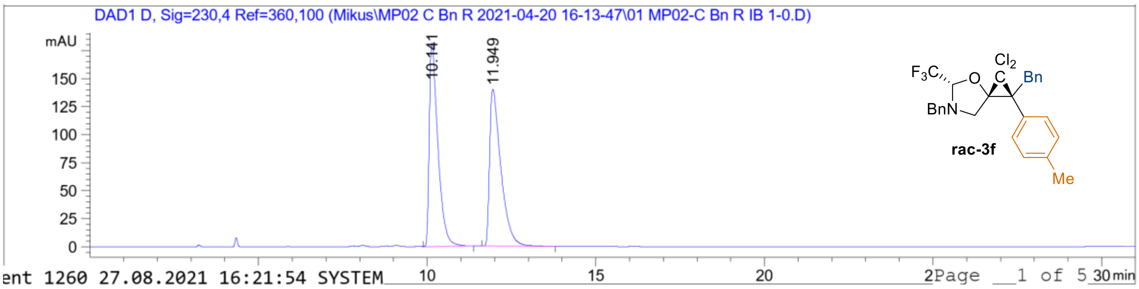

Peak #	RetTime [min]	Type	Width [min]	Area [mAU*s]	Height [mAU]	Area %
1	10.141	BB	0.2655	3228.84131	181.50577	49.8535
2	11.949	BB	0.3452	3247.82056	139.74002	50.1465

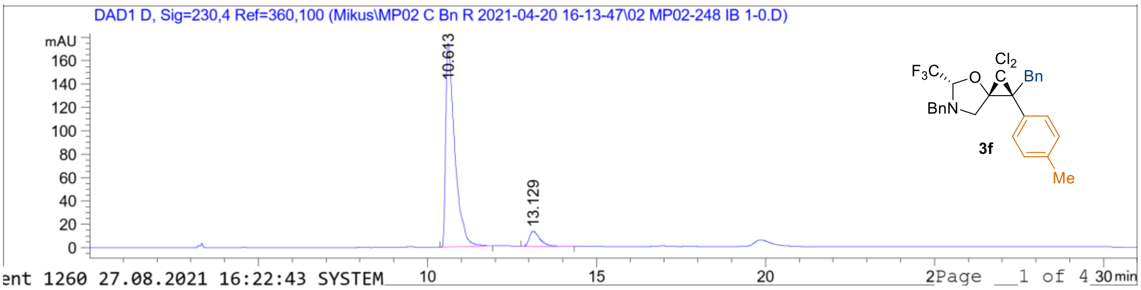

Peak #	RetTime [min]	Type	Width [min]	Area [mAU*s]	Height [mAU]	Area %
1	10.613	BB	0.2735	3251.26709	174.38910	92.0224
2	13.129	BB	0.3284	281.85733	12.92515	7.9776

Chiral HPLC Daicel Chiralpak IB N-5 column: 100:0 hexane/IPA, flow rate 1 mL/min, λ = 210 nm

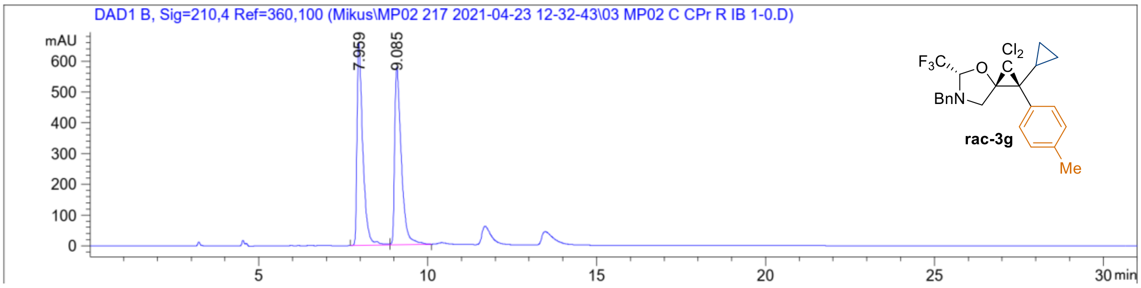

Peak #	RetTime [min]	Type	Width [min]	Area [mAU*s]	Height [mAU]	Area %
1	7.959	BV R	0.1740	7942.53076	660.52557	50.1005
2	9.085	VB	0.2040	7910.67432	576.91895	49.8995

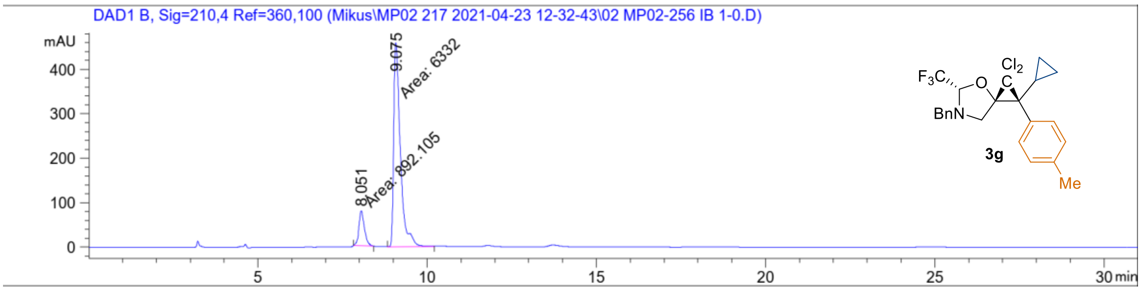

Peak #	RetTime [min]	Type	Width [min]	Area [mAU*s]	Height [mAU]	Area %
1	8.051	MM	0.1887	892.10522	78.80287	12.3490
2	9.075	MM	0.2297	6331.99512	459.51166	87.6510

Chiral HPLC Daicel Chiralpak IB N-5 column: 99:1 hexane/IPA, flow rate 1 mL/min, λ = 230 nm

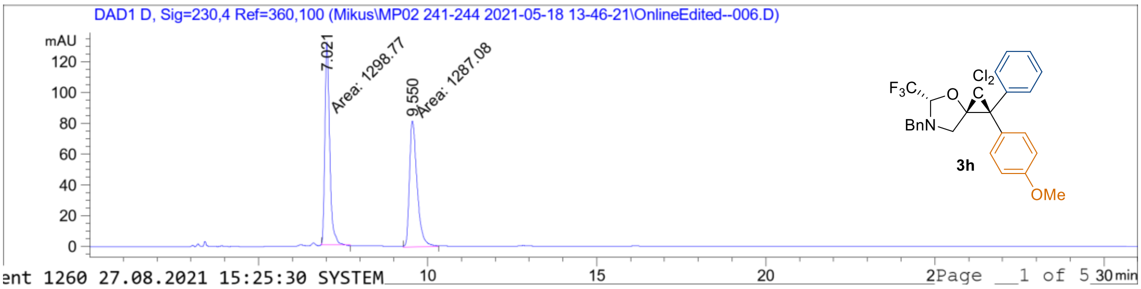

Peak #	RetTime [min]	Type	Width [min]	Area [mAU*s]	Height [mAU]	Area %
1	7.021	MM	0.1646	1298.77246	131.51042	50.2261
2	9.550	MM	0.2621	1287.08179	81.85575	49.7739

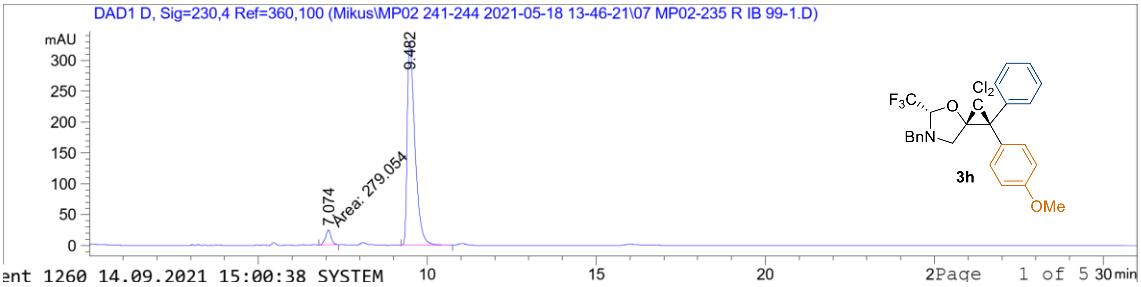

Peak #	RetTime [min]	Type	Width [min]	Area [mAU*s]	Height [mAU]	Area %
1	7.074	MM	0.1926	279.05399	24.14656	5.0687
2	9.482	BB	0.2364	5226.35645	330.71964	94.9313

Chiral HPLC Daicel Chiralpak IA column: 95:5 hexane/IPA, flow rate 1 mL/min, λ = 254 nm

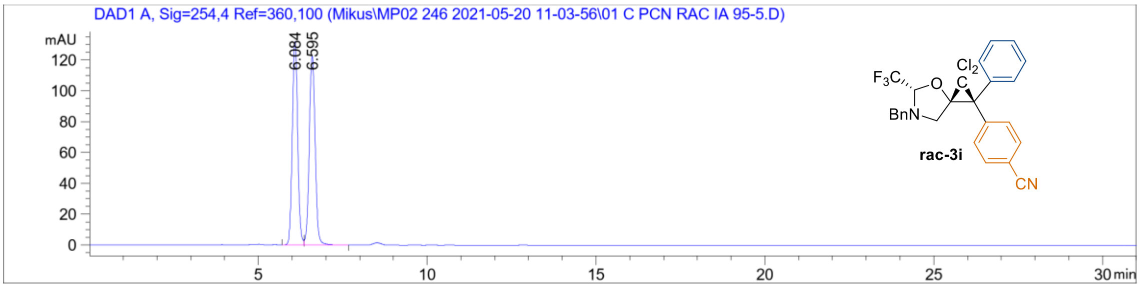

Peak #	RetTime [min]	Type	Width [min]	Area [mAU*s]	Height [mAU]	Area %
1	6.084	BV	0.1759	1470.12927	132.05241	49.5758
2	6.595	VB	0.1924	1495.28674	122.47664	50.4242

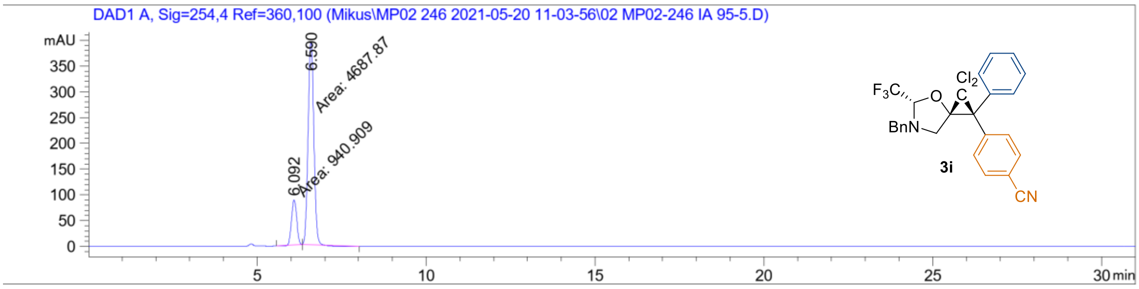

Peak #	RetTime [min]	Type	Width [min]	Area [mAU*s]	Height [mAU]	Area %
1	6.092	MM	0.1797	940.90942	87.27347	16.7160
2	6.590	MM	0.1986	4687.87354	393.37320	83.2840

Chiral HPLC Daicel Chiralpak IB N-5 column: 99:1 hexane/IPA, flow rate 1 mL/min, λ = 230 nm

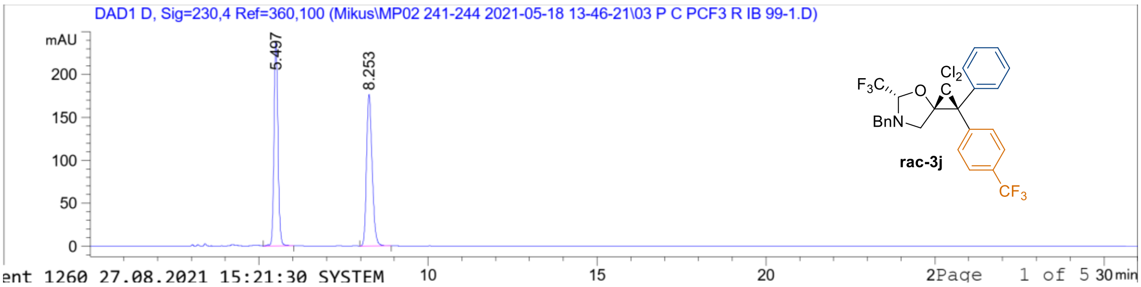

ent 1260 27.08.2021 15:21:30 SYSTEM Page 1 of 5

Peak #	RetTime [min]	Type	Width [min]	Area [mAU*s]	Height [mAU]	Area %
1	5.497	VB	0.1279	1956.67297	238.16035	49.1121
2	8.253	BB	0.1761	2027.41931	176.41267	50.8879

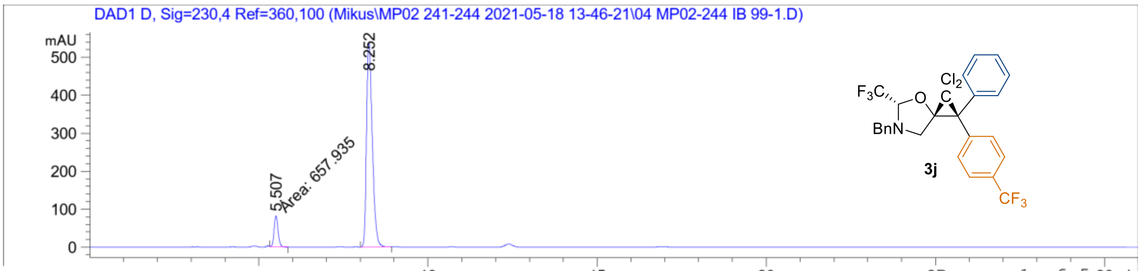

ent 1260 27.08.2021 15:22:38 SYSTEM Page 1 of 5

Peak #	RetTime [min]	Type	Width [min]	Area [mAU*s]	Height [mAU]	Area %
1	5.507	MM	0.1358	657.93530	80.75186	9.3361
2	8.252	BB	0.1822	6389.26563	539.27344	90.6639

Chiral HPLC Daicel Chiralpak IB N-5 column: 99:1 hexane/IPA, flow rate 1 mL/min, λ = 254 nm

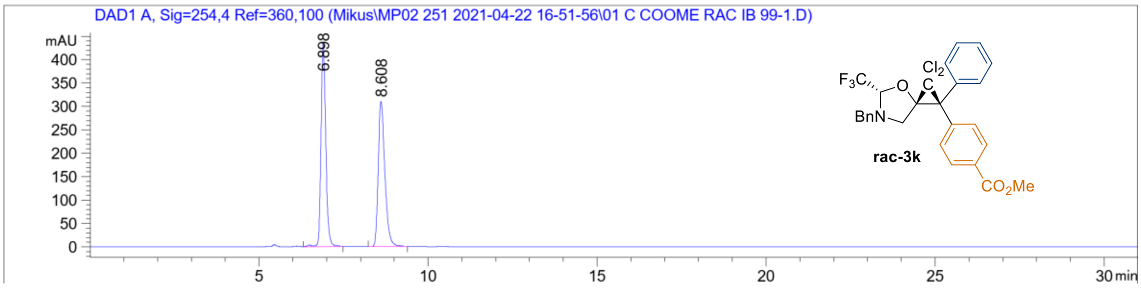

Peak #	RetTime [min]	Type	Width [min]	Area [mAU*s]	Height [mAU]	Area %
1	6.898	VB R	0.1497	4278.39502	435.48608	50.2956
2	8.608	BB	0.2093	4228.11279	309.68945	49.7044

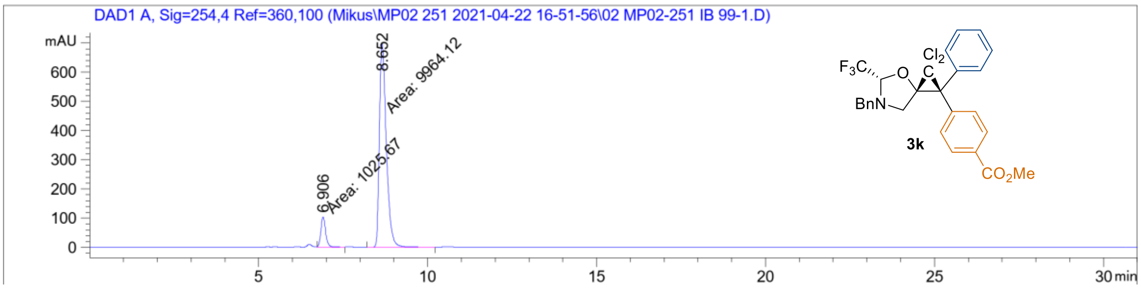

Peak #	RetTime [min]	Type	Width [min]	Area [mAU*s]	Height [mAU]	Area %
1	6.906	MM	0.1656	1025.66772	103.23822	9.3329
2	8.652	MM	0.2370	9964.12012	700.65338	90.6671

Chiral HPLC Daicel Chiralpak IB N-5 column: 99:1 hexane/IPA, flow rate 1 mL/min, λ = 230 nm

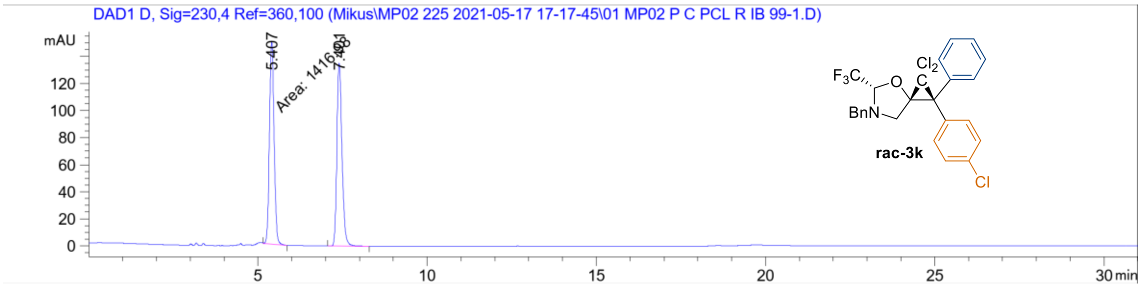

Peak #	RetTime [min]	Type	Width [min]	Area [mAU*s]	Height [mAU]	Area %
1	5.407	MM	0.1579	1416.97791	149.52751	49.8623
2	7.401	BB	0.1610	1424.80286	135.17316	50.1377

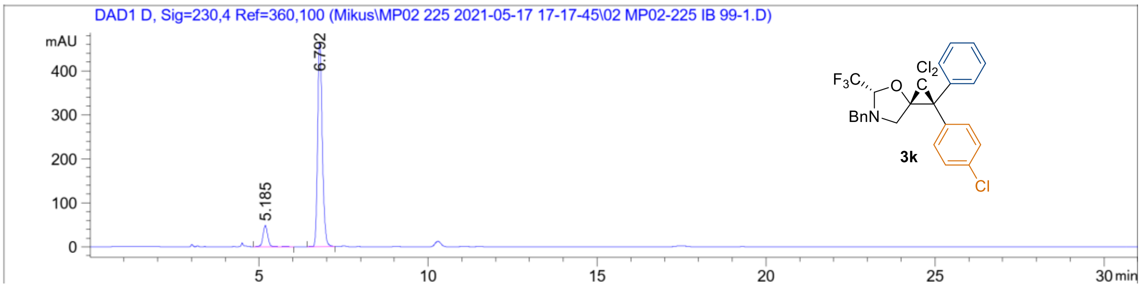

Peak #	RetTime [min]	Type	Width [min]	Area [mAU*s]	Height [mAU]	Area %
1	5.185	BV R	0.1504	485.58105	48.20890	9.8027
2	6.792	BB	0.1485	4467.97266	463.25073	90.1973

Chiral HPLC Daicel Chiralpak IB N-5 column: 99:1 hexane/IPA, flow rate 1 mL/min, λ = 230 nm

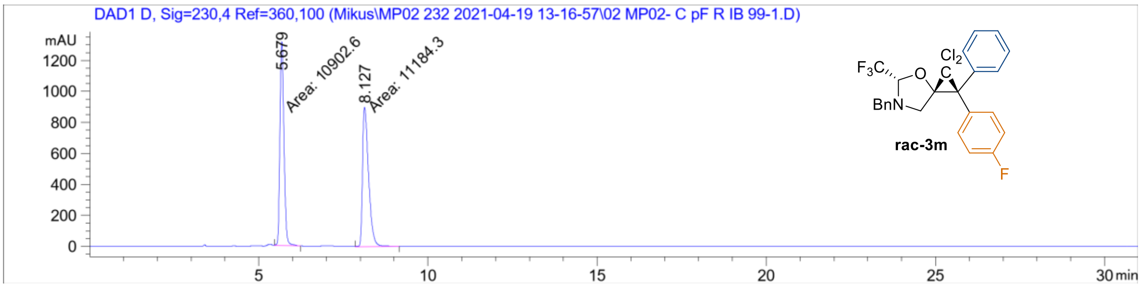

Peak #	RetTime [min]	Type	Width [min]	Area [mAU*s]	Height [mAU]	Area %
1	5.679	MM	0.1380	1.09026e4	1316.84045	49.3622
2	8.127	MM	0.2074	1.11843e4	898.69055	50.6378

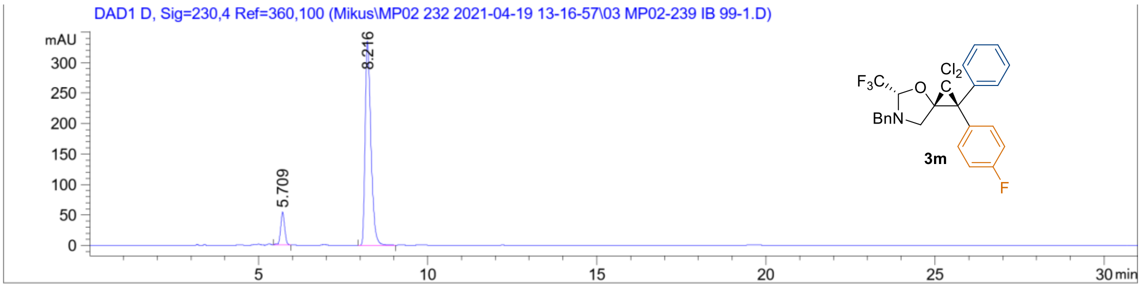

Peak #	RetTime [min]	Type	Width [min]	Area [mAU*s]	Height [mAU]	Area %
1	5.709	VB R	0.1267	444.89334	54.02472	10.0638
2	8.216	BB	0.1804	3975.84644	335.08667	89.9362

Chiral HPLC Daicel Chiralpak IB N-5 column: 99:1 hexane/IPA, flow rate 1 mL/min, λ = 230 nm

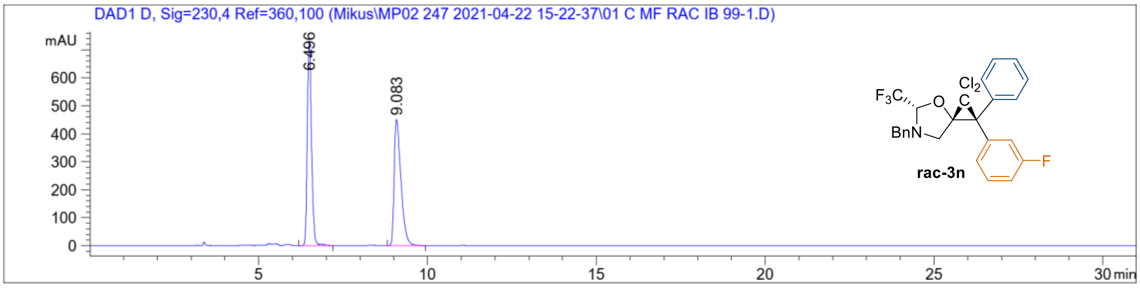

Peak #	RetTime [min]	Type	Width [min]	Area [mAU*s]	Height [mAU]	Area %
1	6.496	BV R	0.1350	6352.05176	728.54663	50.0760
2	9.083	BB	0.2138	6332.76953	451.17392	49.9240

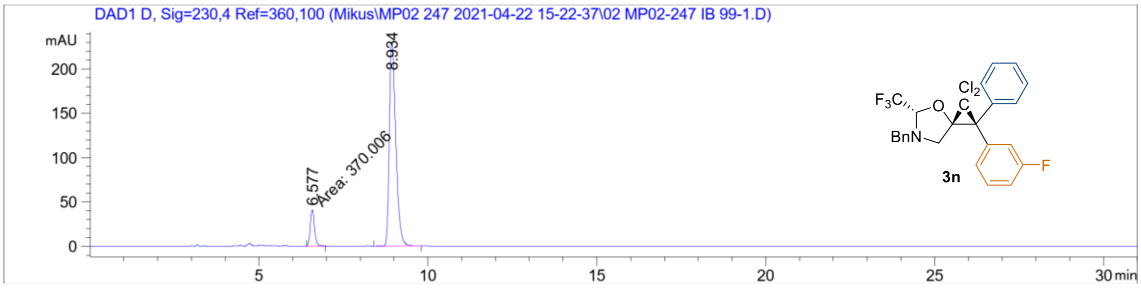

Peak #	RetTime [min]	Type	Width [min]	Area [mAU*s]	Height [mAU]	Area %
1	6.577	MM	0.1497	370.00552	41.18150	10.6923
2	8.934	BB	0.2044	3090.48438	230.48633	89.3077

H.3.Epoxidation products

Chiral HPLC Daicel Chiralpak IB N-5 column: 95:5 hexane/IPA, flow rate 1 mL/min, λ = 210 nm

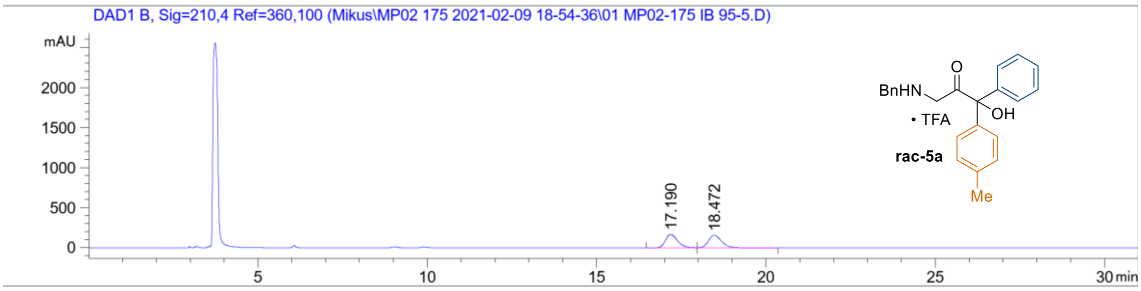

Peak #	RetTime [min]	Type	Width [min]	Area [mAU*s]	Height [mAU]	Area %
1	17.190	BV	0.3871	4216.50391	167.83023	49.4247
2	18.472	VB	0.4213	4314.66650	157.66602	50.5753

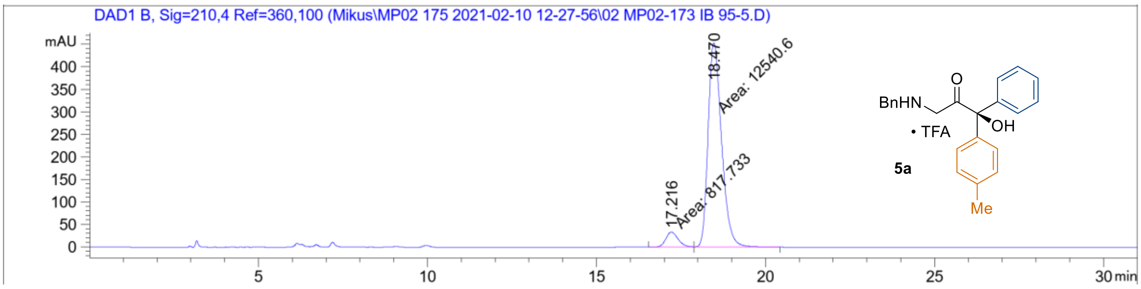

Peak #	RetTime [min]	Type	Width [min]	Area [mAU*s]	Height [mAU]	Area %
1	17.216	MM	0.4143	817.73260	32.89482	6.1215
2	18.470	MM	0.4613	1.25406e4	453.04700	93.8785

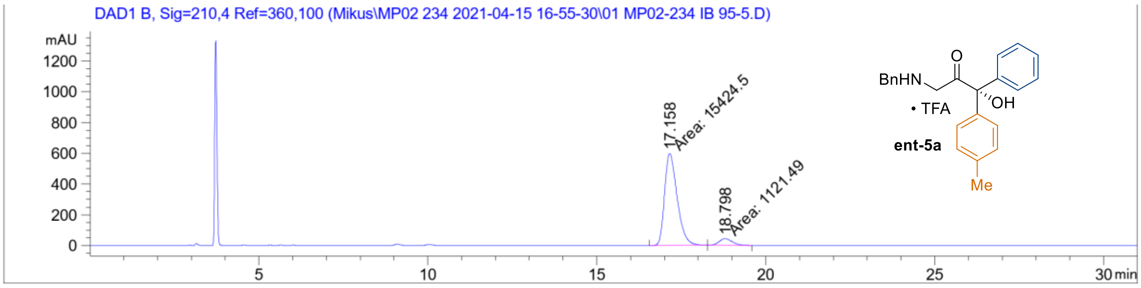

Peak #	RetTime [min]	Type	Width [min]	Area [mAU*s]	Height [mAU]	Area %
1	17.158	MM	0.4308	1.54245e4	596.75092	93.2220
2	18.798	MM	0.4429	1121.49109	42.20643	6.7780

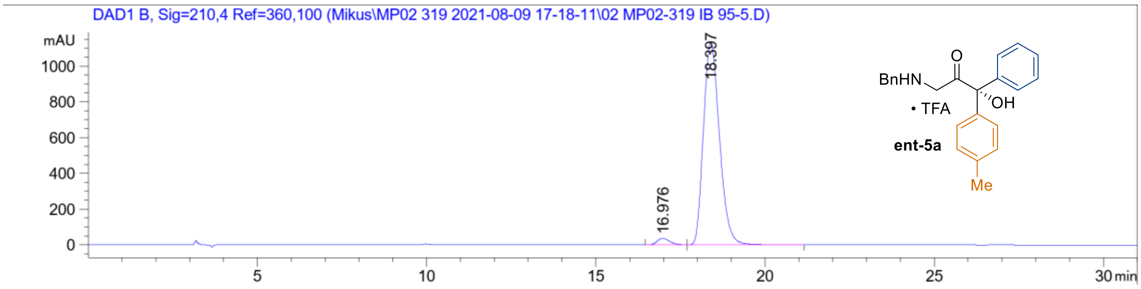

Peak #	RetTime [min]	Type	Width [min]	Area [mAU*s]	Height [mAU]	Area %
1	16.976	BB	0.3961	935.84021	36.62519	2.4525
2	18.397	BB	0.5193	3.72225e4	1132.97546	97.5475

Chiral HPLC Daicel Chiralpak IB N-5 column: 95:5 hexane/IPA, flow rate 1 mL/min, λ = 230 nm

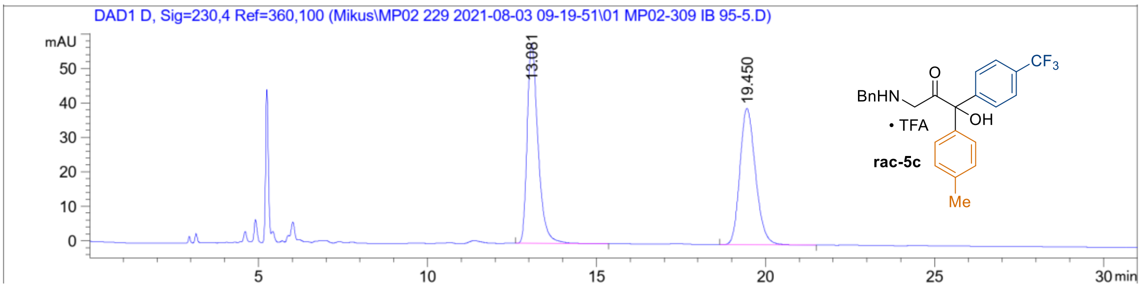

Peak #	RetTime [min]	Type	Width [min]	Area [mAU*s]	Height [mAU]	Area %
1	13.081	BB	0.3404	1296.64673	58.10036	50.3940
2	19.450	BB	0.4982	1276.37219	39.58579	49.6060

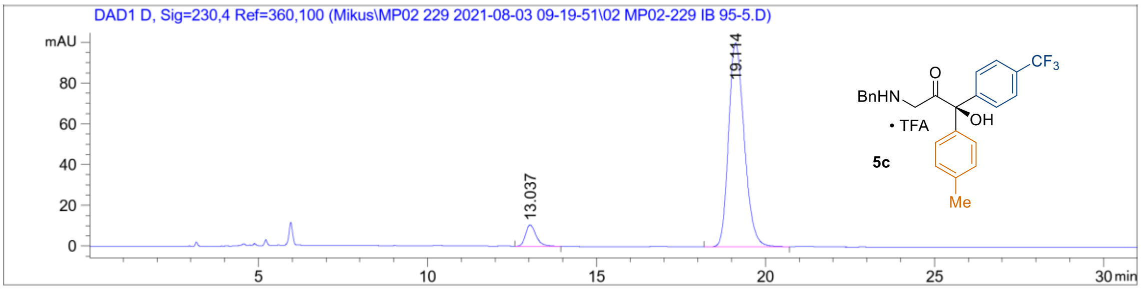

Peak #	RetTime [min]	Type	Width [min]	Area [mAU*s]	Height [mAU]	Area %
1	13.037	BB	0.3331	227.51915	10.48870	6.6099
2	19.114	BB	0.4967	3214.60303	100.09187	93.3901

Chiral HPLC Daicel Chiralpak IB N-5 column: 95:5 hexane/IPA, flow rate 1 mL/min, λ = 210 nm

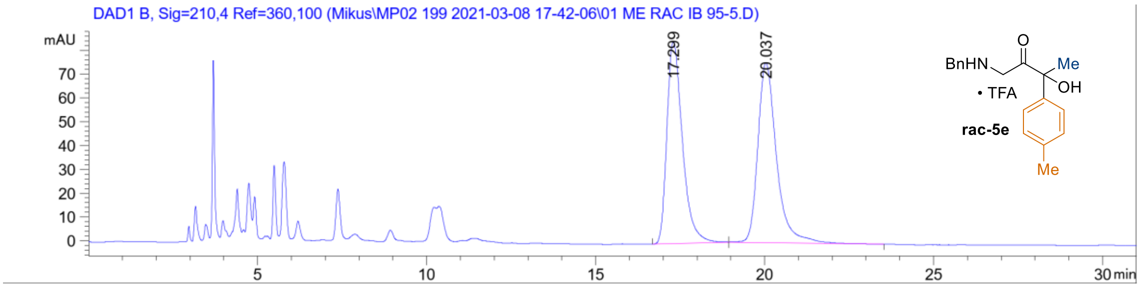

Peak #	RetTime [min]	Type	Width [min]	Area [mAU*s]	Height [mAU]	Area %
1	17.299	BB	0.4899	2706.69775	84.90887	48.3377
2	20.037	BB	0.5810	2892.86548	75.50260	51.6623

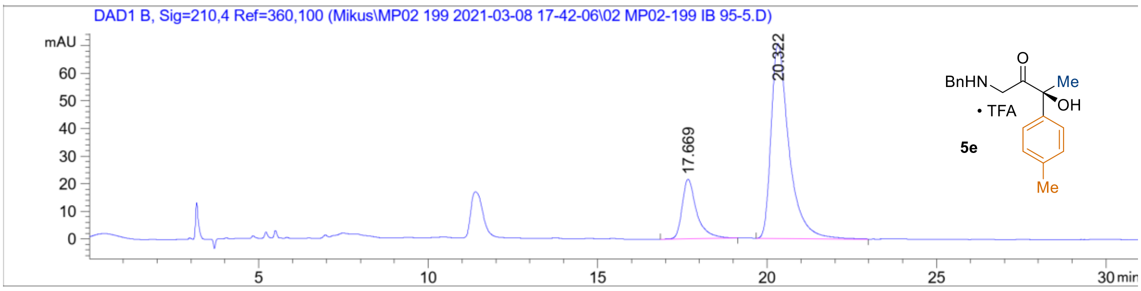

Peak #	RetTime [min]	Type	Width [min]	Area [mAU*s]	Height [mAU]	Area %
1	17.669	BB	0.4426	641.66260	21.59809	19.8812
2	20.322	BB	0.5458	2585.82300	70.51367	80.1188

Chiral HPLC Daicel Chiralpak IB N-5 column: 95:5 hexane/IPA, flow rate 1 mL/min, λ = 230 nm

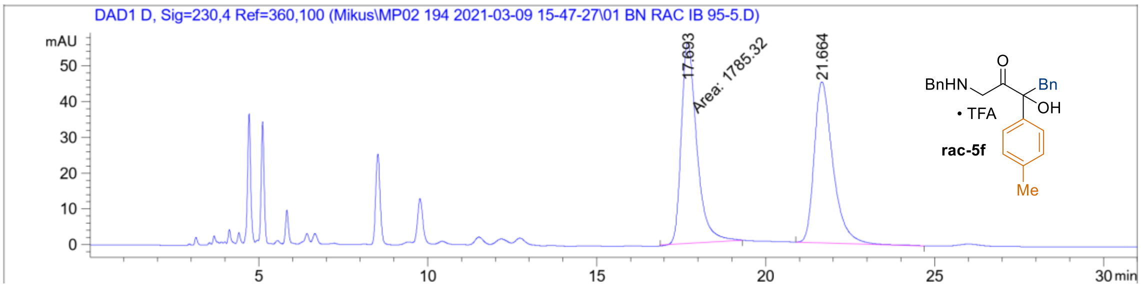

Peak #	RetTime [min]	Type	Width [min]	Area [mAU*s]	Height [mAU]	Area %
1	17.693	MM	0.5300	1785.31824	56.13843	50.7749
2	21.664	BB	0.5823	1730.82617	45.04387	49.2251

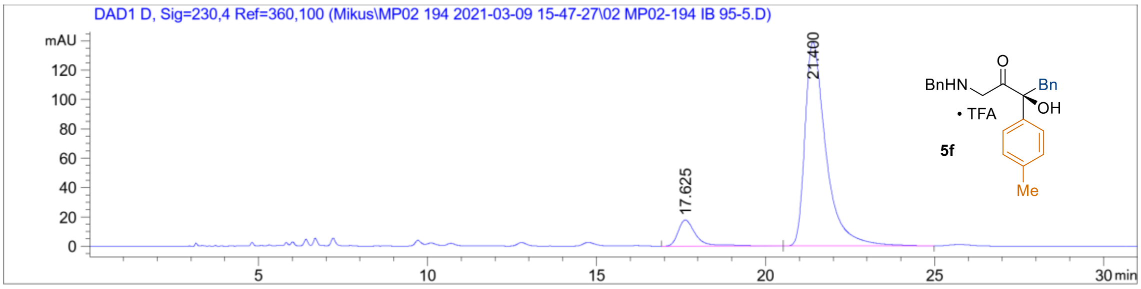

Peak #	RetTime [min]	Type	Width [min]	Area [mAU*s]	Height [mAU]	Area %
1	17.625	MM	0.5546	1680.95789	50.51163	8.8103
2	21.400	BB	0.6490	1.73984e4	400.38589	91.1897

Chiral HPLC Daicel Chiralpak IB N-5 column: 95:5 hexane/IPA, flow rate 1 mL/min, λ = 230 nm

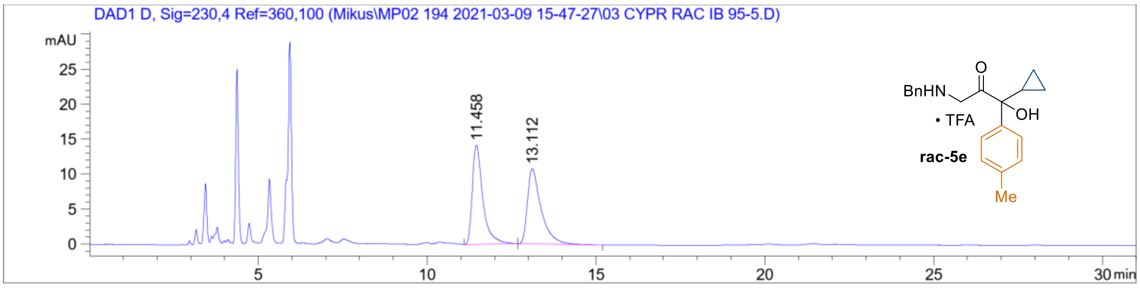

Peak #	RetTime [min]	Type	Width [min]	Area [mAU*s]	Height [mAU]	Area %
1	11.458	BB	0.3254	308.47089	14.19973	50.2735
2	13.112	BB	0.4116	305.11465	10.80113	49.7265

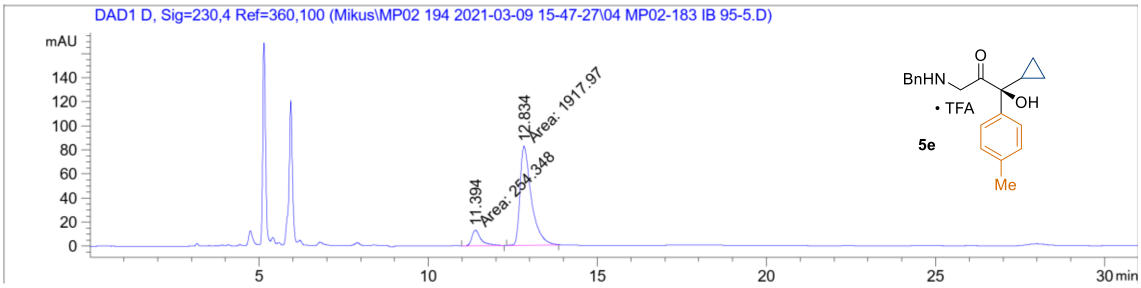

Peak #	RetTime [min]	Type	Width [min]	Area [mAU*s]	Height [mAU]	Area %
1	11.394	MM	0.3206	254.34799	13.22058	11.7086
2	12.834	MM	0.3879	1917.97009	82.41735	88.2914

Chiral HPLC Daicel Chiralpak IB N-5 column: 80:20 hexane/IPA, flow rate 1 mL/min, λ = 210 nm

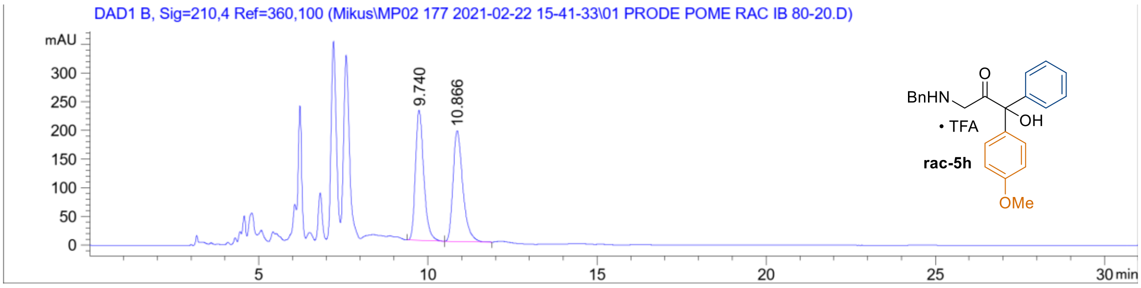

Peak #	RetTime [min]	Type	Width [min]	Area [mAU*s]	Height [mAU]	Area %
1	9.740	BB	0.2639	3891.19141	227.10849	49.9971
2	10.866	BB	0.3069	3891.64014	193.27988	50.0029

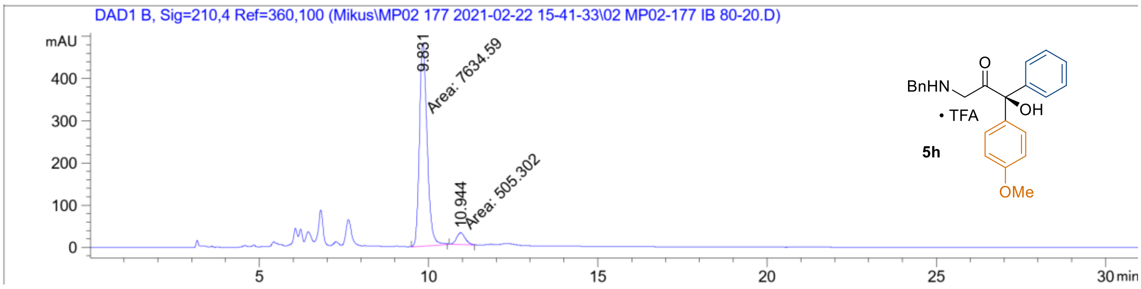

Peak #	RetTime [min]	Type	Width [min]	Area [mAU*s]	Height [mAU]	Area %
1	9.831	MM	0.2638	7634.59375	482.35980	93.7923
2	10.944	MM	0.2997	505.30206	28.09845	6.2077

Chiral HPLC Daicel Chiralpak IB N-5 column: 95:5 hexane/IPA, flow rate 1 mL/min, λ = 210 nm

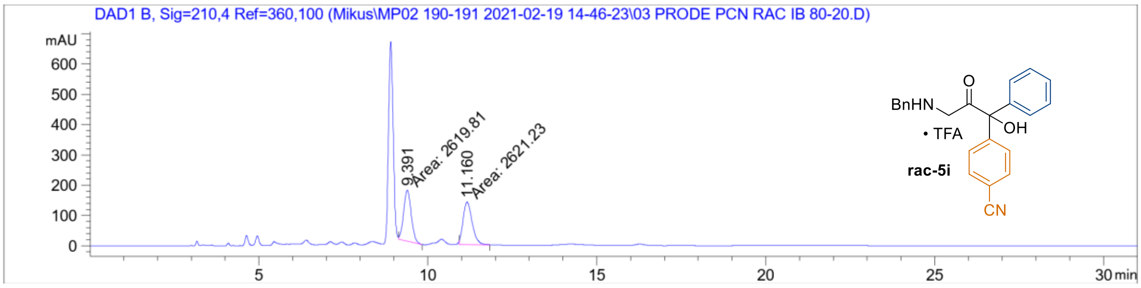

Peak #	RetTime [min]	Type	Width [min]	Area [mAU*s]	Height [mAU]	Area %
1	9.391	MM	0.2584	2619.80518	168.94466	49.9864
2	11.160	MM	0.3117	2621.23267	140.17728	50.0136

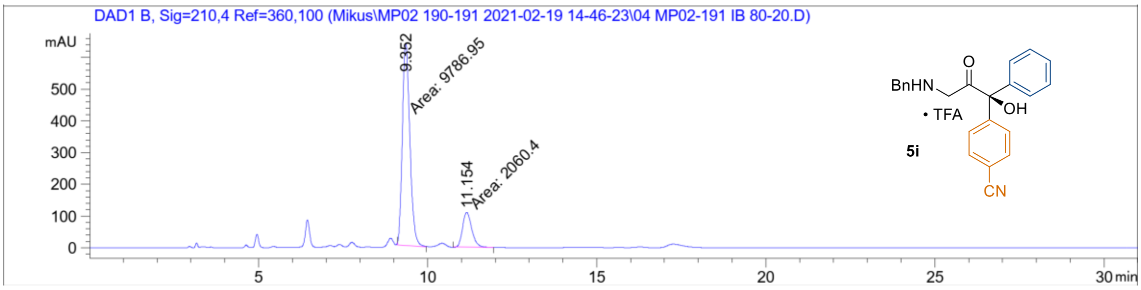

Peak #	RetTime [min]	Type	Width [min]	Area [mAU*s]	Height [mAU]	Area %
1	9.352	MM	0.2561	9786.94922	636.95319	82.6088
2	11.154	MM	0.3134	2060.40112	109.57468	17.3912

Chiral HPLC Daicel Chiralpak IB N-5 column: 95:5 hexane/IPA, flow rate 1 mL/min, λ = 210 nm

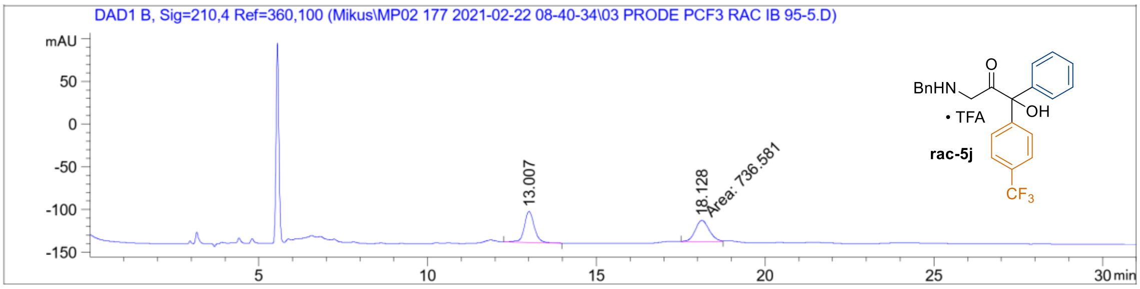

Peak #	RetTime [min]	Type	Width [min]	Area [mAU*s]	Height [mAU]	Area %
1	13.007	BB	0.3149	750.35248	36.34296	50.4631
2	18.128	MM	0.4905	736.58069	25.03076	49.5369

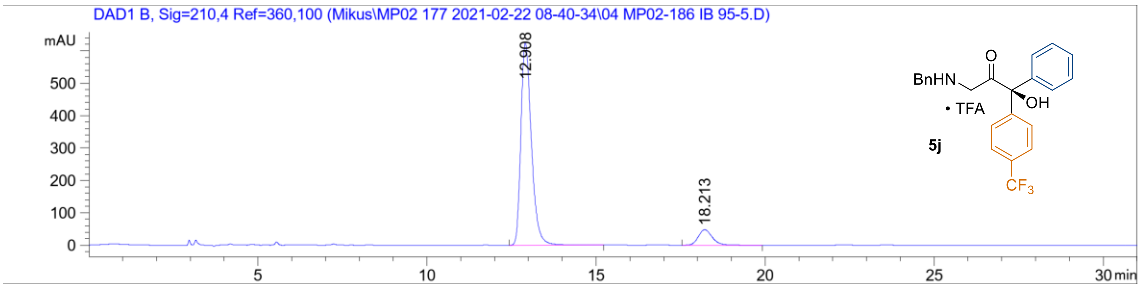

Peak #	RetTime [min]	Type	Width [min]	Area [mAU*s]	Height [mAU]	Area %
1	12.908	BB	0.3296	1.35168e4	626.87183	90.8265
2	18.213	BB	0.4375	1365.19434	47.75249	9.1735

Chiral HPLC Daicel Chiralpak IB N-5 column: 80:20 hexane/IPA, flow rate 1 mL/min, λ = 230 nm

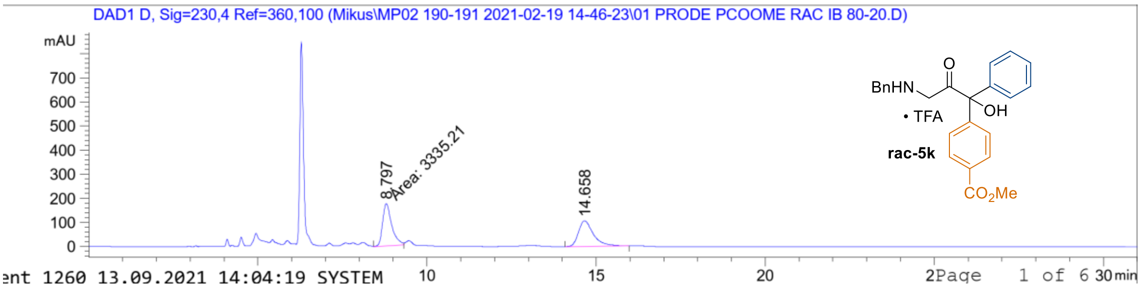

Peak #	RetTime [min]	Type	Width [min]	Area [mAU*s]	Height [mAU]	Area %
1	8.797	MM	0.3153	3335.20947	176.27040	50.3951
2	14.658	BB	0.4614	3282.91919	106.56699	49.6049

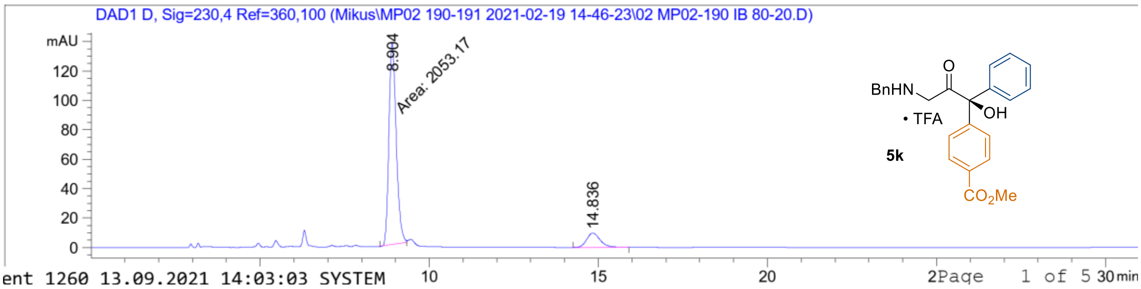

Peak #	RetTime [min]	Type	Width [min]	Area [mAU*s]	Height [mAU]	Area %
1	8.904	MM	0.2585	4416.37793	284.73508	89.6069
2	14.837	MM	0.4339	512.23486	19.67343	10.3931

Chiral HPLC Daicel Chiralpak IB N-5 column: 95:5 hexane/IPA, flow rate 1 mL/min, λ = 230 nm

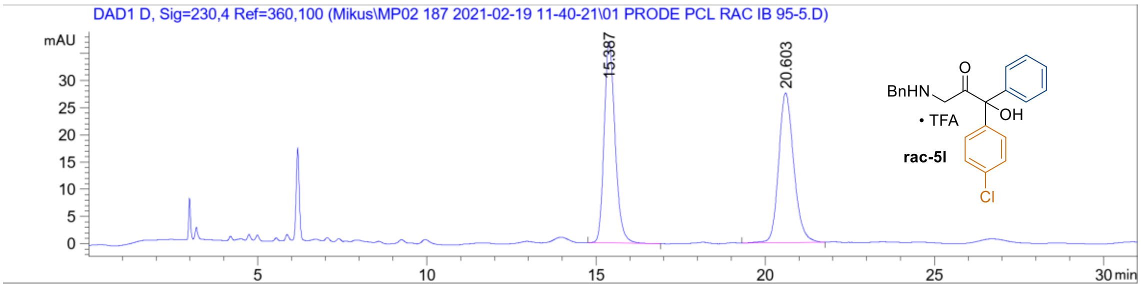

Peak #	RetTime [min]	Type	Width [min]	Area [mAU*s]	Height [mAU]	Area %
1	15.387	BB	0.3445	831.17950	36.93921	49.1924
2	20.603	BB	0.4785	858.47180	27.47680	50.8076

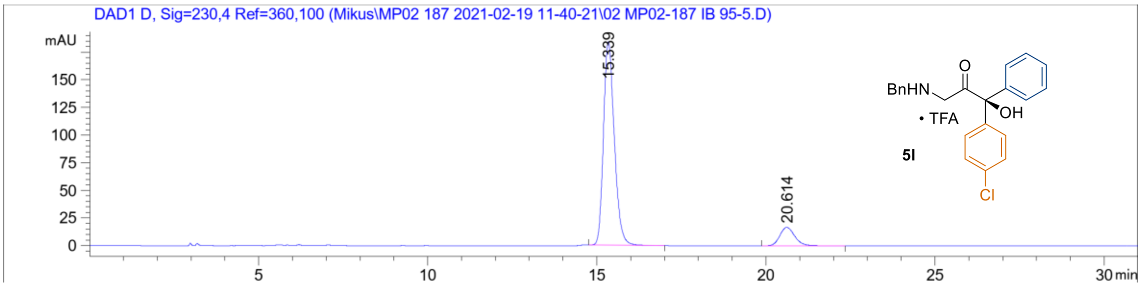

Peak #	RetTime [min]	Type	Width [min]	Area [mAU*s]	Height [mAU]	Area %
1	15.339	BB	0.3438	4097.63721	183.98062	88.8224
2	20.614	BB	0.4707	515.65594	16.77213	11.1776

Chiral HPLC Daicel Chiralpak IB N-5 column: 95:5 hexane/IPA, flow rate 1 mL/min, λ = 230 nm

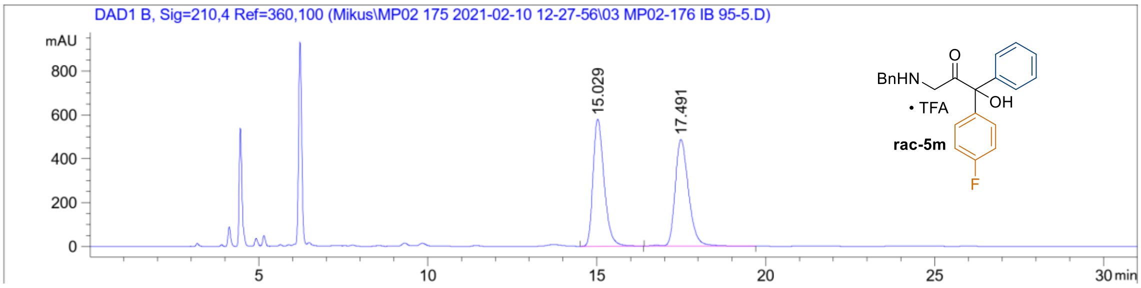

Peak #	RetTime [min]	Type	Width [min]	Area [mAU*s]	Height [mAU]	Area %
1	15.029	BB	0.3499	1.32036e4	579.21368	49.5791
2	17.491	VB R	0.4224	1.34277e4	486.39740	50.4209

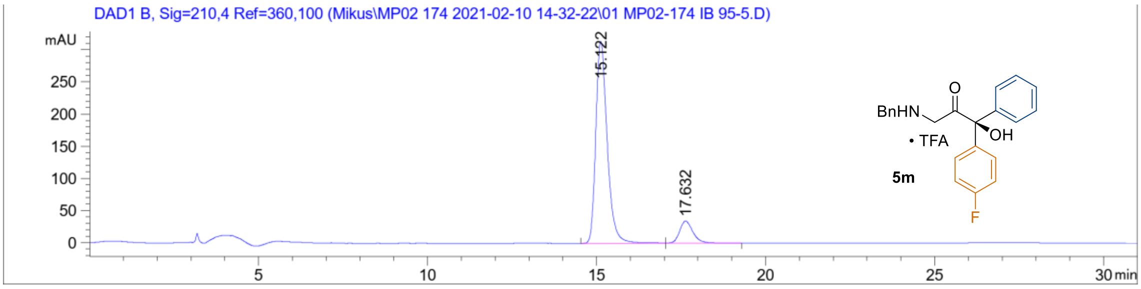

Peak #	RetTime [min]	Type	Width [min]	Area [mAU*s]	Height [mAU]	Area %
1	15.122	BB	0.3579	7318.53662	314.07071	88.6898
2	17.632	BB	0.4189	933.29639	34.14636	11.3102

Chiral HPLC Daicel Chiralpak IB N-5 column: 95:5 hexane/IPA, flow rate 1 mL/min, λ = 230 nm

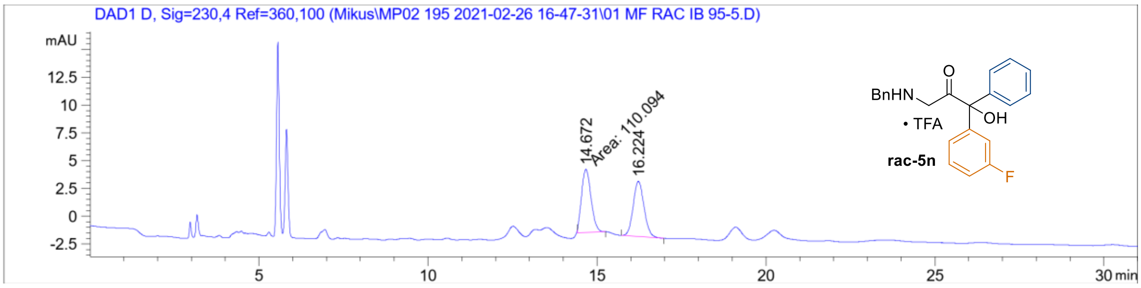

Peak #	RetTime [min]	Type	Width [min]	Area [mAU*s]	Height [mAU]	Area %
1	14.672	MM	0.3232	110.09368	5.67651	49.8366
2	16.224	BB	0.3464	110.81545	4.96421	50.1634

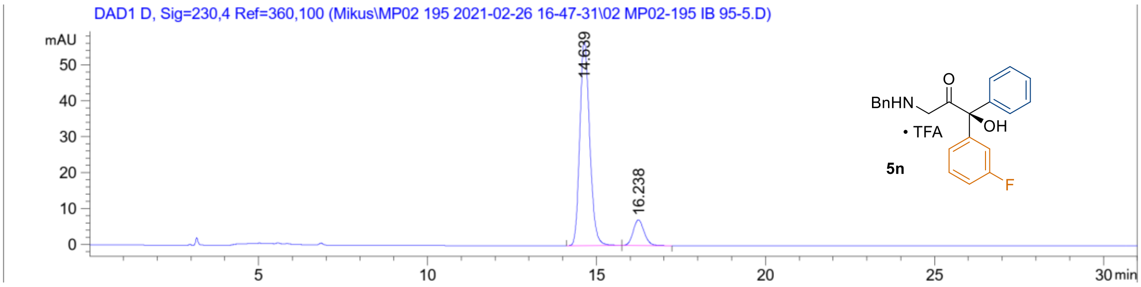

Peak #	RetTime [min]	Type	Width [min]	Area [mAU*s]	Height [mAU]	Area %
1	14.639	BB	0.3128	1155.04785	56.90836	87.8318
2	16.238	BB	0.3474	160.02008	7.14141	12.1682

Chiral HPLC Daicel Chiralpak IB N-5 column: 95:5 hexane/IPA, flow rate 1 mL/min, λ = 210 nm

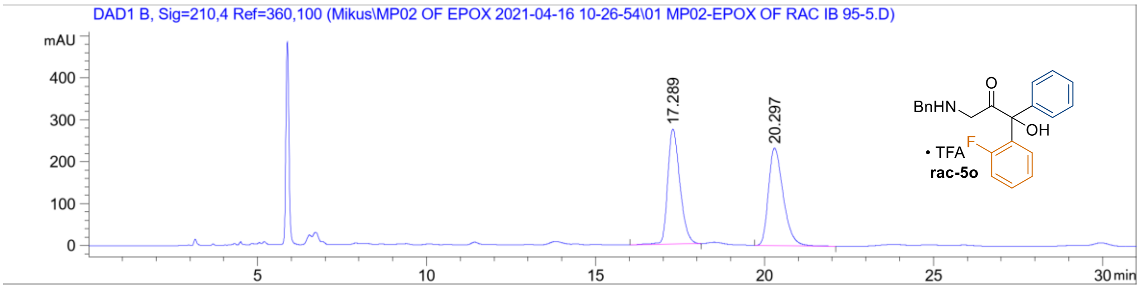

Peak #	RetTime [min]	Type	Width [min]	Area [mAU*s]	Height [mAU]	Area %
1	17.289	BB	0.3881	6928.95020	274.83313	49.5197
2	20.297	BB	0.4658	7063.37305	232.94539	50.4803

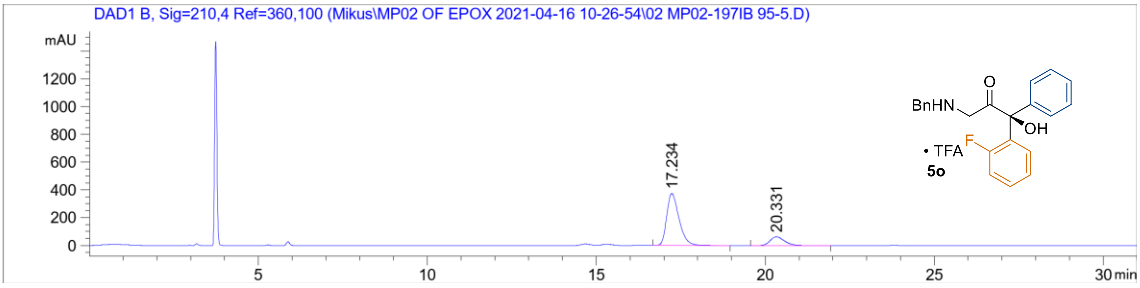

Peak #	RetTime [min]	Type	Width [min]	Area [mAU*s]	Height [mAU]	Area %
1	17.234	BB	0.3895	9447.39258	373.02264	83.1922
2	20.331	BB	0.4605	1908.71057	63.52932	16.8078

Chiral HPLC Daicel Chiralpak IB N-5 column: 90:10 hexane/IPA, flow rate 1 mL/min, λ = 254 nm

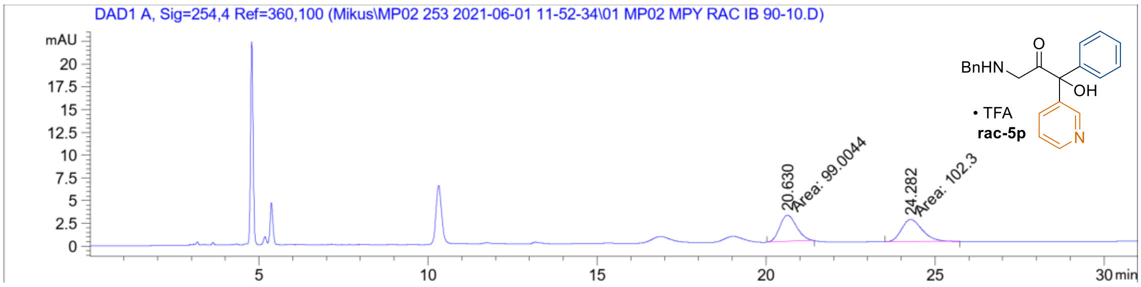

Peak #	RetTime [min]	Type	Width [min]	Area [mAU*s]	Height [mAU]	Area %
1	20.630	MM	0.5746	99.00439	2.87148	49.1815
2	24.282	MM	0.7023	102.29982	2.42767	50.8185

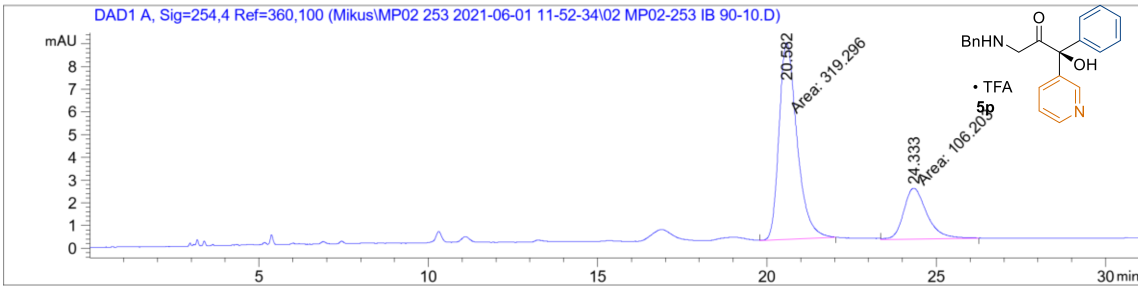

Peak #	RetTime [min]	Type	Width [min]	Area [mAU*s]	Height [mAU]	Area %
1	20.582	MM	0.6144	319.29553	8.66133	75.0404
2	24.333	MM	0.7902	106.20279	2.23993	24.9596

H.4.Product modifications

Chiral HPLC Daicel Chiralpak IA column: 95:5 hexane/IPA, flow rate 1 mL/min, λ = 230 nm

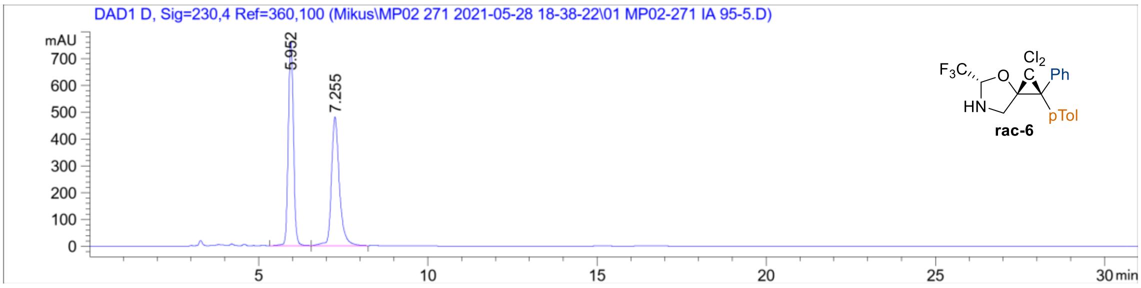

Peak #	RetTime [min]	Type	Width [min]	Area [mAU*s]	Height [mAU]	Area %
1	5.952	BB	0.1684	8104.11230	759.99463	50.8437
2	7.255	BB	0.2464	7835.14746	480.01761	49.1563

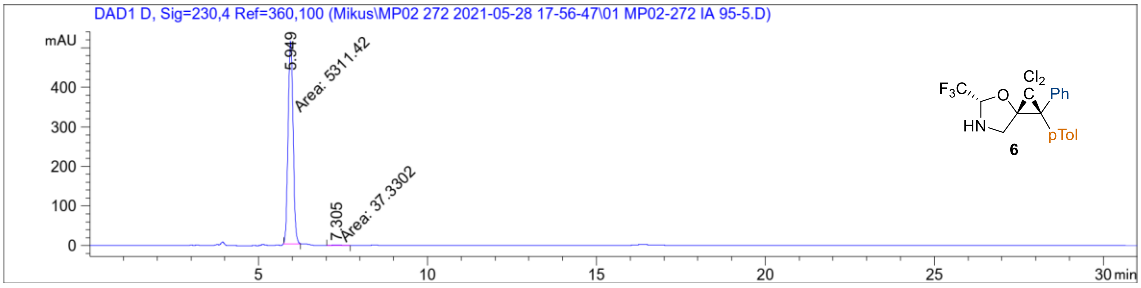

Peak #	RetTime [min]	Type	Width [min]	Area [mAU*s]	Height [mAU]	Area %
1	5.949	MM	0.1729	5311.42236	512.11102	99.3021
2	7.305	MM	0.3229	37.33020	1.92671	0.6979

Chiral HPLC Daicel Chiralpak IB N-5 column: 80:20 hexane/IPA, flow rate 1 mL/min, λ = 210 nm

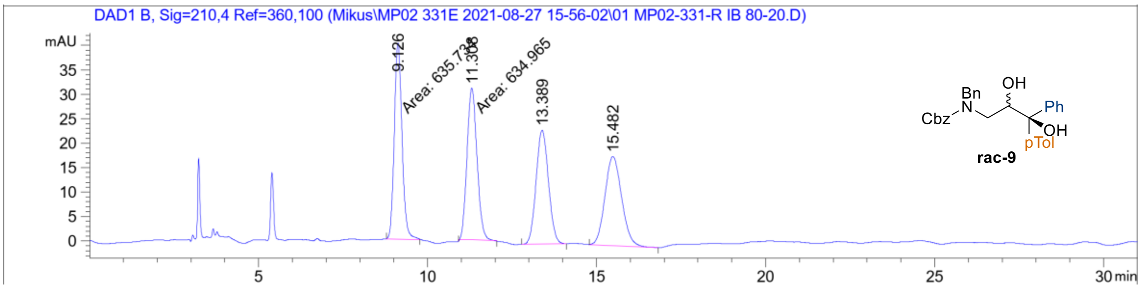

Peak #	RetTime [min]	Type	Width [min]	Area [mAU*s]	Height [mAU]	Area %
1	9.126	MM	0.2646	635.73364	40.05125	25.5716
2	11.308	MM	0.3413	634.96527	31.00769	25.5407
3	13.389	BB	0.4002	590.11926	23.24619	23.7368
4	15.482	BB	0.5305	625.27570	18.21644	25.1509

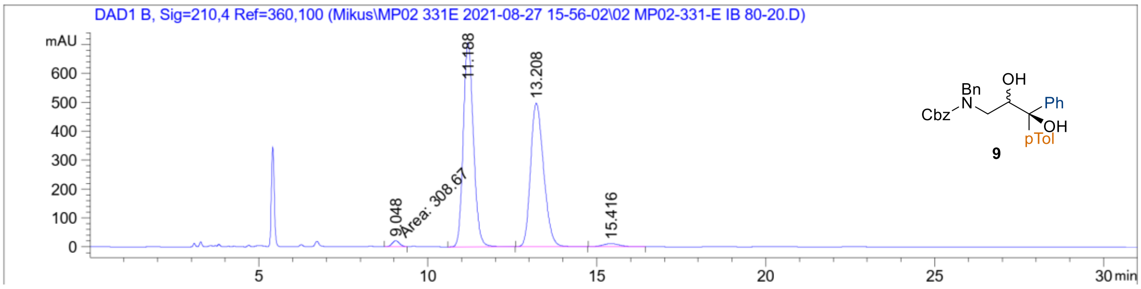

Peak #	RetTime [min]	Type	Width [min]	Area [mAU*s]	Height [mAU]	Area %
1	9.048	MM	0.2469	308.67029	20.83468	1.0743
2	11.188	BB	0.3230	1.47280e4	707.00659	51.2589
3	13.208	BB	0.4170	1.33348e4	497.08673	46.4101
4	15.416	BB	0.5330	361.08643	10.61054	1.2567

Chiral HPLC Daicel Chiralpak IB N-5 column: 80:20 hexane/IPA, flow rate 1 mL/min, λ = 210 nm

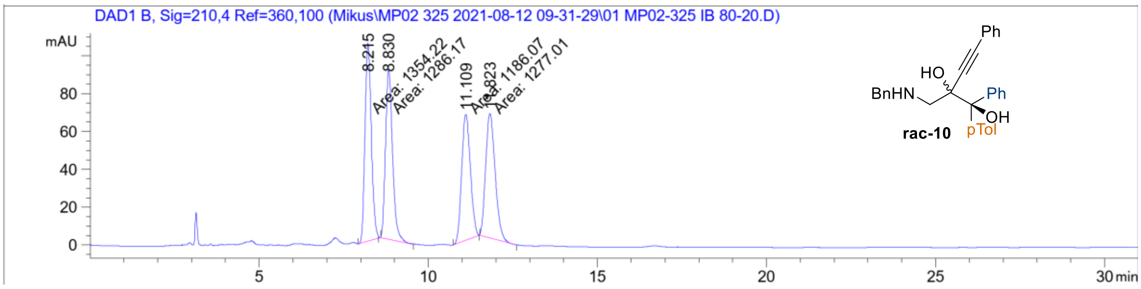

Peak #	RetTime [min]	Type	Width [min]	Area [mAU*s]	Height [mAU]	Area %
1	8.215	MM	0.2156	1354.22290	104.66690	26.5353
2	8.830	MM	0.2392	1286.17102	89.60546	25.2019
3	11.109	MM	0.2976	1186.07043	66.43127	23.2404
4	11.823	MM	0.3242	1277.01367	65.64647	25.0224

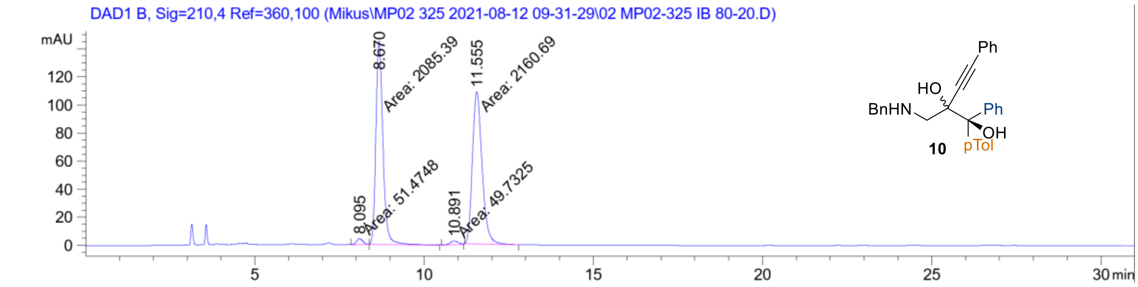

Peak #	RetTime [min]	Type	Width [min]	Area [mAU*s]	Height [mAU]	Area %
1	8.095	MM	0.2064	51.47475	4.15712	1.1841
2	8.670	MM	0.2400	2085.38794	144.80000	47.9699
3	10.891	MM	0.3056	49.73249	2.71257	1.1440
4	11.555	MM	0.3321	2160.69238	108.44670	49.7021
